# Genome-wide analysis of the *cellulose synthase-like (Csl)* gene family in bread wheat (*Triticum aestivum* L.)

**DOI:** 10.1186/s12870-017-1142-z

**Published:** 2017-11-03

**Authors:** Simerjeet Kaur, Kanwarpal S. Dhugga, Robin Beech, Jaswinder Singh

**Affiliations:** 1 Department of Plant Science, McGill University, Sainte Anne de Bellevue, QC, Canada; 2 International Maize and Wheat Improvement Center (CIMMYT), El Batán, Texcoco, Estado de México Mexico; 3 Institute of Parasitology, McGill University, Sainte Anne de Bellevue, Montreal, QC Canada

**Keywords:** Arabinoxylan, Bioenergy, Biofuels, Cell wall, Cellulose, *CesA*, *Csl*, Glucuronoarabinoxylan, Mixed-linked glucan, Wheat

## Abstract

**Background:**

Hemicelluloses are a diverse group of complex, non-cellulosic polysaccharides, which constitute approximately one-third of the plant cell wall and find use as dietary fibres, food additives and raw materials for biofuels. Genes involved in hemicellulose synthesis have not been extensively studied in small grain cereals.

**Results:**

In efforts to isolate the sequences for the *cellulose synthase-like* (*Csl*) gene family from wheat, we identified 108 genes (hereafter referred to as *TaCsl*). Each gene was represented by two to three homeoalleles, which are named as *TaCslXY_ZA*, *TaCslXY_ZB*, or *TaCslXY_ZD*, where X denotes the *Csl* subfamily, Y the gene number and Z the wheat chromosome where it is located. A quarter of these genes were predicted to have 2 to 3 splice variants, resulting in a total of 137 putative translated products. Approximately 45% of *TaCsl* genes were located on chromosomes 2 and 3. Sequences from the subfamilies C and D were interspersed between the dicots and grasses but those from subfamily A clustered within each group of plants. Proximity of the dicot-specific subfamilies B and G, to the grass-specific subfamilies H and J, respectively, points to their common origin. In silico expression analysis in different tissues revealed that most of the genes were expressed ubiquitously and some were tissue-specific. More than half of the genes had introns in phase 0, one-third in phase 2, and a few in phase 1.

**Conclusion:**

Detailed characterization of the wheat *Csl* genes has enhanced the understanding of their structural, functional, and evolutionary features. This information will be helpful in designing experiments for genetic manipulation of hemicellulose synthesis with the goal of developing improved cultivars for biofuel production and increased tolerance against various stresses.

**Electronic supplementary material:**

The online version of this article (10.1186/s12870-017-1142-z) contains supplementary material, which is available to authorized users.

## Background

Plant cell wall consists of three main polysaccharide fractions: cellulose, hemicellulose, and pectin, with lignin and proteins being the other two constituents. Grass walls contain mainly two of the three polysaccharide fractions with pectin being a rather minor constituent. Hemicelluloses are plant cell wall matrix polysaccharides that possess diverse linear or branched structures [1, 2]. These mainly encompass 1–4-β-glucan, 1,3;1,4-β-glucan, galactan, and glucomannan in grasses [3]. In addition, glucuronoarabinoxylan is a major grass wall constituent. Because of the presence of heterogeneous substituents or other linkages on their polymer backbones, hemicelluloses are non-crystalline and can be readily hydrolysed in comparison to cellulose. These polysaccharides can interact with cellulose microfibrils through hydrogen bonds [4].

Hemicellulosic polysaccharide backbones in plants are made by *the cellulose synthase-like* (*Csl*) enzymes, which are members of a much larger superfamily of genes referred to as *glycosyltransferase 2* (*GT2*) [5]. Several other *GTs,* i.e., *xyloglucan α-1,6-xylosyltransferases* (*GT34*), *xyloglucan fucosyltransferases* (*GT37*), and *xyloglucan galactosyltransferases* (*GT47*) have been reported to be involved in the biosynthesis of xyloglucans [6]. Genes encoding Csl enzymes share sequence similarity with the *cellulose synthase A* (*CesA*) gene family known to form cellulose throughout the plant kingdom [7]. A variable number of *Csl* genes ranging from 30 to 50 have been reported from different plant species and are classified into nine subfamilies (*CslA*–*CslH* and *CslJ*) [8, 9]. Cereals generally lack *CslB* and *CslG* families. Among the remaining families, *CslA*, *CslC*, and *CslD* are conserved in all land plants, whereas *CslF*, *CslH* are restricted to grasses [10, 11]. A poorly understood subfamily, *CslJ*, has been reported in grasses as well as dicots, which contrasts with the previous claims of its occurrence only in grasses [12, 13]. Similarly, the subfamilies *CslB* and *CslG* were previously reported to be specific to dicots [14]. However, a recent report established the presence of the *CslB* subfamily in monocots as well [12]. Several of the *Csl* subfamilies have been reported to be involved in the biosynthesis of different cell wall polysaccharides. For example, subfamily *CslA* was shown to form β-1,4-mannan backbone of galactomannan and glucomannan [15, 16]. Similarly, *CslF* and *CslH* subfamilies were shown to make 1–3;1–4-β-glucan in grasses [17, 18], whereas *CslC* genes were associated with the formation of the 1–4-β-glucan backbone of a xyloglucan and some other polysaccharides [19].

Wheat is a major cereal crop grown on the largest area of arable land in the world, is second only to maize in grain production, and feeds approximately 40% of the world population [20]. It has a large genome size (~17 Gb), of which ~80–90% is repetitive [21]. Even after the complete genome sequence became available [22], *Csl* genes remain unidentified and uncharacterized in bread wheat. In general, homeologous copies of most of the genes are located on each of the three chromosomes belonging to each of the subgenomes (A, B, and D), suggesting that the number of *Csl* genes is expected to approximately three-times that of a diploid species like rice. We used publicly available resources to retrieve wheat genome sequence. Large-scale data mining was performed using the Pfam domain models for the identification of *Csl* gene family members, which are reported in this study.

## Methods

### Data sources and sequence retrieval

Wheat genome data were downloaded from the Ensembl Plants FTP server (ftp://ftp.ensemblgenomes.org/pub/current/plants/fasta/triticum_aestivum/), generated by the International Wheat Genome Sequencing Consortium (IWGSC) and converted into a local BLAST database using the UNIX pipeline. BLAST analyses (BLASTN as well as BLASTP) were performed using the stand-alone command line version of NCBI (National Center for Biotechnology Information) blast 2.2.28+ (ftp://ftp.ncbi.nih.gov/blast/executables/LATEST/), released March 19, 2013. A query file was generated from Pfam domain models; PF00535 (*GT2*) domain and PF03552 (*Cellulose_synt*) downloaded from Pfam 30.0 June 2016 release [23]. The sequences of splice variants were also retrieved from Ensembl Plants browser (http://plants.ensembl.org/Triticum_aestivum/Info/Index). Analysis of splice variants was conducted as described by Kim et al. (2007) [24]. Previously known *Csl* sequences from *Arabidopsis thaliana*, *Oryza sativa*, and *Zea mays* were downloaded from the Cell Wall Navigator database [25]. For Brachypodium, sequences were retrieved from phytomine (https://phytozome.jgi.doe.gov). Amino acid sequences of the aforementioned CSL proteins are given in Additional file 1: Figure S1.

### Blast searches for wheat homologs

All query files containing the two Pfam domain models (PF00535 and PF03552) were used to perform the BLASTn searches against the local blast database of bread wheat. All blast hits with E-value >1.0 were removed. Using cut-off E- value <1.0, all previously known *CesA* genes were retrieved. After the compilation of all the sequences below the cut-off value, CD-hit program was used to obtain non-redundant sequences. Higher cut-off E- value was used to ascertain the identification of all the genes that possessed the Pfam domains PF00535 and PF03552. These genes were further filtered through phylogenetic analysis alongwith previously known CSL proteins from Arabidopsis, Brachypodium, maize, and rice, which reflected some non-targeted genes that were removed from further analysis [26]. Phylogenetic analysis was also implemented to categorize different *Csl* sub-families. *CesA* genes were distinguished from the *Csl* genes with the CXC motif, which is diagnostic of the CesA but absent from the Csl proteins [7, 27]. Presence of the conserved domains *Cellulose_synt/GT2* was confirmed using a batch blast search at the CDD (conserved domain database) of NCBI. Homeologous genes from each of the three genomes were named *TaCslXY_ZA*, *TaCslXY_ZB*, or *TaCslXY_ZD*, where *X* denotes the *Csl* subfamily, *Y* the gene number and *Z* the wheat chromosome where it is located. Alignment of the sequences of all newly identified wheat *Csl* genes is given in Additional file 2: Figure S2.

### Protein structure and motif/domain identification

Protein sequences were downloaded from the Ensembl Plants FTP server (ftp://ftp.ensemblgenomes.org/pub/current/plants/fasta/triticum_aestivum/), developed by the International Wheat Genome Sequencing Consortium (IWGSC) [22]. Multiple protein sequence alignments were performed using Clustal Omega (http://www.ebi.ac.uk/Tools/msa/clustalo/) [28]. The resulting alignments were analysed for the presence of conserved motifs (D, D, DXD, QXXRW) of the *GT2* superfamily. Conserved patterns of aligned sequences were highlighted using the sequence manipulation suite: Color align conservation (http://www.bioinformatics.org/sms2/color_align_cons.html) [29]. The conserved domains were predicted using CCD database (http://www.ncbi.nlm.nih.gov/Structure/cdd/cdd.shtml) [22, 30, 31]. Wheat *Csl* genes were named based on their sequence identity, coverage, presence of conserved domains and motifs similar to those of the previously identified rice *Csl* genes. The number of genes in in a subfamily exceeded that of rice, the additional genes were given new names. Because of the resemblance of *CslD* genes with *CesA* genes and their probable role in cellulose synthesis, we specifically focused on the *TaCslD* subfamily. Gene structures and intron evolution of *TaCslD* members were predicted using the gene structure display server 2.0 (http://gsds.cbi.pku.edu.cn/) using the genomic and cDNA sequences.

### Evolutionary relationships of *Csl* genes

A total of 215 CSL proteins from Arabidopsis, maize, rice and wheat were aligned using MAAFT (v1.3.6) [32]. Sequences that did not extend over the conserved core region were removed. Positions where more than 40% of the sequences contained a gap were also removed. The phylogeny and 1000 bootstrap replications of these sequences was inferred using Seqboot (v3.696) [33] and FastTree (v2.1.10) implemented on the Guillimin cluster [34].

The phylogeny of the *CslD* subfamily was also determined separately from Arabidopsis, Brachypodium, maize, rice and wheat. For phylogenetic analysis, the amino acid sequences of CSL proteins were aligned using MUSCLE and their evolutionary history was inferred using Neighbor-Joining methods [35]. The tree was drawn to scale, with branch lengths being equivalent to the evolutionary distances used to infer the phylogenetic tree. Evolutionary distances were computed with a Poisson correction and are given as the number of amino acid substitutions per site. The rate of variation among sites was modeled with a gamma distribution (shape parameter = 1) and all positions containing gaps and missing data were removed. Evolutionary analyses were conducted in MEGA6 [36].

### RNA-seq expression analysis

Publicly available RNA-seq data generated from bread wheat (var. Chinese Spring) was used to study the expression of newly identified wheat *Csl* genes. The data were compiled from five different wheat tissues (spike, leaf, stem, root, and grain) collected at seedling, vegetative and reproductive stages of development [37]. The relative expression of each *TaCsl* subfamily was presented as a heat map generated from the relative abudnace of transcripts (per 10 million reads) for each gene using wheat expression browser powered by expVIP (http://www.wheat-expression.com).

## Results

### Identification and classification of *Csl* gene family members in bread wheat

Database searches for bread wheat using conserved pfam motifs PF00535 and PF03552, which are specific to the *GT2* superfamily, resulted in the identification of 108 cellulose synthase-like (*TaCsl*) genes (Table 1). Two to three homeologous copies of each gene from the A, B and D genomes were common. The identified genes were named following the nomenclature of rice, which shares synteny with wheat. To avoid the complexity of the nomenclature, a suffix corresponding to the chromosome number and the specific wheat genome identifier (A, B, or D) has been used for each gene name [7]. For example, the first gene of subfamily *CslA*; *CslA1* on the long arm of chromosome 1 of genomes A, B, and D is named as *TaCslA1_1AL*, *TaCslA1_1BL*, and *TaCslA1_1DL,* respectively.

**Table 1:** Homeologous copies of the bread wheat *Csl* genes

No.	Ensembl ID	Gene name	Corresponding gene in rice
1	TRIAE_CS42_6BS_TGACv1_513375_AA1639370.1	*TaCslA1_6BS*	*CslA1*
2	TRIAE_CS42_6AS_TGACv1_485966_AA1554960.1	*TaCslA1_6AS*	*CslA1*
3	TRIAE_CS42_2AL_TGACv1_093375_AA0278800.1	*TaCslA2_2AL*	*CslOS09G39920*
4	TRIAE_CS42_2BL_TGACv1_129747_AA0394630.1	*TaCslA2_2BL*	*CslOS09G39920*
5	TRIAE_CS42_2DL_TGACv1_160461_AA0550770.1	*TaCslA2_2DL*	*CslOS09G39920*
6	TRIAE_CS42_1AS_TGACv1_019142_AA0061550.1	*TaCslA2_1AS*	*CslOS09G39920*
7	TRIAE_CS42_7BS_TGACv1_592860_AA1945380.1	*TaCslA3_7BS*	*CslA3*
8	TRIAE_CS42_7DS_TGACv1_623146_AA2050070.1	*TaCslA3_7DS*	*CslA3*
9	TRIAE_CS42_7AS_TGACv1_569190_AA1809650.1	*TaCslA3_7AS*	*CslA3*
10	TRIAE_CS42_6DS_TGACv1_543811_AA1744360.1	*TaCslA4_6DS*	*CslA10/4/2*
11	TRIAE_CS42_6AS_TGACv1_487286_AA1569690.1	*TaCslA4_6AS*	*CslA10/4/2*
12	TRIAE_CS42_6BS_TGACv1_513376_AA1639390.1	*TaCslA4_6BS*	*CslA10/4/2*
13	TRIAE_CS42_2BS_TGACv1_146583_AA0468630.1	*TaCslA5_2BS*	*CslA5/7*
14	TRIAE_CS42_2AS_TGACv1_113418_AA0355820.1	*TaCslA5_2AS*	*CslA5/7*
15	TRIAE_CS42_2DS_TGACv1_177473_AA0578070.1	*TaCslA5_2DS*	*CslA5/7*
16	TRIAE_CS42_3DL_TGACv1_249033_AA0835410.1	*TaCslA6_3DL*	*CslA11*
17	TRIAE_CS42_3B_TGACv1_221079_AA0729630.1	*TaCslA6_3B*	*CslA11*
18	TRIAE_CS42_3AL_TGACv1_197519_AA0666560.1	*TaCslA6_3AL*	*CslA11*
19	TRIAE_CS42_2AS_TGACv1_113300_AA0354190.1	*TaCslA7_2AS*	*CslA5/7*
20	TRIAE_CS42_2DS_TGACv1_177798_AA0584795.1	*TaCslA7_2DS*	*CslA5/7*
21	TRIAE_CS42_3B_TGACv1_220828_AA0720500.1	*TaCslA8_3B*	*CslA11*
22	TRIAE_CS42_3DS_TGACv1_273022_AA0927600.1	*TaCslA8_3DS*	*CslA11*
23	TRIAE_CS42_U_TGACv1_642146_AA2112270.1	*TaCslA9*	*CslA9*
24	TRIAE_CS42_7BL_TGACv1_579090_AA1903960.1	*TaCslA9_7BL*	*CslA9*
25	TRIAE_CS42_7AL_TGACv1_558725_AA1795700.1	*TaCslA9_7AL*	*CslA9*
26	TRIAE_CS42_U_TGACv1_642146_AA2112290.1	*TaCslA10*	*CslA9*
27	TRIAE_CS42_7DL_TGACv1_602617_AA1962870.1	*TaCslA10_7DL*	*CslA9*
28	TRIAE_CS42_7AL_TGACv1_557254_AA1778850.1	*TaCslA10_7AL*	*CslA9*
29	TRIAE_CS42_7BL_TGACv1_578444_AA1895100.1	*TaCslA10_7BL*	*CslA9*
30	TRIAE_CS42_3AS_TGACv1_210508_AA0674280.1	*TaCslA11_3AS*	*CslA11*
31	TRIAE_CS42_3DS_TGACv1_272005_AA0912960.1	*TaCslA11_3DS*	*CslA11*
32	TRIAE_CS42_3B_TGACv1_223332_AA0780350.1	*TaCslA11_3B*	*CslA11*
33	TRIAE_CS42_3DL_TGACv1_251593_AA0882850.1	*TaCslC1_3DL*	*CslC1*
34	TRIAE_CS42_3AL_TGACv1_197197_AA0665370.1	*TaCslC1_3AL*	*CslC1*
35	TRIAE_CS42_3DS_TGACv1_271926_AA0910940.1	*TaCslC3_3DS*	*CslC3*
36	TRIAE_CS42_3B_TGACv1_220758_AA0718310.1	*TaCslC3_3B*	*CslC3*
37	TRIAE_CS42_3AS_TGACv1_211225_AA0686890.1	*TaCslC3_3AS*	*CslC3*
38	TRIAE_CS42_1DL_TGACv1_061928_AA0205730.1	*TaCslC7_1DL*	*CslC7*
39	TRIAE_CS42_1BL_TGACv1_030750_AA0099830.1	*TaCslC7_1BL*	*CslC7*
40	TRIAE_CS42_1AL_TGACv1_001272_AA0028090.1	*TaCslC7_1AL*	*CslC7*
41	TRIAE_CS42_1DL_TGACv1_062162_AA0209740.1	*TaCslC9_1DL*	*CslC10/9*
42	TRIAE_CS42_1BL_TGACv1_030501_AA0092480.1	*TaCslC9_1BL*	*CslC10/9*
43	TRIAE_CS42_5BL_TGACv1_404820_AA1311790.1	*TaCslC10_5BL*	*CslC10/9*
44	TRIAE_CS42_5DL_TGACv1_435778_AA1454840.1	*TaCslC10_5DL*	*CslC10/9*
45	TRIAE_CS42_5AL_TGACv1_374268_AA1195590.1	*TaCslC10_5AL*	*CslC10/9*
46	TRIAE_CS42_1BL_TGACv1_030586_AA0094860.1	*TaCslD1_1BL*	*CslD1*
47	TRIAE_CS42_1AL_TGACv1_001700_AA0034150.1	*TaCslD1_1AL*	*CslD1*
48	TRIAE_CS42_1DL_TGACv1_063091_AA0223780.1	*TaCslD1_1DL*	*CslD1*
49	TRIAE_CS42_2BS_TGACv1_148683_AA0494520.1	*TaCslD3_2BS*	*CslD3*
50	TRIAE_CS42_2DS_TGACv1_177279_AA0572180.1	*TaCslD3_2DS*	*CslD3*
51	TRIAE_CS42_2AS_TGACv1_114244_AA0365360.1	*TaCslD3_2AS*	*CslD3*
52	TRIAE_CS42_1BS_TGACv1_049706_AA0160220.1	*TaCslD4_1BS*	*CslD4*
53	TRIAE_CS42_5BS_TGACv1_425241_AA1392650.1	*TaCslD4_5BS*	*CslD4*
54	TRIAE_CS42_5DS_TGACv1_457675_AA1488780.1	*TaCslD4_5DS*	*CslD4*
55	TRIAE_CS42_7BL_TGACv1_577301_AA1871610.1	*TaCslD5_7BL*	*CslD5*
56	TRIAE_CS42_7AL_TGACv1_559436_AA1799630.1	*TaCslD5_7AL*	*CslD5*
57	TRIAE_CS42_7DL_TGACv1_603510_AA1985050.1	*TaCslD5_7DL*	*CslD5*
58	TRIAE_CS42_5DL_TGACv1_433536_AA1415830.1	*TaCslE1_5DL*	*CslE6/1*
59	TRIAE_CS42_5BL_TGACv1_406235_AA1342600.1	*TaCslE1_5BL*	*CslE6/1*
60	TRIAE_CS42_6DL_TGACv1_526558_AA1687090.1	*TaCslE2_6DL*	*CslE2*
61	TRIAE_CS42_6AL_TGACv1_471004_AA1500600.1	*TaCslE2_6AL*	*CslE2*
62	TRIAE_CS42_6BL_TGACv1_499967_AA1596110.1	*TaCslE2_6BL*	*CslE2*
63	TRIAE_CS42_U_TGACv1_683314_AA2158770.1	*TaCslE3*	*CslE6/1*
64	TRIAE_CS42_6DS_TGACv1_543277_AA1737920.1	*TaCslE4_6DS*	*CslE6/1*
65	TRIAE_CS42_5DL_TGACv1_433536_AA1415840.1	*TaCslE6_5DL*	*CslE6/1*
66	TRIAE_CS42_5BL_TGACv1_406235_AA1342610.1	*TaCslE6_5BL*	*CslE6/1*
67	TRIAE_CS42_5AL_TGACv1_376126_AA1232370.1	*TaCslE6_5AL*	*CslE6/1*
68	TRIAE_CS42_2DL_TGACv1_159781_AA0542640.1	*TaCslF1_2DL*	*CslF1/2/4*
69	TRIAE_CS42_2AL_TGACv1_094713_AA0301960.1	*TaCslF1_2AL*	*CslF1/2/4*
70	TRIAE_CS42_2DL_TGACv1_160109_AA0546890.1	*TaCslF1_2DL*	*CslF1/2/4*
71	TRIAE_CS42_2BL_TGACv1_130934_AA0420130.1	*TaCslF1_2BL*	*CslF1/2/4*
72	TRIAE_CS42_7BL_TGACv1_580651_AA1914920.1	*TaCslF2_7BL*	*CslF1/2/4*
73	TRIAE_CS42_7AL_TGACv1_557532_AA1782680.1	*TaCslF2_7AL*	*CslF1/2/4*
74	TRIAE_CS42_7DL_TGACv1_602590_AA1961740.1	*TaCslF2_7DL*	*CslF1/2/4*
75	TRIAE_CS42_2AS_TGACv1_113659_AA0359050.1	*TaCslF3_2AS*	*CslF3*
76	TRIAE_CS42_2DS_TGACv1_177641_AA0581710.1	*TaCslF3_2DS*	*CslF3*
77	TRIAE_CS42_2BS_TGACv1_148608_AA0494060.1	*TaCslF3_2BS*	*CslF3*
78	TRIAE_CS42_2BS_TGACv1_146146_AA0456710.1	*TaCslF4_2BS*	*CslF1/2/4*
79	TRIAE_CS42_2DS_TGACv1_179076_AA0604160.1	*TaCslF4_2DS*	*CslF1/2/4*
80	TRIAE_CS42_2DS_TGACv1_178985_AA0603230.1	*TaCslF5_2DS*	*CslF3*
81	TRIAE_CS42_2AS_TGACv1_112790_AA0345230.1	*TaCslF5_2AS*	*CslF3*
82	TRIAE_CS42_2BS_TGACv1_148027_AA0489970.1	*TaCslF5_2BS*	*CslF3*
83	TRIAE_CS42_7BL_TGACv1_577473_AA1876170.1	*TaCslF6_7BL*	*CslF6*
84	TRIAE_CS42_7AL_TGACv1_555973_AA1751470.1	*TaCslF6_7AL*	*CslF6*
85	TRIAE_CS42_7DL_TGACv1_607937_AA2011180.1	*TaCslF6_7DL*	*CslF6*
86	TRIAE_CS42_5BL_TGACv1_409916_AA1366600.1	*TaCslF7_5BL*	*CslF7*
87	TRIAE_CS42_5DL_TGACv1_433902_AA1424880.1	*TaCslF7_5DL*	*CslF7*
88	TRIAE_CS42_5AL_TGACv1_374191_AA1193100.1	*TaCslF7_5AL*	*CslF7*
89	TRIAE_CS42_2BS_TGACv1_148916_AA0495580.1	*TaCslF8_2BS*	*CslF8*
90	TRIAE_CS42_2DS_TGACv1_178471_AA0596060.1	*TaCslF8_2DS*	*CslF8*
91	TRIAE_CS42_2AS_TGACv1_112322_AA0335280.1	*TaCslF8_2AS*	*CslF8*
92	TRIAE_CS42_2AS_TGACv1_112322_AA0335290.1	*TaCslF9_2AS*	*CslF9*
93	TRIAE_CS42_2BS_TGACv1_147667_AA0486240.1	*TaCslF9_2BS*	*CslF9*
94	TRIAE_CS42_2DS_TGACv1_177329_AA0573830.1	*TaCslF9_2DS*	*CslF9*
95	TRIAE_CS42_U_TGACv1_641498_AA2096480.1	*TaCslF10*	*CslF9*
96	TRIAE_CS42_1BS_TGACv1_049866_AA0163180.1	*TaCslF10_1BS*	*CslF9*
97	TRIAE_CS42_2AL_TGACv1_094351_AA0296300.1	*TaCslH1_2AL*	*Cslh1/2*
98	TRIAE_CS42_2DL_TGACv1_158387_AA0517170.1	*TaCslH1_2DL*	*CslH1/2*
99	TRIAE_CS42_2BL_TGACv1_129372_AA0380770.1	*TaCslH1_2BL*	*CslH1/2*
100	TRIAE_CS42_3B_TGACv1_221049_AA0728260.1	*TaCslH2_3B*	*Csl*
101	TRIAE_CS42_3DS_TGACv1_273502_AA0931770.1	*TaCslH2_3DS*	*Csl*
102	TRIAE_CS42_3DS_TGACv1_271739_AA0907200.1	*TaCslH3_3DS*	*Csl*
103	TRIAE_CS42_3AS_TGACv1_212952_AA0704280.1	*TaCslH3_3AS*	*CslH3*
104	TRIAE_CS42_3B_TGACv1_222234_AA0760340.1	*TaCslH3_3B*	*Csl*
105	TRIAE_CS42_3DS_TGACv1_272297_AA0918580.1	*TaCslJ1_3DS*	*Csl*
106	TRIAE_CS42_3AS_TGACv1_210908_AA0681280.1	*TaCslJ1_3AS*	*Csl*
107	TRIAE_CS42_3B_TGACv1_221705_AA0747940.1	*TaCslJ2_3B*	*Csl*
108	TRIAE_CS42_3DS_TGACv1_272756_AA0924850.1	*TaCslJ2_3DS*	*Csl*

**Fig. 1:**
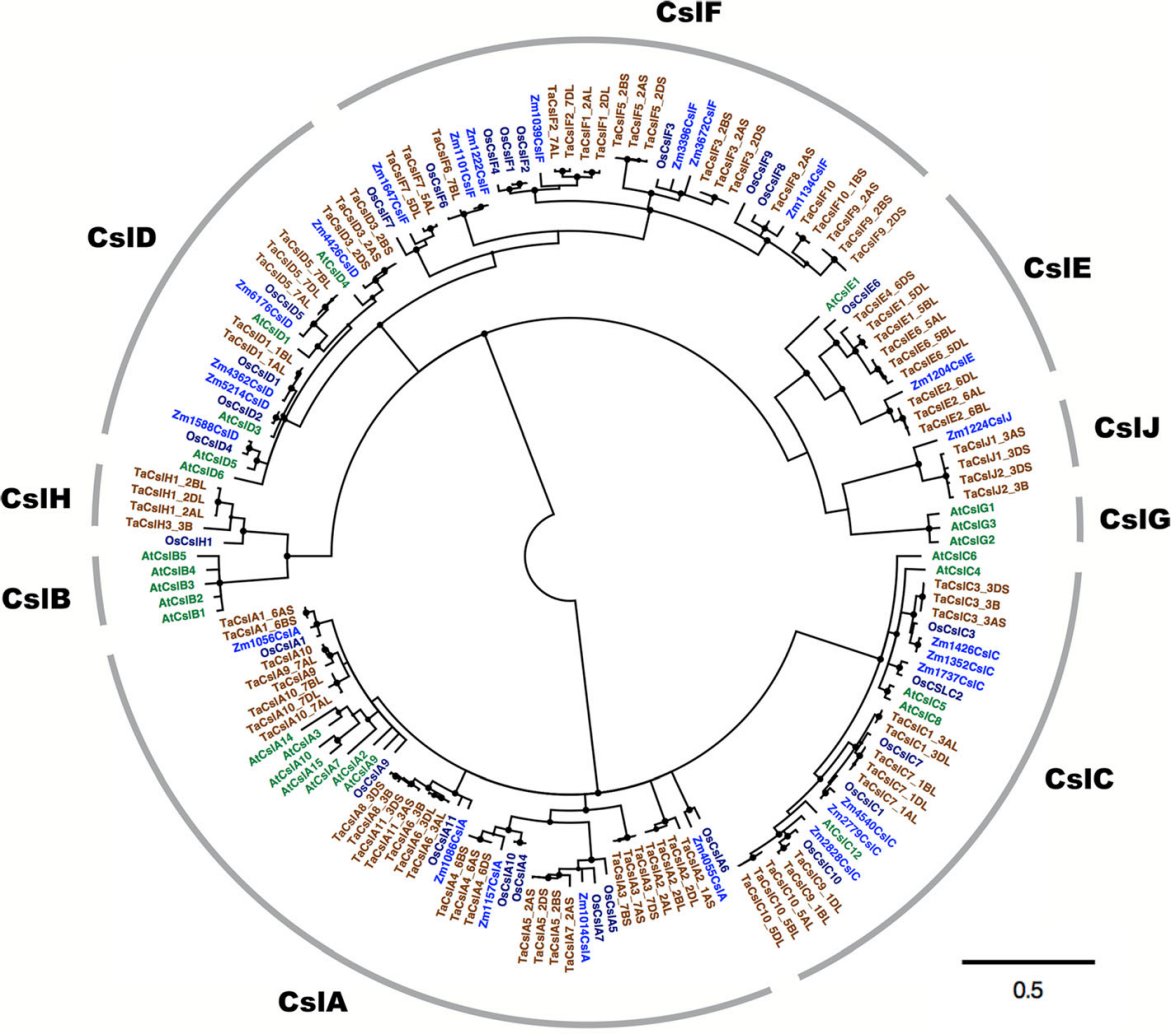
An unrooted maximum likelihood phylogenetic tree of the *Cellulose synthase-like* (*Csl*) gene family from Arabidopsis, maize, rice and wheat using FastTree (v2.1.10) according to Price et al. (35). Nodes with more than 70% support from 1000 bootstrap replications were considered significant and indicated by a black circle. Different colors represent CSL proteins from different species. The scale bar indicates a radial distance equal to 0.5 amino acid substitutions per site. To keep the gene family nomenclature uniform, maize gene models from Gramene were renamed as follows: Zm, first four digits of the locus number, Csl, and the class identifier as described in Schwerdt et al. (9)

**Fig. 2:**
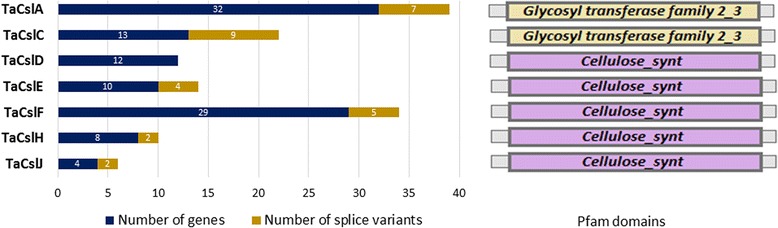
Distribution of the *TaCsl* genes and their splice variants in seven subfamilies and their corresponding pfam domains used to identify *TaCsl* gene family members

An unrooted neighbor-joining (NJ) tree for the 215 derived Csl proteins from Arabidopsis, maize, rice and wheat is shown in Fig. 1. TaCsl proteins grouped into seven subfamilies: TaCslA (32 proteins*)*, TaCslC (13 proteins), TaCslD (12 proteins), TaCslE (10 proteins), TaCslF (29 proteins), TaCslH (8 proteins), and TaCslJ (4 proteins) (Fig. 2). The TaCslA and TaCslC subfamilies were closely related as shown by their taxonomic distribution and phylogenies. As expected, these subfamilies were conserved across the plant species. Although TaCslD is present in all the plant species whereas TaCslF is specific to grasses, their proximity to each other suggests a common origin [12]. Among the sequences common to both dicots and grasses, subfamily CslA appeared to be the most divergent between these two groups of plants. Whereas the sequences within the subfamilies CslC and CslD were interspersed between Arabidopsis and grasses, all the subfamily CslA sequences of Arabidopsis clustered together, separately from the grass CslA sequences. Proximity of the CslB and CslH subfamilies points to their common origin before the separation of grasses from dicots. Similarly, CslG and CslJ apparently had a common origin.

### Splice variants of *Csl* genes

Twenty two of the 108 genes appeared to encode two or more proteins because of the presence of alternative splicing sites, as predicted by Ensembl database, which would result in 137 probable Csl protein products (Table 2). Splice variants were predicted in all the subfamilies of the *TaCsl* genes except *TaCslD* (Table 2). In the subfamily *TaCslA*, 6 genes alternatively spliced to form 13 putative proteins whereas in the subfamily *TaCslC*, 5 genes were alternatively spliced resulting in 14 putative proteins. Similarly, for the subfamilies *TaCslE* and *TaCslF,* alternative splicing resulted in 7 and 10 splice variants, respectively. Alternative splicing of 1 and 2 genes respectively generated 3 and 4 putative proteins in the *CslH* and *CslJ* subfamilies (Fig. 2). More than half (51%) of the splice variants stemmed from exon skipping, ~24% from alternative 5′ and 3′ splice sites, and the rest, ~24%, from intron retention (Table 2).

**Table 2:** Splice variants of the bread wheat *Csl* genes

Ensembl gene ID	Gene name	Predicted amino acids	Spliced exon/introns	Status
TRIAE_CS42_6BS_TGACv1_513375_AA1639370.1	TaCslA1_6BS	581	–	Wild type
TRIAE_CS42_6BS_TGACv1_513375_AA1639370.2		390	Exon 1 and 2	Exon skipping
TRIAE_CS42_6BS_TGACv1_513376_AA1639390.2	TaCslA4_6BS	528	–	Wild type
TRIAE_CS42_6BS_TGACv1_513376_AA1639390.1		393	Exon 1 and 2	Exon skipping
TRIAE_CS42_7AS_TGACv1_569190_AA1809650.1	TaCslA3_7AS	551	–	Wild type
TRIAE_CS42_7AS_TGACv1_569190_AA1809650.2		380	Exon 7, 8 and 9	Exon skipping
TRIAE_CS42_7AS_TGACv1_569190_AA1809650.3		503	Exon 9	Exon skipping
TRIAE_CS42_7DL_TGACv1_602617_AA1962870.2	TaCslA10_7DL	515	–	Wild type
TRIAE_CS42_7DL_TGACv1_602617_AA1962870.1		555	Intron 8	Intron retention
TRIAE_CS42_3DL_TGACv1_249033_AA0835410.2	TaCslA6_3DL	524	–	Wild type
TRIAE_CS42_3DL_TGACv1_249033_AA0835410.1		572	Intron 1	Intron retention
TRIAE_CS42_3B_TGACv1_221079_AA0729630.1	TaCslA6_3B	571	–	Wild type
TRIAE_CS42_3B_TGACv1_221079_AA0729630.2		538	Exon 2	Exon skipping
TRIAE_CS42_5BL_TGACv1_404820_AA1311790.1	TaCslC10_5BL	712	–	Wild type
TRIAE_CS42_5BL_TGACv1_404820_AA1311790.2		468	Exon 5	Alternative 5′ site
TRIAE_CS42_5BL_TGACv1_404820_AA1311790.3		504	Exon 1	Exon skipping
TRIAE_CS42_5DL_TGACv1_435778_AA1454840.1	TaCslC10_5DL	708	–	Wild type
TRIAE_CS42_5DL_TGACv1_435778_AA1454840.2		502	Exon1	Exon skipping
TRIAE_CS42_5AL_TGACv1_374268_AA1195590.3	TaCslC10_5AL	703	–	Wild type
TRIAE_CS42_5AL_TGACv1_374268_AA1195590.2		496	Exon 5	Alternative 5′ site
TRIAE_CS42_5AL_TGACv1_374268_AA1195590.1		501	Exon 5	Exon skipping
TRIAE_CS42_3DL_TGACv1_251593_AA0882850.1	TaCslC1_3DL	704	–	Wild type
TRIAE_CS42_3DL_TGACv1_251593_AA0882850.2		493	Exon 5	Exon skipping
TRIAE_CS42_3DL_TGACv1_251593_AA0882850.3		679	Exon 1	Alternative 3′ site
TRIAE_CS42_3AL_TGACv1_197197_AA0665370.1	TaCslC1_3AL	704	–	Wild type
TRIAE_CS42_3AL_TGACv1_197197_AA0665370.2		560	Exon 5	Alternative 3′ site
TRIAE_CS42_3AL_TGACv1_197197_AA0665370.3		679	Exon 5	Alternative 5′ site
TRIAE_CS42_6AL_TGACv1_471004_AA1500600.1	TaCslE2_6AL	667	–	Wild type
TRIAE_CS42_6AL_TGACv1_471004_AA1500600.2		737	Intron 8	Intron retention
TRIAE_CS42_6AL_TGACv1_471004_AA1500600.3		635	Exon 4	Alternative 5′ site
TRIAE_CS42_5DL_TGACv1_433536_AA1415830.1	TaCslE1_5DL	728	–	Wild type
TRIAE_CS42_5DL_TGACv1_433536_AA1415830.2		684	Exon 4	Exon skipping
TRIAE_CS42_5BL_TGACv1_406235_AA1342600.1	TaCslE1_5BL	734	–	Wild type
TRIAE_CS42_5BL_TGACv1_406235_AA1342600.2		728	Exon 1	Exon skipping
TRIAE_CS42_2DS_TGACv1_177641_AA0581710.1	TaCslF3_2DS	847	–	Wild type
TRIAE_CS42_2DS_TGACv1_177641_AA0581710.2		735	Exon 2	Alternative 3′ site
TRIAE_CS42_2DS_TGACv1_179076_AA0604160.1	TaCslF4_2DS	783	–	Wild type
TRIAE_CS42_2DS_TGACv1_179076_AA0604160.2		700	Exon 1	Exon skipping
TRIAE_CS42_2BS_TGACv1_147667_AA0486240.1	TaCslF9_2BS	877	–	Wild type
TRIAE_CS42_2BS_TGACv1_147667_AA0486240.2		796	Exon 1	Exon skipping
TRIAE_CS42_5BL_TGACv1_409916_AA1366600.1	TaCslF7_5BL	745	–	Wild type
TRIAE_CS42_5BL_TGACv1_409916_AA1366600.2		815	Intron 2	Intron retention
TRIAE_CS42_5AL_TGACv1_374191_AA1193100.1	TaCslF7_5AL	792	–	Wild type
TRIAE_CS42_5AL_TGACv1_374191_AA1193100.2		807	Intron 1	Intron retention
TRIAE_CS42_2AL_TGACv1_094351_AA0296300.1	TaCslH1_2AL	737	–	Wild type
TRIAE_CS42_2AL_TGACv1_094351_AA0296300.2		660	Exon 9	Exon skipping
TRIAE_CS42_2AL_TGACv1_094351_AA0296300.3		480	Exon 6, 7, 8 and 9	Exon skipping
TRIAE_CS42_3AS_TGACv1_210908_AA0681280.1	TaCslJ1_3AS	738	–	Wild type
TRIAE_CS42_3AS_TGACv1_210908_AA0681280.2		766	Intron 4	Intron retention
TRIAE_CS42_3DS_TGACv1_272756_AA0924850.2	TaCslJ2_3DS	609	–	Wild type
TRIAE_CS42_3DS_TGACv1_272756_AA0924850.1		734	Intron 1	Intron retention

### Conserved motifs and domains

All predicted TaCSL proteins contain either the pfam *glycosyltransferase family 2_3* (GT) domain (PF13641) or the *cellulose_synt* domain (PF03552), considered to be the signature domains of the *GT2* superfamily [12, 26]. Subfamilies *TaCslA* and *TaCslC* contained *GT 2_3*, and *CslD, CslE, CslF, CslH,and CslJ* contained the *cellulose_synt* domain (Fig. 2). All the *TaCsl* translanted products contained the motifs D, DXD, D and QXXRW except eight truncated genes that lacked some of these motifs apparently because of the missing sequence (*TaCslA7_2DS*, *TaCslD4_1BS*, *TaCslD4_5BS*, *TaCslF2_7BL*, *TaCslF6_7AL*, *TaCslF6_7DL*, *TaCslH3_3AS*, *TaCslH2_3B*). Rice *CesA10*, *11* and *CslH3* also contained only the DXD but lacked the D and QXXRW motifs [38]. The variable amino acids in the conserved motifs DXD and QXXRW were diverse in different subfamilies of *Csl* genes, for example, for *TaCslA* (DMD, QQH/FRW); *TaCslC* (DMD, QQHRW); *TaCslD* (DCD, QVLRW); *TaCslE* (DCD, QHKRW); *TaCslF* (DC/GD, QI/VL/VRW); *TaCslH* (DCD QF/YKRW); *TaCslJ* (DCD, QNKRW). These motifs are highlighted in alignment files in the text file S_2a-f.

### Phylogenetic analysis of the *CslD* subfamily

**Fig. 3:**
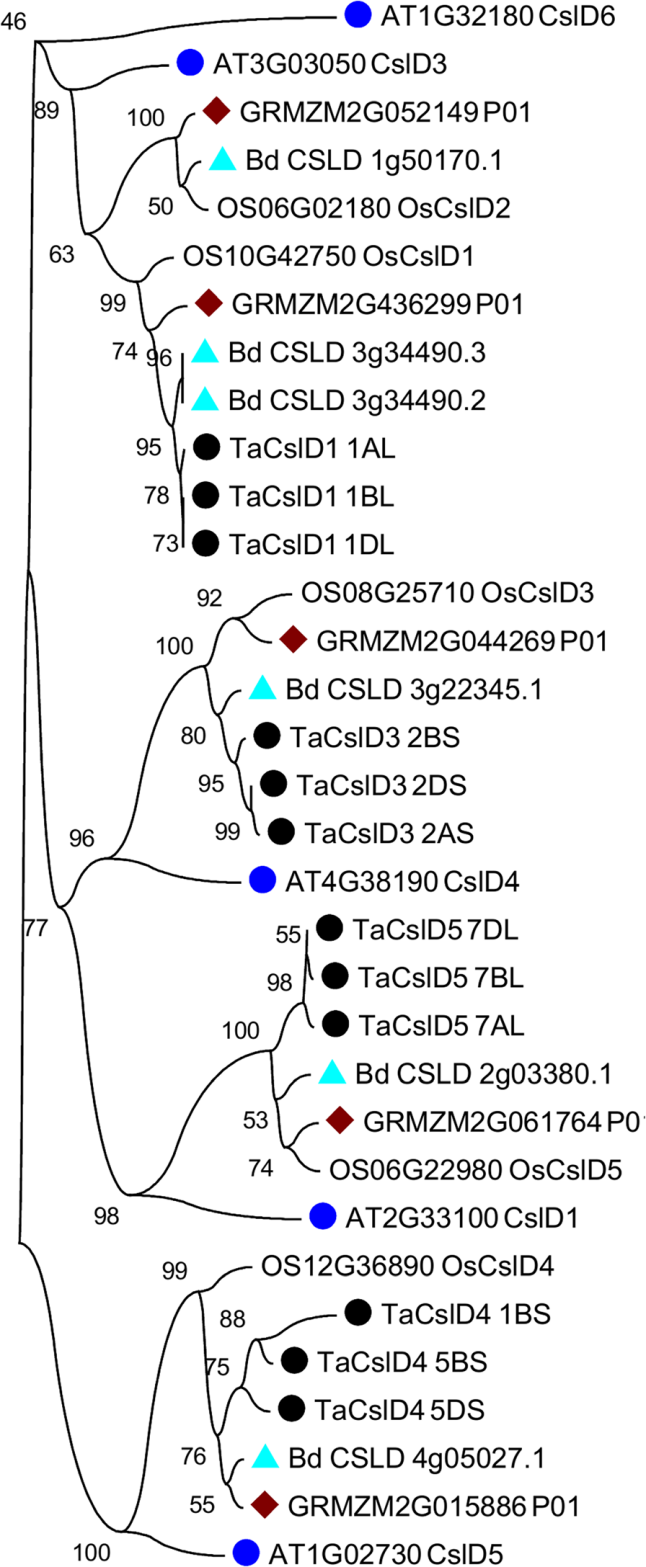
An unrooted phylogenetic tree representing the *CslD* subfamily from Arabidopsis, Brachypodium, maize, rice and wheat using Neighbour Joining (NJ) method with 1000 replicates to generate bootstrap values that are shown beside the each node forming the *Csl* clusters. Different colors and shapes represent orthologous *Csl* genes from different species. Arabidopsis-blue circles, Brachypodium- sky blue triangels, maize-brown rectangles-, rice-no marker, and wheat-black circles

The evolutionary history of the *CslD* subfamily from Arabidopsis, Brachypodium, rice, maize and wheat was inferred using the Neighbor-Joining method, in MEGA6 [36], after grouping the orthologs from various species into different clades (Fig. 3). Rice *Csl* genes were used as reference because their complete nomenclature is well documented. All the genes grouped into three clades. The first clade contained *CslD2* and *CslD1* genes from rice and their orthologs from the remaining species. The three homeologous genes of wheat branched together with *OsCslD1*; wheat genes under this clade were named *TaCslD1_1AL*, *TaCslD1_1BL*, and *TaCslD1_1DL*. The second clade contained two subgroups with the orthologs of rice genes *CslD3* and *CslD5* from different species. The genes in the first subgroup were named *TaCslD3_2AS*, *TaCslD3_2BS*, and *TaCslD3_2DS,* and those of the second subgroup *TaCslD5_7AL*, *TaCslD5_7BL*, and *TaCslD5_7DL.* The last clade was composed of the orthologs of the rice *CslD4* and wheat genes *TaCslD4_5BS*, *TaCslD4_1BS* and *TaCslD4_5DS*. Here we found only two homeologs of *TaCslD4*, but a gene from the 1BS genome (*TaCslD4_1BS*) of wheat grouped together with *TaCslD4* genes (bootstrap = 1000), pointing to a translocation from its original A genome (Table 1). This gene shared sequence identity of 85% with *TaCslD4_5BS* at the amino acid level. *OsCslD* genes shared 73–86% sequence identity with the corresponding wheat orthologs.

### Gene structure and intron evolution of *TaCslD* subfamily

**Fig. 4:**
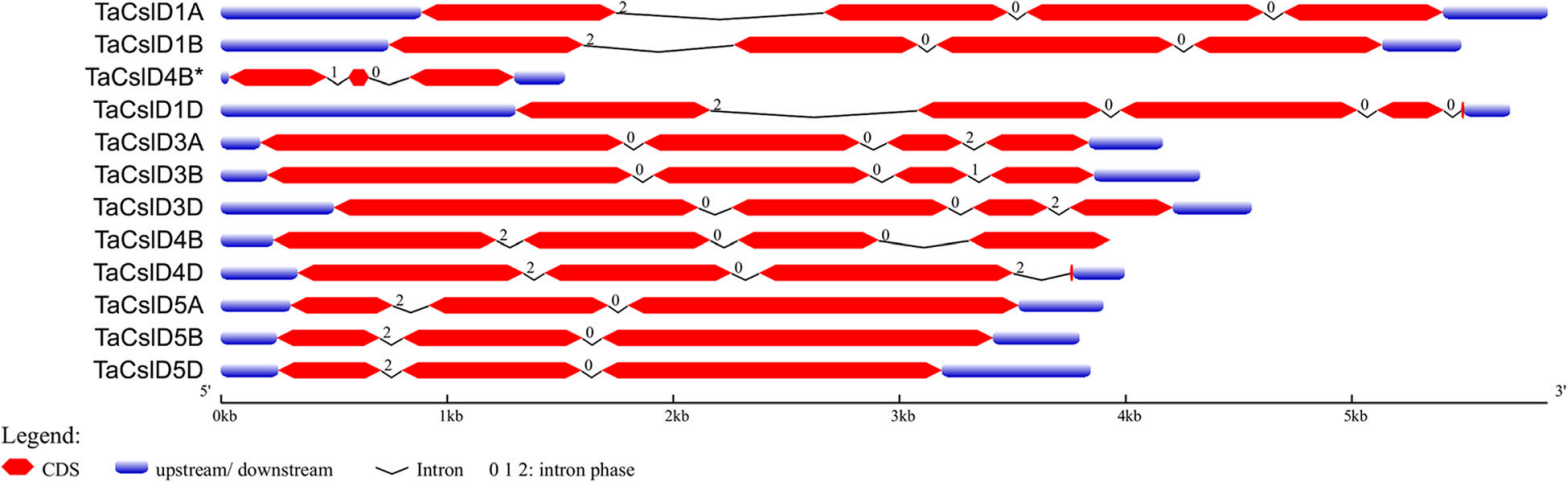
Structural features and phases of intron evolution of the *CslD* subfamily genes. Drawn to scale, exons are represented by red boxes and introns by back lines. Corresponding phases of intron evolution (0, 1, and 2) for the *CslD* genes are shown on the top of the black lines

The 12 *TaCslD* genes identified from bread wheat ranged in size from 1519 to 5864 bp. The *TaCslD4_1BS* gene was the shortest and *TaCslD1_1AL* was the longest. Homeologous copies of all the genes shared sequence identity ranging from 87 to 94% at the nucleotide level. The variation in size among different genes was primarily because of the number and length of introns but also because of a lack of the complete sequences in the database (Fig. 4). The number of introns in all the genes varied from 2 to 4. Two homeologs: *TaCslD1_1AL* and *TaCslD1_1BL* each contained three introns whereas, a third homeolog (*TaCslD1_1DL*) had four. The genes *TaCslD3*, *TaCslD4* and their homeologs contained three introns each, except *TaCslD4_1B*S with only two introns. *TaCslD5* and its homeologs also had two introns each. For the phases of introns, the genes from the *TaCslD* subfamily exhibited variable patterns of distribution. Introns 1, 2 and 3 of *TaCslD1_1AL*, *TaCslD1_1BL* and *TaCslD1_1DL* were in 2, 0, and 0 phase whereas the 4th intron of *TaCslD1_1DL* was in 0 phase. Introns 1 and 2 of *TaCslD3_2AS*, *TaCslD3_2BS* and *TaCslD3_2DS* both were in 0 phase. The third intron of these genes was in phase 2, 1 and 2 respectively. The genes *TaCslD4_5BS*, *TaCslD4_5DS*, *TaCslD5_7AL*, *TaCslD5_7BL* and *TaCslD5_7DL* had intron 1 and 2 in phases 2 and 0, respectively, and the third intron of *TaCslD4_5BS* and *TaCslD4_5DS* was in phase 0 and 2, respectively. *TaCslD4_1BS* had introns 1 and 2 in phases 1 and 0. The largest proportion of introns (60%) of all the genes was in phase 0, followed by phase 2 (34%) with a few in phase 1 (6%).

### Expression analysis of *TaCsl* genes from bread wheat

**Fig. 5:**
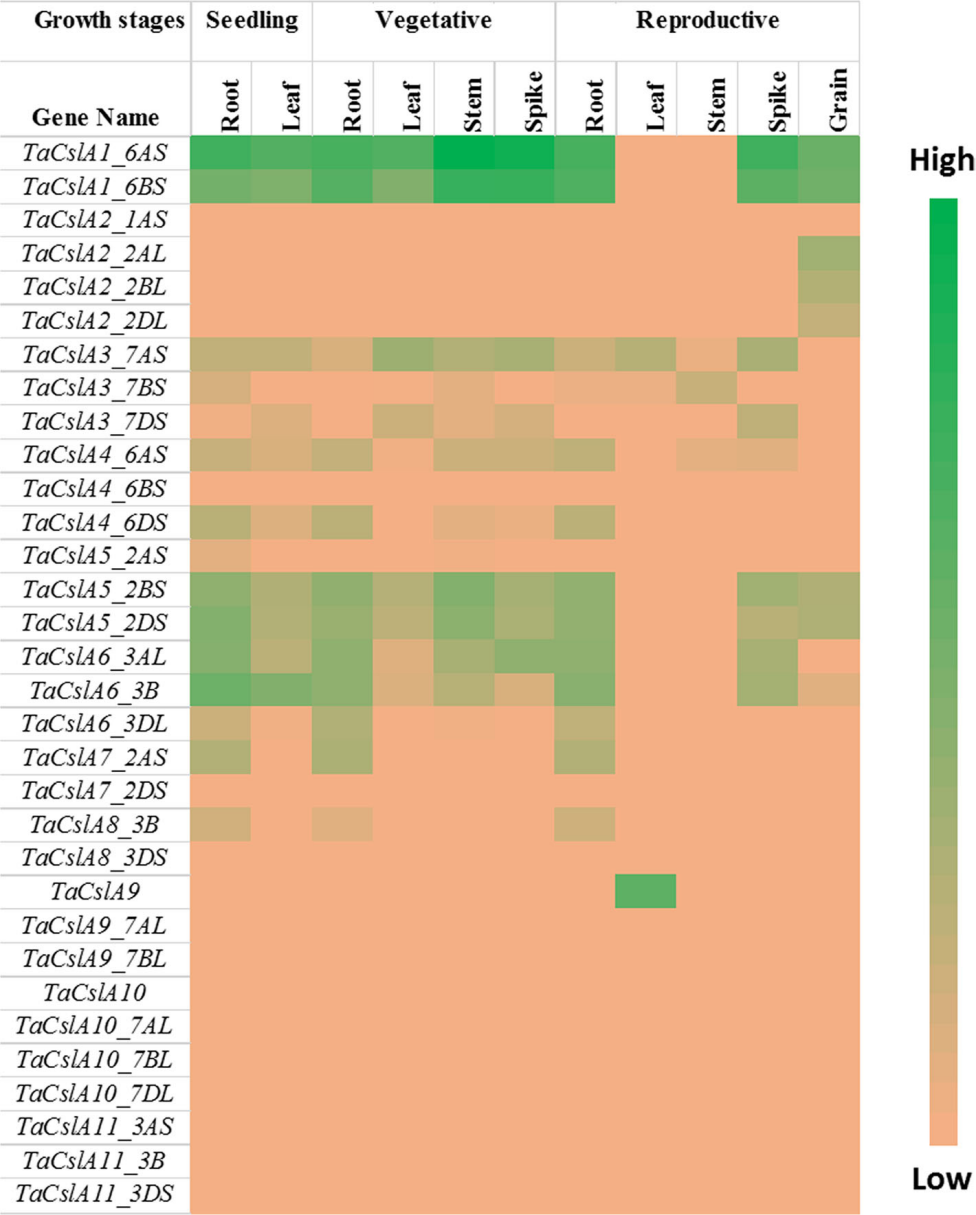
Heat map showing the expression profiling of wheat *TaCslA* genes at seedling, vegetative and reproductive stages. RNA-seq data were obtained from root, leaf, stem, spike and grain of the *Chinese spring* cultivar. The respective transcripts per 10 million values were used to construct heat map with the scale bar showing expression of the genes

**Fig. 6:**
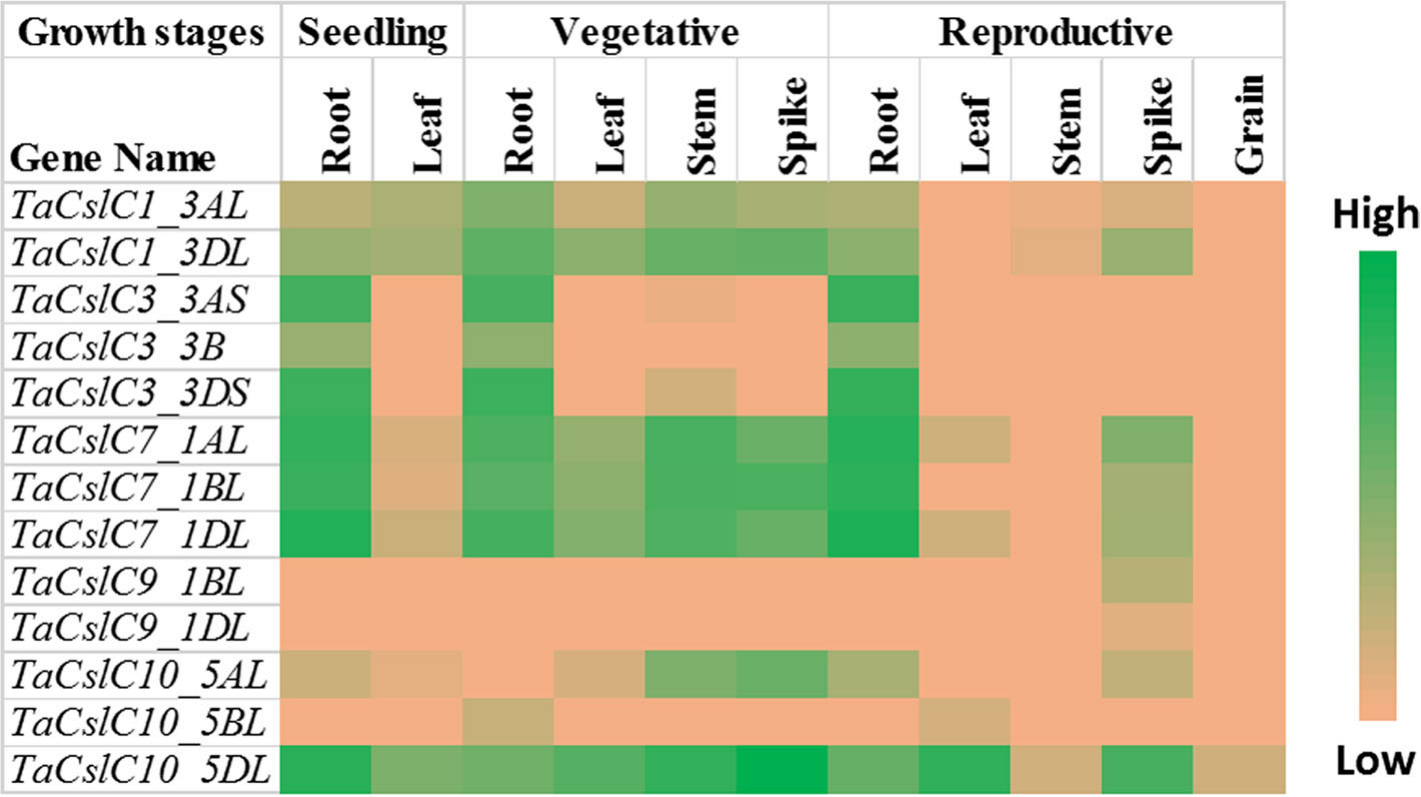
Heat map of the expression profiling of wheat *TaCslC* genes at seedling, vegetative and reproductive stages. RNA-seq data were obtained from root, leaf, stem, spike and grain of Chinese spring cultivar. The respective transcripts per 10 million values were used to construct heat map with scale bar showing expression of the genes

**Fig. 7:**
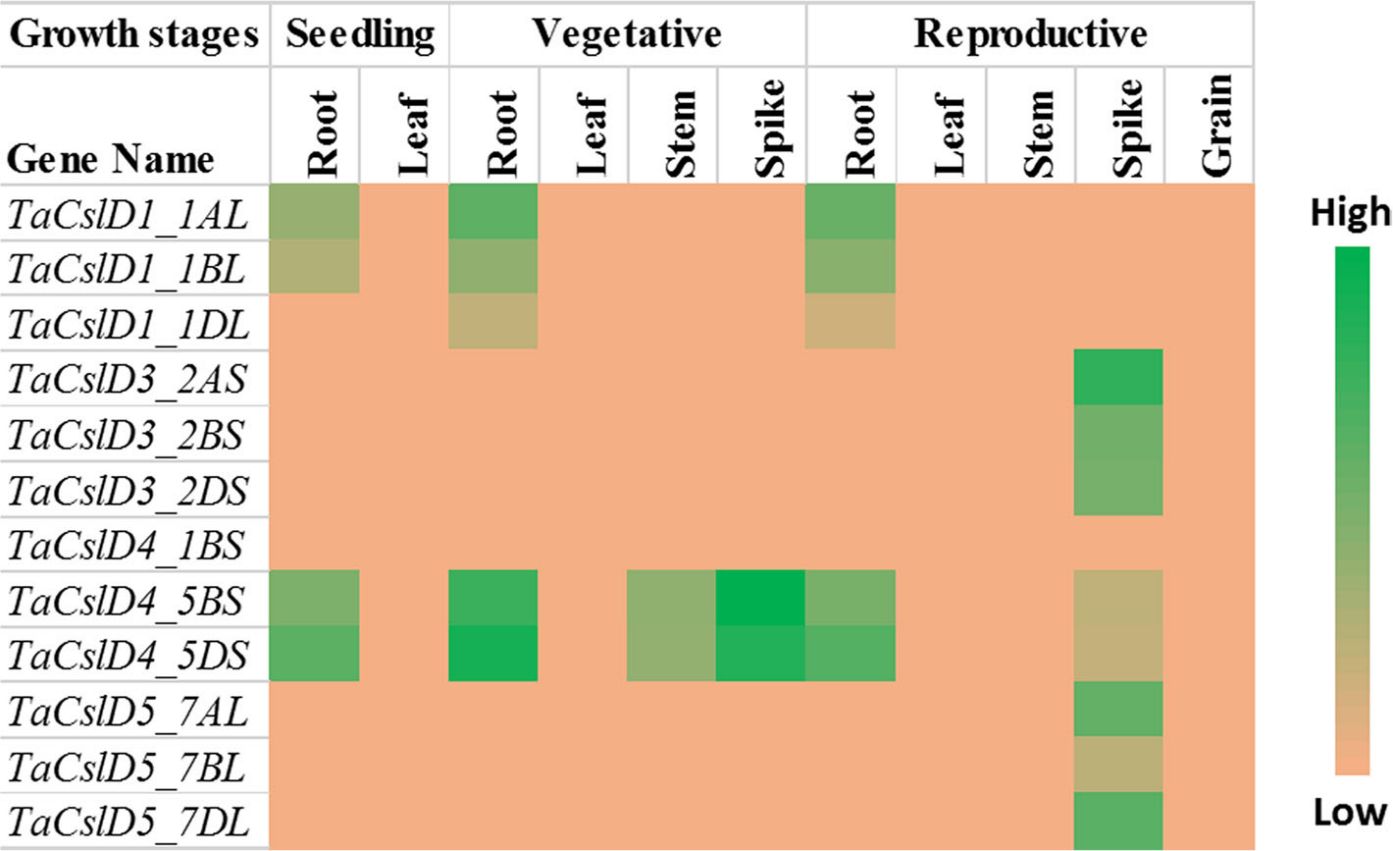
Heat map of the expression profiling of wheat *TaCslD* genes at seedling, vegetative and reproductive stages. RNA-seq data were obtained from root, leaf, stem, spike and grain of Chinese spring cultivar. The respective transcripts per 10 million values were used to construct heat map with scale bar showing expression of the genes

**Fig. 8:**
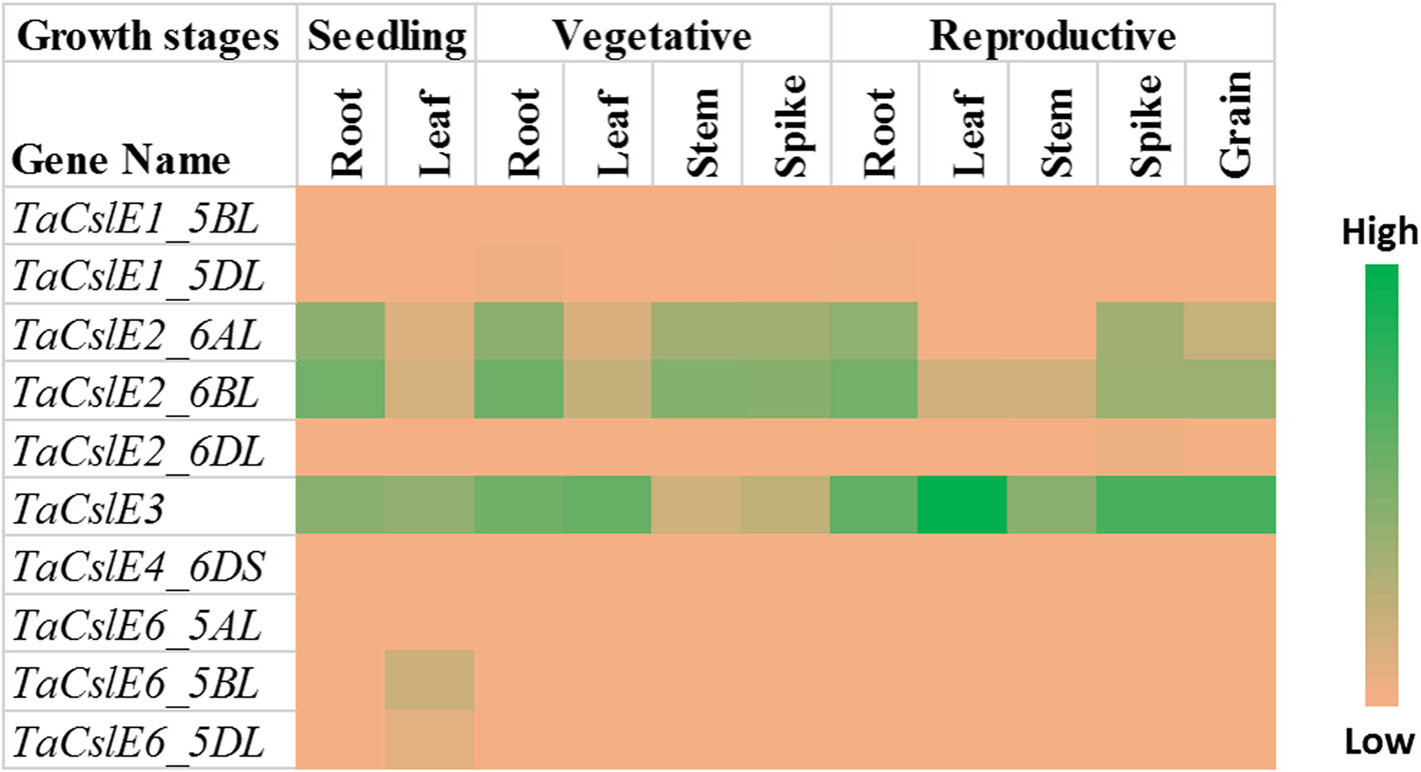
Heat map of the expression profiling of wheat *TaCslE* genes at seedling, vegetative and reproductive stages. RNA-seq data were obtained from root, leaf, stem, spike and grain of Chinese spring cultivar. The respective transcripts per 10 million values were used to construct heat map with scale bar showing expression of the genes

**Fig. 9:**
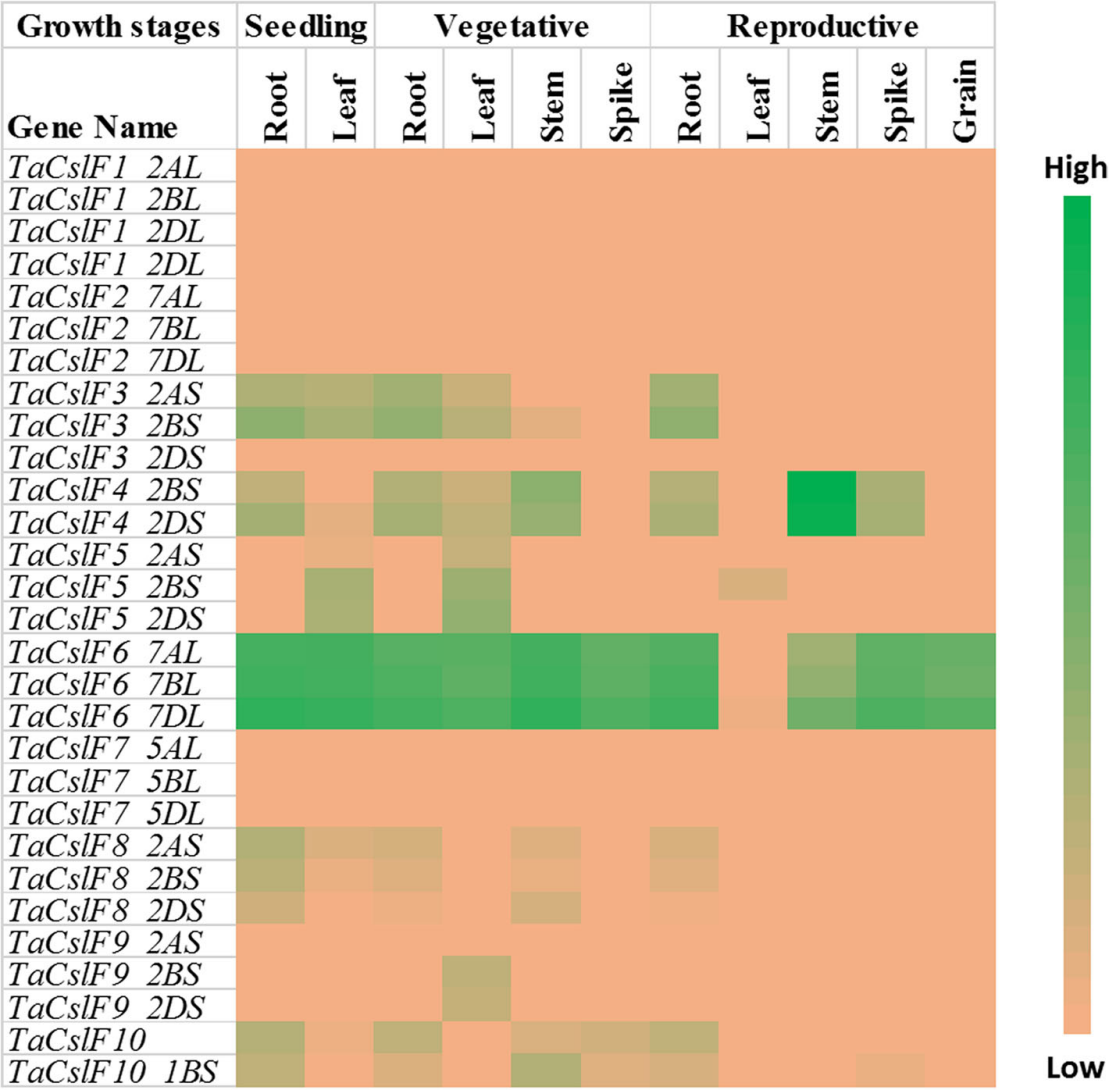
Heat map of the expression profiling of wheat *TaCslF* genes at seedling, vegetative and reproductive stages. RNA-seq data were obtained from root, leaf, stem, spike and grain of Chinese spring cultivar. The respective transcripts per 10 million values were used to construct heat map with scale bar showing expression of the genes

**Fig. 10:**
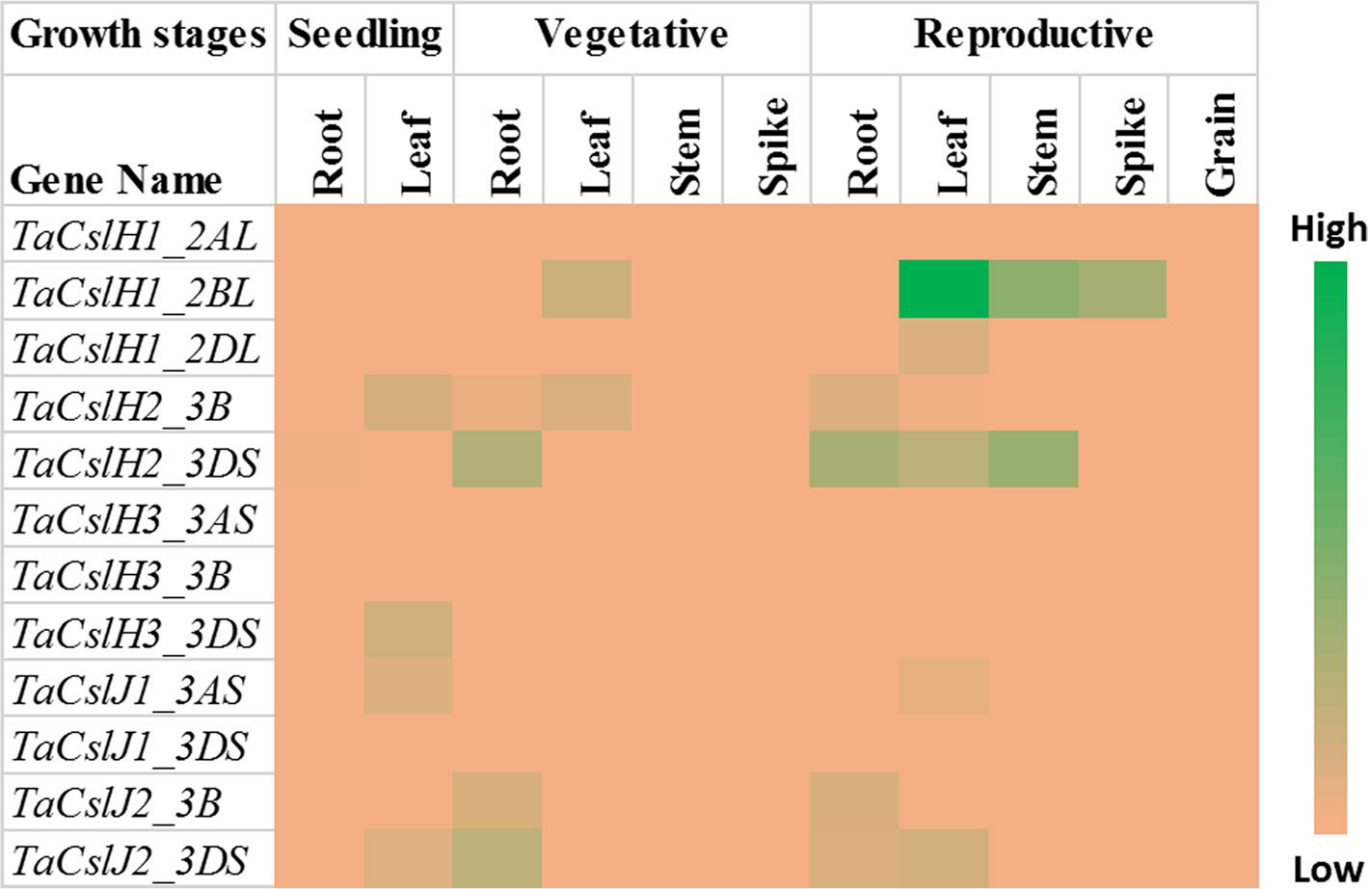
Heat map of the expression profiling of wheat *TaCslH* and *TaCslJ* genes at seedling, vegetative and reproductive stages. RNA-seq data were obtained from root, leaf, stem, spike and grain of Chinese spring cultivar. The respective transcripts per 10 million values were used to construct heat map with scale bar showing expression of the genes

Publicly available RNA-Seq datasets were used to analyse the expression of *TaCsl* genes over three developmental stages and different tissues of wheat including root, stem, leaf, spike, and grain. Expression data were available for 32 of the *TaCslA* genes. Two genes (*TaCslA1_6AS* and *TaCslA1_6BS*) were expressed in all the tissues except reproductive stem and leaves. Four genes (*TaCslA5_2BS*, *TaCslA5_2DS*, *TaCslA6_3B*, and *TaCslA6_3AL*) were expressed moderately. *TaCslA9* gene was highly expessed in the leaf tissue from the reproductive stage while the transcript abundance of the remaining genes was low (Fig. 5). *TaCslC* subfamily genes, wtht the exception of *TaCslC3, TaCslC9* and two homeologs of *TaCslC10*, were expressed highly in root and spike tissues. Two genes, *TaCslC1* and *TaCslC7* and their homeologs displayed moderate to high expression in all the tissues at seeding and vegetative stage. One gene (*TaCslC10_5DL*) exhibited moderate to high expression in all the tissues studied except reproductive stem and grain (Fig. 6). Expression of most of the genes of the *TaCslD* subfamily ranged from moderate to a high in the spike and root tissues but was very low in all the other tissues (Fig. 7). Three of the 10 *TaCslE* subfamily genes (*TaCslE2_6AL*, *TaCslE2_6BL* and *TaCslE3*) were expressed from moderate to high levels in all the tissues.The remaining genes were expressed at a very low level in all the tissues (Fig. 8). A mixed pattern of expression was observed in the large *TaCslF* subfamily. Three genes (*TaCslF6_7AL*, *TaCslF6_7BL* and *TaCslF6_7DL*) were highly expressed in all the tissues except the leaves at the reproductive stage. Two genes (*TaCslF4_2BS* and *TaCslF4_2DS*) were highly expressed in the stem tissue, but only at a low or moderate level in all other tissues. All other genes expressed at low or moderate levels in one or more tissues (Fig. 9). In the *TaCslH* subfamily, one of the eight genes, *TaCslH1_2BL*, was expressed from moderate to high levels in the leaf, stem and spike tissues. The remaining genes were expressed from low to moderate levels in all the tissues (Fig. 10). Three out of four members of the subfamily *TaCslJ* were expressed from low to moderate levels in the leaf and root tissues while one gene (*TaCslJ1_3DS*) was poorly expressed in all the tissues studied (Fig. 10).

## Discussion

Grass cell walls contain 20–40% non-cellulosic polysaccharides. The proportion and composition of these polysaccharides varies in different plant species [39]. After the first report demonstrating the β-glucan synthase activity in a *Csl*-encoded protein was published [15], several members of the *Csl* gene family have been reported to be involved in the formation of the backbone of the hemicellulosic polysaccharides [16, 18, 19, 26, 38, 40, 41]. As information on the identify of the *Csl* genes in wheat was lacking, we undertook this study to fill this gap.

We retrieved 108 *TaCsl* genes from wheat using two conserved domains, PF00535, and PF03552, which were previously shown to be present in the derived proteins of all the *Csl* genes [12]. These genes include homeologs from A, B and D genome of bread wheat. Similar patterns of homeologous genes were found for *FLOWERING LOCUS T* (*FT*), *Pairing homeologous 1* (*Ph1*) and *ADP-glucose pyrophosphorylase* (*AGPase*) gene families of hexaploid wheat. Approximately, a quarter of the identified *Csl* genes were predicted to be alternatively spliced, possibly contributing to the diversity of encoded enzymes. A recent study suggested that alternative splicing was common in plants and accounted for about 20% of the loci transcribed in the leaf and spike tissues of *Aegilops tauschii*. In the case of germinating barley embryos, 14–20% of intron-containing genes were alternatively spliced [42]. This phenomenon, apparently meant to increase the fitness of an organism, has not thus far been reported for the *Csl* genes from other species [43].

**Fig. 11:**
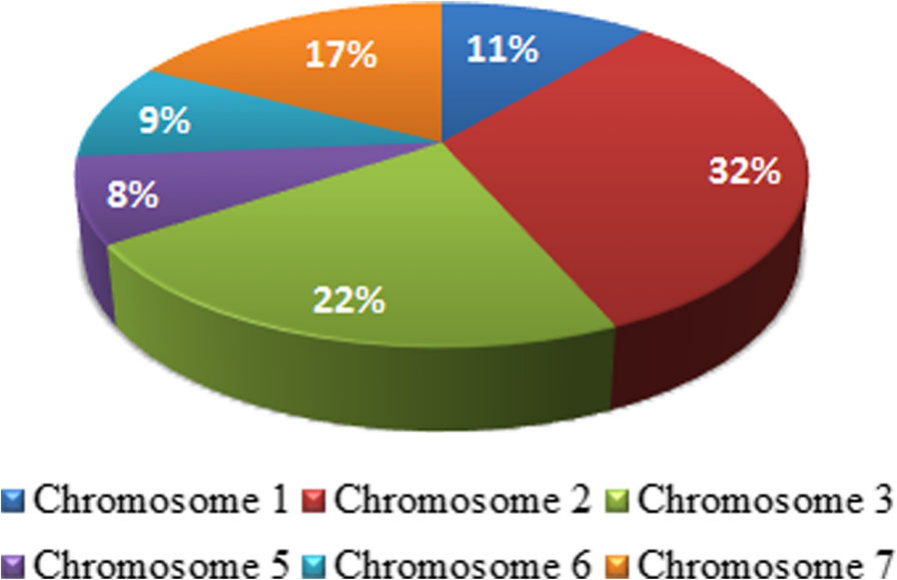
Pie chart showing the percentage of *TaCsl* genes on wheat chromosomes

The *TaCsl* genes were distributed across all the wheat chromosomes except one, chromosome 4 (Fig. 11). A similar trend of *Csl* gene distribution was observed in barley [9, 44, 45]. More than half the *TaCsl* genes were located on only two chromosomes: 2 (32%) and 3 (22%). This suggests hyper-multiplication of the *Csl* genes on these chromosomes although the reasons for this phenomenon are unknown. It appears, though, that *cis* duplication of the *Csl* genes was favored over *trans* duplication in wheat. Five of the nine *CslF* genes in barley were located on chromosome 2H [40]. In fact, the barley *CslF* gene was assigned its role in mixed-linked glucan (MLG) formation via syntenic orthology with rice long before the barely genome sequence became available [40] A detailed analysis of the rice syntenic region corresponding to a known QTL for MLG from barley, which had been published previously, initially led to the breakthrough of the role of *CslF* in the formation of this polysaccharide [40]). A similar cluster of *CslF* genes was also detected in the conserved syntenic regions of Brachypodium and sorghum on chromosomes 1and 2, respectively [9].

The observation that only half of genes from the subfamily *CslA* were expressed at varying levels in the studied tissues suggests that the apparently silent genes may provide a backup under stressful conditions. Alternatively, they may express only transiently in specialized cells or cell parts at levels too low to be detected by the method used to study expression. The first biochemical evidence for the relationship of *CslA* genes with mannan synthase activity came from the expression of a guar *CSLA* cDNA in soybean somatic embryos [15]. Subsequent studies in insect cells demonstrated the role of *CslA* family members in the glucomannan synthases [16, 46]. Reverse genetic and biochemical approaches in *Arabidopsis* and *Dendrobium officinale* have also allowed association of certain *CslA* genes with glucomannan biosynthesis [41, 47]. A recent study in wheat suggested the involvement of a gene from the *CslA* subfamily in the development of tillers, cell wall composition and stem strength. This study further suggested the probable role of *CslA* gene transcript levels in carbon partitioning throughout the plant [48].

For the subfamilies *TaCslC* and *TaCslD*, most of the genes were relatively highly expressed in the root and spike tissues during the vegetative as well as reproductive phases. Heterologous expression in *Pichia* revealed that the *CslC*-encoded enzymes made β-1,4-glucan, the backbone of xyloglucan [19]. The *CslD* subfamily is conserved in all land plants and is most closely related to the *CesA* gene family with 40–50% sequence similarity at the amino acid level [49]. Similar to *CesAs*, the *CslD* subfamily is ubiquitous in all plant genomes examined to date, unlike other, taxa-specific *Csl* subfamilies [50]. Previous reports also showed the involvement of certain members of the *CslD* subfamily in tip growth, for example development of root hairs and pollen tube elongation [51, 52], normal plant growth [50, 53], and meristem morphology [53, 54]. More recently, their role in resistance against biotic stresses has been described [55]. Adding to this discussion, our in silico expression analysis suggests the involvement of certain *TaCslD* genes in spike development. This suggestion is supported by the observation that a mutant, *slender leaf 1 (sle1)*, which encodes the CSLD4 protein in rice, reduces the number and width of spikelets in the panicle [56].

Two groups of *Csl* genes, *CslF* and *CslH,* have evolved independently in grasses [57]. A third group *CslJ*, originally believed to be specific to grasses, was recently identified in some dicots [11, 13]. Although *TaCslF6* gene showed higher expression in all the studied tissues except the leaf tissue from reproductive stage, it was the only member of the *TaCslF* subfamily which expressed highly in the grain tissue. Several studies have demonstrated the functional role of *CslF6* and *CslH* in the synthesis of MLG [18, 44, 58, 59]. Only one member of all the genes in these families, *CslF6*, was expressed in the grain, suggesting that it was responsible for MLG formation. MLG is a desirable polysaccharide as a dietary fiber but undesirable for the brewery industry because it causes haze in beer. It should be possible to select natural variants for the expression of the *CslF6* gene to select for an increased or reduced MLG content depending upon the target market for the grain.

Differential expression patterns were observed among homeologous copies from three different genomes of bread wheat, which agree with the previous studies reporting unequal contributions of the three genomes toward gene expression. Interestingly, the homeologous copies of *TaCslD* genes also differed from each other in terms of intron phase evolution, indicating structural and functional divergence of homeologous gene copies (Fig. 4). Most introns were present in phase 0, which is in accordance with previous findings showing an intron bias in favour of phase 0 [7, 60, 61]. The three homeologs of each gene were not observed for all the genes reported in this study. This could be because of the incomplete sequencing information or because of the elimination of the genes during the allopolyploidization of wheat.

## Conclusions

We have identified 108 *TaCsl* genes in bread wheat and classified them into seven subfamilies (*CslA, CslC, CslD, CslE, CslF, CslH,* and *CslJ*). Two or three homeoalleles were identified for most of the *Csl* genes. Although located on all the wheat chromosomes except chromosome 4, the *Csl* genes were especially concentrated on chromosomes 2 and 3, suggesting selective, localized duplication in *cis* phase. Only one of the 29 *CslF* genes, *CslF6*, was expressed in the grain, suggesting its role in mixed-linked glucan formation. Neither *CslJ* nor *CslH* was expressed in the grain. Information in this report will be helpful in designing experiments to alter wall composition in wheat for improving grain quality, culm strength, or culm composition for biofuels.

## Additional files

Additional file 1: Figure S1. FASTA sequences of CSL proteins used for the phylogenetic analysis. (PDF 453 kb)

Additional file 2: Figure S2. List of Csl subfamily genes, their protein sizes (number of amino acids), and multiple protein sequence alignments for different subfamilies. The conserved motifs (D, D, DXD, QXXRW) diagnostic of CSL proteins are highlighted with red boxes for each of the subfamilies. (PDF 465 kb)

## Abbreviations

CesA: Cellulose synthase
Csl: Cellulose synthase-like
GT: Glycosyltransferase
MLG: Mixed-linked glucan

## References

[1] Pauly M, Keegstra K (2008). Cell-wall carbohydrates and their modification as a resource for biofuels. Plant J.

[2] Sandhu APS, Randhawa GS, Dhugga KS (2009). Plant cell wall matrix polysaccharide biosynthesis. Mol Plant.

[3] Sorek N, Yeats TH, Szemenyei H, Youngs H, Somerville CR (2014). The implications of Lignocellulosic biomass chemical composition for the production of advanced biofuels. Bioscience.

[4] Pauly M, Gille S, Liu L, Mansoori N, de Souza A, Schultink A, Xiong G (2013). Hemicellulose biosynthesis. Planta.

[5] Richmond TA, Somerville CR (2000). The cellulose synthase superfamily. Plant Physiol.

[6] Rai KM, Thu SW, Balasubramanian VK, Cobos CJ, Disasa T, Mendu V. Identification, characterization, and expression analysis of Cell Wall related genes in Sorghum Bicolor (L.) Moench, a food, fodder, and biofuel crop. Front Plant Sci. 2016;1287. 10.3389/fpls.2016.01287 PMC5006623 27630645

[7] Kaur S, Dhugga KS, Gill K, Singh J. Novel structural and functional motifs in cellulose synthase (CesA) genes of bread wheat (Triticum Aestivum, L.). PLoS One. 2016;11(1):e0147046. 10.1371/journal.pone.0147046 PMC4714848 26771740

[8] Hazen SP, Scott-Craig JS, Walton JD (2002). Cellulose synthase-like genes of rice. Plant Physiol.

[9] Schwerdt JG, MacKenzie K, Wright F, Oehme D, Wagner JM, Harvey AJ, Shirley NJ, Burton RA, Schreiber M, Halpin C (2015). Evolutionary dynamics of the cellulose synthase gene superfamily in grasses. Plant Physiol.

[10] Burton RA, Collins HM, Kibble NA, Smith JA, Shirley NJ, Jobling SA, Henderson M, Singh RR, Pettolino F, Wilson SM (2011). Over-expression of specific HvCslF cellulose synthase-like genes in transgenic barley increases the levels of cell wall (1,3;1,4)-beta-d-glucans and alters their fine structure. Plant Biotechnol J.

[11] Farrokhi N, Burton RA, Brownfield L, Hrmova M, Wilson SM, Bacic A, Fincher GB (2006). Plant cell wall biosynthesis: genetic, biochemical and functional genomics approaches to the identification of key genes. Plant Biotechnol J.

[12] Yin Y, Johns MA, Cao H, Rupani M (2014). A survey of plant and algal genomes and transcriptomes reveals new insights into the evolution and function of the cellulose synthase superfamily. BMC Genomics.

[13] Vogel JP, Garvin DF, Mockler TC, Schmutz J, Rokhsar D, Bevan MW, Barry K, Lucas S, Harmon-Smith M, Lail K (2010). Genome sequencing and analysis of the model grass Brachypodium Distachyon. Nature.

[14] Dhugga KS (2012). Biosynthesis of non-cellulosic polysaccharides of plant cell walls. Phytochemistry.

[15] Dhugga KS, Barreiro R, Whitten B, Stecca K, Hazebroek J, Randhawa GS, Dolan M, Kinney AJ, Tomes D, Nichols S (2004). Guar seed ß-mannan synthase is a member of the cellulose synthase super gene family. Science.

[16] Liepman AH, Wilkerson CG, Keegstra K (2005). Expression of cellulose synthase-like (Csl) genes in insect cells reveals that CslA family members encode mannan synthases. Proc Natl Acad Sci U S A.

[17] Burton RA, Wilson SM, Hrmova M, Harvey AJ, Shirley NJ, Medhurst A, Stone BA, Newbigin EJ, Bacic A, Fincher GB (2006). Cellulose synthase-like CslF genes mediate the synthesis of cell wall (1, 3; 1, 4)-ß-D-glucans. Science.

[18] Doblin MS, Pettolino FA, Wilson SM, Campbell R, Burton RA, Fincher GB, Newbigin E, Bacic A (2009). A barley cellulose synthase-like CSLH gene mediates (1,3;1,4)-beta-D-glucan synthesis in transgenic Arabidopsis. Proc Natl Acad Sci U S A.

[19] Cocuron JC, Lerouxel O, Drakakaki G, Alonso AP, Liepman AH, Keegstra K, Raikhel N, Wilkerson CG (2007). A gene from the cellulose synthase-like C family encodes a beta-1,4 glucan synthase. Proc Natl Acad Sci U S A.

[20] Gupta PK, Mir RR, Mohan A, Kumar J (2008). Wheat genomics: present status and future prospects. Int J Plant Genomics.

[21] Mayer KF, Rogers J, Doležel J, Pozniak C, Eversole K, Feuillet C, Gill B, Friebe B, Lukaszewski AJ, Sourdille P (2014). A chromosome-based draft sequence of the hexaploid bread wheat (Triticum Aestivum) genome. Science.

[22] Consortium IWGS (2014). A chromosome-based draft sequence of the hexaploid bread wheat (Triticum Aestivum) genome. Science.

[23] Finn RD, Coggill P, Eberhardt RY, Eddy SR, Mistry J, Mitchell AL, Potter SC, Punta M, Qureshi M, Sangrador-Vegas A (2016). The Pfam protein families database: towards a more sustainable future. Nucleic Acids Res.

[24] Kim E, Magen A, Ast G (2007). Different levels of alternative splicing among eukaryotes. Nucleic Acids Res.

[25] Girke T, Lauricha J, Tran H, Keegstra K, Raikhel N (2004). The cell wall navigator database. A systems-based approach to organism-unrestricted mining of protein families involved in cell wall metabolism. Plant Physiol.

[26] Yin Y, Huang J, Xu Y (2009). The cellulose synthase superfamily in fully sequenced plants and algae. BMC Plant Biol.

[27] Richmond T (2000). Higher plant cellulose synthases. Genome Biol.

[28] Sievers F, Wilm A, Dineen D, Gibson TJ, Karplus K, Li W, Lopez R, McWilliam H, Remmert M, Söding J (2011). Fast, scalable generation of high-quality protein multiple sequence alignments using Clustal omega. Mol Syst Biol.

[29] Stothard P (2000). The sequence manipulation suite: JavaScript programs for analyzing and formatting protein and DNA sequences. BioTechniques.

[30] Marchler-Bauer A, Derbyshire MK, Gonzales NR, Lu S, Chitsaz F, Geer LY, Geer RC, He J, Gwadz M, Hurwitz DI. CDD: NCBI's conserved domain database. Nucleic Acids Res. 2014:gku1221. 10.1093/nar/gku1221 PMC4383992 25414356

[31] Kaur R, Singh K, Singh J (2013). A root-specific wall-associated kinase gene, HvWAK1, regulates root growth and is highly divergent in barley and other cereals. Funct Integr Genomics.

[32] Katoh K, Misawa K, Ki K, Miyata T (2002). MAFFT: a novel method for rapid multiple sequence alignment based on fast Fourier transform. Nucleic Acids Res.

[33] Felsenstein J (1996). Phylogeny inference package (version 3.2). Cladistics.

[34] Price MN, Dehal PS, Arkin AP (2010). FastTree 2–approximately maximum-likelihood trees for large alignments. PLoS One.

[35] Saitou N, Nei M (1987). The neighbor-joining method: a new method for reconstructing phylogenetic trees. Mol Biol Evol.

[36] Tamura K, Stecher G, Peterson D, Filipski A, Kumar S (2013). MEGA6: molecular evolutionary genetics analysis version 6.0. Mol Biol Evol.

[37] Choulet F, Alberti A, Theil S, Glover N, Barbe V, Daron J, Pingault L, Sourdille P, Couloux A, Paux E (2014). Structural and functional partitioning of bread wheat chromosome 3B. Science.

[38] Wang L, Guo K, Li Y, Tu Y, Hu H, Wang B, Cui X, Peng L. Expression profiling and integrative analysis of the CESA/CSL superfamily in rice. BMC Plant Biol. 2010;10 10.1186/1471-2229-10-282 PMC3022907 21167079

[39] Saxena IM, Brown R (1995). Identification of a second cellulose synthase gene (acsAII) in Acetobacter xylinum. J Bacteriol.

[40] Burton RA, Wilson SM, Hrmova M, Harvey AJ, Shirley NJ, Medhurst A, Stone BA, Newbigin EJ, Bacic A, Fincher GB (2006). Cellulose synthase-like CslF genes mediate the synthesis of cell wall (1,3;1,4)-beta-D-glucans. Science.

[41] Goubet F, Barton CJ, Mortimer JC, Yu X, Zhang Z, Miles GP, Richens J, Liepman AH, Seffen K, Dupree P (2009). Cell wall glucomannan in Arabidopsis is synthesised by CSLA glycosyltransferases, and influences the progression of embryogenesis. Plant J.

[42] Zhang Q, Zhang X, Wang S, Tan C, Zhou G, Li C (2016). Involvement of alternative splicing in barley seed germination. PLoS One.

[43] Zhou Y, Zhou C, Ye L, Dong J, Xu H, Cai L, Zhang L, Wei L (2003). Database and analyses of known alternatively spliced genes in plants. Genomics.

[44] Schreiber M, Wright F, MacKenzie K, Hedley PE, Schwerdt JG, Little A, Burton RA, Fincher GB, Marshall D, Waugh R (2014). The barley genome sequence assembly reveals three additional members of the CslF (1, 3; 1, 4)-β-glucan synthase gene family. PLoS One.

[45] Burton RA, Jobling SA, Harvey AJ, Shirley NJ, Mather DE, Bacic A, Fincher GB (2008). The genetics and transcriptional profiles of the cellulose synthase-like HvCslF gene family in barley. Plant Physiol.

[46] Suzuki S, Li L, Sun Y-H, Chiang VL (2006). The cellulose synthase gene superfamily and biochemical functions of xylem-specific cellulose synthase-like genes in Populus Trichocarpa. Plant Physiol.

[47] He C, Wu K, Zhang J, Liu X, Zeng S, Yu Z, Zhang X, da Silva JAT, Deng R, Tan J. Cytochemical localization of polysaccharides in Dendrobium Officinale and the involvement of DoCSLA6 in the synthesis of Mannan polysaccharides. Front Plant Sci. 2017;8:173. 10.3389/fpls.2017.00173 PMC5306395 28261235

[48] Hyles J, Vautrin S, Pettolino F, MacMillan C, Stachurski Z, Breen J, Berges H, Wicker T, Spielmeyer W (2017). Repeat-length variation in a wheat cellulose synthase-like gene is associated with altered tiller number and stem cell wall composition. J Exp Bot.

[49] Doblin MS, De Melis L, Newbigin E, Bacic A, Read SM (2001). Pollen tubes of Nicotiana Alata express two genes from different β-glucan synthase families. Plant Physiol.

[50] Hunter CT, Kirienko DH, Sylvester AW, Peter GF, McCarty DR, Koch KE (2012). Cellulose Synthase-like D1 is integral to normal cell division, expansion, and leaf development in maize. Plant Physiol.

[51] Kim CM, Park SH, Je BI, Park SH, Park SJ, Piao HL, Eun MY, Dolan L, Han CD (2007). OsCSLD1, a cellulose synthase-like D1 gene, is required for root hair morphogenesis in rice. Plant Physiol.

[52] Yuo T, Shiotani K, Shitsukawa N, Miyao A, Hirochika H, Ichii M, Taketa S (2011). Root hairless 2 (rth2) mutant represents a loss-of-function allele of the cellulose synthase-like gene OsCSLD1 in rice (Oryza Sativa L.). Breed Sci.

[53] Li M, Xiong G, Li R, Cui J, Tang D, Zhang B, Pauly M, Cheng Z, Zhou Y (2009). Rice cellulose synthase-like D4 is essential for normal cell-wall biosynthesis and plant growth. Plant J.

[54] Bernal AJ, Jensen JK, Harholt J, Sørensen S, Moller I, Blaukopf C, Johansen B, De Lotto R, Pauly M, Scheller HV (2007). Disruption of ATCSLD5 results in reduced growth, reduced xylan and homogalacturonan synthase activity and altered xylan occurrence in Arabidopsis. Plant J.

[55] Douchkov D, Lueck S, Hensel G, Kumlehn J, Rajaraman J, Johrde A, Doblin MS, Beahan CT, Kopischke M, Fuchs R. The barley (Hordeum Vulgare) cellulose synthase-like D2 gene (HvCslD2) mediates penetration resistance to host-adapted and nonhost isolates of the powdery mildew fungus. New Phytol. 2016;212:421–33. 10.1111/nph.14065 27352228

[56] Yoshikawa T, Eiguchi M, Hibara K-I, Ito J-I, Nagato Y (2013). Rice SLENDER LEAF 1 gene encodes cellulose synthase-like D4 and is specifically expressed in M-phase cells to regulate cell proliferation. J Exp Bot.

[57] Burton RA, Collins HM, Kibble NA, Smith JA, Shirley NJ, Jobling SA, Henderson M, Singh RR, Pettolino F, Wilson SM (2011). Over-expression of specific HVCSLF cellulose synthase-like genes in transgenic barley increases the levels of cell wall (1, 3; 1, 4)-β-D-glucans and alters their fine structure. Plant Biotechnol J.

[58] Taketa S, Yuo T, Tonooka T, Tsumuraya Y, Inagaki Y, Haruyama N, Larroque O, Jobling SA (2012). Functional characterization of barley betaglucanless mutants demonstrates a unique role for CslF6 in (1,3;1,4)-beta-D-glucan biosynthesis. J Exp Bot.

[59] Nemeth C, Freeman J, Jones HD, Sparks C, Pellny TK, Wilkinson MD, Dunwell J, Andersson AAM, Aman P, Guillon F (2010). Down-regulation of the CSLF6 gene results in decreased (1,3;1,4)-beta-D-Glucan in endosperm of wheat. Plant Physiol.

[60] Lynch M (2002). Intron evolution as a population-genetic process. Proc Natl Acad Sci U S A.

[61] Bhattachan P, Dong B (2017). Origin and evolutionary implications of introns from analysis of cellulose synthase gene. J Syst Evol.

