# Supplementary material for: Genome-wide analysis of the cellulose synthase-like (Csl) gene family in bread wheat (Triticum aestivum L.)
Source: BMC Plant Biol. 2017 Nov 3;17:193. doi: 10.1186/s12870-017-1142-z (PMC5670714; doi:10.1186/s12870-017-1142-z)
Supplement: Supplementary file 1 — FASTA sequences of CSL proteins used for the phylogenetic analysis. (PDF 453 kb) [file 12870_2017_1142_MOESM1_ESM.pdf]

Additional file - Figure S1: FASTA sequences of *CSL* proteins used for the phylogenetic analysis

>CSLA_2BS_TGACv1_146583_AA0468630.1_Triticum_aestivum
MEAAEIGGALLFALAAAAALFSAVSTGAVDFSHPLAVGGRVDFQETISWFIGIFDGSSSSSSAAGGVSLAEVYELWVRVR
GRVIAPALQVAVWACMVMSVMLVVEALYNCVVSLGVKAVGWRPEWRFKWEPLAGDDEEKGGAHYPMVLVQIPM
YNELEVYKLSIGAACELQWPKDRIIVQVLDDSTDPFIKNLVELECESWAVKGLNIKYATRSSRKGFKAGALKKGMECDYA
KQCEYVAIFDADFQPEPDFLLRTVPFFVHNPEVALVQARWSFVNDTASLLTRVQKMFFDYHFKVEQEAGSATFSFFSFN
GTAGVWRTSAIKEAGGWKDRTTVEDMDLAVRATLKGWKFVYVGDIRVKSELPSTYKAYCRQQFRWSCGGAHLFRKV
AKDILTAKDVSLIKKFHMLYSFFLVRRVVAPTVACILYNIILPISVMIPELFLPVWGIAYIPTVLLVVTAIRHPKNLHILPFWIL
FESVMTMHRMRAALSGLFELSEFNEWVVTKKTGNNFEDSEVPLLQKTRKRLRDRVNFREIVFSAFLFFCASYNLVFTGK
TSYYFNLYLQGLAFVCLGLNFTGTCSCCQ

>CSLA_2AS_TGACv1_113418_AA0355820.2_Triticum_aestivum
MEAAEIGGALLFALAAAAALFAAVSTGAIDFSRPLAVGGRVDFQEAISWFIGVFDGSSSSSAAGGVSLAEVYELWVRVR
GRVIAPALQVAVWACMVMSVMLVVEALYNCVVSLGVKAVGWRPEWRFKWEPLAGDDEEKGGAHYPVVLVQIPMY
NELEVYKLSIGAACELQWPKDRIIVQVLDDSTDPFIKNLVELECESWSVKGLNIKYATRSSRKGFKAGALKKGMEYDYAK
QCEYVAIFDADFQPEPDFLLRTVPFFVHNPEVALVQARWSFVNDNASLLTRVQKMFFDYHFKVEQEAGSATFSFFSFN
GTAGVWRAAAIKEAGGWKDRTTVEDMDLAVRATLKGWKFVYVGDIRVKSELPSTYKAYCRQQFRWSCGGAHLFRKV
AKDILTAKDVSLIKKFHMLYSFFLVRRVVAPTLACILYNIILPISVMIPELFLPIWGIAYIPTVLLVVTAIRHPKNLHILPFWILF
ESVMTMHRMRAALSGLFELSEFNEWVVTKKTGNNFEDNEVPLLQKTRKRLRDRVNFREIVFSAFLFFCASYNLVFPGKT
RYYFNLYLQGLAFVCLGLNFTGTCSCCQ

>CSLA_2DS_TGACv1_177473_AA0578070.1_Triticum_aestivum
MEAAEIGGALLFALAAAAALFAAVSTGAVDFSHPPAVGGRVDFQEAISWFIGVFDGSSSSSSAAGGVSLAEVYELWVR
VRGRVIAPALQVAVWACMVMSVMLVVEALYNCVVSLGVKAVGWRPEWRFKWEPLAGDDEEKGGAHYPMVLVQIP
MYNELEVYKLSIGAACELQWPKDRIIVQVLDDSTDPFIKNLVELECESWAVKGLNIKYATRSSRKGFKAGALKKGMECDY
AKQCEYVAIFDADFQPEPDFLLRTVPFFVHNPEVALVQARWSFVNDTASLLTRVQKMFFDYHFKVEQEAGSATFSFFSF
NGTAGVWRTAAIKEAGGWKDRTTVEDMDLAVRATLKGWKFVYVGDIRVKSELPSTYKAYCRQQFRWSCGGAHLFRK
VAKDILTAKDVSLIKKFHMLYSFFLVRRVMAPTVACILYNIILPISVMIPELFLPVWGIAYIPTVLLVVTAIRHPKNLHILPFWI
LFESVMTMHRMRAALSGLFELSEFNEWVVTKKTGNNFEDNEVPLLQKTRKRLRDRVNFREIVFSAFLFFCASYNLVFPG
KTSYYFNLYLQGLAFVCLGLNFTGTCSCCQ

>CSLA_2AS_TGACv1_113300_AA0354190.1_Triticum_aestivumMEAGEIGGALLFVLAAAAAVLAAVSTGAVD
FSHPPAVGGQLDFQETISWFTGVYNGASYSSGAGAVSLAEVHELWVRVRGRVIAPALQVTVWACMVMSVMLAVEAL
YNCVVSLGVKAIGWRPEWRFKWEPLAGDEEKGSAHYPMVLVQIPMYNELEVYKLSIGAACELKWPKDRMIVQVLDNS
TDPLIKNLVELECETWVTKGLNIKYAPRSGQKGFKAGALKKGMECDYARQCEYVAIFDADFQPEPDFLLRTIPFFVHNPK
VALVQARWSFVNGTVSLLTRIQKMFFDYHFKVEQEAGSATFAFFSFNGTAGVWRTAAIKEAGGWKDRTTVEDMDLAI
RATLKGWKFIYVGDIRVKSELPSSYKAYCRQQFRWACGGANLFRKVAIDILTSKDVSVVKKFYMLYSFLFVRRVVAPAVA
CILSNIIVPLSVMIPELYLPVWGVAYIPAVLLVVTAIRNPKNIHLLPFWILFESVMTIHRTRAALVGLFEFSEFNEWVVTKKT
GNNFEDNKVPLLQKTRKRLRDRVNFPEILFSAFLFFCASYNLVFPGKTSYYFNLYLQGLAFAFLGLNFSGTCTCFQ

>CSLA_2DS_TGACv1_177798_AA0584795.1_Triticum_aestivumMAAWNPETHGSGAIIVGADDCETTVEDEM
AAGRDANTKLFHRVANGRKLKNFIPAISVEGITITDQAAKEEAFFEAYSELLGRCGSREHTLDLDYLGIESINLEDQDLVFQ
EEEVWKVVRDMPSDRALGPNGFIGVFFQKAWAIVKRDVMAALNKLFLNNGRGFGRLNQALITLIPKNHEACQIKDFRP

ICLVHSIPKLASKLLATRLCPRMGELVHAKQSAFIKGRNIHDNFLQVRQLARKLYKRKTKSVMLKLDISRAFDSLSWPFLFE
VLRVKGFSRTWRFWIATLLTTASSRVVVNGCVGKKFMHACGLRQGDSISPLLFVIAMDVLSAMILKARETNAVSKIPGC
APIQRLSLYVDDVVMFIKPSWTDLWFVQEALRVFGEASGLKVNFSKSSAVMIRSEEEEEVLVRKAMPWKMETFPIKYLG
LQLGIKQLTRSEWQPVVDQALKMMPGWQRGPVTRPGRLPLVNQVVRARPIHHLIVAEAPKRALDRVDKGCRAFFWA
GSEEIQGGQCAVAWRGVYRPKQMGGLGVVDLHKHGIALRLSLSQTSFSEQSRSSCTIQKLLLFKLSGPSNLNGTVSLLTR
IQKMFFDYHFKVEQEAGSATFAFFSFNGTAGVWRTAAIKEAGGWKDRTTVEDMDLAIRATLKGWKFIYVGDIRVKSEL
PSSYKAYCRQQFRWACGGANLFRKVAVDILTSKDVSVIKKFYMLYSFLFVRRVVAPAVACILSNIIVPLSVMIPELYLPVW
GVAYIPTVLLVVTAIRNPKNIHLLPFWILFESVMTIHRTRAALVGLFELTEFDEWLVTKKTGNNFEDNKVPLLQKTRKRLR
DRVNFPEILFSAFLFFCASYNLVFPGKTSYYFNLYFQGLAFAFLGLNFTGTCTCFQ

>CSLA_6BS_TGACv1_513375_AA1639370.1_Triticum_aestivumMDAAVGLPDAWSQVRAPVIVPLLKLAVAVC
LLMSVLLFLERLYMAVVIVGVKLLGRRPERRYKCDPICEDDDPELGSAAFPIVLVQIPMFNEREVYQLSIGAVCGLSWPSD
RLVVQVLDDSTDPLIKEMVRMECERWAHKGINITYQIREDRKGYKAGALKAGMKHGYVRECEYMVIFDADFQPDPDF
LHRTIPYLHHNPEIALVQARWRFVNADECLMTRMQEMSLDYHFKVEQEVSSSVCAFFGFNGTAGVWRIAAVNEAGG
WKDRTTVEDMDLAIRASLKGWKFVYLGDVQVKSELPSTFKAFRFQQHRWSCGPANLFRKMLLEIVTNKKVTIWKKFH
VIYNFFLVRKIVAHIVTFTFYCIIIPTTIFVPEVHIPKWGCVYIPTIITLLNSVGTPRSFHLLFFWILFENVMSLHRTKATLIGLLE
AGRANEWVVTEKLGSAMKMKSANKASARKSFMRMWERLNVPELGVGAFLFSCGWYDVAFGKDNFFIYLFFQSMAF
FVVGVGYVGTIVPPS

>CSLA_6AS_TGACv1_485966_AA1554960.1_Triticum_aestivumMDAAVGLPDAWSQVRAPVIVPLLKLAVALC
LLMSVLLFLERLYMAVVIVGVKLLGRRPERRYKCDPICEDDDPELGSAAFPVVLVQIPMFNEREVYQLSIGAVCGLSWPS
DRLVVQVLDDSTDPLVKEMVRMECERWAHKGINITYQIREDRKGYKAGALKAGMKHGYVRECEYMVIFDADFQPDP
DFLHRTIPYLHHNPEIALVQARWRFVNADECLMTRMQEMSLDYHFKVEQEVSSSVCAFFGFNGTAGVWRIAAVNEA
GGWKDRTTVEDMDLAIRASLKGWKFVYLGDVQVKSELPSTFKAFRFQQHRWSCGPANLFRKMLVEIVTNKKVTIWKK
FHVIYNFFLVRKIVAHIVTFTFYCIIIPTTIFVPEVHIPKWGCVYIPTIITLLNSVGTPRSFHLLFFWILFENVMSLHRTKATLIGL
LEAGRANEWVVTEKLGSAMKMKSANKASARKSFMRMWERLNVPELGVGAFLFSCGWYDVAFGKDNFFIYLFFQSM
AFFVVGVGYVGTIVPQS

>CSLA_U_TGACv1_642146_AA2112270.1_Triticum_aestivumMAAALLPGTRITFSGAWQQVRGPVIVPLLRAS
VLLCVAMSAMLLAEKVYMAVVVLALRLLGRRPELQYRWEPMRDGDDPELGSAAYPMVLVQIPMYNEREVYQLSIGA
ACGLSWPSDRIIVQVLDDSTDPVVKELVQVECQRWARKGVNIKYETRNNRRGYKAGALKEAMKHGYVKDCDLVAIFD
ADFQPEPDFLSRSVPFLVHNPDIALVQARWKFVNADECLMTRMQEMSLDYHFKVEQEVGSSTYAFFGFNGTAGVWRI
SALNEAGGWKDRTTVEDMDLAVRASLKGWKFVYLGDLKVKNELPSTFKAFRYQQHRWSCGPANLFRKMVMEIIKNK
KVTLWKKIHVVYNFFFLRKVVAHIMTFVFYCLVIPATVLVPEVEVPKWGCVYIPAIITLLSVVGTPRSVHLVIFWALFENV
MSLHRTKATFIGLLEAHTVNEWVVTEKLGDTVKTKMPSKALKKLRIGIGERLHLWELGVAAYLFICGCYSISFGNNHYFIF
LLMQSIAFFIVGVGYVGTFVTQ

>CSLA_7BL_TGACv1_579090_AA1903960.1_Triticum_aestivumMSTLPRVWQIAAAWEQVRGPVIVPLLRVSV
LLCLAMSAMLFAEKVYMAVVVLAVRLLGRRPEQQYRWEPVGDGNDPELGSAAYPMVLVQIPMYNEREVYQLSIGAA
CGLSWPSDRIIVQVLDDSTDPVIKELVQVECRRWARKGVNIKYEIRDNRRGYKAGALKEGMKHGYVKDCDLVAIFDADF
QPEPDFLSRSVPFLVHNPDIALVQARWKFVNADECLMTRMQEMSLDYHFKVEQEVGSSTYAFFGFNGTAGVWRISAL
NEAGGWKDRTTVEDMDLAVRASLKGWKFVYIGDLKVKNELPSTFKAFRYQQHRWSCGPANLFRKMVMEIVRNKKVT
LWKKIHVIYNFFLVRKVVAHIVTFVFYCVVERIDMVD

>CSLA_7AL_TGACv1_558725_AA1795700.1_Triticum_aestivumMSTLPGVWQIAAAWEQVRGPVIVPLLRASV
LLCLAMSAMLFAEKVYMAVVVLAVRLLGRRPERQWKWEPVGEDDPELGSAAYPMVLVQIPMYNEREVYQLSIGAAC
GLSWPSDRIVVQVLDDSTDPVIKELVQVECRRWARKGVNIKYEIRDNRRGYKAGALKEGMKHGYVKDCDLVAIFDADF
QPEPDFLWRAVPFLVHNPDIALVQARWKFVNADECLMTRMQEMSLDYHFRVEQEVGSSTYAFFGFNGTAGVWRISA
LNEAGGWKDRTTVEDMDLAVRASLKGWKFVYLGDLKVKNELPSTFKAFRYQQHRWSCGPANLFRKMVMEIIRNKKV
TLWKKIHVVYSFFLVRKVVAHIVTFVFYCLVIPATVLVPEVEVPKWGCVYIPTIITLLNAVGTPRSVHLVVFWVLFENVMS
LHRAKATFIGLLEAGTVNEWVVTEKLGDTLKAKMPSKALKKLRMRIGERLHLWELGVAAYLFLCGCYDISFGNNRYFIFL
FMQSIAFFIVGVGYVGTFVAQ

>CSLA_6DS_TGACv1_543811_AA1744360.1_Triticum_aestivumMAPLGADAAAAAWAAVRARAVAPALTAA
VWACLAMSAMLLLEAACMSLVSLVAVRLLRLRPERRFKWEPMAGALEGGEADVEDPPASAGRREFPMVLVQIPMYN
EKEVYKLSIGAVCALTWPPDRIIIQVLDDSTDPIIKELVELECQEWASKKIDIKYEVRNNRKGYKAGALKKGMEHVYAQQC
EFVAIFDADFQPESDFLLKTIPFLVHNPKIALVQTRWKFVNYDACLMTRIQKMSLDYHFKVEQESGSFMHAFFGFNGTA
GVWRVSAINESGGWKDRTTVEDMDLAVRACLKEWEFLYVGDIRVKSELPSTFKAYRHQQHRWTCGAANLFRKMGW
EIVTNKGVSIWKKWHLLYSFLFVRRVIAPILTFLFYCVVIPLSAMVPEVHIPVWGLVYIPTAITVMNAIRNPGSLHLMPFWI
LFENVMSMHRMRAALTGLLETAHVNDWVVTEKVGDLVKDDFDVPLLEPLKPTECVERIYIPELLLALYLLICASYDYVLG
SQTYFMYIYLQALAFIVLGFGFVGMKTPCS

>CSLA_6AS_TGACv1_487286_AA1569690.1_Triticum_aestivumMAPLSAGAAAAAWAAVRARAVAPALTAAV
WACLAMSAMLLLEAACMSLVSLVAVRLLRLRPERRFKWEPMTGALEGGEADVEDPAGRREFPMVLVQIPMYNEKEV
YKLSIGAVCALTWPPDRIIIQVLDDSTDPIIKELVELECQEWASKKIDIKYEVRNNRKGYKAGALKKGMEHVYAQQCEFVA
IFDADFQPESDFLLKTIPFLVHNPKIALVQTRWKFVNYDACLMTRIQKMSLDYHFKVEQESGSFMHAFFGFNGTAGVW
RVSAINESGGWKDRTTVEDMDLAVRACLKEWEFLYVGDIRVKSELPSTFKAYRHQQHRWTCGAANLFRKMGWEIVT
NKGVSIWKKWHLLYSFLFVRRVIAPILTFLFYCVVIPLSAMVPEVHIPVWGLVYIPTAITIMNAIRNPGSLHLMPFWILFEN
VMSMHRMRAALTGLLETAHVNDWVVTEKVGDVVKDDFEVPLLEPLKPTECVERIYIPELLLALYLLICASYDYVLGSQTY
FMYIYLQALAFIVLGFGFVGMKTPCS

>CSLA_6BS_TGACv1_513376_AA1639390.2_Triticum_aestivumMAPLGADAAAAAWAAVRARAVSPALTAAV
WACLAMSAMLLLEAACMSLVSLVAVRLLRLRPQRRFKWEPMPGALPGAEADAEDPPGRREFPMVLVQIPMYNEKEV
YKLSIGAVCALTWPPDRIIIQVLDDSTDPIIKELVELECQEWASKKIDIKYEVRNNRKGYKAGALKKGMEHVYAQQCEFVA
IFDADFQPESDFLLKTIPFLVHNPKIALVQTRWKFVNYDACLMTRIQKMSLDYHFKVEQESGSFMHAFFGFNGTAGVW
RVSAINESGGWKDRTTVEDMDLAVRACLKEWEFLYVGDIRVKSELPSTFKAYRHQQHRWTCGAANLFRKMGWEIVT
NKGVSIWKKWHLLYSFLFVRRVIAPILTFLFYCVVIPLSAMVPEVHIPVWGLVYIPTAITIMNAIRNPGSLHLMPFWILFEN
VMSMHRMRAALTGLLETAHVNDWVVTEKVGDLVKDDFDVPLLEPLKPTECVERIYIPELLLALYLLICASYDYVLGSQTY
FMYIYLQALAFIVLGFGFVGTKTPCS

>CSLA_U_TGACv1_642146_AA2112290.1_Triticum_aestivumMSTLPGAWHVAAAWEQVRGPVIVPLLRASVL
LCLAMSAMLFAEKVYMAVVVLAVRLLGRRPERQYQWEPMGDDDPELGSAAYPMVLVQIPMYNEREVYQLSIGAACG
LSWPSDRIVVQVLDDSTDPVIKELVRVECRRWARKGVNIKYEIRDNRRGYKAGALKEGMKHGYVKDCDLVAIFDADFL
WRAVPFLVHNPDIALVQARWKFVNADECLMTRMQEMSLDYHFKVEQEVGSSTYAFFGFNGTAGVWRISALNEAGG
WKDRTTVEDMDLAVRASLKGWKFVYLGDLKVKNELPSTFKAFRYQQHRWSCGPANLFRKMVMEIIRNKKVTLWKKI
HVVYSFFLVRKVVAHIVTFVFYCLVIPATVLVPEVEVPKWGCVYIPTIITLLNAVGTPRSVHLVVFWVLFENVMSLHRAKA

TFIGLLEVGTVNEWVVTEKLGDTLKAKMPSKALRKLRMRIGERLHLWELGVAAYLFLCGCYDISFGNNRYFIFLFMQSIA
FFIVGVGYVGTFVAQ

>CSLA_7BS_TGACv1_592860_AA1945380.1_Triticum_aestivumMAGDGEGAAAFAVAKAEWLAGSGGLPLLR
WWRASGGGELLRGWDAVRAGAVAPALAAVSGACLAMSAMLLAEAVFMAAASLVRRRPERRYSAGPLGAQDGEDE
DEERGLLGYPMVLVQIPMYNEREVYKLSIGAACGLSWPSDRVIVQVLDDSTDPTIKDLVELECKIWAKKGKNVKYEVRN
NREGYKAGALKEGMLHAYVQQCDFLAVFDADFQPEPDFLMRTIPYLSRNPQIALVQARWEFVNPNECLMTRIQKMTL
DYHFKVEQEAGSSTFAFFGFNGTAGVWRISAIKEAGGWDDRTTVEDMDLAVRAGLKGWKFVYVGDVKVKSELPSNLK
AYRRQQHRWTCGAANLFRKMGAEILLTKEVSLWWKLYLLYSFFLVRKVVAHVVPFVLYCVVIPFSVLIPEIKIPAWGVVYI
PTAITILYAVRNPSSIHFIPFWILFENVMSFHRTKATFIGLLELGSVNEWVVTEKLGSVSNTKPVPQILEKPRCRFWDRWT
VSELLFAVFLFVCATYNLVYGSDFYFIYIYLQAITFIIVGTGFCGTSNS

>CSLA_7DS_TGACv1_623146_AA2050070.1_Triticum_aestivumMAGDGEGAAAFAAAKAEWLDGSGGLPLLR
WWRASGGGELLGRWDAVRAGAVAPALAAVSGACLAMSAMLLAEAVFMAAASLVRRRPERRYSAGPLGAQDGEDE
ERGLLGYPMVLVQIPMYNEREVYKLSIGAACGLSWPSDRVIVQVLDDSTDPTIKDLVELECKIWAKKGKNVKYEVRNNR
EGYKAGALKEGMLHAYVQQCDFLAVFDADFQPEPDFLMRTIPYLARNPQIALVQARWEFVNPNECLMTRIQKMTLDY
HFKVEQEAGSSTFAFFGFNGTAGVWRISAIKEAGGWDDRTTVEDMDLAVRAGLKGWKFVYVGDVKVKSELPSNLKAY
RRQQHRWTCGAANLFRKMGAEILLTKEVSLWWKLYLLYSFFLVRKVVAHVVPFVLYCVVIPFSVLIPEIKIPAWGVVYIPT
AITVLYAVRNPSSIHFIPFWILFENVMSFHRTKATFIGLLELGSVNEWVVTEKLGSASNTKPVPQILERPRCRFWDRWTVS
ELLFAVFLFVCATYNLVYGSDFYFIYIYLQAITFIIVGTGFCGTSNS

>CSLA_7AS_TGACv1_569190_AA1809650.1_Triticum_aestivumMAGDGEGAGDGEGAAAFAAAKAEWLGGS
GGLPLLRWWRASGGGELLRGWDAVRAGAVAPALAAVSGACLAMSAMLLAEAVFMAAASLVRRRPERRYSAGPLGA
QDGEDEERGLLGYPMVLVQIPMYNEREVYKLSIGAACGLSWPSDRVIVQVLDDSTDPTIKDLVELECKIWAKKGKNVKY
EVRNNREGYKAGALKEGMLHAYVQQCDFLAVFDADFQPEPDFLMRTIPYLARNPQIALVQARWEFVNPNECLMTRIQ
KMTLDYHFKVEQEAGSSTFAFFGFNGTAGVWRISAIKEAGGWDDRTTVEDMDLAVRAGLKGWKFVYVGDVKVKSEL
PSNLKAYRRQQHRWTCGAANLFRKMGAEILLTKEVSLWWKLYLLYSFFLVRKVVAHVLPFVLYCVVIPFSVLIPEIKIPAW
GVVYIPTAITILYAVRNPSSIHFIPFWILFENVMSFHRTKATFIGLLELGSVNEWVVTEKLGSVSNTKPVPQILERPRCRFW
DRWTVSELLFAVFLFVCATYNLVYGSDFYFIYIYLQAITFIIVGTGFCGTSNS

>CSLA_7DL_TGACv1_602617_AA1962870.1_Triticum_aestivumMEAAEQIAVVWKQVRGPVIVPLLRASVMV
CLAMCVILFVEKVYMAVVIVAMRLIGRRPERQWRWEPLRDDDPELGNAAYPMVLVQIPMYNEREVYKKSIGAACGLS
WPSDRIVIQVLDDSTDPAIKELVQVECQRWANKGVNIKYEIRDNRRGYKAGALKEGMKHGYVKDCDFVVIFDADFQPE
PDYLSRAMPFLIHNPEIALIQARWVFVNANECLMTRMQEMSLDYHFKVEQEVGSSAYAFFGFNGTAGVWRISALNEA
GGWKDRTTVEDMDLAVRASLKGWKFVYLGDLRVKSELPSTFKAFRYQQHRWSCGPANLFRKMLMEIVKNQKVTLW
KKIYVIYNFFFVRKIIGHILTSVFYCLVIPATVFVPEIEIPRWGYFYIPTIITLLNAVGTPRSFHLVIFWVLFENVMSLHRTKATF
SGLLELGRVNEWVVTEKLGDVLKMKVQSKVTKKLRMRIRERYIIIIGLLMCISQLSYLNFNMESGCSFWSLVLQPISSFVE
VTTFCLAKDITISFSSCNPSLSLSSVWDLLGHLSPTDNALRSWCNVVRKDSV

>CSLA_7AL_TGACv1_557254_AA1778850.1_Triticum_aestivumMEAAEQIAVVWKQVRGPVIAPLLRASVMV
CLAMCVILFVEKVYMAVVIVAMRLIGRHPERQWRWEPLRDDDPELGNAAYPMVLVQIPMYNEREVYKKSIGAACGLS
WPSDRIVIQVLDDSTDPAIKELVQAECHRWANKGVNIKYEIRDNRRGYKAGALKEGMKHGYVKDCDYVVIFDADFQPE
PDYLSRAMPFLVHNPEIALVQARWVFVNANECLMTRMQEMSLDYHFKVEQEVGSSAYAFFGFNGTAGVWRISALNE

AGGWKDRTTVEDMDLAVRASLKGWKFVCLGDLRVKSELPSTFKAFRYQQHRWSCGPANLFRKMLMEIVKNQKVTL
WKKIYVIYNFFLVRKIIGHILTSVFYCLVIPATVFVPEVEIPRWGYFYIPTVITLLNAVGTPRSFHLVIFWVLFENVMSLHRTK
ATFSGLLELGRVNEWVVTEKLGDILKMKVQSKVTKKLRMRIRERLQLLELGVAAYIFFCGSYDLLFGKRYYYVFLFMQSIA
FFVVGVGFVGTLVPN

>CSLA_7BL_TGACv1_578444_AA1895100.1_Triticum_aestivumMEAAEQIAVVWKQVRGPVIVPLLRASVMVC
LAMCVILFVEKVYMAVVIVAMRLIGRRPERQWRWEPLRDDDPELGNAAYPMVLVQIPMYNEREVYKKSIGAVCGLS
WPSDRIVIQVLDDSTEPAIKELVQVECQRWANKGVNIKYEIRDNRRGYKAGALKEGMKHGYVKDCDFVVIFDADFQPE
PDYLSRAMPFLIHNPEIALVQARWVFVNANECLMTRMQEMSLDYHFKVEQEVGSSAYAFFGFNGTAGVWRISALNEA
GGWKDRTTVEDMDLAVRASLKGWKFVYLGDLRVKSELPSTFKAFRYQQHRWSCGPANLFRKMLMEIVKNQKVTLW
KKIYVIYNFFFVRKIIGHILTSVFYCLVIPATVFVPEVEIPRWGYFYIPTVITLLNAVGTPRSFHLVIFWVLFENVMSLHRTKAT
FSGLLELGRVNEWVVTEKLGDVLKMKVQSKVTKKLRMRIRERLQLLELGVAAYIFFCGSYDLLFGKRYYYIFLFMQSIAFF
VVGVGFVGTLVPN

>CSLA_3DL_TGACv1_249033_AA0835410.1_Triticum_aestivumMAGAGEEFMAGVWAEVPVRVDWAAVAA
QCAWAGAQARAFLVVPAIRLLVVLSLAMTVMILLEKIFVAAVCFAAKAFGHRPERRYQWRPIAAGAAAAARGDEEAGL
VGGGGGSAAFPVVLVQIPMYNEREVYKLSIGAACALEWPSDRVVIQVLDDSTDPAVKDLVEIECQRWKGKGVNIKYEV
RGNRKGYKAGALKEGLKHDYVQECEFIAMFDADFQPESDFLLRTVPFLVHNPDIALVQTRWKFVNSDECLLTRFQEMS
LDYHFKFEQEAGSIVYSFFGFNGTAGVWRISAIDDAGGWKDRTTVEDMDLAVRTALKGWKFVYVGAVKVRSELPSTFK
AYRFQQHRWSCGPANLFKKMLVEILESKKVSFWSKLHLLYDFFFVGKIAAHTVTFIYYCFAIPLSVFFPEIQIPLWGVVYVP
TVITLCKALGSPSSFHLVILWVLFENVMSLHRIRAAITGLLDAGRVNEWVVTEKLGDANKTKPATEVLDAVKVIDVELTTP
LVPKLKKRRIRLWDKYNCSEIFVGTCIVICGFYDLFYANKGYYIYLFIQGLAFLVVGFEYIGTRPPTPSA

>CSLA_3B_TGACv1_221079_AA0729630.1_Triticum_aestivumMAGAGEEFMAAVWAGLPVRVDWAAVAAQ
CAWAGMQARAFLVVPAIRLLVLLSLAMTVMILLEKVFVAAVCFAAKAFGHRPERRYQWRPIAAGAAAAARGDEEAGV
GGGGSAAFPVVLVQIPMYNEREVYKLSIGAACALEWPAERVVIQVLDDSTDPVVKDLVEIECQRWKGKGVNIKYEVRG
NRKGYKAGALKEGLKHDYVQECEFIAMFDADFQPESDFLLRTVPFLVHNPDIALVQTRWKFVNSDECLLTRFQEMSLDY
HFKFEQEAGSIVYSFFGFNGTAGVWRIAAIDDAGGWKDRTTVEDMDLAVRTALKGWKFVYVGAVKVRSELPSTFKAY
RFQQHRWSCGPANLFKKMLVEILESKKVSFWSKLHLLYDFFFVGKIAAHTVTFIYYCFAIPLSVFFPEIQIPLWGVVYVPTV
ITLCKALGSPSSFHLVILWVLFENVMSLHRIRAAVTGLLDAGRVNEWVVTEKLGDANKTKPAMEVLDAVKVIDVELTTPL
VPKLKKRRIRLWDKYNCSEIFVGTCIIISGFYDLFYANKGYYIYLFIQGLAFLVVGFEYIGTRPPTPSAG

>CSLA_3AL_TGACv1_197519_AA0666560.1_Triticum_aestivumMAGAGEEFMASAAGVWAELPVRVDWAAV
AAQCAWAGAQARAFLVVPAIRLLLVLSLTMTVMILLEKIFVAAVCFAAKAFGHKPERRYQWRPIAASACKTGGVDEEAS
VGGGSSAFPVVLVQIPMYNEREVYKLSIGAACALEWPSDRVVIQVLDDSTDPAVKDLVEIECQRWKGKGVNIKYEVRG
NRKGYKAGALKEGLKHDYVQECEFIAMFDADFQPESDFLLRTVPFLVHNPDIALVQTRWKFVNSDECLLTRFQEMSLDY
HFKFEQEAGSIVYSFFGFNGTAGVWRISAIDDAGGWKDRTTVEDMDLAVRTALKGWKFVYVGAVKVRSELPSTFKAYR
FQQHRWSCGPANLFKKMLVEILENKKVSFWSKLHLLYDFFFVGKIAAHTVTFIYYCFAIPLSVFFPEIQIPLWGVVYVPTVI
TLCKALGSPSSFHLVILWVLFENVMSLHRIKAAVTGLLDAGRVNEWVVTEKLGDANKTKPAMEALDAVKVIDVELATPL
VPKLKKRRIKLWDKYNCSEIFVGTSIIICGFYDLFYANKGYYIYLFIQGLAFLVVGFEYIGTRPPTPSAE

>CSLA_3B_TGACv1_220828_AA0720500.1_Triticum_aestivumMAMAATAGLWAEVPVRLDWATVAAQCSLA
GEQARAFLVVPALRLLVLLSLAMTVMILLEKLFVAAVCYSAKAFRHRPESRYRWRPITASACKTGGDDEEDGIVVVGSGS

GRAAFPVVLVQIPMYNEREVYKVSIGAACALEWPSDRMVIQVLDDSTDPVVKELVKTECQRWKGKGVNIRYEVRGNR
KGYKAGALKQGLMRDYVRECEFIAMFDADFQPESDFLLRTVPFLVHNPDIALVQTRWKFVNSDKCLLTRFQEMSLDYH
FKFEQEAGSIVYSFFGFNGTAGVWRISAINDAGGWEDRTTVEDMDLAVRTSLLGWKFVYVGAVKVKSELPSTFKAYRF
QQHRWSCGPANLFKKILLDILKNKKVSFWSKLHLLYDFFFVGKIAAHTVTFIYYCFAIPVSVFFPEIQIPLWGVVYVPTVITL
CKALGSPSSFHLVILWVLFDNVMSLHRIKATITGLLDTRRVNEWVVTEKLGDANKTEPAMERLDDVQVIDVELSTPLVPK
LEKRRTRLWHKYNCSEIFVGTFIIICGCYDVLYAKKGYYIYLFIQGLAFLVIGFEYIGTRPPSTE

>CSLA_3DS_TGACv1_273022_AA0927600.1_Triticum_aestivumMAATVGLREEVPVRLDWATVAAQCAWAG
EQTRSFLVVPAVRLLVLLSLAMTVMILLEKLFVAAVCYAAKAFGHRPESRYKWGPIAASACKTGGDDEEDGIVVVGSGS
GSGAFPVVLVQIPMYNEREVYKVSIGAACALEWPSDRMVIQVLDDSTDPVVKELVKTECQRWKGKGVNIRYEVRGNR
KGYKAGALKQGLMRDYVRECKFIAMFDADFQPESDFLLRTVPFLVHNPDIALVQTRWKFVNSDKCLLTRFQEMSLDYH
FKFEQEAGSIVYSFFGFNGTAGVWRISAINDAGGWEDRTTVEDMDLAVRTALLGWKFVYVGAVKVGSELPSTFKAYRF
QQHRWSCGPANLFKKMLLDILRKKKVSFWSKLHLLYDFFFVGKIAAHMVTFIYYCFAIPVSVFFPEIQIPLWGVVYVPTVI
TLCKALGSPSSFHLVILWVLFDNVMSLHRIKATITGLLDTRRVNEWVVTEKLGDANKTEPAMEGLDDVQVIDVELSTPLV
PKLEKRRTRLWDKYNCSEIFVGTCIIICGCYDVLYAKKGYYIYLFIQGLAFLVIGFEYIGTRPPSIE

>CSLA_2AL_TGACv1_093375_AA0278800.1_Triticum_aestivumMKGVSMLTMARAAWAAVRHAVVVPLLQL
AVYLCAAMSLMLFAERLYMGLVVAALWLRRRRRQRRNPGRNKGGDDDVGDLESGAAEDLPVVLVQIPMFNEKQVY
RLSIGAACGLWWPADKLVIQVLDDSTDAGIRAMVEAECRRWAGKGVHIRYENRSNRSGYKAGAMREGLKKGYAKDC
ELVAVFDADFQPDADFLRRTVPVLQADPAVALVQARWRFVNADECILTRIQEMSLDYHFSVEQEVGSACHGFFGFNG
TAGVWRVQALADAGGWKDRTTVEDMDLAVRASMRGWRFVYAGDVQVRNELPSSFKAYRYQQHRWSCGPANLM
RKMFWEIVASRQVSAWKKVHVLYGFFFVRKVVAHLVTFLFYCVVIPAYVLVGGQDVRLPKYVAMYVPAIITLLNAVCTP
RSWHLLVFWILFENVMSMHRSKATIIGLVEASRANEWVVTEKLGSVTSTPAATTTMATNKGAMKKKKSQSSILAPEIV
MGLCLLYCAVYDIFFGHDHFYVYLLMQSAAAFVIGFGYVGSQ

>CSLA_2BL_TGACv1_129747_AA0394630.1_Triticum_aestivumMRGVSMLTMARAAWAAVRYAVVVPLLQL
AVYLCAAMSLMLFAERLYMGLVVAALWLRRRRRQRRNPSRNKGDDDGGAGDLESGGGEDLPMVLVQIPMFNEKQV
YRLSIGAACGLWWPADKLVIQVLDDSTDAGIRALVEAECRRWAGKGVQIRYENRSNRSGYKAGAMREGLKKGYARDC
ELVAVFDADFQPDADFLRRTVPVLQADPAVALVQARWRFVNADECILTRIQEMSLDYHFSVEQEVGSACHGFFGFNG
TAGVWRVQALADAGGWKDRTTVEDMDLAVRASMRGWRFVYAGDVQVRNELPSSFKAYRYQQHRWSCGPANLM
RKMFWEIVASRQVSAWKKVHVLYGFFFVRKVVAHLVTFLFYCVVIPAYVLVGGQDVRLPKYVAMYVPAIITLLNAVCTP
RSWHLLVFWILFENVMSMHRSKATIIGLVEASRANEWVVTEKLGSVTSTPAAATTMAANKGAMKKKKSQSSILAPEIV
MGLCLLYCAVYDIVFGHDHFYVYLLMQSAAAFVIGFGYVGSQ

>CSLA_2DL_TGACv1_160461_AA0550770.1_Triticum_aestivumMEKKKRRSSISFLLSFGGGRRRMKGVSMLT
MARAAWAVVRYAVVVPLLQLAVYLCAAMSLMLFAERLYMGLVVAALWLRRRRRQRRSPSRNKGGDDDDLESGAAE
DLPLVLVQIPMFNEKQVYRLSIGAACGLWWPADKLVIQVLDDSTDAGIRAMVEAECRRWAGKGVQIRYENRSNRSGY
KAGAMREGLKKGYAKDCELVAVFDADFQPDADFLRRTVPVLQADPSVALVQARWRFVNADECILTRIQEMSLDYHFS
VEQEVGSACHGFFGFNGTAGVWRVQALADAGGWKDRTTVEDMDLAVRASMRGWRFVYAGDVQVRNELPSSFKAY
RYQQHRWSCGPANLMRKMFWEIVASRQVSAWKKVHVLYGFFFVRKVVAHLVTFLFYCVVIPAYVLVGGQDVRLPKY
VAMYVPAIITLLNAVCTPRSWHLLVFWILFENVMSMHRSKATIIGLVEASRANEWVVTEKLGSVTSSTPAATTTMATNK
GATKKKKSQSSILAPEIVMGLCLLYCAVYDIVFGHDHFYVYLLMQSAAAFVIGFGYVGSQ

>CSLA_1AS_TGACv1_019142_AA0061550.1
_Triticum_aestivumMSMLPMARAAWLVLRYAVVVPLLQLAIYLCVVMSLMLFADRLYMGLVVAVLWLYRRCRNRN
QRNKGDDDNLESDDADRPMVLVQIPMFNEKQVFRLSIGAACGLWWPADKLVIQVLDDSTDAGIRSLVEAECRRWAG
KGVHIRYENRSNRSGYKAGAMRDGLKKQYVKDCEFVAVFDADFQPDADFLRHTVPVLEADPAVALVQARWRFVNAD
ECILTRMQEMSLDYHFSVEQEVGSAFHGFFSFNGTAGVWRLHALADAGGWKDRTTVEDMDLAVRASMRGWRFVY
AGDVQVRNELPSSFKAYRYQQHRWSCGPPNLMRKMFWEIVANKQVSAWKKLHVLYGFFFVRKVVAHLATFLFCCVVI
PVYVLVGGQDVWLPQYVPMYVAAVLTLLNAVCTPRSCHLLVFWILFENVMSIHRCKATIIGLLEASRANEWVVTEKLG
GSTTSTPAAATTTMVAKKKKSSSSFLAPEIVMGLFLLYCALYDIVFGHDHFYVYLLMQSAAAFVIGFGYVGSQ

>CSLA_3AS_TGACv1_210508_AA0674280.1_Triticum_aestivumMAMAATAWLWVEVPVRVDWPAVAAQCA
WAGEQARAFLVVPAVRLLVLLSLAMTVMILLEKLFVAAVCYAAKAFGHRPESRYQWRPIAASACKTGGDDEEDGIVVV
GSAAFPVVLVQIPMYNEREVYKVSIGAACALEWPSDRMVIQVLDDSTDPVVKDLVKIECQRWKSKGVNIRYEVRQNRK
GYKAGALKEGLMRDYVRECEFIAMFDADFQPESDFLLRTVPFLVHNPDIALVQTRWKFVNSDECLLTRFQEMSLDYHFK
FEQEAGSIVYSFFGFNGTAGVWRISAINDAGGWKERTTVEDMDLAVRTALLGLKFVYVGAVKVKSELPSTFKAYRFQQ
HRWSCGPANLFKKVLVEILHNKKVSFWCKLHLLYDFFFVGKITAHTVTFIYYCFAIPVSVFFPEIQIPLWCVVYVPTVITLCK
ALGSPSSFHLVILWVLFDNVMSLHRIKATITGLLDARRVNEWVVTEKLGDANKTEPAVEGLNDVQVIDVELSTPLVPKLE
KRRTRLWDKYNCSEIFVGTCIIICGCYDVLYANKGYYIYLFIQGLAFLVIGFEYIGTRPPNTE

>CSLA_3DS_TGACv1_272005_AA0912960.1_Triticum_aestivumMAATAWLWAEVPVPVRVDWAAVAAQCA
WAGQQARALLVVPTVRLLVLLSLAMTVMILLEKLFVAAVCYAAKAFGHRPESRYRWRPIAASACKAGGGDEEDGIVIV
GSSSGSAAFPVVLVQIPMYNEREVYKVSIGAACALEWPSDRMVIQVLDDSTDPVVKDLVKIECQRWKSKGVNIRYEVRE
NRKGYKAGALKQGLMRDYVRECEFIAMFDADFQPESDFLLRTVPFLVHNPDIALVQTRWKFVNSDECLLTRFQEMSLD
YHFKFEQEAGSIVYSFFGFNGTAGVWRISAINDAGGWNDRTTVEDMDLAVRTALLGWKFVYVGDVKVRSELPSTFKAY
RFQQHRWSCGPANLFKKMLVDILENKKVSFWSKLHLLYDFFFVGKIAAHTLTFIYYCFAIPVSVFFPEIQIPLWGVVYVPT
VITLCKSLGSPSSFHLVILWVLFENVMSLHRIKATITGLLDTRRVNEWVVTEKLGDANKTEPAMEGLDDVQVIDVELSTPL
VPKLEKRRTRLWDKYNCSEIPVGTCIIICGCYDVLYAKKGYYIYLFIQGLAFLVVGFEYIGTRPPSAE

>CSLA_3B_TGACv1_223332_AA0780350.1_Triticum_aestivumMLLLKIDIAKAFDTVSWEYILELLQRMNFPAH
WRDRIALLLSSVSSAYLLKGDPGPAILHQRGLRQGDPLSAILFILVIVPLHRMLEAAQQAGTIAPLPAGAARLRVTLYADD
AIFFANPVRQEIDTIMQLLQGFGEAAGLRGNPQKSSAATLNYGSIDLIDVLKNFSGTRVGFPIRYLGLPLCIGRLPLCTRVG
FPIRYLGWLLGKANSCIAPPLAVASHVLVRCVLSALPAFAMAVLRIPKRFYKDVDKARWRFLWVHDHEVTGGRCKVNW
RLVTSPVDHGGLGIPSMERFARALCLRWLWLAWTDPARPWARMGTPCDDKDRALFASATTVTVGDGNRVLFWHCS
WLGEQPVRQDYPNLFRRSTRKNRMMADAIRDDRWIMDLRRSGAGEEVMAMAATAGLWAEVPVRLDWATVAAQC
ALAGEQARAFLVVPAVRLLVLLSLAMTVMILLEKLFVAAVCYAAKALGHRPERRYKWGPVAASACKTGGDDEEDGIVG
VGSGSGSAAFPVVLVQIHMYNEREDLVKIECQRWKSKGVNIRYEVRQNRKGYKAGALKEGLIRDYVRECEFIAMFDAD
FQPESDFLLRTVPFLVHNPDIALVQTRWKFVNSDECLLTRFLEMSLDYHFKFEQEAGSIVYSFFGFNGTAGVWRISAIND
AGGWKERTMVEDMDLVVRTALLGLKFVYIGAVKVKSELPSTFKAYRFQQHRWSCGPANLFKKMLVEILQNKKVSFWS
KLHLLYDFFFVGKIIAHIVTFIYYCFATPVSVFFPEIQIPLWGVVYVPTVITLCKALGSPSSFHLVILWVLFDNVMSLHRIKATI
TGLLDARRVNEWVVTEKLGDTNKTEPAMEGLNDVQVIDVELSTPLVPKLEKRYNCSEIFVGTCIIICGCYDVLYANKGCYI
YLFIQGVAFLVIGFEYIGTRPPGAE

>CSLC_1DL_TGACv1_062162_AA0209740.1_Triticum_aestivumMAPWNGLWGGRAAIAGGNAYRDMPVIVK
MENPNWSISEINGGGDNGEDFLARVGGQRRRVKNTKQITWVFRLKAHRAAGCLARLTSAAVALGGAARRRVVAGRT

DSDAADGECEDVEERDPASRRSRFYTLIKACLMMSVFLLVVELAAYSNGRVNLAIFINSFNTSWIRFRATYVAPPLQLLA
DACVVLFLVQSADRLFQSLGCFYILVKRIKPKPLSPALADAEDPDAGYYPMVLVQIPMCNEKEVYRQSIAAVCNLDWPR
SNFLVQVLDDSDDVATQALIKEEVEKWRHSGAHIVYRHRVLREGYKAGNLKSAMSCSYVKDYEYVAIFDADFQPYPDFL
KRTVPHFKDNEDLGLVQARWSFVNKDENLLTRLQNINLCFHFEVEQQVNGVFINFFGFNGTAGVWRIKAVEDSGGW
MERTTVEDMDIAVRAHLKGWKFVFLNDVECQCELPESYEAYRKQQHRWHSGPMQLFRLCLPDIIRCKIVFWKKANLIF
LFFLLRKLILPFYSFTLFCIILPMTMFVPEAELPDWVVCYIPVLMSFLNIAPAPKSFPFIIPYLLFENTMSVTKFNAMISGLFQ
LGSTYEWVVTKKSGRSLEGDLISLAPKGLKQLKYGSVPAINVAIKEQSKAKKESKKYNRIYKKELAMSLLLLSAAARSLLSK
QGIHFYFLLFQGISFLLVGLDLIGQDIK

>CSLC_1BL_TGACv1_030501_AA0092480.1_Triticum_aestivumMENPNWSISEINIDDDNSEDFLARVGGQRR
RVKNTKQITWVFRLKAHRAAGCLSWLTSAAFALGGATRRRVVAGRTDSNATDGECKDVEEWAPASRRSRFYTLIKACL
MMFVCLLIVELAAYSNGKGNLAVFINSFNTSWIRFRAAYIAPPLQLLANACVVLFLVQSADRVFQSLGCFYILVKRIKPKPL
FLALSDAEDPDAGYYPMVLVQIPMCNEKEVYRQSIAAVCNLDWPRSNFLVQVLDDSDDVTTQALIKDEVEKWRHSGA
HIVYRHRVLREGYKAGNLKSAMSCSYVKDYEYVAIFDADFQPYPDFLKRTVPHFKDNEDLGLVQARWSFVNKDENLLTR
LQNINLCFHFEVEQQVNGVFINFFGFNGTAGVWRIKAVEDSGGWMERTTVEDMDIAGWKFVFLNDVECQCELPETY
EAYRKQQHRWHSGPMQLFRLCLPDIIRCKIVFWKKANLIFLFFLLRKLILPFYSFTLFCIILPMTMFVPEAELPDWVVCYIP
VLMSFLNIAPAPKSFPFIIPYLLFENTMSVTKFNAMISGLFQLGSTYEWVVTKKSGRSLEGDLISLAPKGLKQLKYGSVPAI
NVAIKEKLKAKKESKKYNRIYKKELAMSLLLLSAAIRSLLSKQGIHFYFLLFQGISFLLVGLDLIGQDIK

>CSLC_5BL_TGACv1_404820_AA1311790.1_Triticum_aestivumMAPWTGLWGARAGAGAGAGAYRGTPVVV
KMENPNWSISEISPEDAEDEDFLVSGAGAARRSRKGGRGKNAKQITWVLLLKAHRAAGCLASLASAAVTLGAAARRRV
ADGRTDADAGAPGSAGESPVLRSRFYAFIRAFLLLSLLLLAVELAARLHGWDLAASALALPIIGVESLYASWLRLRAAYLAP
LLQFLTDACVVLFLIQSADRLIQCLGSFYITVKRIKPTLKSPALPDAEDPDAGYYPMVLVQIPMCNEKEVYQQSIAAVCNL
DWPRSNFLVQVLDDSDDPTTQSLIREEVAKWQQTGARILYRHRVLRDGYKAGNLKSAMACSYVKDYEFVAIFDADFQP
NPDFLKRTVPHFKDNDELGLVQARWSFVNKDENLLTRLQNINLCFHFEVEQQVNGVFLNFFGFNGTAGVWRIKALEES
GGWMERTTVEDMDIAVRAHLHGWKFIFLNDVECQCELPESYEAYRKQQHRWHSGPMQLFRLCIPDIIKSKISVWKKF
NLIFLFFLLRKLILPFYSFTLFCIILPMTMFVPEAELPDWVVCYIPALMSLLNILPSPKSFPFIIPHLLFENTMSVTKFNAMISG
LFQLGSAYEWVVTKKSGRSSEGDLISLAAAAPPRELRHHPKTGSAPSLEALMVLKEQQPSPKKEGKKQQKKHNRIYKKEL
ALSLLLLTAAARSLLTKQGIHFYFLLFQGISFLLVGLDLIGEQVE

>CSLC_5DL_TGACv1_435778_AA1454840.1_Triticum_aestivumMAPWTGLWGARAGAGAGAYRGTPVVVK
MENPNWSISEISPEDAEDEDFLVSGAGAARRRKGGRGKNAKQITWVLLLKAHRAAGCLASLASAAVTLGAAARRRVA
DGRTDADAGATPGSAGESPVLRSRFYAFIRAFLLLSLLLLAVELAARFHGWDLAASALALPIIGVESLYASWLRLRAAYLAP
LLQFLTDACVVLFLIQSADRLIQCLGSFYITVKRIKPRLKSPALPDAEDPDAGYYPMVLVQIPMCNEKEVYQQSIAAVCNL
DWPRSNFLVQVLDDSDDPTTQSLIREEVAKWQQTGARILYRHRVLRDGYKAGNLKSAMACSYVKDYEFVAIFDADFQP
NPDFLKRTVPHFKDNDELGLVQARWSFVNKDENLLTRLQNINLCFHFEVEQQVNGVFLNFFGFNGTAGVWRIKALEES
GGWMERTTVEDMDIAVRAHLHGWKFIFLNDVECQCELPESYEAYRKQQHRWHSGPMQLFRLCIPDIIKSKISVWKKF
NLIFLFFLLRKLILPFYSFTLFCIILPMTMFVPEAELPDWVVCYIPALMSLLNILPSPKSFPFIIPYLLFENTMSVTKFNAMISGL
FQLGSAYEWVVTKKSGRSSEGDLISLAAAPPRELRHHPKTGSAPSMEALMVLKEQPSPKKEGKKQQKKHNRIYKKELAL
SLLLLTAAARSLLTKQGIHFYFLLFQGISFLLVGLDLIGEQVE

>CSLC_5AL_TGACv1_374268_AA1195590.3_Triticum_aestivumMAPWTGLWGARAGAGAYRGTPVVVKME
NPNWSISEISPEDAEDEDFLVSGAARRKGGRGKNAKQITWVLLLKAHRAAGCLASLASAAVTLGAAARRRVADGRTDA

DAGAPGPARESPVLRSRFYAFIRAFLLLSLLLLAVELAARFHRWDLAASALALPIIGVESLYASWLRLRAAYLAPLLQFLTDA
CVVLFLIQSADRLIQCLGSFYITVKRIKPRLRSPALPDAEDPDAGYYPMVLVQIPMCNEKEVYQQSIAAVCNLDWPRSNF
LVQVLDDSDDPTTQSLIREEVAKWQQTGARILYRHRVLRDGYKAGNLKSAMACSYVKDYEFVAIFDADFQPNPDFLKR
TVPHFKDNDELGLVQARWSFVNKDENLLTRLQNINLCFHFEVEQQVNGVFLNFFGFNGTAGVWRIKALEESGGWME
RTTVEDMDIAVRAHLHGWKFIFLNDVECQCELPESYEAYRKQQHRWHSGPMQLFRLCIPDIIKSKISVWKKFNLIFLFFL
LRKLILPFYSFTLFCIILPMTMFVPEAELPDWVVCYIPALMSLLNILPSPKSVPFIIPYLLFENTMSVTKFNAMISGLFQLGSA
YEWVVTKKSGRSSEGDLISLAAAAPPRELRQQQKTGSAPSLEALMVLKEEQASPRKEGKKQKKHNRIYKKELALSLLLLTA
AARSLLTKQGIHFYFLLFQGISFLLVGLDLIGEQVE

>CSLC_1DL_TGACv1_061928_AA0205730.1_Triticum_aestivumMAPSFWGREARLSDGGGGTPVVVKMENP
NWSISEMEQEAVPGSPAGLAAGKAGRGKNARQITWVLLLKAHRAAGRLTGAASAALAVAAAARRRVAAGRTDGDAA
PGESTALRARFYGCLRLFVVLSMLLLAVEVAAYLQGWHLQMPEMPEMPGQLAMDGLLAVDGLAASAYAGWMRVRL
QYIAPPLQFLTNSCVVLFMIQSVDRLVLCLGCLWIKLRGIKPVPIAADKDDVEAGDEDFPMVLVQMPMCNEREVYQQSI
GAICALDWPRSNFLVQVLDDSDDATTSALIKEEVEKWQREGVRIVYRHRVIRDGYKAGNLKSAMNCSYVKDYEYVVIFD
ADFQPQADFLKRAMPHFKGKDDVGLVQARWSFVNNDENLLTRLQNINLCFHFEVEQQVNGAFLNFFGFNGTAGVW
RIKALEDSGGWMERTTVEDMDIAVRAHLKGWKFLYLNDVECQCELPESYEAYRKQQHRWHSGPMQLFRLCFVDIIKS
KIGFWKKCNLIFLFFLLRKLILPFYSFTLFCVILPMTMFVPEAELPAWVVCYIPATMSIMSILPSPKSFPFIVPYLLFENTMSV
TKFNAMISGLFQLGSAYEWVVTKKSGRSSEGDLVALVEKHTVQQQQRVGSAPDLAGLAAKDSSLPKKDAPKKKQKHN
RIYRKELALSFLLLTAAARSVLSAQGIHFYFLLFQGVSFLVMGLDLIGEQVE

>CSLC_1BL_TGACv1_030750_AA0099830.1_Triticum_aestivumMAPSFWGREARLSDGGGGTPVVVKMENP
NWSISEMEQEAVPGSPAGLAAGKAGRGKNARQITWVLLLKAHRAAGRLTGAASAALAVAAAARRRVAAGRTDGDAA
PGESTALRARFYGCLRLFVVLSMLLLAVEVAAYLQGWHLQMPEMPEMPGQLAMDGLLAVDGLAAAAYAGWMRVRL
QYIAPPLQFLTNSCVVLFMIQSVDRLILCLGCLWIKLRGIKPVPIAADKDDVEAGEEDFPMVLVQMPMCNEREVYQQSI
GAICALDWPRSNFLVQVLDDSDDATTSALIKEEVEKWQREGVRIVYRHRVIRDGYKAGNLKSAMNCSYVKDYEYVVIFD
ADFQPQADFLKRAMPHFKGKDDVGLVQARWSFVNNDENLLTRLQNINLCFHFEVEQQVNGAFLNFFGFNGTAGVW
RIKALEDSGGWMERTTVEDMDIAVRAHLKGWKFLYLNDVECQCELPESYEAYRKQQHRWHSGPMQLFRLCFVDIIKS
KIGFWKKCNLIFLFFLLRKLILPFYSFTLFCVILPMTMFVPEAELPAWVVCYIPATMSIMSILPSPKSFPFIVPYLLFENTMSV
TKFNAMISGLFQLGSAYEWVVTKKSGRSSEGDLVALVEKHTVQQQQRVGSAPDLAGLAAKDSSLPKKDAPKKKQKHN
RIYRKELALSFLLLTAAARSVLSAQGIHFYFLLFQGVSFLVMGLDLIGEQVE

>CSLC_1AL_TGACv1_001272_AA0028090.1_Triticum_aestivumMAPSFWGREARLSDGGGGTPVVVKMENP
NWSISEMEQEPVPGSPAGLAAGKAGRGKNARQITWVLLLKAHRAAGRLTGAASAALAVAAAARRRVAAGRTDGDAA
PGESTALRARFYGCLRVFVVLSMLLLAVEVAAYLQGWHLQMPQMPEMPGQLAMDGLLAVDGLAAAAYAGWMRVR
LQYIAPPLQFLTNSCVVLFMIQSVDRLVLCLGCLWIKLRGIKPVPIAADKDDVEAGDEDFPMVLVQMPMCNEREVYQQ
SIGAICALDWPRSNFLVQVLDDSDDATTSALIKEEVEKWQREGVRIVYRHRVIRDGYKAGNLKSAMNCSYVKDYEYVVIF
DADFQPQADFLKRAMPHFKGKDDVGLVQARWSFVNNDENLLTRLQNINLCFHFEVEQQVNGAFLNFFGFNGTAGV
WRIKALEDSGGWMERTTVEDMDIAVRAHLKGWKFLYLNDVECQCELPESYEAYRKQQHRWHSGPMQLFRLCFVDII
KSKIGFWKKCNLIFLFFLLRKLILPFYSFTLFCVILPMTMFVPEAELPAWVVCYIPATMSIMSILPSPKSFPFIVPYLLFENTM
SVTKFNAMISGLFQLGSAYEWVVTKKSGRSSEGDLVALVEKHTAQQQQRVGSAPDLAGLAAKDSSLPKKDAPKKKQKH
NRIYRKELALSFLLLTAAARSVLSAQGIHFYFLLFQGVSFLVMGLDLIGEQVE

>CSLC_3DL_TGACv1_251593_AA0882850.1_Triticum_aestivumMAPWWGQEARGGVSGGVTGTPVVVKMQ
TPDWAISEVPPPGSPAAGGKDGRGKNARQITWVLLLKAHRAAGKLTGAATAALSVAAAARRRVAAGRTDSDADNAP
PGLGGSPALRTRLYGFIRASLLLSVLLLAADVAAHAQGWHLAALPDLEAVEGLFAAGYAAWMRARAAYLGPALQFLTN
ACVVLFMIQSADRLILCLGCFWIKLRGIRPVPNAAAAAGNGNGKGSDDVEAGAQEEGDFPMVLVQIPMCNEKEVYQQ
SIGAVCNLDWPRSNFLVQVLDDSDDAATSALIREEVEKWQREGVRVLYRHRVIRDGYKAGNLKSAMNCSYVKDYEFVV
IFDADFQPQEDFLKLTVPHFKGKEDVGLVQARWSFVNKDENLLTRLQNINLCFHFEVEQQVNGAFLNFFGFNGTAGV
WRIKALEDSGGWMERTTVEDMDIAVRAHLKGWKFLYLNDVECQCELPESYEAYRKQQHRWHSGPMQLFRLCFVDII
KSKIGFWKKFNLIFLFFLLRKLILPFYSFTLFCVILPMTMFAPEAELPAWVVCYIPATMSLLNILPAPKSFPFIVPYLLFENTM
SVTKFNAMISGLFQLGSAYEWVVTKKSGRSSEGDLVALVENEKQSKQLRVGSAPNLDSLAAKEELYPKAEPKPKKKKHN
RLYRKELALSFLLLTAAARSLLSVQGIHFYFLLFQGVSFLVVGLDLIGEQVE

>CSLC_3AL_TGACv1_197197_AA0665370.1_Triticum_aestivumMAPWWGQEARGGVSGGVTGTPVVVKMQ
TPDWAISEVPPPGSPAAGGKDGRGKNARQITWVLLLKAHRAAGKLTGAATAALSVAAAARRRVAAGRTDSDADADG
APPGPGAGRPALRTRLYGFIRASLLLSLLLLAADVAAHAQGWHLAALPDLEAVEGLFAAGYAAWMRARAAYLGPALQF
LTNACVVLFMIQSADRLILCLGCFWIKLRGIRPVPNAATAGNGKGSDDVEAGAQEEEEGEFPMVLVQIPMCNEKEVYQ
QSIGAVCNLDWPRSNFLVQVLDDSDDAATSALIREEVEKWQREGVRILYRHRVIRDGYKAGNLKSAMNCSYVKDYEFV
VIFDADFQPQEDFLKLTVPHFKGKEDVGLVQARWSFVNKDENLLTRLQNINLCFHFEVEQQVNGAFLNFFGFNGTAGV
WRIKALEDSGGWMERTTVEDMDIAVRAHLKGWKFLYLNDVECQCELPESYEAYRKQQHRWHSGPMQLFRLCFVDII
KSKIGFWKKFNLIFLFFLLRKLILPFYSFTLFCVILPMTMFAPEAELPAWVVCYIPATMSLLNILPAPKSFPFIVPYLLFENTM
SVTKFNAMISGLFQLGSAYEWVVTKKSGRSSEGDLVALVENEKQSKQQRVGSAPNLDSLAAKEELYPKSEPKKKKHNRL
YRKELALSFLLLTAAARSLLSVQGIHFYFLLFQGVSFLVVGLDLIGEQVE

>CSLC_3DS_TGACv1_271926_AA0910940.1_Triticum_aestivumMASSWWGDKEEHGTPVVVKMDNPYSLVEI
DGPGMDSSEKARRSKNAKQFKWVLLLRAHRAVGCVAWLAGGFWGLLGAVNRRVRRSRDADAEPDAEASGRGRHM
LGFLRAFLLLSLAMLAFETAAYLKGWHYFPRDLPEHYLRQLPEHLQNLPEHLRHLPENLRHLPENLRHLPDGLRMPEQQ
EIQGWLHRAYVAWLAFRIDYIAWAIEKLSGFCIVLFMVQSIDRILLCLGCFWIKLRGIKPGLKAAANKRGSKYADDDDLE
DGDDLGAYFPMVLLQMPMCNEKEVYETSISHVCQIDWPRDRMLVQVLDDSDDETCQMLIRAEVTKWNQRGVNIIYR
HRLSRTGYKAGNLKSAMSCEYVKDYEFVAIFDADFQPNPDFLKLTVPHFKGNPELGLVQARWSFVNKDENLLTRLQNIN
LCFHFEVEQQVNGIYLNFFGFNGTAGVWRIEALEDSGGWMERTTVEDMDIAVRAHLQGWKFIYLNDVKVLCELPESY
QAYRKQQHRWHSGPMQLFRLCLPAIIKSKIPLWKKANLVMLFFLLRKLILPFYSFTLFCVILPLTMFVPEAELPIWVICYVP
MIMSVLNILPAPKSFPFVIPYLLFENTMSVTKFNAMVSGLFQLGSSYEWVVTKKAGRTSSESDIFAMAEETDTATRPAPR
LVRGVSEAGLEAWAKTHQLDNKDLQLKAQAEEVTSLAAAIKKTSKAKPPNRIFKKELALAFLLLIAATRSLLSAQGLHFYFL
LFQGVTFLVVGLDLIGEQVS

>CSLC_3B_TGACv1_220758_AA0718310.2_Triticum_aestivumMASSWWGDKEEHGTPVVVKMDNPYSLVEID
GPGMDSSEKARRSKNAKQFKWVLLLRAHRAVGCVAWLAGGFWGLLGAVNRRVRRSRDADAEPDAEASGRGRHML
GFLRAFLLLSLAMLAFETAAYLKGWHYFPRDLPEHYLRQLPEHLQNLPENLRHLPENLRHLPDGLRMPEQQEIQGWLH
RAYVAWLAFRIDYIAWAIEKLSGFCIVLFMVQSIDRILLCLGCFWIKLRGIKPGLKAAASKRGSKYADENDLEDGDDLGAY
FPMVLLQMPMCNEKEVYETSISHVCQIDWPRDRMLVQVLDDSDDETCQMLIRAEVTKWSQRGVNIIYRHRLSRTGYK
AGNLKSAMSCEYVKDYEFVAIFDADFQPNPDFLKLTVPHFKGNPELGLVQARWSFVNKDENLLTRLQNINLCFHFEVEQ
QVNGIYLNFFGFNGTAGVWRIEALEDSGGWMERTTVEDMDIAVRAHLQGWKFIYLNDVKVLCELPESYQAYRKQQH
RWHSGPMQLFRLCLPAIIKSKIPLWKKANLVMLFFLLRKLILPFYSFTLFCVILPLTMFVPEAELPIWVICYVPMIMSVLNIL
PAPKSFPFVIPYLLFENTMSVTKFNAMVSGLFQLGSSYEWVVTKKAGRTSSESDIFAMAEETNTATRPAPRLVRGVSEA

GLEAWAKTHQLDNKDLQLKAEAEEVTSLAAAIKKTSKAKPPNRIFKKELALAFLLLIAATRSLLSAQGLHFYFLLFQGVTFL
VVGLDLIGEQVS

>CSLC_3AS_TGACv1_211225_AA0686890.2_Triticum_aestivumMASSWWGDKEEHGTPVVVKMDNPYSLVEI
DGPGMDSSEKARRSKNAKQFKWVLLLRAHRAVGCVAWLAGGFWGLLGAVNRRVRRSRDADAEPDAEASGRGRHM
LGFLRAFLLLSLAMLAFETAAYLKGWHYFPRDLPEHYLRQLPEHLQNLPEHLRHLPENLRHLPDGLRMPEQQEIQGWLH
RAYVAWLAFRIDYIAWAIEKLSGFCIVLFMVQSIDRILLCLGCFWIKVRGIKPGLVATKKRGNKYADDNDLEDGDDLGAY
FPMVLLQMPMCNEKEVYETSISHVCQIDWPRDRMLVQVLDDSDDETCQMLIRAEVTKWNQRGVNIIYRHRLSRTGYK
AGNLKSAMSCEYVKDYEFVAIFDADFQPNPDFLKLTVPHFKGNPELGLVQARWSFVNKDENLLTRLQNINLCFHFEVEQ
QVNGIYLNFFGFNGTAGVWRIEALEDSGGWMERTTVEDMDIAVRAHLQGWKFIYLNDVKVLCELPESYQAYRKQQH
RWHSGPMQLFRLCLPAIIKSKIPLWKKANLVMLFFLLRKLILPFYSFTLFCVILPLTMFVPEAELPIWVICYVPMIMSVLNIL
PAPKSFPFVIPYLLFENTMSVTKFNAMVSGLFQLGSSYEWVVTKKAGRTSSESDIFAMAEKTDTATRPAPRLVRGVSEA
GLEAWAKTHQLDNKDLQLKAEAEEVTSLAAAIKKTSKAKPPNRIFKKELALAFLLLIAATRSLLSAQGLHFYFLLFQGVTFL
VVGLDLIGEQVS

>CSLD_2BS_TGACv1_148683_AA0494520.1_Triticum_aestivumMSKAPRNPGGGSAGAPKSSSGQPVKFARRT
PSGRYLSLSREDIDMEGEMGPDYANYTVHIPPTPDNQPMKDGSEPTAVAMKAEEQYVSNSLFTGGFNSVTRAHLMD
RVIDSDVKHPQMAGAKATRCAMPACDGKVMRNERGEEVDPCECRFKICRDCYLDAQKDGCLCPGCKEHYKIGDYAD
DDPHDVSAGKSLLARNQNGEFDHNRWLFESSGTYGYGNAFMPKGGMYEDDLDEDGAGGDGGMPADLSQKPFKPL
TRKIPMPTSIISPYRIFIVIRFFVLIFYLTWRIRNPNMEALWLWGMSIVCELWFAFSWLLDMLPKVNPINRSTDLAVLKEKF
ETHSPSNPHGRSDLPGLDVFVSTADPEKEPVLTTANTILSILAVDYPVEKLACYVSDDGGALLTFEAMAEAASFANIWVP
FCKKHDIEPRNPDSYFALKGDPTKGKRRSDFVKDRRKVKREYDEFKVRINGLPDSIRRRSDAFNAREDMKMLKHLRETG
ADPSEQPKVKKATWMADGTHWPGTWAVSSPDHAKGNHAGILQVMLRPPSPDPLYGMHDEDQLVDYSDVDTRLP
MLVYMSREKRPGYDHNKKAGAMNALVRCSAVMSNAPFILNFDCDHYINNTQAIREAMCFMMDRGGERICYIQFPQR
FEGIDPSDRYANHNTVFFDGNMRALDGLQGPMYVGTGCMFRRFALYGFDPPRTAEYTGWLFKKKKVTNFKDPESDT
QQLKAEDFDAELTAQLVPRRFGNSSAMLASIPIAEFQARPIADHPAVLHGRPPGTLTVPRPPLDPPTVAEAVSVISCWYE
DKTEWGDRVGWIYGSVTEDVVTGYRMHNRGWRSVYWISKRDAFLGTAPINMTDRLHQVLRWATGSVEIFFSRNNA
FLASRKLMFLQRVAYLNVGIYPFTSIFLLTYCFIPALSLFSGFFIVQTLNVAFLFYLLTITVTLIALGILEVKWSGIELEDWWRN
EQFWLISGISAHLYAVVQGLLKVMAGIEISFTLTAKAAAEDNEDIYADLYVVKWSSLLIPPITIGMLNIIAIAFAFARTIYSD
NPRWGKFIGGGFFSFWVLAHLNPFAKGLMGRRGKTPTIIFVWSGLISITISLLWVALSPPEANSTGGARGGGFQFP

>CSLD_2DS_TGACv1_177279_AA0572180.1_Triticum_aestivumMSKAPRNPGGGSAGAPKSSSGQPVKFARR
TPSGRYLSLSREDIDMEGEMGPDYANYTVHIPPTPDNQPMKDGAERTAVAMKAEEQYVSNSLFTGGFNSVTRAHLM
DRVIDSDVKHPQMAGARPARCAMPACDGKVMRNERGEEIEPCECRFKICRDCYLDAQKDGCLCPGCKEHYKIGDYAD
DDTHDVSAGKSLLARNQNGEFDHNRWLFESSGTYGYGNAFMPKGGMYEDDLDEDGAAGDDGMQDMNQKPFKPL
TRKIPMPASIISPYRIFIVIRFFVLIFYLTWRIRNPNMEALWLWGMSIVCELWFAFSWLLDMLPKVNPINRSTDLAVLKEK
FETPSPSNPHGRSDLPGLDVFVSTADPEKEPVLTTANTILSILAVDYPVEKLACYVSDDGGALLTFEAMAEAASFANIWVP
FCKKHDIEPRNPDSYFALKGDPTKGKRRSDFVKDRRKVKREYDEFKVRINGLPDSIRRRSDAFNAREDMKMLKHLRETG
ADPSEQPKVKKATWMADGTHWPGTWAVSSPDHAKGNHAGILQVMLRPPSPDPLYGMHDEDQLIDYSDVDTRLPM
LVYMSREKRPGYDHNKKAGAMNALVRCSAVMSNAPFILNFDCDHYINNNQAVREAMCFMMDRGGERICYIQFPQR
FEGIDPSDRYANHNTVFFDGNMRALDGLQGPMYVGTGCMFRRFALYGFDPPRTAEYTGWLFKKKKVTNFKDPESDT
QKLKAEDFDAELTAQLVPRRFGNSSAMLASIPIAEFQARPIADHPAVLHGRPPGTLTVPRPPLDPPTVAEAVSVISCWYE
DKTEWGDRVGWIYGSVTEDVVTGYRMHNRGWRSVYWISKRDAFLGTAPINMTDRLHQVLRWATGSVEIFFSRNNA

FLASRKLMFLQRVAYLNVGIYPFTSIFLLTYCFIPALSLFSGFFIVQTLNVAFLFYLLTITVTLIALGILEVKWSGIELEDWWRN
EQFWLISGISAHLYAVVQGLLKVMAGIEISFTLTAKAAAEDNEDIYADLYVVKWSSLLIPPITIGMLNIIAIAFAFARTIYSD
NPRWGKFIGGGFFSFWVLAHLNPFAKGLMGRRGKTPTIIFVWSGLISITISLLWVALSPPEANSTGGARGGGFQFP

>CSLD_2AS_TGACv1_114244_AA0365360.1_Triticum_aestivumMSKAPRNPGGGSAGAPKSSSGQPVKFARR
TPSGRYLSLSREDIDMEGEMGPDYANYTVHIPPTPDNQPMKDGAEPTAVAMKAEEQYVSNSLFTGGFNSVTRAHLM
DRVIDSDVKHPQMAGAKATRCAMPACDGKVMRNERGEEIDPCECRFKICRDCYLDAQKDGCLCPGCKEHYKIGDYAD
DDPHDVSSGKSLLARNQNGEFDHNRWLFESSGTYGYGNAFMPKGGMYEDDLDEDGVGGDGGMQDMNQKPFKPL
TRKIPMPTSIISPYRIFIVIRFFVLIFYLTWRIRNPNMEALWLWGMSIVCELWFAFSWLLDMLPKVNPINRSTDLAVLKEKF
ETPSPSNPHGRSDLPGLDIFVSTADPEKEPVLTTANTILSILAVDYPVEKLACYVSDDGGALLTFEAMAEAASFANIWVPF
CKKHDIEPRNPDSYFALKGDPTKGKRRSDFVKDRRKVKREYDEFKVRINGLPDSIRRRSDAFNAREDMKMLKHLRETGA
DPSEQPKVKKATWMADGTHWPGTWAVSSPDHAKGNHAGILQVMLRPPSPDPLYGMHDEDQLIDYSDVDTRLPML
VYMSREKRPGYDHNKKAGAMNALVRCSAVMSNAPFILNFDCDHYINNNQAVREAMCFMMDRGGERICFIQFPQRF
EGIDPSDRYANHNTVFFDGNMRALDGLQGPMYVGTGCMFRRFALYGFDPPRTAEYTGWLFKKKKVTNFKDPDSDTQ
QLKAEDFDAELTAQLVPRRFGNSSAMLASIPIAEFQARPIADHPAVLHGRPPGTLTVPRPPLDPPTVAEAVSVISCWYED
KTEWGDRVGWIYGSVTEDVVTGYRMHNRGWRSVYWISKRDAFLGTAPINMTDRLHQVLRWATGSVEIFFSRNNAFL
ASRKLMFLQRVAYLNVGIYPFTSIFLLTYCFIPALSLFSGFFIVQTLNVAFLFYLLTITITLIALGILEVKWSGIELEDWWRNEQ
FWLISGISAHLYAVVQGLLKVMAGIEISFTLTAKAAADDNEDIYADLYVVKWSSLLIPPITIGMLNIIAIAFAFARTIYSENPR
WGKFIGGGFFSFWVLAHLNPFAKGLMGRRGKTPTIIFVWSGLISITISLLWVALSPPEANSTGGARSGGFQFP

>CSLD_1BL_TGACv1_030586_AA0094860.1_Triticum_aestivumMGSKGILKNSGSSRMPPHGPSKPPTAPTSAP
QVVFGRRTESGRFISYSRDDLDSEISSVDFQDYHVHIPMTPDNQPMEEDGTKADEQYVSSSLFTGGFNSVTRAHVMDK
QGPDSDIGRSGPKGSICMVEGCDSKIMRNGRGEDILPCECDFKICVDCFTDAVKGGGGVCPGCKELYKHTEWEEVLSN
SSNELTRALSLPHGPGGKMERRLSLVKQGTMNNQSGEFDHNRWLFETKGTYGYGNAIWPDDNVDDDGRNGVPGHP
KELMSKPWRPLTRKLQIPAAVISPYRLLVLIRLVALAFFLMWRIKHQNDDAIWLWGMSIVCELWFAFSWVLDQLPKLCP
INRATDLSVLKEKFETPTPSNPTGKSDLPGIDIFVSTADPEKEPVLVTANTILSILAVDYPVDKLACYVSDDGGALLTFEAM
AEAASFANFWVPFCRKHDIEPRNPDSYFNLKRDPFKNKVKADFVKDRRRIKREYDEFKVRVNGLPDSIRRRSDAYHARE
EIQAMNLQREKIKAGGDEQFEPVKIPKATWMADSTHWPGTWIHSSQDHARGDHAGIIQVMLKPPSDMPMYGNIEK
SPLDFSEVDTRLPMLVYMSREKRPGYDHNKKAGAMNALVRASAIMSNGPFILNLDCDHYVYNSKAFREGMCFMMDR
GGDRLCYVQFPQRFEGIDPSDRYANHNTVFFDINMRALDGLQGPVYVGTGCLFRRIALYGFDPPRSKDHSPGFCGCCL
PRRRKASASNANPEETMALRMGDFDGDSMNLATFPKKFGNSSFLIDSIPVAEFQGRPLADHPSVKNGRPPGALTIPREI
LDASIVAEAISVVSCWYEEKTEWGTRVGWIYGSVTEDVVTGYRMHNRGWKSVYCVTQRDAFRGTAPINLTDRLHQVL
RWATGSVEIFFSRNNALFASSKMKVLQRIAYLNVGIYPFTSIFLIVYCFLPALSLFSGQFIVQTLNVTFLTYLLIITITLCLLAML
EIKWSGIALEEWWRNEQFWLIGGTSAHLAAVMQGLLKVVAGIEISFTLTSKQVGDDIDDEFAELYEVKWTSLMIPPLTII
MVNLVAIAVGFSRTIYSTDIDDEFAELYEVKWTSLMIPPLTIIMVNLVAIAVGFSRTIYSTIPQWSKLLGGVFFSFWVLAHL
YPFAKGLMGRRGRTPTIVYVWAGLVSITISLLWIAINPPSSAANQQLGGSFSFP

>CSLD_1AL_TGACv1_001700_AA0034150.2_Triticum_aestivumMGSKGILKNSGSSRVPPHGPSKPPTAPTSAP
QVVFGRRTESGRFISYSRDDLDSEISSVDFQDYHVHIPMTPDNQPMEEDGTKADEQYVSSSLFTGGFNSVTRAHVMDK
QGPDSDMGRSGPKGSICMVEGCDSKIMRNGRGEDILPCECDFKICVDCFTDAVKGGGGVCPGCKELYKHTEWEEVLS
NSSNELTRALSLPHGPGGKMERRLSLVKQGTMNNQSGEFDHNRWLFETKGTYGYGNAIWPDDNVDDDGRNGVPG
HPKELMSKPWRPLTRKLQIPAAVISPYRLLVLIRLVALAFFLMWRIKHQNDDAIWLWGMSIVCELWFALSWVLDQLPKL
CPINRATDLSVLKEKFETPTPSNPTGKSDLPGIDIFVSTADPEKEPVLVTANTILSILAVDYPVDKLACYVSDDGGALLTFEA

MAEAASFANFWVPFCRKHDIEPRNPDSYFNLKRDPFKNKVKADFVKDRRRIKREYDEFKVRVNGLPDSIRRRSDAYHAR
EEIQAMNLQREKIKAGGDEQFEPVKIPKATWMADSTHWPGTWIHPSQDHARGDHAGIIQVMLKPPSDMPMYGNIE
KSPLDFSGVDTRLPMLVYMSREKRPGYDHNKKAGAMNALVRASAIMSNGPFILNLDCDHYVYNSKAFREGMCFMMD
RGGDRLCYVQFPQRFEGIDPSDRYANHNTVFFDINMRALDGLQGPVYVGTGCLFRRIALYGFDPPRSKDHSPGFCGCC
LPRRRKASASNANPEETMALRMGDFDGDSMNLATFPKKFGNSSFLIDSIPVAEFQGRPLADHPSVKNGRPPGALTIPRE
ILDASIVAEAISVVSCWYEEKTEWGTRVGWIYGSVTEDVVTGYRMHNRGWKSVYCVTQRDAFRGTAPINLTDRLHQVL
RWATGSVEIFFSRNNALFASSKMKVLQRIAYLNVGIYPFTSIFLIVYCFLPALSLFSGQFIVQTLNVTFLTYLLIITVTLCLLAM
LEIKWSGIALEEWWRNEQFWLIGGTSAHLAAVMQGLLKVVAGIEISFTLTSKQVGDDIDDEFAELYEVKWTSLMIPPLTI
IMVNLVAIAVGFSRTIYSTIPQWSKLLGGVFFSFWVLAHLYPFAKGLMGRRGRTPTIVYVWAGLVSITISLLWIAINPPST
AANQQLGGSFSFP

>CSLD_1BS_TGACv1_049706_AA0160220.1_Triticum_aestivumMSCKMRGCDMLALAATRPMICEECYMDCV
AASGNCPGCKEAYSAGSDTDDSVDEDDDDAISSSEERDQMPMTSMSKRFSMVHSIKMPMSSSNDKPADFDHARWL
FETKGTYSYGNALWPENEHGGGGNNAGATFGFVGIEEPPNFATWMSDGSQWASTWLAGATDHARGNHAGIIQRP
GYNHNKKAGAMNALVRTSAIMSNGPFILNLDCDHYVHNSAALREGMCYMLDCRGDRVCYVQFPQRFEGIDPNDRYA
NHNLVFFDVAMRAMDGLQGPMYVGGCIFRRIALYGFSPPRATKHHGWLGRKIKLFLRKPTMGKKTDRELVMAILQK

>CSLD_1DL_TGACv1_063091_AA0223780.1_Triticum_aestivumMGSKGILKNSGSSRVPPHGPSKPPTAPTSAP
QVVFGRRTESGRFISYSRDDLDSEISSVDFQDYHVHIPMTPDNQPMEEDGTKADEQYVSSSLFTGGFNSVTRAHVMDK
QGPDSDMGRSGPKGSICMVEGCDSKIMRNGRGEDILPCECDFKICVDCFTDAVKGGRGVCPGCKELYKHTEWEEVLS
NSSNELTRALSLPHGPGGKMERRLSLVKQGTMNNQSGEFDHNRWLFETKGTYGYGNAIWPDDNVDDDGRNGVPG
HPKELMSKPWRPLTRKLQIPAAVISPYRLLVLIRLVALAFFLMWRIKHQNDDAIWLWGMSIVCELWFALSWVLDQLPKL
CPINRATDLSVLKEKFETPTPSNPTGKSDLPGIDIFVSTADPEKEPVLVTANTILSILAVDYPVDKLACYVSDDGGALLTFEA
MAEAASFANFWVPFCRKHDIEPRNPDSYFNLKRDPFKNKVKADFVKDRRRIKREYDEFKVRVNGLPDSIRRRSDAYHAR
EEIQAMNLQREKIKAGGDEQFEPVKIPKATWMADSTHWPGTWIHSSQDHARGDHAGIIQVMLKPPSDMPMYGNIE
KSPLDFSEVDTRLPMLVYMSREKRPGYDHNKKAGAMNALVRASAIMSNGPFILNLDCDHYVYNSKAFREGMCFMMD
RGGDRLCYVQFPQRFEGIDPSDRYANHNTVFFDINMRALDGLQGPVYVGTGCLFRRIALYGFDPPRSKDHSPGFCGCC
LPRRRKASASNANPEETMALRMGDFDGDSMNLATFPKKFGNSSFLIDSIPVAEFQGRPLADHPSVKNGRPPGALTIPRE
ILDASIVAEAISVVSCWYEEKTEWGTRVGWIYGSVTEDVVTGYRMHNRGWKSVYCVTQRDAFRGTAPINLTDRLHQVL
RWATGSVEIFFSRNNALFASSKMKVLQRIAYLNVGIYPFTSIFLIVYCFLPALSLFSGQFIVQTLNVTFLTYLLIITVTLCLLAM
LEIKWSGIALEEWWRNEQFWLIGGTSAHLAAVMQGLLKNSTK

>CSLD_5BS_TGACv1_425241_AA1392650.1_Triticum_aestivumMSRRLSLPAGSPVTVTVSPTKGKGAGGGSP
GDGVVRRGSGLTSPVPRHSIGSSTATLQVSPVRRSGGSRYASRDGADASAEFVHYTVHIPPTPDRTTASASTDVPAAEEE
GEVLPQRSYVSGTIFTGGLNCATRAHVLSNSADGARPAASANMSCKMRGCDMPAFLNAGRGGHPPCDCGFMICEEC
YMDCVAAAGNCPGCKEAYSAGSDTDDSVDEDDDDAISSSEERDQMPMTSMSKRFSMVHSIKMPMPSSNGKPADFD
HARWLFETKGTYGYGNALWPKNEHGGGGNNAGATSGFVGIEEPPNFGARCRRPLTRKTSVSQAILSPYRMLIAIRLVAL
GFFLAWRIRHPNPDAMWLWALSVTCEVWFAFSWLLDSLPKLCPVNRSCDLDVLADRFELPTARNPKGRSDLPGIDVF
VSTADPEKEPPLVTANTILSILAADYPVEKLACYLSDDGGALLTFEALAETASFARTWVPFCRKHGVEPRCPESYFGQKRD
FLKNKVRLDFVRERRKVKREYDEFKVRVNSLTEAIRRRSDAYNAGEELRARRRLQEEAVAAGGALGTAPLAETGAVKAT
WMSDGSQWPGTWLTGATDHARGNHAGIIQAMLAPPTSEPVLGGEPAESGALIDTTGVDIRLPMLVYVSREKRPGYD
HNKKAGAMNALVRTSAIMSNGPFILNLDCDHYVHNSAALREGMCYMLDRGGDRVCYVQFPQRFEGIDPNDRYANH
NLVFFDVAMRAMDGLQGPMYVGTGCIFRRTALYGFSPPRATEHHGWLGRKKIKLFLRKPTMGKKTDRESEHESMLPP

IEDDDHNQLGDGVRGDLLLPQQRTVRHPADEAPAARGLLQRGHVPVHLHVPHRLLRAPGRLPLHRQVHRPAPERHVP
RLPAHHHHHAVPAGAAGDQVVRDHAARVVAQRAVLGDRRHQRAPGCGAAGPPQGDRRRGHLLHAHVQAGRRRR
RGGGHVRGAVRGAVELPDGAPRDHHDAERGGAGGGDGEDAVQRVPAVEQAAGRRLLQLLGAVPPLPL

>CSLD_5DS_TGACv1_457675_AA1488780.1_Triticum_aestivumMSRRLSLPASSPVTVTVSPTRGKGAGGGSP
GDGVVRRGSGLTSPVPRHSIGSSTATLQVSPVRRSGGSRYASRDGADASAEFVHYTVHIPPTPDRNTASASTDAPVAEE
EGEVLPQRSYVSGTIFTGGLNCTTRAHVLSNSADGARPAASVNMSCKMRGCDMPAFLNAGRGGRPPCDCGFMICEE
CYMDCVAAAGNCPGCKEAYSAGSDTDDSVDEDDDDAISSSEERDQMPMTSMSKRFSMVHSIKMPMPSSNGNGGG
KPADFDHARWLFETKGTYGYGNALWPKNEHGGGGNTAGATSGFVGIEEPPNFGARCRRPLTRKTSVSQAILSPYRMLI
AIRLVALGFFLAWRIRHPNPDAMWLWALSVTCEVWFAFSWLLDSLPKLCPVNRSCDLDVLADRFELPTARNPKGRSDL
PGIDVFVSTADPEKEPPLVTANTILSILAADYPVEKLACYLSDDGGALLTFEALAETASFARTWVPFCRKHGVEPRCPESYF
GQKRDFLKNKVRLDFVRERRKVKREYDEFKVRVNSLTEAIRRRSDAYNAGEELRARRRLQEEAVAAGGALGAAPLAETG
AVKGTWMSDGSQWPGTWLTGATDHARGDHAGIIQAMLAPPTSEPVLGGVPAESGALIDTTGVDIRLPMLVYVSREK
RPGYDHNKKAGAMNALVRTSAIMSNGPFILNLDCDHYVHNSAALREGMCYMLDRGGDRVCYVQFPQRFEGIDPNDR
YANHNLVFFDVAMRAMDGLQGPMYVGTGCIFRRTALYGFSPPRATEHHGWLGRKKIKLFLRKPTTGKKTDRESEHES
MLPPIEDDDHNQLGDIESSALMPKRFGSSATFVSSIPVAEYQGRLLQDMPGVHQGRPAGALAVPREPLDAATVGEAIS
VISCFYEEKTEWGRRIGWIYGSVTEDVVTGYRMHNRGWRSVYCVTRRDAFRGTAPINLTDRLHQVLRWATGSVEIFFS
RNNALFATRRMKLLQRVAYFNVGMASSR

>CSLD_7BL_TGACv1_577301_AA1871610.1_Triticum_aestivumMASDHTNYTVFMPPTPDNQPGAAPAPASG
GSTKPDNLPLPRYTSGSKLVNRRSGDDGAAGGAKMDRGLSTEHVASPSKSLLVRSQTGEFDHNRWLFETQGTYGIGNA
YWPQDENDDGAGMGGGSVKMEDLVDKPWKPLSRKVAIPPGILSPYRLLVLVRFVALFLFLIWRATNPNPDAMWLW
GISIVCEYWFALSWLLDQMPKLNPINRAADLAALREKFESKTPSNPTGRSDLPGLDVFISTADPYKEPPLVTANTLLSILAT
DYPVEKLFVYISDDGGALLTFEAMAEACAYAKVWVPFCRKHSIEPRNPEAYFTQKGDPTKGKKRPDFVKDRRWIKREYD
EYKVRINDLPEAIKRRAKAMNAHERKIARETAAASSDAAPPPVKATWMADGTHWPGTWLDSAPDHGKGDHASIVQV
MIKNPHHDVVYGEADDHAYLDFTNVDVRIPMFVYLSREKRPGYDHNKKAGAMNAMVRASAVLSNGPFMLNFDCDH
YVYNCQAIREAMCYMLDRGGDRICYIQFPQRFEGIDPSDRYANHNTVFFDGNMRALDGLQGPMYVGTGCLFRRYAIY
GFNPPRAVEYHGLVGQTRVPIDPHARSGDGVADELRPLSDHPDHEAPQRFGKSKMFIESIAVAEYQGRPLADHPSVRN
GRPAGALLMPRPPLDAATVAEAVSVISCWYEDNTEWGLRVGWIYGSVTEDVVTGYRMHNRGWRSVYCITKRDAFRG
TAPINLTDRLHQVLRWATGSVEIFFSKNNAMLASRRLMFLQRMSYINVGIYPFTSLFLIMYCLLPALSLFSGQFIVATLDPT
FLCYLLLITVTLVLLCLLEVKWSGIGLEEWWRNEQFWVIGGTSAHLAAVLQGLLKVAAGIEISFTLTAKAAAEDDDDPFAE
LYLIKWTSLFIPPLAIIGINIIAMVVGVSRCVYAEIPQYSKLLGGGFFSFWVLAHYYPFAKGLMGRRGRTPTIVYVWAGLISI
TVSLLWITISPPDDRVSQSGIEV

>CSLD_7AL_TGACv1_559436_AA1799630.1_Triticum_aestivumMASDHTNYTVFMPPTPDNQPGAASAPASG
GPTKPDNLPLPRSSGSKLVNRRSGDDGAAGGGKMDRRLSPVQVASPSKSLLVRSQTGEFDHNRWLFETQGTYGIGNA
YWPQEDNDDGAGMGGGSVKMEDLVDKPWKPLSRKVAIPPGILSPYRLLVLVRFVALFLFLVWRATNPNPDAMWLW
GISIVCEYWFALSWLLDQMPKLNPINRAADLAALREKFESKTPSNPTGRSDLPGLDVFISTADPYKEPPLVTANTLLSILAT
DYPVEKLFVYISDDGGALLTFEAMAEACAYAKVWVPFCRKHSIEPRNPEAYFTQKGDPTKGKKRPDFVKDRRWIKREYD
EYKVRINDLPEAIKRRAKAMNAHERKIARETAAASSDAAPPPVKATWMADGTHWPGTWLDSAPDHGKGDHASIVQV
MIKNPHHDVVYGDADDHAYLDFTNVDVRIPMFVYLSREKRPGYDHNKKAGAMNAMVRASAVLSNGPFMLNFDCDH
YVYNCQAIREAMCYMLDRGGDRICYIQFPQRFEGIDPSDRYANHNTVFFDGNMRALDGLQGPMYVGTGCLFRRYAIY
GFNPPRAVEYHGLVGQTRVPIDPNARSGDGVADELRPLSDHPDHEAPQRFGKSKMFIESIAVAEYQGRPLADHPSVRN

GRPPGALLMPRPPLDAATVAEAVSVISCWYEDNTEWGLRVGWIYGSVTEDVVTGYRMHNRGWRSVYCITKRDAFRG
TAPINLTDRLHQVLRWATGSVEIFFSKNNALLASRRLMFLQRMSYINVGIYPFTSLFLIMYCLLPALSLFSGQFIVATLDPTF
LCYLLLITITLVLLCLLEVKWSGIGLEEWWRNEQFWVIGGTSAHLAAVLQGLLKVAAGIEISFTLTAKAAAEDDDDPFAEL
YLIKWTSLFIPPLAIIGINIIAMVVGVSRCVYAEIPQYSKLLGGGFFSFWVLAHYYPFAKGLMGRRGRTPTIVYVWAGLISIT
VSLLWITISPPDDRVSQSGIEV

>CSLD_7DL_TGACv1_603510_AA1985050.1_Triticum_aestivumMASDHTNYTVFMPPTPDNQPGAAPTPASG
GSTKPENLPLPRYTSGSKLVNRRSGDDGAAGGAKMDRWLSTEQVASPSKSLLVRSQTGEFDHNRWLFETQGTYGIGN
AYWPQDDNDDGAGMGGGSVKMEDLVDKPWKPLSRKVAIPPGILSPYRLLVLVRFVALFLFLVWRATNPNPDAMWL
WGISIVCEYWFALSWLLDQMPKLNPINRAADLAALREKFESKTPSNPTGRSDLPGLDVFISTADPYKEPPLVTANTLLSIL
ATDYPVEKLFVYISDDGGALLTFEAMAEACAYAKVWVPFCRKHSIEPRNPEAYFTQKGDPTKGKKRPDFVKDRRWIKRE
YDEYKVRINDLPEAIKRRAKAMNAHERKIARETAAASSDAAPPPVKATWMADGTHWPGTWLDSAPDHGKGDHASII
QVMIKNPHHDVVYGDADDHAYLDFTNVDVRIPMFVYLSREKRPGYDHNKKAGAMNAMVRASAVLSNGPFMLNFDC
DHYVYNCQAIREAMCYMLDRGGDRICYIQFPQRFEGIDPSDRYANHNTVFFDGNMRALDGLQGPMYVGTGCLFRRY
AIYGFNPPRAVEYHGLVGQTRVPIDPHARSGDGIADELRPLSDHPDHEAPQRFGKSKMFIESIAVAEYQGRPLADHPSV
RNGRPAGALLMPRPPLDAATVAEAVSVISCWYEDNTEWGLRVGWIYGSVTEDVVTGYRMQNRGWRSVYCITKRDAF
RGTAPINLTDRLHQVLRWATGSVEIFFSKNNAMLASRRLMFLQRMSYINVGIYPFTSLFLIMYCLLPALSLFSGQFIVATL
DPTFLCYLLLITVTLVLLCLLEVKWSGIGLEEWWRNEQFWVIGGTSAHLAAVLQGLLKVAAGIEISFTLTAKAAAEDDDDP
FAELYLIKWTSLFIPPLAIIGINIIAMVVGVSRCVYAEIPQYSKLLGGGFFSFWVLAHYYPFAKGLMGRRGRTPTIVYVWAG
LISITVSLLWITISPPDDRVSQSGIEV

>CSLE_6DL_TGACv1_526558_AA1687090.1_Triticum_aestivumMAGSSVSGGGGGRPPLFATEKPKRVLAYRLY
AGTIFAGILLIWFYRATHIPARGSSSLGWRAGLGLLVAELWFGLYWVLTLSVRWNPVRRATFKDRLSERYDDDQLPGVD
IFVCTADPALEPPMLVISTVLSVMAYDYPPEKLNIYLSDDAGSAVTFYALHEASEFAKHWIPFCKNYKVEPRSPAAYFAKG
ATPHDACSPQEFLRMKELYKDLTDRMNSVVHSGKIPEVPECNHRGFSEWNETITSGDHPSVVQILIDRNKRKAVDVDG
NALPKLVYMAREKRPQEQHHFKAGSLNALIRVSSVISNSPVILNVDCDMYSNNSESIRDALCFFLDEEQGQDIGFVQYP
QNFDNVVHNDSYGNPINVVNELDNPCLDGWGGMCYYGTGCFHRRETLSGQIYSKDYKEDWARGVGIAENADELEET
SKSLVTCTYEHNTPWGIEKGVRYGCPLEDVITGLQIQCHGWRSVYYNPARKGFLGKAPTSLGQILVQHKRWSEGFLQM
SLSNYSPFLLGHGKIKLGLQMGYSVCGFWALNSFPTFYYVIIPSLCFLSGVSVFPEITSPWCIPFIYVVVASYSWSLMESLQ
CGDTAVEWWNAQRMWLMRRTTSYLLAAIDTIGGMLGVSESGFELTVKVDESQALERYKKGKMEFGPISGMFVIITTIA
LFNLVCLVLGLGRVLLRGGAEGLGPLFLQAVLCVAIVVINAPVYEALFIRRDSGGLPYFVTLVSLCFVSSLCLQAI

>CSLE_6AL_TGACv1_471004_AA1500600.1_Triticum_aestivumMAGSSVSGGGGRPPLFATEKPKRVLAYRVYA
GTIFAGILLIWFYRATHIPARGSSSLGWRAGLGLLVAEILFGLYWVLTLSVRWNPVRRTTFKDRLSERYDDDQLPGVDIFV
CTADPALEPPMLVISTVLSVMAYDYPPEKLNIYLSDDAGSAVTFYALHEASEFAKHWIPFCKNYKVEPRSPAAYFAEGAT
PHDACSPQELLRMKELYKDLTDRVNSVVHSGKIPEVPECNHRGSSEWNEMITSGDHPSIVQILIDRNKRKAVDVDGNA
LPKLVYMSREKRPQEQHHFKAGSLNALIRVSSVISNSSVILNVDCDMYSNNSESIRDALCFFLDEEQGQDIGFVQYPQNF
DNVVHNDIYGNPINVVNELDNPCLDGWGGMCYYGTGCFHRRETLSGQIYSKDYKEDWDRGVGIAENADELEETSKSL
VTCTYEHNTPWGIEKGVRYGCPLEDVITGLQIQCRGWRSVYYNPARKGFLGMAPTSLGQILVQHKRWSEGFLQISLSNY
SPFLLGHGKIKLGLQMGYSVCGFWALNSFPTFYYVIIPSLCFLSGVSVFPEITSPWCIPFIYVVVASYSWSLMESLQCGDTA
VEWWNAQRMWLMRRTTSYLLAAIDTIGGMLGVSESGFELTVKVDESQALERYKKGKMEFGPISGMFVIITTIALFNLV
CLVVGLGRALLREGTAGLGPLFLQAVLCVAIVVINAPVYEALFIRRDSGSLPYFVTLVSLCFVSSLCLQAI

>CSLE_6BL_TGACv1_499967_AA1596110.2_Triticum_aestivumMAGSSVSGGGRPPLFATEKPKRVLAYRLYAG
TIFAGILLIWFYRATHIPERGDSSLGWRAGLGLLVAELLFGLYWVLTLSVRWNPVRRTTFKDRLSERYDDDQLPGVDIFVC
TADPALEPPMLVISTVLSVMAYDYPPEKLNIYLSDDAGSAVTFYALHEASEFAKHWIPFCKNYKVEPMSPAAYFAEGATP
HDACSPQELLRMKELYKDLTDRVNSVVHSGKIPEVPECNHRGFSVWNETITSGDHPSIVQILIDRNKRKAVDVDGNALP
KLVYMAREKRPQEQHHFKAGSLNALIRVSSVISSSPVILNVDCDMYSNNSESIRDALCFFLDEEQGQDIGFVQYPQNFD
NVVHNDIYGNPINVVNELDNTCLDGWGGMCYYGTGCFHRRETLSGQIYSKDYKEDWARGVGIAENADELEETSKSLV
TCTYEHNTPWGIEKGVRYGCPLEDVITGLQIQCRGWRSVYYNPARKGFLGMAPTSLGQILVQHKRWSEGFLQISLSNYS
PFLLGHGKIKLGLQMGYSVCGFWALNSFPTFYYVIIPSLCFLSGVSVFPEITSPWCIPFIYVVAASYSWSLMESLQCGDTA
VEWWNAQRMWLMRRTTSYLLAAIDTIGGMLGVSESGFELTVKVDESQALERYKKGKMEFGPISGMFVIITTIALFNLV
CLVLGLGRVVLREGAAGLGPLFLQAVLCVAIVVINAPVYEALFIRRDSGSLPYFVTLVSLCFVSSLCLQAI

>CSLE_U_TGACv1_683314_AA2158770.1
peptideMQIRVSSVISNSPIIMNVDCDMYSNNNDAVRDALCFFLDEEMGHKIGFVQYPQNYNNLSKNDIYGNSLHVIN
EVEMGGMDSLGGPLYIGTGCFHRREILCGRKFTKDYQEDWNAGIKDKLQESIDETEEKAKSLAACTYEHGTQWGDEIG
VKYGCAVEDVITGLAIHCRGWESVYNNPKKPAFMGVGPTTLAQTILQHKRWSEGNLSIFLSKYNVFLFGHGKTKLRHQ
MGYHIYGLWAPNSLATLYYVIIPSLALLKGTPLFPEITSPWIAPFVYVFCVKNMYSLYEALSSGDTLKGWWNGQRMWLV
KRITSYLFGVLDNLRKLLGLSKMNFVVSPKVSDEDESKRYEQEIMEFGSSDPEYVIIGTITLLNLVCLLGGLSKVMKVGWN
NIHLDALFPQLILCGMVVITSIPFYEAMFLRKDKGRIPFPVTLASIGFVMLALLPAIV

>CSLE_5DL_TGACv1_433536_AA1415840.1_Triticum_aestivumMVAIGRRTGQQHGHWRLAAESPPYLGPRD
GEEHEAVRDGDSRGPGGVQAPRRHGGRRILLLLYYRATRVPAAGEGRAAWLGMLAAELWYAAYWVVTQSVRWSPV
RRRPFIDRLAARHGETLPCVDIFVCTADPYSEPPSLVVSTILSLMAYNYPPEKLSVYLSDDGGSILTFYGMWEASLFAKHW
LPFCKRYNIEPRSPAAYFSESDGHQELCNPKEWSLIKDMFDKMTERIDTVVMSGKVPEEIKASHKGFYEWNQEITSKNH
QPIVQILIDSKDQNAVDNEGKVLPTLVYMAREKRPQHHHNFKAGAMNALIRVSSVISNSPIIMNVDCDMYSNNNDAV
RDALCFFLDEEMGHKIGFVQYPQNYYNLSKNNIYGNSLHVINEVEMGGMDSLGGPMYIGTGCFHRREILCGRKFTEDY
QEDWNAGIKDKLQESIDETEEKAKSLAACTYEHGTQWADEIGVKYGCVVEDVNTGLAIHCRGWDSVYNNPKRPAFM
GVGPTTLAQTILQHKRWSEGIFSIFLSKYNVFLFAHGKTKLRHQMGYHIYGLWAPNSLATLYYVIIPSLALLKGTSLFPEITS
PWIAPFVYVFCVKNMYSLYEALLSGDTLKGWWNGQRMWLVKRITSYLFGVLDNLRKLLGLSKINFVVTPKVSDEDESK
RYEQEIMEFGSSDPEYVIIAAIALLNLVCLMGGLSKVMKGGWNVHLDALFPQLILCGMLVITSIPFYEAMFLRKDKGRIPF
PVTLASIGFVMLALLAKIV

>CSLE_5BL_TGACv1_406235_AA1342610.1_Triticum_aestivumMERSRRLFETETHGGRAAYRLHAVTVAAGIL
LVLYYRATHVPAAGEGRATWLGMLAAELWYAAYWVVTQSVRWSPVRRRPFIDRLAARHGERLPCVDIFVCTADPYSE
PPSLVVSTILSLMAYNYPPEKLSVYLSDDGGSILTLYGMWEASLFAKHWLPFCKRYNIEPRSPAAYFSESDGHQELCTPKE
WSLIKDMFDKMTERIDTAVMSGKVPEEIKARHKGFYEWNQEISSKNHQPIVQILIDGKDQNAVDNEGKVLPTLVYMA
REKRPQHHHNFKAGAMNALIRVSSVISNSPIIMNVDCDMYSNNNDAVRDALCFFLDEEMGHKIGFVQYPQNYNNLSK
NNIYGNSLHVINEVEMGGMDSLGGPMYIGTGCFHRREILCGRKFTEDYQEDWNAGIKDKLQESIDETEEKAKSLAACTY
EHGTQWGDEIGVKYGCVVEDVNTGFAIHCRGWESVYNNPKKPAFMGVGPTTLAQTILQHKRWSEGIFSIFLSKYNVFL
FAHGKTKLQHQMGYHIYGLWAPNSLATLYYVIIPSLALLKGTSLFPEITSPWIAPFVYVFCVKNMYSLYEALSSGDTLKGW
WNGQRMWLVKRITSYLFGVLDNLRKLLGLSKMNFVVTPKVSDEDESKRYEQEIMEFGSSDPEYVIIGTITLLNLVCLLGG
LSKVMKGGWNEHLDALFPQLILCGMVVITSIPFYEAMFLRKDKGRIPFPVTLASIGFVMLALLATIV

>CSLE_5AL_TGACv1_376126_AA1232370.2_Triticum_aestivumMERTRLFETETHGGRAAYRLHAVTVAAGILL
LLYYRATRVPAAGEGRAAWLGMLAAELWYAAYWVVTQSVRWSPVRRRPFRDRLAARHGERLPSVDIFVCTADPYSEP
PSLVVSTILSLMAYNYPPEKLSVYLSDDGGSILTYYGMWEASLFAKHWLPFCKRYNIEPRSPAAYFSQSDGHQELCTPKE
WSLIKDMFDEMTERIDTAVMSGKVPEEIKARQKGFHEWNQEITSKNHQPIVQILIDGKDQNAVDNEGNVLPTLVYMA
REKRPQHHHNFKAGAMNALIRVSSVISNSPIIMNVDCDMYSNNNDAVRDALCFFLDEEMGHKIGFVQYPQNYNNLSK
NDIYGNSLQVINEVEMAGMDSLGGPLYIGTGCFHRREILCGRKFTKDYQEDWNAGIKDKLQESIDETEEKAKSLAACTYE
HGTQWGDEIGVKYGCAVEDVITGLAIHCRGWESVYNNPKKPAFMGVGPTTLAQTILQHKRWSEGNLSIFLSKYNVFLF
GHGKTKLRHQMGYHIYGLWAPNSLATLYYVIIPSLALLKGTPLFPEITSPWIAPFVYVFCVKNMYSLYEALSSGDTLKGW
WNGQRMWLVKRITSYLFGVLDNLWKLLGLSKMNFVVSPKVSDEDESKRYEQEIMEFGSSDPEYVIIGTITLLNLVCLLG
GLSKVMKVGWNNIHLDALFPQLILCGMVVITSIPFYEAMFLRKDKGRIPFPVTLASIGFVMLALLPAIV

>CSLE_5DL_TGACv1_433536_AA1415830.1_Triticum_aestivumMERTRRLFETETHGGRAAYRLHAVTVAAGIL
LLLYYRATHVPAAGEGRAAWLGMLAAELWYAAYWAVTQSVRWSPVRRLPFIDRLAARYGERLPCVDIFVCTADPHSE
PPSLVISTVLSLMAYNYPAEKISVYLSDDGGSVLTFYALWEASLFAKHWIPFCKRYNIEPRSPAAYFSESDGHQDLCSPKE
WSLIREMYEDMTERIDTAVLSGKISEEVKANHKGFHEWDQENTSKNHQPIVQILIEGKDKNANDDEGNVLPTLVYMAR
EKRPQHHHNFKAGAMNALIRVSSVISNSPIIMNVDCDMYSNNCDTIRDALCFFLDEEMGHKIGFVQFPQNYNNLTKN
NIYGNSHQVTNQVLMGGMDSVGGPMYVGTGCFHRREILCGRRFTEDYKEDWNGGIKDKTQESIVEIEEKAKSLAASTY
EHDTQWGDEIGIKYGYPAEDIVTGLGIHCRGWKSVHSNPPRPAFLGVAPTTLAQTLLQHKRWSEGSFSIFLSKYCPFMF
GHGKIKLRHQMGYSIYGLWAPNSIPTLYYVIIPSLALLKGISLFPEITSPWMSPFIYVLCVKNMYSLYEALSCGDTLKGWW
NEQRMWMVRRITSYLYGLTDTVRKLLGLSKMTFAVTSKVSEESESKRYEQEIMEFGSSAPEYVIIATVALLNLNCLVVGL
CQIMTGGWNILLNVFSPQLILCGMLVITNIPFYEAMFVRKDKGRIPFSVTLASIGFAILALLVPIV

>CSLE_5BL_TGACv1_406235_AA1342600.1_Triticum_aestivumMERSRRLFETETHGGRAVYRLHAVTVAAGIL
LLLYYRATRVPAAGEGRAAWLGMLAAELCYAAYWVVTQSVRWSPLHRRPCRDRLAARYGERLPCVDIFVCTADPHSE
PPSLVISTVLSLMAYNYPAEKISVYLSDDGGSILTFYALWEASLFAKHWIPFCKRYNIEPRSPATYFSESDGHQDMCTPKE
WSLIREMYEDMTERIDTAALSGKISEEVKENHKGFHEWDQENTSKNHQPIVQILIEGKDKNANDDEGNVLPTLVYMAR
EKRPQHHHNFKAGAMNALIRVSSVISNSPIIMNVDCDMYSNNYDTIRDALCFFLDEEMGHKIGFVQFPQNYNNLTKN
NIYGNSHQVTNQVLMGGMDSVGGPMYVGTGCFHRREILCGRRFTKDYKEDWDGGIKDKTQESIDEIEEKAKSLAAST
YEHDTQWGDEIGIKYGYPAEDIVTGLEIHCRGWKSVHSNPPRPAFLGVAPTTLAQTLLQHKRWSEGSFSIFLSKYCPFMF
GHGKIKLRHQMGYSIYGLWAPNSIPTLYYVIIPSLALLKGISLFPEITSPWISPFIYVVCVKNMYSLYEALSCGDTLKGWWN
EQRMWMVRRITSYLYGLTDTVRKLLGLSKMTFAVTSKVSEENESKRYEQEIMEFGSSAPEYVIIATVALLNLICLVGGLSQ
ILTGGWNILLNVFSPQLILCGMLVITNIPFYEAMFVRKDKGRMPFSVTLASISFAMLALLVPIVYNSRYI

>CSLE_6DS_TGACv1_543277_AA1737920.1_Triticum_aestivumMERRLFETVRHGGRALYRLHAVTVAASTLLV
LYYRATRVPGSGGRRAAWLGMLAAELWYAAYWVVTQSVRWSPVRRCTFRDRLTARYGDRLPGVDIFVCTADPLSEPP
SLVISTILSVMAYNYLAEKLSVYLSDDGGSVLTFYAMWEASLFAKHWLPFCKRYNIEPRSPAAYFSESYQDLCTPKEWSFI
KDMYDEMTERIDTAVISRKIPEEIRSNHKGFYEWNPEITSKNHQPIVQVLIDGKDQKGVDSEGNVLPTLVYMAREKRPQ
HHHNFKAGAMNALIRVSSVISNSPVIMNVDCDMYSNNNDTIRDALCFFLDEEMGHKIGFVQYPQNYNNMTKNNLYG
NSLHVINEVEMGGMDSLGGPLYVGTGCFHRREILRGKRFTKDYQEDWNGGIKGRIQDTSIGEIEEKAKSLATCTYEHDT
QWGDEIGLKYGCPVEDVITGLAIHCRGWESVYSNPTRPAFMGLGPTTLAQTLLQHKRWSEGSFSIFLSKYCPFLFGHGK
TNLRHQMGYCIYGLWAPNSLPTLYYVVIPSLALLKGIPLFPKITSPWIAPFIYAFCVKNMYSLYEALSCGDTLKGWWNGQ
RMWLVKRITSFLFGAIDTVRKLLGLSKMTFVVTPKVSIEDESKRYEEEIMEFGSSTPEYVIIATIALLNLVCLLGGLSQIMTG
AWNIHLDAFSPQLILCGMLVITNMPFYEAMFLRNDKGKIPFTVTLASFGFVMLDFLVPIV

>CSLF_2DL_TGACv1_159781_AA0542640.1_Triticum_aestivumMAAAVTRRVNALRVEVPDGNADTANAPAA
KRILDAKDDVWVSADDGTSAGNGNQPLLFRTMKVKGSILHPYRFLILVRLVAVAAFFAWRLEHRNHDGTWLWATSM
VADAWFGFSWLLNQLTKLNPIKRVPDLATLADQHGEAILPGIDVFVTTADPVDEPVLYTVNTVLSILAADYPIDKYACYLS
DDGGTLVHYEAMTQVASFAALWAPFCRKHCVEPRSPENYFGMKAQPYAGSMPGDFTRDRRRVRREYDEFMVRIDSL
STTIRQRSDAYNNGDGVHATRMADGAPWPGTWIEQAENHRRAQHAGIVQVILEHPGCKPQLGSSASTDNPFDFNN
VDMRLPMLVYISREKRLGYDNQKKAGAMNAMLRISALLSNAPFIINFDCDHYINNSKALRAPMCFMLDPRDGQNTAF
VQFPQRFDDVDPTDRYANHNRVFFDGTMLALNGLQGPSYLGTGTMFRRVTLYGMEPPRYRVENIKLVDNAHEFGNS
TSFTNSMPDGAIQERSITPVLVDEGLINDLATLITCAYEDGSSWGRDIGWVYNIATEDVVTGFRIHRQGWRSMYCSME
PAAFRGTAPINLTERLYQVLRWSGGSLEVFFSHNNALIAGRRLHPLQRIAYFNMSTYPIVTVFILAYNFFPVMWLFSEQLY
IQRPFGTYIAYLVAVIAMMHVIGMFEVKWAGITLLDWCRNEQFYLIAATGVYPTAVLYMALKLVTGKGMHFRLTSKQT
EACSRDKFANLYTVRWVPLLIPTTAVLVVNVAAVGAAIGKAAAWGFSTDQARHVLLGMVFNVGTLMLLYPFALGIMG
KRGKTPVILFVLLLMAIAAVGLLYVTLYAPYPQESLTFLSW

>CSLF_7BL_TGACv1_580651_AA1914920.1_Triticum_aestivumMATDTVADAAEGRRARDDVWVAAEEGDM
PEASAGRPLLFRTMKVKGSILHPYRFLILVRLVAIVAFFAWRVEHRNHDGVWLWATSMVADAWFGFSWLLNQLPKLN
PIKRVPDLAALADRHGEAVLPGIDVFVTTVDPVDEPVMYTVNTILSILAADYPVDKYACYLSDDGGTLVHYEAMLQVAS
FAALWVPFCRKHCVEPRSPENYFGMKTRPYVGGMAGEFMSDHRRVRREYGEFKVRIDSLSSTIRRRSDAYNKGDDDV
HATWMADGTQWPGTWIEQADNHRRGQHAGIVKVMLDHPSCKPQLGSSASTNKPVDLSNVDTRLPMLVYISREKRP
GYDNQKKAGAMNVMLRVSALLSNAPFVINFDCDHYINNSQALRAPMCFMLDPRDGQNTAFVQFPQRFDDVDPTDR
YSNHNRVFFDGTMLSLNGLQGPTYLGTGTMFHRVALYGMEPQRYRAENIKLVGKGAELGKSTPFLNSIPDGAIQDRSIT
PVSVDEGLMSDLATLMTCAYEDRTSWGRDVGWVYNIATEDVVTGFRIHRQGWRSMYCSMEPAAFRGTAPINLTERL
YQVLGGQAAPSRCSSPTATLSSPAVGSTLYSASLTSTCRSTRSPRCSS

>CSLF_7AL_TGACv1_557532_AA1782680.1_Triticum_aestivumMPLRVEALVATDTASAAAAEGRRAKDDVW
VAEEGDMSGASAGRPLLFRTMKVKGSILHPYRFLILVRLVAIVAFFAWRVEHRNHDGTWLWATSMVADAWFGFSWL
LNQLPKLNPIKRVPDLVALADRHGEAILPGIDVFVTTVDPVDEPVLYTVNTILSILAADYPVDKYACYLSDDGGTLVHYEA
MLQVASFAALWVPFCRKHCVEPRSPENYFGMKTRPYVGGMAGEFMSDHRRVRREYGEFKVRIDSLSTTIRRRSDAYN
KGDDGVHATWMADGTQWAGTWIEQADNHRRGHHAGIVQVMLDHPSCKPQLGSSVSTNSPIDLSNVDTRLPMLVYI
SREKHPGYDNQKKAGAMNVMLRVSALLSNAPFVINFDCDHYINNSRALRAPMCFMLDPRDGQNTAFVQFPQRFDN
VDPTDRYSNHNRVFFDGTMLSLNGLQGPTYLGTGTMFRRVALYGMEPPRYKAENIKLVGKAAELGNSTPFLKSIPDGAI
QERSITPVLVDEALTSDLATLMTCAYEDRTSWGRDVGWVYNIATEDVVTGFRIHRQGWRSMYCSMEPAAFRGTAPIN
LTERLYQVLRWSGGSLEAFFSHSNALIASRRLHPLQRIAYLNMSIYPIATMFILAYSFFPVMWLFSEESYYIQRPFGTFIMY
LVAVIAMMHVIGMFEVKWAGITLQDWWRNEQFYMITATGVYPTAVLYMALKLIRGKGIYFRLTSKQTEACSGEKFAD
LYTVRWVPLLIPTVAVLVVNIAAIGAAIGKAATWGFFTDQAWHAVLGMVFNVGTLVLLYPFALGIMGQWGKRPGILLV
MLVMAIGTVGLLYVTLQQDGHRMSFLTRPSG

>CSLF_7DL_TGACv1_602590_AA1961740.1_Triticum_aestivumMALRVEALVATDTAAAEGRRAKDDVWVAA
EEGDMSGASAGRPLLFRTMKVKGSILHPYRFLILVRLVAIIAFFAWRVEHRNHDGMWLWATSMVADAWFGFSWLLN
QLPKLNPIKRVPDLAALADLHGEAVLPGIDVFVTTVDPVDEPVMYTVNTILSILAADYPVDKYACYLSDDGGSLVHYEAM
IQIVHFAALWVPFCRKHCIEPRSPENYFGMKTRPYVGGMAGEFMSDHRRVRREYGEFKVIIDSLSTTIRRRSDAYNKRD
DGVHATWMADGTQWAGTWIEQADNHRRGQHAGIVQVMLDHPSCKPQLGSSARTNNPIDLSNVDTRLPMLVYISR
EKHPGYDNQKKAGAMNVMLRVSALLSNAPFVINFDCDHYINNSQALRAPMCFMLDPRDGQNTAFVQFPQRFDDVD
PTDRYSNHNRVFFDGTMLSLNGLQGPTYLGTGTMFRRVALYGMEPPCYRAENIKLVGKAAELGNSTPFLNSIPDGAIQE

RSITPVLVDEGLSNDIATLMTCTYEDGSSWGRDVGWVYNIATEDVVTGFRIHRQGWRSMYCSMEPAAFRGTAPINLT
ERLYQVLRWSGGSLEVFFSHSNALIASRRLNPLQRIAYLNMSIYPIATMFILAYSFFPVMWLFSEQSYYIQRPFGTFIMYLV
VVIAMMHVIGMFEVKWAGITLQDWWRNEQFYMIAATGVYPTAVLYMALKLIRGKGIYFRLTSKQTEACSDEKFADLY
TVRWVPLLIPTVAVLIVNVTAVGAAIGKAATWGFFTDQAWHAVLGMVFNVGTLVLLYPFALGIMGQWGKRPGILLV
MLVMAIGTVGLLYVTLQQDGHRMSFLTRPSG

>CSLF_2AL_TGACv1_094713_AA0301960.1_Triticum_aestivumMMAAAVTRRSNALRVDVPDGDAVAVSVVA
DSPVAKRGLGAKEDVWVAVDEGGMSGDGNRPLLFRTMKVKGSILHPYRFLMLMRLVAVVIFFKWRMEHKNHDGV
WLWTVSMTADVWFGFSWLLNQLPKLNPIKRVPDLAALADRHDDATLPGIDVFVTTVDPVDEPVLYTVNTILSILAADY
PVDNYACYLSDDGGTLVHYEAMVQVASFAALWVPFCRKHCVEPRSPESYFGIKTHSYAGGMAGEFMRDRRRVRREYE
EFKVRIDSLSTTIRQRSDAYNSKNKGGVSATWMADGTQWPGTWVEQAENHRRGQHAGIVQVLLDHPSCEPQLGSPA
STDNPFDFSNVDTRLPMLVYISREKRPGYDNQKKAGAMNVMLRVSALLSNAPFVINFDCDHYINNSQALRAPMCFML
DPRDGQNTAFVQFPQRFDDVDPTDRYANHNRVFFDGTMLALNGLQGPTYLGTGTMFRRVSLYGIEPPRYRAENTKLV
RKAGEFGYSTSFVNSVPDAAIQDRSITPVLVDEGLRKDLTTLMTCAYEDGSSWGRDAGWVYNIATEDVVTGFRIHRQG
WRSMYCSMEPAAFRGTAPINLTERLYQVLRWSGGSLEVFFSHSNALIASRRLHLLQRIAYLNMSTYPIVTVFILSYNFFPV
MWLFSEQLYIQRPFGTYMAYLVAIIAMVHLIGMFEVRWSGITLLDWFRNEQFYMIGATGVYPTAVLYMLLKLVTGKGI
YFRLTSKQTEACSNDKFADLYTVRWVPLLIPTTAVIIVNVAAVGAAIGKAATWGFFTDEARHALLGMVFNMGILVLLYPF
ALGIMGKWGKRPIILFIVLVMAISAVGLLYVMLHAPYTGEWSQVAVSLGKASLTGPSGSG

>CSLF_2DL_TGACv1_160109_AA0546890.1_Triticum_aestivumMAAAVTRRSNALRVDVPGGEAVAVSVAAD
SPVAKRGLGAKDDVWVAADEGGIMSGDGNRPLLFRTMKVKGSILHPYRFLMLVRLVAVVAFFKWHVEHKNQDSVW
LWTASMTADPWFGFSWLLNQLPKLNPIKRVPDLADRHDDATLPRIDVFVTTVDPVDEPVLYTVNTILSILAADYPIDNY
ACYISDDGGTLVHYEAMVQVASFAALWVPFCRKHCVEPRSPESYFGIKTRSYIGGMAGEFMRDHRRVRREYEEFKVRI
DSLSTTIRQRSDAYNSSNKGGVSATWMADGTHWPGTWVEQAENHRRGQHAGIVQVLLDHPSCKPQLGSPASTDNP
FDFSNIDTRLPMLVYISREKRPGYDNQKKAGAMNVMLRVSVLLSNAPFVINFDCDHYINNSQALRAPMCFMLDPHDG
QNTAFVQFPQRFDDVDPTDRYANHNRVFFDGTMLALNGLQGPTYLGTGTMFRRVSLYGIEPPRYRAENTKLVRKTGE
FGYSTSFVNSVPDAAIQDRSITPVLVDEHLRKDLATLMTCAYEDGSSWGRDAGWVYNIATEDVVTGFRIHRQGWRSM
YCSMEPAAFRGTAPINLTERLYQVLRWSGGSLEVFFSHSNALIASRRLHPLQRIAYLNMSTHPIVTVFILSYNFFPVMWLF
SEQLYIQRPFGMYMGYLVAIIAMVHLIGMFEVRWSGITLLDWFRNEQFYMIGATGVYPTAVLYMLLKLATGKGIYFRLT
SKQTEACSNDKFADLYTVRWVPLLIPTTAVIIVNVAAVGAAIGKAATWGFFTDEARHALLGMVFNMGILVLLYPFALGI
MGKWAKRPIILFIVLVMAISVVGLLYVSLHAPYTGEWSQVAVSLGKASLTGPSGSG

>CSLF_2BL_TGACv1_130934_AA0420130.1_Triticum_aestivumMVQVASFAALWVLFCRKHCVEPRSPESYFG
MKTRSYAGGMAGEFMRDHRRVRREYEEFKVRIDSLSTTIRQRSDAYNSNNKGGVSATWMADGTQWPGTWVEQAE
NHRRGQHAGIVQVLLDHPSFKPQLGSPASTDNPFDFSNVDTRLPMLVYISREKRPGYDNQKKAGAMNVMLRVSALLS
NAPFVINFDCDHYINNSQALRAPMCFMLDPHDGQNTAFVQFPQRFDDVDPTDRYANHNRVFFDGTMLALNGLQGP
TYLGTGTMFRRVALYGIEPPHYRAENTKLVCKTGEFGYSTSFINSVPDAAIQDRSITPVLVDERLSKDLATLMTCAYEDGS
SWGRDAGWVYNIATEDVVTGFRIHRQGWHSMYCSMEPAAFRGTAPINLTERLYQVLRWSGGSLEVFFSHNNALIASR
RLHPLQRITYLNMSTYPIVTVFILSYNFFPVMWLFSEQLYIQRPFGTYMAYLVGIIAMVHLIGMFEVRWSGITLLDWFRN
EQFYMIGATGVYPTAVLYMLLKLVTGKGIYFRLTSKQTEGCSNDKFADLYTVRWVPLLIPTAAVIIVNVAAIGAAIGKAAT
WGFFTDEARHALLGMVFNMGILVLLYPFALGIMGKWGKRPIILFIVLVMAISVVGLLYVTLHAPYTGEWSQVAVSLGKA
SLTGPSGSG

>CSLF_2DS_TGACv1_178985_AA0603230.1_Triticum_aestivumMTTSPATDAGAATGLSEPLLSNRNGVHAGA
LVVTPVAANGHGGKAKDKYWKDVDQPGDMAVAPDLENGGGRPLLFSNRRVKNIILYPYRVLILIRVIAVILFVGWRIKN
NNSDVMWFWVISVVADVWFSLSWLSYQLPKYNPIKMIPDLATLRKQFDTPGRSSQLPGIDVIVTTASATDEPILYTMN
CVLSILAADYHIGRCNCYLSDDSGSLVLYEALVETAKFAALWVPFCRKHQIDPRAPESYFELEGPLCGGASHKEFIQDYKH
VCTQYEEFKKHLDMLPNTIRQRADIYSKTGTKDEDAKVTWMADGTQWPGTWLDPAEKHRAGHHAGIVKIVQSHPEH
VVPLGVHESNDSSLNFDGVDMRLPMLVYVAREKCPGVEHNKKAGALNAELRISALLSNAPFFINFDCDHYINNSEALHA
AVCFMLDPREGDNTGFVQFPQRFDNVDPTDRYGNHNRVFFDGAMYGLNGQQGPTYLGTGCMFRRLALYGIDPPCW
RAEDIIVDSNRFGNSLPFLNSVLAAIKQEEGVTLPPTLDDSFLEEMTKVVSSSYDDSTDWGRGIGYIYNMATEDIVTGFRI
HGQGWRSMYATMEREAFRGTAPINLTERLRQIVRWSGGSLEMFFSHISPLFAGRRLSLVQRLSYINFTIYPLTSLFILMYA
FCPVMWLLPTEILVQRPYTRYIVYLIIVIVMIHVIGMFEIMWAGITWLDWWRNEQFFMIGSVTAYPTAVLHMVVNILTK
KGIHFRVTTKQPVADTDDKYAEMYEVHWVPMMIPAVVVLFSNILAIGVAIGKSILYMGTWSAAQKRHGALGLLFNLWI
MVLLYPFALAIIGRWAKRTGILFILLPIAFLSTSLMYIGVHTFLLHFFPSMLI

>CSLF_2AS_TGACv1_112790_AA0345230.1_Triticum_aestivumMTTSPATHDGAATGLSEPLLPNRNGVHAGA
LVVTPVVANGHGGGDKLKGDLKAKDKYWKDVDQPDDVAAAPDLENGGGRPLLFSNRRVKNIILYPYRVLILIRVIAVILF
VGWRIKHNNSDVMWFWVMSVVADVWFSLSWLSYQLPKYNPIKMIPDLATLRKQFDTPGRSSQLPGIDVIVTTASAT
DEPILYTMNGVLSILAADYHIGRCNCYLSDDSGSLVLYEALVETAKFAALWVPFCRKHQIEPRAPESYFELEGTLCGGASH
KEFIQDYKHVRTQYDEFKKHLDMLPNTIRQRSDIYSRTGTKDEDATVTWMADGTQWPGTWLDPTEKHRPGHHAGIV
KIVQSHPEHVVPLGVQESNDNPLNFDDVDMRLPMLVYVAREKSPGVEHNKKAGALNAELRISALLSNAPFFINFDCDH
YINNSEALRAAICFMLDPREGDNTGFVQFPQRFDNVDPTDRYGNHNRVFFDGAMYGLNGQQGPTYLGTGCMFRRLA
LYGIDPPCWRDEDIIVDSNRFGNSLLFLNSVLAAIKQEEGVTLQPPLDDSFLEEVTKVVSSSYDDSTDWGRGIGYIYNMA
TEDIVTGFRIHGQGWRSMYATMEREAFRGTAPINLTERLRQIVRWSGGSLEMFFSHISPLFAGRRLSLVQRLSYINFTIYP
LTSLFILMYAFCPVMWLLPTEILVQRPYTRYIVYLIIVIAMIHVIGMFEIMWAGITWLDWWRNEQFFMIGSVTAYPTAVL
HMVVNLLTKKGIHFRVTTKQPVADTDDKYAEMYEVHWVPMMVPAVVILFSNILAIGVAIGKSVLYMGTWSAAQRRH
GALGLLFNLWIMVLLYPFALAIIGRWAKRTGILFILLPIAFLSTALMYIGIHTFLLHFFPSMLI

>CSLF_2BS_TGACv1_148027_AA0489970.1_Triticum_aestivumMTTSPATAAGAATGLSEPLLSNGNGVHAGA
LVVTPVVANGHGGDKLKGDLKAKDKYWKDVDQPDDVAAAPDLENGGGRPLLFSNRRVKNIILYPYRVLILIRVIAVILFV
GWRIKHNNSDVMWFWVMSVVADVWFSLSWLSYQLPKYNPIKMIPDLATLRKQFDTPGSSSQLPGIDVIVTTASATDE
PILYTMNCVLSILAADYHIGRCNCYLSDDSGSLVLYEALVETAKFAALWVPFCRKHQTEPRAPARYFELEGPLCGGASHKE
FIQDYKHVRMQYEEFKKHLDMLPNTIRQRSDIYSKTGTKDEDAKVTWMADGTQWPGTWVDPAEKHRAGHHAGIVKI
VQSHPEHVVPLGVQESNDNPLNFDDVDMRLPMLVYVAREKSPGVEHNKKAGALNAELRISALLSNAPFFINFDCDHYI
NNSEALRAAVCFMLDPREGDNTGFVQFPQRFDNVDPTDRYGNHNRVFFDGAMYGLNGQQGPTYLGTGCMFRRLAL
YGIDPPCWRAEDMIVDSNRFGNSLPFLNSVLAAIKQEEGVTLPPPLDDSFLEEMTKVVSSSYDDSTDWGRGIGYLYNM
ATEDIVTGFRIHGQGWRSMYVTMEREAFRGTAPINLTERLRQIVRWSGGSLEMFFSHISPLFAGRRLSLVQRLSYINFTIY
PLTSLFILMYAFCPVMWLLPTEILIQRPYTRYIVYLLIVIAMIHVIGMFEIMWAGITWLDWWRNEQFFMIGSVTAYPTAV
LHMVVNLLTKKGIHFRVTTKQPVADTDDKYAEMYEVHWVPMMVPAVVVLFSNILAIGVAIGKSVLYMGTWSAAQKR
HGALGLLFNMWIMVLLYPFALAIIGRWAKRTGILFILLPIAFLSTALMYIGIHTFLLHFFPSMLI

>CSLF_2AS_TGACv1_113659_AA0359050.1_Triticum_aestivumMASAAGAGGANAGLADPLLASAKKPVGAK
GKHWVAADKDQRRAAKESGGEDGRPLLFRTYKVKGTLLHPYRALIFIRLIAVLLFFVWRIKHNKSDVMWFWTMSVVG
DVWFGFSWLLNQLPKFNPVKTIPDMVALRRQYDLPDGTSTLPGIDVFVTTADPIDEPILYTMNCVLSILASDYPVDRCAC
YLSDDSGALIQYEALVETAKFATLWVPFCRKHCIEPRAPESFFEQEAPLYTGSAPEEFKNDHNSVYIEYDEFKECLDSLSSAI

SKRSDAYNSMKTEEGDANATWMANGTQWPGSWIDTTEIHRKGHHAGIVKVVLDHSIRGHNLGSQASTHNLNFAST
DVRLPMLVYISRGKNPSYDHNKKAGALNAQLRASALLSNAQFIINFDCDHYINNSQALRAAMCFMLDQRQGDSTAFV
QFPQRFDNVDPSDRYGNHNRVFFDGTMLALNGLQGPSYLGTGCMFRRIALYGIDPPEWRHANIVVDDKRFGSSIPFLE
SVSKAINQERSTIPPPISETLVAEMERVVSASHDKATGWGKGVGYIYDIATEDIVTGFRIHGQGWRSMYCTMERDAFCG
IAPINLTERLHQIVRWSGGSLEMFFSLNNPLIGGRRIQSLQRVSYLNMTVYPVTSLFILLYALSPVMWLIPDEVYIQRPFTK
YVVFLLVIILMIHVIGWLEIKWAGVTWLDYWRNEQFFMIGSTSAYPAAVLHMVVNLLTKKGIHFRVTSKQTAADTNDK
FADLYDMRWVPMLIPTTVVLIANVGAIGVAMGKTIVYMGAWTIAQKTHAALGLLFNVWIMVLLYPFALAIMGRWAK
RPVILVVLLPVAFTIVCLVYVAVHILLLSYLTF

>CSLF_2DS_TGACv1_177641_AA0581710.2_Triticum_aestivumMASAAGAGGANAGLADPLLASAKKPVGAK
GKHWVAADKDQRRAAKESGGEDGRPLLFRTYKVKGTLLHPYRALIFIRLIAVLLFFVWRIKHNKSDIMWFWTLSVVGDV
WFGFSWLLNQLPKFNPVKTIPDMVALKRQYDLPDGTSTLPGIDVFVTTADPIDEPILYTMNCVLSILASDYPVDRCACYL
SDDSGALIQYEALVETAKFATLWVPFCRKHCIEPRAPESYFELEAPLYTGSAPEDFKNDHSSVHREYDEFKEHLDSISSAIS
KRSDAYNSMKTEEGDAKATWMANGTQWPGSWIDTTEIHRKGHHAGIVKVVLGHSIRGHNLGSQASTNNLNFASTD
VRLPMLVYISRGKNPSYDHNKKAGALNAQLRASALLSNAQFIINFDCDHYINNSQALRAAMCFMLDQRQGDSTAFVQ
FPQRFDNVDPSDRYGNHNRVFFDGTMLALNGLQGPSYLGTGCMFRRIALYGIDPPEWRHDNIVVDDKRFGSSIPFLES
VSKAINQERSTIPPPISETLVAEMERVVSASHDKATGWGKGVGYIYDIATEDIVTGFRIHGQGWRSMYCTMERDAFCGI
APINLTERLHQIVRWSGGSLEMFFSLNNPLIGGRRIQALQRVSYLNMTVYPVTSLFILLYALSPVMWLIPDEVYIQRPFTK
YVVFLLVIILMIHVIGWLEIKWAGVTWLDYWRNEQFFMIGSTSAYPAAVLHMVVNLLTKKGIHFRVTSKQTAADTNDK
FADLYDMRWVPMLIPTTVVLIANVGAIGVAMGKTIVYMGAWTIAQKTHAALGLLFNVWIMVLLYPFALAIMGRWAK
RPVILLVLLPVAFTIVCLVYVAVHILLLSYLTF

>CSLF_2BS_TGACv1_148608_AA0494060.1_Triticum_aestivumMASAVGAGGANAGLADPLLASRDGGAKKP
VGAKGKHWVAADKDQRRAAKESGGEEGRPLLFRTYKVKGTLLHPYRALIFIRLIAVLLFFVWRIKHNKSDIMWFWTMS
VVGDVWFGFSWLLNQLPKFNPVKTIPDMVALRRQYDLPDGTSTLPGIDVFVTTADPIDEPILYTMNCVLSILASDYPVD
RCACYLSDDSGALIQYEALIETAKFATLWVPFCRKHCIEPRAPESYFELEAPLYTGSASEEFKNDHSSVHREYDEFKEHLDS
LSSAISKRSDAYNSMKTGEGDAKATWMANGTQWPGSWIDTTEIHRKGHHAGIVKVVLDHSVRGHNLGSQASTHNLN
FANTDVRLPMLVYISRGKNPSYDHNKKAGALNAQLRASALLSNAQFIINFDCDHYINNSQALRAAMCFMLDQRQGDS
TAFVQFPQRFDNVDPSDRYGNHNRVFFDGTMLALNGLQGPSYLGTGCMFRRIALYGIDPPEWRHDNIVVDDKRFGSS
IPFLDSVSKAINQERSTIPPPISETLVAEMERVVSASHDKATGWGKGVGYIYDIATEDIVTGFRIHGQGWRSMYCTMER
DAFCGIAPINLTERLHQIVRWSGGSLEMFFSLNNPLIGGRRIQSLQRVSYLNMTVYPVTSLFILLYALSPVMWLIPDEVYI
QRPFTKYVVFLLVIILMIHVIGWLEIKWAGVTWLDYWRNEQFFMIGSTSAYPAAVLHMVVNLLTKKGIHFRVTSKQTAA
DTNDKFADLYDMRWVPMLIPTTVVLIANVGAIGVAMGKTIVYMGAWTIAQKTHAALGLLFNVWIMVLLYPFALAIM
GRWAKRPVILLVLLPVAFTIVCLVYVAVHILLLSYLTF

>CSLF_U_TGACv1_641498_AA2096480.1_Triticum_aestivumMPSPAAVGGGRLADPLLAADVVVGAKDKYWV
PADEREILASQKSGAGEDGRAPLLYRTFRVKGPLINLYRLLTLVRVIVVILFFTWRMRHRDSDAMWLWWISVVGDLWF
GVTWLLNQITKLRPRKCVPSISVLRDHLDQPDGGSDLPLLDVFINTVDPVDEPMLYTMNSILSILATDYPVEKYATYFSDD
GGSLVHYEGLQLAAEFAASWVPFCRKHCVEPRAPESYFWAKMRGEYAGSAPKEFLDDHRRMRAAYEEFKARLDGLSA
AIEQRSEACNRANGKDKEECANATWMADGSTQWQGTWIKPAKGHRKGHHPAILQVMLDQPSKDPELGMAASSDH
PLDFSAVDARLPMLVYIAREKRPGYDHQKKAGAMNVQLRVSALLSNAPFIINFDGDHYVNNSQAFRAAICFMLDPRDG
ADTAFVQFPQRFDDVDPTDRYCNHNRMFFDATLLGLNGIQGPSFVGTGCMFRRVALYSADPPRWRPDDAKEAKASR
YRPNMFGKSTSFINSMPAAANQERSVPSPATVGEAELADAMTCAYEDGTEWGNDVGWVYNIATEDVVTGFRLHRTG

WRSTYCAMEPDAFRGTAPINLTERLYQILRWSGGSLEMFFSRFCPLLAGRRLHPMQRVAYINMTTYPVSTFFICMYYLY
PVMWLFQGEFYIQRPFQTFALFVVVIIATVELIGMVEIRWAGLTLLDWVRNEQFYIIGTTGVYPMAMLHILLRSLGIKGV
SFKLTAKKLTGGARERLAELYDVQWVPLLVPTVVVMAVNVAAIGAAAGKAIVGRWSAAQVAGAASGLVFNVWMLLL
LYPFALGIMGRWSKRPYILFIVLVTAVAATASMYVALAGSLPYLHSGIKLV

>CSLF_2BS_TGACv1_146146_AA0456710.1_Triticum_aestivumMAAAVTRRANALRVEAPDGNTESGRASLAA
DSPVAKRAVDAKDDVWVAADEGEASGSIAGDGNRTPLFRTFKVKGSILHPYRFMILVRLVAIVAFFAWRVKHKNHDGV
WLWATSMVADVWFGFSWLLNQLPKLNPVKRVPDLAALADHSGDANLPGIDIFVTTVDPVDEPLLYTVNTILSILATDY
PVDKYACYLSDDGGTLVHYEAMIEVANFAVLWVPFCRKYCVEPRSPENYFGMKTQPYAGSMAGEFMRDHRRVRREY
DELKVRVDSLSTTIRQRSDAYNSSTKGDGVRATWMADGTQWPGTWIEQVENHRRGQHAGIVQVILGHPSCKPQLGS
PASSDNPLDFSNVDTRLPMLVYMSREKRPGYNHQKKAGAMNVMLRVSALLSNAPFVVNFDGDHYINNSQALCAPM
CFMLDPRDGQNTAFVQFPQRFDDVDPTDRYANHNRVFFDGTMLSLNGLQGPSYLGTGTMFRRVALYGMEPPRYRA
ENIKLAGKVNEFGSSTSFINSMPDGAIQERSITPVLVDEALSNDLATLMTCAYEDGSSWGRDVGWVYNIATEDVVTGFR
MHRQGWRSMYCSMEPAAFRGTAPINLTERLYQVLRWSGGSLEMFFSHSNALMAGRRLHPLQRIAYLNMSTYPIVTVF
ILAYNLFPVLWLFSEQFYIQRPFAWGFFTDQARHVLLGMLFNVWILVLLYPFALGIMGKWGKRPVILFVMLVMAVGAV
GLLYVAFHAPYPADFSEVAASLGEASLTGPSG

>CSLF_2DS_TGACv1_179076_AA0604160.1_Triticum_aestivumMAAAVTRRANALRAEAPDGNAESGRASLA
ADSPAAKRAVDAKDDVWVAADEGDTSGAIAGDGNRPPLFRTFKVKGSILHPYRFMILVRLVAIVAFFAWRVKHKNHD
GVWLWATSMVADVWFGFSWLLNQLPKLNPVKRVPDLAALADHSGDANLPGIDIFVTTVDPVDEPLLYTVNTILSILAT
DYPVDKYACYLSDDGGTLVHYEAMIEVANFAVLWVPFCRKYCVEPRSPENYFGMKTQPYAGSMAGEFMRDHRRVRR
EYDEFKVRVDSLSTTIRQRSDAYNSSKKGDGVRATWMADGTQWPGTWIEQVENHRRGQHAGIVQVILGHPSCKPQL
GSPASADNPLDFSNVDTRLPMLVYMSREKRPGYNHQKKAGAMNVMLRVSAMLSNAPFVVNFDGDHYINNSQALRA
PMCFMLDPRDGQNTAFVQFPQRFDDVDPTDRYANHNRVFFDGTMLSLNGLQGPSYLGTGTMFRRVALYGMEPPRY
RAENIKLAGKVNEFGSSTSFINSMPDGAIQERSITPVLVDEALSNDLATLMTCAYEDGSSWGRDVGWVYNIATEDVVTG
FRMHRQGWRSMYCSMEPAAFRGTAPINLTERLYQVLRWSGGSLEMFFSHSNALMAGRRLHPLQRVAYLNMSTYPIV
TVFILAYNLFPVLWLFSEQFYIQRPFGTYIMYLVAVIAMIHVIGMFEVKWAGITLLDWCRNEQFYMIGATGVYPTAVLY
MALKLVTGKGIYFRLTSKQTDACSNDKXXXXXXXXXXXXXXXXXXXXXXXGCRCCSRPSWCSS

>CSLF_2AS_TGACv1_112322_AA0335290.1_Triticum_aestivumMVSPATGGGRGGNAGLAEPLLATNDDSDG
AKHVFGAKAKHWVPADEKEMAASRECGGEDGRPLLYRTFKVRGFLVNTYRFLNLARLTAVIVFFAWRVQHPDSDAM
WLWWISVVGDFWFGLSWWLNQVPKLNPTICIPTIPLLRQQFDLPDGGSNLPVLDVFISTVDPVEEPMLHTMNSILSILA
TDYPVDKYATYLSDDGGSLLHYDGLVETAKFAALWVPFCRKHHVEPRAPESYFGMKVRPYKGNLPEEFLDDHRRLRREY
EEFKTRLDALFTVIPQRSEAHGREDAKGGGGAKATWMADGTQWPGTWTEPAEGHRKGDHAGIIQVMLSQPSGEPQ
LGAPASSDDNPLDFSAVDVRLPMLVYVSREKRPGYDHQKKAGALNVQLRVSALLSNAPFIINFDCDHYINNSQAFRAA
MCFMMDRRDGDNVAFVQFPQRFDDVDPTDRYANHNRMFFDATMLGMNGIQGPSYVGTGSMFRRVALYGADPPR
WRPDDVKVLENPNKFGKSMTFINSIPVAANQERSVMSPVSLDEPATTELADVMTCAYEDGTEWGDGVGWVYDMAT
EDAVTGFRLHRTGWRSMYCDMEPPAFCGTAPINMTERMYQILRWSGGSLEVFFSRFCPLLAGRRLHPMQRVAYTNM
TFYPLSALFVVCYHLLPLMWVFNGRFYIQKPYPTYVMYVLVIIVSNEVIGMVEIVWAGLTLLDWFRNEQFYMICATGVY
PTAVLHVVLRSLGLKGMSFKMTAKQLATGARERFAELYNVQWAPLLIPTLVVIAVNVVAIGAAVGKAITWGWSAGQV
VEAASGLMFNVWILLMFYPFALGVIGRWGKRPYVLFAMFVAAFAAIAAVYVAVQAALAGNLLYFQLGHWSIGGAVSL
PSRRV

>CSLF_2BS_TGACv1_147667_AA0486240.1_Triticum_aestivumMVSPATSGGRGGNAGLADPLLATNDDSDG
ARHVFGAKAKYWAPADEKEMTASRECSGEDGRPLLYRTFKVKGFLVNTYRFLNLARLTAVIVFFAWRVQHPDSDAMW
LWWISVVGDFWFGLSWWLNQVPKLNPTICIPTIPLLRQQFDLPDGGSNLPVLDVFISTVDPVEEPMLHTMNSILSILAT
DYPVDKYATYLSDDGGSLLHYDGLVETAKFAALWVPFCRKHHVEPRAPESYFGMKIRPYTGNLPEEFLDDHRRLRREYE
EFKTRLDALFTVIPQRSEAHGREDAKGGGGKATWMADGTQWPGTWTEPAEGHRKGDHAGIIQVMLSQPSSEPQLG
EPASSDDGPLDFSAVDVRLPMLVYVSREKRPGYDHQKKAGALNVQLRVSALLSNAPFIINFDCDHYINNSQAFRAAMC
FMMDRRDGDNVAFVQFPQRFDDVDPTDRYANHNRMFFDATMLGMNGIQGPSYVGTGSMFRRVALYGADPPRW
RPDDVKVLENPNKFGKSMTFINSIPVAANQERSVMSPVSLDEPATTELADVMTCAYEDGTEWGDGVGWVYDMATE
DAVTGFRLHRTGWRSMYCDMEPPAFCGTAPINMTERMYQILRWSGGSLEVFFSRFCPLLAGRRLHPMQRVAYTNMT
FYPLSALFVVCYHLLPLMWVFNGRFYIQKPYPTYVMYVLIIIISNEVIGMVEIVWAGLTLLDWFRNEQFYMICATGVYPT
AVLHVVLRSLGLKGISFKMTAKQLATGARERFAELYDVQWAPLLIPTLVVIAVNVVAIGAAVGKAITWGWSAGQVVEA
ASGLMFNVWILLMFYPFALGVIGRWGKKPYVLFAMFVAAFAAIAAVYVAVQAALAGNLPYFQLGHWSIGGAVSLPSR
RV

>CSLF_2DS_TGACv1_177329_AA0573830.1_Triticum_aestivumMVSPAASGGGNAGLADPLLATNDNSEGAR
HVFGAKAKYWVPADEKEIAASRECGGEDGRPLLYRTFKVKGMLVNTYRFLNLARLTAVIVFFAWRVQHPDSDAMWL
WWISVVGDFWFGLSWWLNQVPKLNPTICIPTIPLLRQQFDLPDGGSNLPVLDVFISTVDPVEEPMLHTMNSILSILATD
YPVDKYATYLSDDGGSLLHYDGLVETAKFAALWVPFCRKHHVEPRAPESYFGVKIRPYMGNLPEEFLDDHGRLRREYEE
FKTRLDALFTLIPQRSEAHGREDAKGGGGKATWMADGTQWPGTWTEPAEGHRKGDHAGIIQVMLSQPSSEPQLGEP
ASSDHSPLDFSAVDVRLPMLVYVSREKRPGYDHQKKAGALNVQLRVSALLSNAPFIINFDCDHYINNSQAFRAAMCFM
MDRRDGDNVAFVQFPQRFDDVDPTDRYANHNRMFFDATMLGMNGIQGPSYVGTGSMFRRVALYGADPPRWRPD
DVKVLENPNKFGKSMTFINSIPVAANQERSVMSPVSLDEPATTELADVMTCAYEDGTEWGDGVGWVYDMATEDAV
TGFRLHRTGWRSMYCDMEPPAFCGTAPINMTERMYQILRWSGGSLEVFFSRFCPLLAGRRLHPMQRVAYTNMTFYP
LSALFVVCYHLLPLMWVFNGQFYIQKPYPTYVMYVLIIIVSNEVIGMVEIVWAGLTLLDWFRNEQFYMICATGVYPTAV
LHVVLRSLGLKGMSFKMTAKQLATGARERFAELYDVQWAPLLIPTLVVIAVNVVAIGVAVGKAITWGWSAGQVVEAA
SGLMFNVWILLMFYPFALGVIGRWGKRPFVLFAMFVAAFAAIAAVYVAVQAALAGNLPYFQLGHRSIGGAVSLASRRV

>CSLF_2BS_TGACv1_148916_AA0495580.1_Triticum_aestivumMIYTMNSIISILAADYPVDKHACYLSDDGGSII
HYDGLLETAKFAALWVPFCRKHSIEPRAPESYFSLNTRPYTGNAPQDFVNDRRHMCREYDEFKERLDALFTLIPKRSDVY
NHAAAKEGAKATWMADGTQWPGTWIDPAENHKKGQHVGIVKVMLKHPSYEPELGLGASTNSPLDFSAVDVRLPML
VYISREKSPSCDHQKKAGAMNVQLRVSALLTNAPFIINFDGDHYVNNSKAFRAGICFMLDRREGDNTAFVQFPQRFDD
VDPTDRYCNHNRVFFDATLLGLNGIQGPSYVGTGCMFRRVALYGVDPPRWRPDDVKIVDSSTKFGSSASFISSILPAAD
QERSIMSPPALEEPVMADLAHVMTCAYEDGTEWGREVGWVYNIATEDVVTGFRLHRNGWRSMYCRMEPDAFAGT
APINLTERLYQILRWSGGSLEMFFSRNCPLLAGRRLHPMQRIAYANMTAYPVSSVFLVFYLLFPVIWIFRGQFYIQKPFPT
YVLYLVIVIALTELIGMVEIKWAGLTLLDWIRNEQFYIIGATAVYPTAVFHIVLKLFGLKGVSFKLTAKQVASSTSDKFAELY
AVQWAPMLIPTMVVIAVNVCAIGASIGKAIVGGWSLMQMADAGLGLVFNAWILVLIYPFALGMIGRWSKRPYILFILF
VIAFILIALVDIAIQAMRSGFVRFHFKSSGGATFPTSWGL

>CSLF_2DS_TGACv1_178471_AA0596060.1_Triticum_aestivumMIYTMNSIISILAADYPVDKHACYLSDDGGSII
HYDGLLETAKFAALWVPFCRKHSIEPRAPESYFSLNTRPYTGNAPQDFVNDRRHMCREYDEFKERLDALFTLIPKRSDVY
NHAAAKEGAKATWMADGTQWPGTWIDPAENHKKGQHVGIVKVMLKHPSYEPELGLGASTNSPLDFSAVDVRLPML
VYISREKSPSCDHQKKAGAMNVQLRVSALLTNAPFIINFDGDHYVNNSKAFRAGICFMLDRREGDNTAFVQFPQRFDD
VDPTDRYCNHNRVFFDATLLGLNGIQGPSYVGTGCMFRRVALYGVDPPRWRPDDVKIVDSSTKFGSSASFISSILPAAD

QERSIMSPPALEESVMADLAHVMTCAYEDGTEWGSDVGWVYNIATEDVVTGFRLHRNGWRSMYCRMEPDAFAGT
APINLTERLYQILRWSGGSLEMFFSRNCPLLAGRRLHPMQRIAYANMTAYPVSSVFLVFYLLFPVIWIFRGQFYIQKPFPT
YVLYLVIVIALTELIGMVEIKWAGLTLLDWIRNEQFYIIGATAVYPTAVFHIVLKLFGLKGVSFKLTAKQVASSTSDKFAELY
AVQWAPMLIPTMVVIAVNVCAIGASIGKAIVGGWSLMQMADAGLGLVFNAWILVLIYPFALGMIGRWSKRPYILFILF
VIAFILIALVDIAIQAMRSGFVRFHFKSSGGATFPTSWGL

>CSLF_2AS_TGACv1_112322_AA0335280.1_Triticum_aestivumMGSLAAANGAGHASNGAGVADQALALEN
GTGNGHKAGVANRATPPLQANGGSKVAKKISPKDKYWVAADEGEMAAAIADGGEDGRRPLLYRTFKVKGILLHPYRL
LSLIRLVAIVLFFVWRVRHPYADGMWLWWISMVGDLWFGVTWLLNQVAKLNPVKRVPNLTLLEQQFDLPDGNSNLP
CLDVFINTVDPINEPMIYTMNSIISILAADYPVDKHACYLSDDGGSIIHYDGLLETAKFAALWVPFCRKHSIEPRAPESYFSL
NTRPYTGNAPQDFVNDRRHMCREYDEFKERLDALFTLIPKRSDVYNHAAAKEGAKATWMADGTQWPGTWIDPAEN
HKKGQHVGIVKVMLKHPSYEPELGLGASTNSPLDFSAIDVRLPMLVYISREKSPSCDHQKKAGAMNVQLRVSALLTNAP
FIINFDGDHYVNNSKAFRAGICFMLDRREGDNTAFVQFPQRFDDVDPTDRYCNHNRVFFDATLLGLNGIQGPSYVGTG
CMFRRVALYGVDPPRWRPDNVKIVDSSTKFGSSASFISSILPAADQERSIMSPPALEEPVMADLAHVMTCAYEDGTEW
GREVGWVYNIATEDVVTGFRLHRNGWRSMYCRMEPDAFAGTAPINLTERLYQILRWSGGSLEMFFSRNCPLLAGRRL
HPMQRIAYANMTAYPVSSVFLVFYLLFPVIWIFRGQFYIQKPFPTYVLYLVIVIALTELIGMVEIKWAGLTLLDWIRNEQFY
IIGATAVYPTAVFHIVLKLFGLKGVSFKLTAKQVASSTSDKFAELYAVQWAPMLIPTMVVIAVNVCAIGASIGKAIVGGW
SLMQMADAGLGLVFNAWILVLIYPFALGMIGRWSKRPYILFILFVIAFILIALVDIAIQAMRSGFVRFHFKSSGGATFPTS
WGL

>CSLF_5BL_TGACv1_409916_AA1366600.2_Triticum_aestivumMSMTYISKKHDYAATLDEKEQPKDQKSASVE
SLLVRTTKLTTVTIKLYRIMVFVRMAIFVLFFKWRISTALAMISDGATTVRAMWTMPIAGELWFALMWVLDQLPKMQP
VRRTVFATALEEPLLPTMDVFVTTADPEKEPPLVTVNTILSILAADYPPDKLTCYVSDDGGALLTREAVAHAARFARLWV
PFCRKHGVEPRNPEAYFCPGVKARVVSRAAYMGRSWPELARDRRRVRREYEELRLRIDALHAGDVRRQPWSRGTPEY
HAGVVEVLVGPPGSTPELGVSDLLDLTSVDVRVPAVVYMCREKRHGRVHHRKAGAMNALLRTSAVLSNAPFILNLDCD
HYVSNSQALRAGVCLMLDRGGSNVAFVQFPQRFDGVDPADRYANHNRVFFDCTELGLDGLQGPIYVGTGCMFRRAA
LYNADPPLWRPHGGDRDAGKDVATEADKFGISTPFLVSVRAALNLNRSEQWNTTSPPRSFDGAAVGEATALVSCGYE
DRTAWGRDIGWIYGTVTEDVATGFCMHRRGWRSAYCATAPDAFRGTAPINLTDRLHQVLRWAAGSLEIFFSRNNALL
AGARLHPLQRLAYLNTTVYPFTSIFLLLYCLLPAIPLVTRSASTSAFSVNTPPSATYIGFVAALMLTLAMVAALEVRWSGITL
GEWWRNEQFWMVSATSAYAAAVVQVALKVAAGKEIAFKLTSKQRAPSAGGGVVKRQVRGAVRREMDGADGSDG
GGADGERGVHGSSGTRGTVEERPRGGARDGVQRVGGGASPPVRPWSHGPLEQDVEPPALARRSVHSSITMFCPPFA
YALIWLLFG

>CSLF_5DL_TGACv1_433902_AA1424880.1_Triticum_aestivumMSMTYITKKHDYAATLDEKEPSEDQKSASVK
NLLVRTTKLTTVTIKLYRLMVFVRLTIFVLFFKWRVSTALTVISDGTTTARAMWTMSIAGELWFALMWVLDQLPKMQP
VRRTVYVTALEEPRLPTMDVFVTTTDPEKEPPLVTVNTILSILAADYPPDKLTCYVSDDGGALLTREAVAHAACFARLWV
PFCRKHGVEPRNPEAYFCPGVKARVVSRADYMGRSWPELARDRRRVRREYEELRLRIDALHAGDVRPQQWSRGTAEN
HAGVVEVLVGPPGSTPELGVSDLLDLSSVDVRVPAVVYMCREKRHGRVHHRKAGAMNALLRTSAVLSNAPFILNLDCD
HYVNNSQALRAGVCLMLDRGGSNVAFVQFPQRFDGVDPADRYANHNRVFFDCTELGLDGLQGPIYVGTGCMFRRA
ALYNADPPLWRPHGGDRDAGKDVATEADKFGISTPFLGSVRAALGLNRSEQWNTTTKPPRSFDGAAVGEATALVSCG
YEDRTAWGRDIGWIYGTVTEDVATGFCMHRRGWRSAYCATAPDAFRGTAPINLTDRLHQVLRWAAGSLEIFFSRNNA
LLAGARLHPLQRLAYLNTTVYPFTSIFLLLYCLLPAIPLVTRSASASAFSVTMPPSGTYMGFVAALMLTLAMVAVLEVRWS
GITLGEWWRNEQFWMVSATSAYAAAVVQVALKVSAGKEIAFKLTSKQRASSPGGGVKERFAELYAVRWTVLMVPTA

VVLAVNVMSMAAAVQEGRWRKGPAAVLAMAFNAWVVVHLHPFALGLMGRWSKTLSPLLLLVVGFTVLSLCFVLHL
HML

>CSLF_5AL_TGACv1_374191_AA1193100.1_Triticum_aestivumMSMTYITKKHDYVASLDGKESPEHEKSASVE
RLLVRTTKLTTVTIKLYRLVVFVRMIIFVLFFKWRSSTALAMISDGTTTVRAMWTMSIAGELWFALMWVLDQLPKMQP
VRRTVYATALEESLLPAMDVFVTTADPEKEPPLVTVNTILSILAADYPPDKLTCYVSDDGGALLTREAVAQAAWFARLW
VPFCRKHGVEPRNPEAYFCPGVKARVVSRADYRAKSWPELARDRRRVRREYEELRLRIDALHAGDVRRQQWSRGTAE
DHAGVVEVLVGPPGSTPELGVSDLLDLGSVDVRVPAVVYMCREKRHGRVHHRKAGAMNALLRTSAVLSNAPFILNLD
CDHYVNNSQALRAGVCLMLDRGGSNVAFVQFPQRFDGVDPADRYANHNRVFFDCTELGLDGLQGPIYVGTGCMFR
RAALYNADPPLWRPHGDRDAGKDVAAEADKFGISTPFLGSVRAALNLNQSEQWNTTSPPRSFDGAAVGEATALVSCG
YEDRTAWGRDIGWIYGTVTEDVATGFCMHRRGWSSAYCATAPDAFRGTAPINLTDRLHQVLRWAAGSLEIFFSRNNA
LLAGPRLHPLQRLAYLNTTVYPFTSIFLLLYCLLPAIPLVTRNASTSAFSVNTPPSATYIAFVAALMLTLAMVAVLEVRWSGI
TLGDWWRNEQFWMVSATSAYAAAVVQVALKVAAGKEIAFKLTSKHRASNSGGGVVKDRFAELYAVRWTVLMVPTA
VVLAVNVTSMAAAVQEGRWRKGPAAVLAMAFNAWVVVHLYPFALGLMGRWSKTLSPLLLLVVVFTVLSLCFVLHLH
ML

>CSLF_7BL_TGACv1_577473_AA1876170.1
peptideMAPAVAGGGRVRSNEPAAAAASDKPCVCGFQVCACTGSAAVASAASSLDMDIVAMGQIGAVNDESWVGV
ELGEDGETDESGAAVDDRPVFRTEKIKGVLLHPYRVLIFVRLIAFTLFVIWRISHKNPDAMWLWVTSICGEFWFGFSWLL
DQLPKLNPINRVPDLAVLRQRFDRPDGTSTLPGLDIFVTTADPIKEPILSTANSVLSILAADYPVDRNTCYVSDDSGMLLTY
EALAESSKFATLWVPFCRKHGIEPRGPESYFELKSHPYMGRAQDEFVNDRRRVRKEYDEFKARINSLEHDIKQRNDGYN
AANAHREGEPRPTWMADGTQWEGTWVDASENHRRGDHAGIVRVLLNHPSHRRQTGPPASADNPLDFSGVDARLP
MLVYVSREKRPGHDHQKKAGAMNALTRASALLSNSPFILNLDCDHYINNSQALRAGICFMVGRDSDTVAFVQFPQRFE
GVDPTDLYANHNRIFFDGTLRALDGMQGPIYVGTGCLFRRITVYGFDPPRINVGGPCFPRLAGLFAKTKYEKPSLEMTM
AKAKAAPVPAKGKHGFLPLPKKTYGKSDAFVDSIPRASHPSPYAAAAEGIVADEATIVEAVNVTAAAFEKKTGWGKEIG
WVYDTVTEDVVTGYRMHIKGWRSRYCSIYPHAFIGTAPINLTERLFQVLRWSTGSLEIFFSKNNPLFGSTYLHPLQRVAYI
NITTYPFTAIFLIFYTTVPALSFVTGHFIVQRPTTMFYVYLGIVLSTLLVIAVLEVKWAGVTVFEWFRNGQFWMTASCSAY
LAAVCQVLTKVIFRRDISFKLTSKLPSGDEKKDPYADLYVVRWTPLMITPIIIIFVNIIGSAVAFAKVLDGEWTHWLKVAGG
VFFNFWVLFHLYPFAKGILGKHGKTPVVVLVWWAFTFVITAVLYINIPHMHSSGGKHTTVHGHHGKKFVDAGYYNWP

>CSLF_7AL_TGACv1_555973_AA1751470.1
peptideMAPAVAGGGRVRSNEPAAAAAPAAASGKPCVCGFQVCACTGSAAVASAASSLDMDIVAMGQIGAVNDES
WVGVELGEDGETDESGVAVDDRPVFRTEKIKGVLLHPYRVLIFVRLIAFTLFVIWRISHKNPDAMWLWVTSICGEFWFG
FSWLLDQLPKLNPINRVPDLAVLRQRFDRPDGTSTLPGLDIFVTTADPIKEPILSTANSVLSILAADYPVDRNTCYVSDDSG
MLLTYEALAESSKFATLWVPFCRKHGIEPRGPESYFELKSHPYMGRAQDEFVNDRRRVRKEYDEFKARINSLEHDIKQRN
DGYNAANAHREGEPRPTWMADGTQWQGTWVDASENHRRGDHAGIVLVLLNHPSHRRQTGPPASADNPLDFSGV
DVRLPMLVYVXXXXXXXXXXXXXXXXXXXXXXXXXXXXXXXXXXXXYANHNRIFFDGTLRALDGMQGPIYVGTGCLFRRITV
YGFDPPRINVGGPCFPRLAGLFAKTKYEKPGLEMTMAKAKAAPVPAKGKHGFLPLPKKTYGKSDAFVDSIPRASHPSPY
AAAAEGIVADEATIVEAVNVTAAAFEKKTGWGKEIGWVYDTVTEDVVTGYRMHIKGWRSRYCSIYPHAFIGTAPINLTE
RLFQVLRWSTGSLEIFFSKNNPLFGSTYLHPLQRVAYINITTYPFTAIFLIFYTTVPALSFVTGHFIVQRPTTMFYVYLGIVLST
LLVIAVLEVKWAGVTVFEWFRNGQFWMTASCSAYLAAVCQVLTKVIFRRDISFKLTSKLPSGDEKKDPYADLYVVRWTP
LMITPIIIIFVNIIGSAVAFAKVLDGEWTHWLKVAGGVFFNFWVLFHLYPFAKGILGKHGKTPVVVLVWWAFTFVITAVL
YINIPHMHSSGGKHTTVHGHHGKKFVDAGYYNWP

>CSLF_7DL_TGACv1_607937_AA2011180.1_Triticum_aestivumMVGRDSDTVAFVQFPQRFEGVDPTDLYAN
HNRIFFDGTLRALDGMQGPIYVGTGCLFRRITVYGFDPPRINVGGPCFPRLAGLFAKTKYEKPGLEMTMAKAKAAPVPA
KGKHGFLPLPKKTYGKSDAFVDSIPRASHPSPYAAAAEGIVADEATIVEAVNVTAAAFEKKTGWGKEIGWVYDTVTEDV
VTGYRMHIKGWRSRYCSIYPHAFIGTAPINLTERLFQVLRWSTGSLEIFFSKNNPLFGSTYLHPLQRVAYINITTYPFTAIFLI
FYTTVPALSFVTGHFIVQRPTTMFYVYLGIVLSTLLVIAVLEVKWAGVTVFEWFRNGQFWMTASCSAYLAAVCQVLTKV
IFRRDISFKLTSKLPSGDEKKDPYADLYVVRWTPLMITPIIIIFVNIIGSAVAFAKVLDGEWTHWLKVAGGVFFNFWVLFHL
YPFAKGILGKHGKTPVVVLVWWAFTFVITAVLYINIPHMHSSGGKHTTVHGHHGKKFVDAGYYNWP

>CSLF_1BS_TGACv1_049866_AA0163180.1
peptideMASPAAGGGGRLADPLLATDVVVGPKDKYWVPADEREILASHRSGAGGDDGRAPLLYRTFRVKGPLINLYRL
LTLVRVIVVILFFTWRMRHRDSDAMWLWWISVVGDLWFGVTWLLNQITKLRPRKCVPSISVLREQLDQPDGGSDLPLL
DVFINTVDPVDEPMLYTMNSILSILATDYPVDKYATYFSDDGGSLVHYEGLQLAAEFAASWVPFCRKHCVEPRAPESYF
WAKMRGEYAGTAPKEFLDDHRRMRAAYEEFKVRLDGLSAAIEQRSEACNRANGKEEGADATWMADGSTQWQGT
WIKPAKGHRKGHHPAILQVMLDQPSKDPELGMAASSGHPLDLSAVDARLPMLVYIAREKRPGYDHQKKAGAMNVQL
RVSALLSNAPFIINFDGDHYVNNSQAFRAAMCFMLDPRDGADTAFVQFPQRFDDVDPTDRYCNHNRMFFDATLLGL
NGIQGPSFVGTGCMFRRVALYSADPPRWRPDDAKEAKASRYRPNMFGKSTSFINSVPAAANQERSVPSPATVGEAEL
ADAMTCAYEDGTEWGNDVGWVYNIATEDVVTGFRLHRTGWRSTYCAMEPDAFRGTAPINLTERLYQILRWSGGSLE
MFFSRFCPLLAGRRLHPMQRIAYINMTTYPVSTFFICMYYFYPVMWLFQGEFYIQRPFQTFALFVVIVIATVELIGMVEIR
WAGLTPLDWFRNEQFYIIGTTGVYPMAMLHIILRSLGIKGVSFKLTAKKLTSGTRERLAELYDVQWVPLLVPTVVVMAV
NVAAIGAAAGKAIAGRWSAAQVAGAASGLVFNVWMLLLLYPFALGIMGRWSKRPYILFIVLVTAVAATASVYVALAGS
LPYLHSGIKLV

>CSLJ_3AS_TGACv1_210908_AA0681280.2_Triticum_aestivumMAARPSQDAPLQLHTVQTDQPLATVNRLLA
ALHVALAAAAIAHRGAHVMLAPDLVLLFLWALSQAPMWRPVSRAAFPSRLSRPALPAVDVMVVTADPDKEPAAKVM
NTVVSAMALDYPGGRLSVYLSDDAGSPRTLLAARKAYALARAWVPFCRKYGVRCPCPDRFFAGDDQLDLGDHHRQEL
DDDRLRIKNVYETFKEGVEEVMNDATLSQSWTKADHHAHVEIITDEQGQDSSHSNSGDGDGDEDAMPLLVYVSREKR
RSSTHHFKAGALNVLLRVSSLMSNSPYVMVLDCDMYCNSRSSLLEAMCFHLDGRRRADLAFVQFPQMFHNLSSSDIYA
NELRPIFWVRKKTNRPCIASVIFSEFSSNLGACMVQTRWKGLDGLRGPILSGTGFCVRRDAVYGAGPGSSQEQFSGVEV
GELKRRFGVSNGHIASLRRSGTGSTIVAAGDVLPQDAELVASCDYETGTEWGEDVGFLYQSVVEDYFTGYRQLYCRGW
TSVYCFPATGSRPPFLGSVPTNLNDALVQNKRWMSGLLAVGLSRHCPLASAAAISVPQSMGFAYYAFTPLYAFPLLCYA
TVPQLCFLRGATSFPEAASTLWFAAVFASSSLQHLVEVSVAKRGLAARTWWNEQRFWALNAVTGQIFACLGVALSLVG
AGGRAVDFDLTSKASGDRLYRDGVFDFAGCSALLLPATTLCLLNAAALVGGVWKMVGRGGNVSGTGELFLLCYVAALS
YPLLQGMFLRRDPARVPARITAVSVAIVATLLSLFG

>CSLJ_3B_TGACv1_221705_AA0747940.1_Triticum_aestivumMAAKPSQDAPLQLHTVEVDQPIATVNRLLAVL
HVALAAAAIAHRGAHVMLAADLVLLFLWALSQAPMWRPVSRAAFPSRLSRAALPAVDVMVVTADPDKEPAAKVMN
TVVSAMALNYPGGRLSVYLSDDAGSPRTLLAARKAYAFARAWVPFCRKYGVRCPCPDRFFAGDDQLDLDGHHRQELD
DDRLRIKKMYETFKEGVEEVMSDAALSQSWTKADHDAHVEIITGDEQDSSNSNSGDGEEDEDATPLLVYVSRGKRRSS
THHFKAGALNVLLRVSSLMSNSPYVMVLDCDMYCNSRSSILEAMCFHLDGRRRADLAFVQFPQMFHNLSTSDIYANEL
RSIFWTRWKGLDGLRGPILSGTGFCARRDAIYGALPASSQDQFSGVEVGELKRRFGVSNGHIASLRRPGTGSTIVARDAL
PQDAELVACCDYETGTEWGEEVGFLYQSVVEDYFTGYRQLYCRGWTSVYCFPATGSRPPFLGSVPTNLNDALVQNKR
WMSGMLAVGLSRHCPLASAAAISVPESMGFAYYAFMALYAFPVLCYAIVPQLCFFRGGTSFPEASTLWFAAVFVSSSL
QHLVEVSVAKRGLAARTCWNEQRFWALNAVTGQLFACLSVALNLVDGAGGRAVDFDLTSKASDDRLYRDGVFDFAG

CSTLLLPATTLCLLNAAALVGGVWKMVGRGGNMPGELFLLCYIAALSYPLLQGMFLRRDLARVPARITAMSVAMVATL
LSLFG

>CSLJ_3DS_TGACv1_272756_AA0924850.1_Triticum_aestivumMATKPSQDAPLPLHTVQTDQPLATVNRLLA
AVHLALGAAAIAHRGAHVMLAADLVLLFLWALSQAPMWRPVSRTAFPSRLSRAALPAVDVMVVTADPEKEPAAKVM
NTVVSAMALDYPGGRLSVYLSDDAGSPRTLLAARKAYAFARAWVPFCRKYGVRCPCPDRFFAGDDKLDLGSHHHHEL
ADDRLRIKNMYETFNEGVREVMSDADLSQSCTKADHDAHVEIITGDEQDSSNSNSGDGEEDEDAMPLLVYVSREKRRS
STHHFKAGALNVLLRVSSLLSNSPYVMVLDCDMYCNSRSSILEAMCFHLDGRRRADLAFVQFPQMFHNLSSSDIYANEL
RSIFWTRWKGLDGLRGPILSGTGFCARRDAIYGARPASSQDQFSGVEVGELKRRFGVSNGHIASLRRSGTGSTIVARDAL
PQEDAELVASCAYETGTEWGEQVGFLYQSVVEDYFTGYRQLYCRGWTSVYCFPAAASRPPFLGSVPTNLNDALVQNK
RWMSGMLAVGLSRHCPLASAAICVPQSMGFAYYAFMALYAFPVLCYATVPQLCFLRGGTSFPGAASTLWFAAVFASS
SLQHLVEVSVAKRGLALRTWWNEQRFWALNAVTGQLFACLGVALNLVGAGGRAVDFDLTSKASDDRLYRDGVFDFA
GCTTLLLPATTLCLLNAAALVGGVWKMVGRGGSVSGELFLLCYVAALSYPLLQGMFLRRDPARVPAPITAMSVAMVAA
LLSLFG

>CSLJ_3DS_TGACv1_272297_AA0918580.1_Triticum_aestivumMAAEPSQDAPLQLHTVQTDQPLATVNRHLA
ALHVALAAAAIAHRGAHVMLAADLVLLFLWALSQAPMWRPVSRAAFPSRLSRAALPAVDVMVVTADPDKEPAAKV
MNTVVSAMALDYPGGRLSVYLSDDAGSPRTLLAARKAYAFARAWVPFCRKYGVRCPCPDRFFAGDDQLDLGGHHRQ
ELDDDRLRIKNMYETFKEGVEKVMNDAALSQSWTKADHDAHVEQDSSNSNSGDGEEDEDAMPLLVYVSREKRRSSA
HHFKAGALNVLLRVSSLMSNSPYVMVLDCDMYCNSWSSVLEAMCFHLDGRRRADLAFVQFPQMFHNLSSSDIYANE
LRSIFWAGPTGLRDAVERRGRPPGPILSGTGFCVRRDAVYGAGPGSSQDHQSSGVEVGELKRRFGVSNGHIASLRRSGT
GSTIVARDGLPQPQEDAELVASCDYETGTEWGEEVGFLYQSVVEDYFTGYRQLYCPGWTSVYCFPATGTRPPFLGSVPT
NLNDALVQNKRWMSGMLAVGLSRHCPLASAAAVSVPQSMGFAYYAFMALYAFPVLCYATVPQLCFLRGGTSFPGES
ALWFAAVLASSSLQHLVEVSFAKRGLAARAWWNEQRFWALNAVTGQLFACVSVALSLVGAGGRAVDFDLTSKASDD
RLYRDSVFDFAGCSALLLPATTLCLLNTAALVGGVWKMVGRGGSVSGELFLLCYVAALSYPLLEGMFLRRDPARVPAWI
TAMSVAMVATLLSLFG

>CSLH_3DS_TGACv1_271739_AA0907200.1_Triticum_aestivumMSSAMKLQERVIVPRTAWKLADIFILCLLFAL
LSCRVLSLGEGGAGAASVAALVCEAWFTFVWILNMNIRWNPVRFHTYPENLSQRMDGLPAVDMLVTTADPELEPPL
MTVNTVLSLLAMDYPDVDKLACYVSDDGCSPVTCYALHEAARFAGLWVPFCKRHGVGVRAPFMYFASRPEPELAGDN
FSDEWTFIKSEYDKLVSRIESADEGSLLRDDDAGEFTEFMEAKRGDHPGIVKVLWDNSKSSRTGEGFPNLVYVSREKSRK
HDHHYKAGAMNVLARVSAVMTNAPIILNVDCDMFVNNSQVVLHAMCLLLGFDDETCSGFVQVPQRFYGKLKDDPF
GNQMEVLREKLFGGLAGLQGIYYLGMGCFHRRKIIYGVAPSSSAAIKHEREGSRSYEDLRTKFGASVELVESARNIYSGEI
PPSPMIDISSRIQVAKQVSSCNYETDTHWGQEIGWSYGSMAEDILTGQRIHSSGWKSTLLDTNPPAFLGCAPTGGPASL
TQYKRWATGLLEILLGQNSPIMATVFKRLQFRQSLAYLVFYVWSMRAPFELCYALLGPFCLFRNQSFLLKASNHGFSIQL
ALFLSYNIYNFVEYMECGLSARTWWNNMRMQRIVSISSWLLDFLSVVLKTIGLSKTVFEVTRKDKSTSDGDPSTHETDL
GWFTFDSSPVFILVTTVAILNIATIAIGVWRHAIFWMITGNHDCQNIGELCVLDG

>CSLH_3AS_TGACv1_212952_AA0704280.1_Triticum_aestivumMIDISSRIQVAKQVSSCNYETDTHWGQEIG
WSYGSMAEDILTGQRIHSSGWKSTLLDTNPPAFLGCAPTGGPASLTQYKRWATGLLEILLGQNSPIIATIFKRLQFRQFLA
YLVFYVWSMRAPFELCYALLGPFCLFRNQSFLLKASNHGFSIQLALFLSYNIYNFVEYMDCGLSARTWWNNMRMQRIV
SISSWLLAFLSVVLKTIGLSKTVFEVTREDKSTSDGDPSTHETDLGWFTFDSSLVFIPVTTVAILNIATIAIGVWRHAIFWMI
TGNHDWQNIGEFICCGWAILYFWPFIKGLVGRGRYGIPWNVKLKAWVIVVAFLYFCRGD

>CSLH_3B_TGACv1_222234_AA0760340.1_Triticum_aestivumMSSAMKLQERVSVPRTAWKLADIFILCLLFAL
LSCRVASLREGGASVAALVCEAWFTFVWIINMNIKWNPVRFNTYPENLSQRTDELPAVDMLVTTADPELEPPLMTVNT
VLSLLAVDYPDVDKLACYVSDDGCSPVTCYALREAAGFARLWVPFCKRHGVGVRAPFMYFASSRPEPELAGDWTFIKSE
YDKLVSRIESADEGSLLRHDDAADFTEFKEAKRGDHPAIVKVLWDNSKSSRTGSGDGFPNLVYVSREKTRKHDHHYKAG
AMNVLARVSAVMTNAPIILNMDCDMFVNNPQVVLHAMCLLLGFNDETCSGFVQVPQRFYAKLKDDPFGNQIEVLRE
KLLGGLSGLQGIYYLGTGCFHRRKIIYGVAPPSFAAVKHERQGSLTYEDLRTKFGASVELAESARNIYSREIPLKPMIDISSRI
QVAKQVSSCNYETGTHWGQEIGWSYGSMAEDILTGQRIHSAGWKSTSPDTNPPAFLGCAPTGGPASLTQYKRWATG
LLEILLGPNTPIIATIFKRLQFRQYLGYLVFYVWSMRAPFELCYALLGPFCLFRNHSFLLKASNHGFSIQLALFLSYNIYNFVE
YMECGLSARTWWNNMRMQRIVSLSSWLLAFLSVVLKTIGLSKTVFEVTRKDKSTSDGDPSTHETDLGWFTFDSSPVFIP
MTAVAILNIVTIAIGVWRHAIFWMTTGNHDCQNIGEFLCCGLMILYFWPFIKGLVGRGRYGIPWNVKLKAWVIVVAFL
YFCRGD

>CSLH_3B_TGACv1_221049_AA0728260.1_Triticum_aestivumMSSAMKLQERVTVPRTAWKLADIFILCLLLVL
LSCRVASLGEGGAGAAALVCEAWFTFVWILNMNIKWNPVRFHTYPENLSQRMDELPAVDMLVTTADPELEPPLMTV
NTVLSLLAVDYPDVDKLACYVSDDGCSPVTCYALREAAGFARLWVPFCKRHGVGVRAPFIYFASSRPEPDLAGDKFSDD
WIFIKSEYDKLVSLIESADEASLLRHDHAGEFTEFKGAECGDHPAIVKVLWDNSKSSGTGEGFPNLVYVSREKSRKHDHH
YKAGAMNVLARVSAVMTNAPIILNVDCDMFVNNPQVVLHATCLLLGFDDETCSGFVQVPQRFYGKLKDDPFGNQME
VLRSLSYEDLLTKFGASMELVESSRNIYSVEIPPKPMIDITSRIQVAKQVSTCNYETGTHWGEEASNHGFSIQLALFLSYNIY
NFVEYKECGLSARTWWNNMRMRINLLLAPCFP

>CSLH_3DS_TGACv1_273502_AA0931770.1_Triticum_aestivumMGSAMKLQERVILPRTAWKLADIFILCLLFAL
LSCRVASLGDGGAGAASVAALVCEAWFTFVWILNMNIKWNPVRFHTYPENLSQRMDELPAVDMLVTTADPELEPPL
MTVNTVLSLLAVDYPDVDKLACYVSDDGCSPATCYALREAAWFARLWVPFCKRHDVRVRAPIIYFASRLEPELAGDTFS
DEWTFIKSEYDKLVSRIESADEGSLLRHDDAGEFTEFMEAERTDHPAIVKVLWDNSKSSRTGEAFPHLVYVSSEKSRKHH
HHYKAGAMNVLARVSAVMTNAPIILNVDCDMFVNNSQVVLHAMCLLLGFDDETCSGFVQVPQRFYGKLKDDPFGN
QMEVLREKLLGGLSGLQGIFYLGTGCFHRRKIIYGVAPSSFAAVKHEREGSLSYEDLRTKFGASVELVESTRNIYSREIPPKP
MVNISSCIQVAKQVSSCNYETGTHWGQEIGWSYGSMAEDILTGQRIHSAGWKSTLLDTNPPAFLGCAPTGGPASLTQ
YKRWATGVLEILLGQNCPIIATIFKRLQFRQCLAYLVFYVWSMRAPFELCYALLGPFCLFRNHSFLLKHQTMVSASN

>CSLH_2AL_TGACv1_094351_AA0296300.3_Triticum_aestivumMAGGKKLHERVALGRTAWMLADFVILLLLL
ALVARRAASLGERGGTWLAALVCEAWFAFVWILNMNGKWSPVRFDTYPENLSHRLEELPAVDMFVTTADPALEPPLI
TVNTVLSLLALDYPDVGKLACYVSDDGCSPVTCYALREAAKFASLWIPFCKRYDVGVRAPFMYFSSAPEVGTGTADHEFL
ESWALMKTEYEKLASRIENADEVSILRDGGEEFAEFIDAERGNHPTIVKVLWDNSKSKAGEGFPHLVYLSREKSPRHRHN
FKAGAMNVLTRVSAVMTNAPIMLNVDCDMFANNPQVALHAMCLLLGFDDEIHSGFVQAPQKFYGGLKDDPFGNQ
MQVITKKIGGGLAGIQGTFYGGTGCFHRRKVIYGMPPPDTVKHETRGSPSYKELQAKFGSSKELIESSRNIISGDLLARPT
VDISSRVEMAKQVGDCNYEAGTCWGQEIGWVYGSMTEDILTGQRIQAAGWESALLDTDPPAFLGCAPTGGPASLTQ
FKRWATGLLEILISRNSPILGTIFKGLQLRQCLGYLIVDAWPVRAPFELCYALLGPFCLLTNQSFLPTASDEGFHIPAALFLTY
NIYHLMEYKECGLSVRAWWNNHRMQRITSASAWLLAFLTVILKTLGLSETVFEVTRKESSTSSDGGAGTDDADPGLFTF
DSAPVFIPVTALSVLNIVALTVAAWRAVVGTVAGVHGGPGVGEFVCCGWMVLCFWPFVRGLVSSGKYGIPWSVRVK
AGLIVAAFVHLCTRN

>CSLH_2DL_TGACv1_158387_AA0517170.1_Triticum_aestivumMAGGKKLQERVALGRTAWMLADFVILLLLL
ALVARRAASLGERGGTWLAALVCEAWFAFVWILNMNGKWSPVRFDTYPDNLSHRMEELPAVDMFVTTADPALEPPL

ITVNTVLSLLALDYPDVGRLACYVSDDGCSPVTCYALREAAKFAGLWVPFCKRHDVGVRAPFMYFSSAPEVGNGTVDH
EFLESWALMKSQYEKLARRIENADEGTIMRDGGDEFAEFIDAERGNHPTIVKVLWDNSKSKAGEEFPHLVYLSREKSPR
HRHNFKAGAMNVLTRVSAVMTNAPIMLNVDCDMFANNPQVALHAMCLLLGFDDEIHSGFVQAPQKFYGGLKDDPF
GNQMQVITKKIGGGLAGIQGMFYGGTGCFHRRKVIYGVPPPDTVKHEMKGSPSYKELQAKFGSSKELIESSRNIISGDLL
ARPTVDLSSRVEMAKQVGDCNYEAGTCWGQEIGWVYGSMTEDILTGLRIHAAGWESALLDTEPPAFLGCAPTGGPAS
LTQFKRWATGLLEILISQNSPILGTIFRRLQLRQCLAYLIVEAWPVRAPFELCYALLGPFCLLTNQSFLPTASDEGFRIPAALF
LTCHIYHLMEYKECGLSVRAWWNNHRMQRITSASAWLLAFLTVILKTLGLSETVFEVTRKESSTSSDGGAGTDEADPGL
FTFDSAPVFIPVTVLSMLNIVALAVAAWRAVVGAAAGVHGGPGIGEFVCCGWIVLCFWPFVRGLVSRGKYGIPWSVRV
KAGLIVAAFVHICTRN

>CSLH_2BL_TGACv1_129372_AA0380770.1_Triticum_aestivumMHRGEDSLSGLYKCTLAFVACGCGWSCGVV
LLASLLLLVASYLSATAMAGGKKLQERVALGRSAWMLADFVILFLVLALVARRAASLGERGGTWLAALVCEAWFAFVW
ILNMNGKWSPVRFDTYPENLSHRMEELPAVDMFVTTADPALEPPLITVNTVLSLLALDYPHVGKLACYVSDDGCSPLTC
YSLREAAKFASLWVPFCKRHDVGVRAPFMYFSSAPEVDTGTVDHEFLESWALMKSEYEKLASRIENADEVSILRDGGDE
FAEFIDAERGNHPTIVKVLWDNSKNKTGEGFPHLVYLSREKSPRHRHNFKAGAMNVLTRVSAVMTNAPIMLNVDCD
MFANNPQVALHAMCLLLGFDDEIHSGFVQAPQKFYGGLKDDPFGNQMQVITKKIGGGLAGIQGTFYGGTGCFHRRK
VIYGMPPPDTVKHETRGSPSYKELQAKFGSSKELIESSRNIISGDLLARPTVDISSRVEMAKQVGDCNYEAGTCWGQEIG
WVYGSMTEDILTGQRIQAAGWESALLDTDPPAFLGCAPTGGPASLTQFKRWATGLLEILISRNSPILGTIFRRLQLRQCL
AYLIVNAWPMRAPFEMCYALLGPFCLLTNQSFLPTTSNEGFRIPAALFLSYHVYHLMEYKECGLSVRAWWNNHRMQRI
TSASAWLLAFLTVILKTLGLSETVFEVTRKESSTSSDGGTGTDEADTGLFTFDSAPVFIPVTALSMLNIVALAVAAWRAVV
GTAAGVHGGPGVGEFVCCGWMVLCFWPFMRGLVSSGKYGIPWSVRVKAGLIVAAFVHLCTRN

>AT1G23480_CslA3MSPFLKFFLFLYDYLSPSSFFLVQRNTLGASLDTTDGVVRSGIIGEIIYIWKQTRIFVFIPILKCLVTIC
LVMSLLLFIERVYMSIVVVFVKLLRRTPEKVHKWEPINDDDLELANTNYPMVLIQIPMYNEKEVCQLSIGAACRLSWPLD
RMIVQVLDDSTDPASKELVNAECDKWARKGINIMSEIRDNRIGYKAGALKAGMMHNYVKQCEFVAIFDADFQPDPDF
LERTIPFLIHNHEISLVQCRWKFVNANECLMTRMQEMSLNYHFVAEQESGSSIHAFFGFNGTAGVWRIAALNEAGGW
KDRTTVEDMDLAVRACLHGWKFVYVHDVEVKNELPSTFKAYRFQQHRWSCGPANLWRKMTMEILQNKKVSAWKKL
YLIYNFFFIRKIVVHIFTFVFYCLILPTTVLFPELQVPKWATVYFPTTITILNAIATPRSLHLLVFWILFENVMSMHRTKATFIG
LLEAGRVNEWVVTEKLGDTLKSKLIGKATTKLYTRFGQRLNWRELVVGLYIFFCGCYDFAYGGSYFYVYLFLQSCAFFVAG
VGYIGTFVPTV

>AT1G24070_CslA10MTTFLKSLIFLQDSCLAFLSLMFHRGSSEDAAEALKKLETSINGARISFDTTWTREFRSLFIVPLFK
CLVAFCLIISLLVFIEGIYMNLVVLYVKVFERKPEKVYRWEAMQEDIELGHETYPMVLVQIPMYNEKEVLQLSIGAACRLI
WPLDRLIVQVLDDSTDQTIKELVNTECAKWESKGVNIKCERRDNRNGYKAGALKEGMKHNYVKLCNYVVIFDADFQPE
PDYLQHSVPFLVHNPEVALVQARWRFMNANKCLMTRMQEMSLNYHFMAEQESGSTRHAFFSFNGTAGVWRMAA
MEEAGGWHDRTTVEDMDLAVRAGLLGWKFVFLNDLTVKSELPSKFKAFRFQQHRWSCGPANLFRKMIMEIIRNKRV
TIWKKLYLVYSFFFLRKIIVHCFTFIFYCVILPTSVFFPEVNIPAWSTFYIPSMITLCIVIATPRSFYLVIFWILFENVMSMHRTK
GTFIGILERQRVNEWVVTEKLGDALKTKLLPRIGKPSNMFLERVNSKEIMVGIYILCCACYGLFFGNTLLYLYLFMQAVAFL
ISGVGFVGT

>AT2G35650_CslA7MSPLPIFHRLPHATFSSFLLSLSQAGSSKTSVAFLNAFKSEDIIARIGLWWQLIRAVVVVPVFKFLV
LLCLVMSVMFFVEVMYMGIVVLYVKLFKRKPEKFYKWEAMEDDVECGSASYPMVLVQIPMYNEKEVCEQSIAAACKIS
WPSNRIIIQVLDDSTDPASKELVKKECDRWSKEGVNITFEIRDNRNGYKAGALREGMRHSYVKQCDYVAIFDADFQPDP

DFLHRTVPFLIHNPKLALVQGRWEFVNAGQCMMTRLQEMSLSYHFTIEQQVGSSTFAFFGFNGTAGVWRISALNESG
GWNDQTTVEDMDLAVRATLRGWKFLYIDDLKVKSELPCSFKALRSQQHRWTCGPANLLRKMAGQIIRSENVSLWKK
WYMLYSFFFMRKIVAHILTFCFYCVILPATVLFPEVTVPKWAAFYLPSLITLLIAIGRLRSIHLLAFWVLFENAMSLLRAKAL
VMGLFETGRVQEWVVTEKLGDTLKTKLIPQVPNVRFRERVHLLELLVGAYLLFCGIYDIVYGKNTLYVYLLFQSVAFFVVG
FGFVGKYVPASSYLA

>AT3G56000_CslA14MATLSDGLFDDMSVLGVIGYVLEQTRFIFLVPILKRLVNLCQVVSVLLFVDAAYMAIVVAIVKLL
GRTPQKVLKWESFKSDDIELAPSSNHPMVLIQIPIFNEKEVCQLSIGAACKLSWPLDRMIIQVLDDSTEEESQKLVRLECK
KWESEGITIKSEVRGGFREGFKAGALTAGMKHSYVDEYKCEFVVIFDADFQPEPDFLERTVPFLVHNPEIALVQAGWKY
GNADECCMTRIQEMSLNYHFAVEQKSGSSILGFFGFNGTAGVWRIKALNEAEGWKDRTIVEDMDLAVRAYLRGSKFV
YVDDVKVKNELPSSFQAYRFQQHRWSCGPANLFKKIAMEIIKNQNVSLWKKVYLIYNFFFLRKIVVHIFTFVFYCVILPAT
VIFPEIEVPKWTTIYIPATITILNAIATPKSFYLILYWILFENVMAMHRSIGTLIGLLETSRVKEWIVTQKLGESNNLRENLIFP
DHYSFPERLRWREIMVGMYLFICGYYDFVFGRTYLYVYLFLQSIAFFVVGVGYVGMPVPSTPVQTSE

>AT4G13410_CslA15MFLLLKPLLSLHDLSLNLLSVMFHGETLKASVDGVGINMSTMWRETRNVFIVPLFKCIVVMCLII
SLLVFVESVYMNLVVLYVKLFNRKPEKVYKWEAMQEDMELGHQNYPMVLVQIPMYNEREVFELSIGAACRLTWPSDR
LIVQVLDDSTDPAIMELVSMECTKWASKDININYERRENRNGYKAGALKHGMRHSYVKQCQYLAIFDADFQPEPDYLQ
RAIPFLIHNPEVALVQARWRFVNANTCLMTRMQEMSLNYHFMAEQQSGSTRHAFFGFNGTAGVWRMVAMEEAG
GWKDRTTVEDMDLAVRVGLLGWKFIFVNDLEVKSELPSQFKAFRFQQHRWSCGPANLIRKMTMEIIHNKRVKIWKKF
YVIYSFFFLRKIVVHFFTYFFYCVILPTSVFLPEVNIPNWSTIYVPSVITLLSAIATPRSFYLVIFWVLFENVMAMHRTKGTLIG
LFEGGRVNEWVVTEKLGDTLNTKLLPQNGRLPKRVNLKEMMMGIYILCCACYDFAFGNAFLYLYLFMQATAFLISGVG
FVGT

>AT4G16590_CslA1MVDLVVQVVDDFTDPAVREGVDVEIAKWQSQGINIRCERRDNRNGYKAGAMKEALTQSYVK
QCDFVAVFDADFQPEPDYLIRAVPFLVHNPDVALVQARWIFVNANKCLMTRMQEMSLNYHFKVEQESGSTRHAFFG
FNGTAGVWRISAMEAAGGWKSRTTVEDMDLAVRVGLHGWKFVYLNDLTVRNELPSKFKAYRFQQHRWSCGPANLF
RKMTMEIIFNKRVSIWKKFYVIYSFFFVRKVAVHFLTFFFYCIIVPTSVFFPEIHIPSWSTIYVPSLISIFHTLATPRSFYLVIFW
VLFENVMAMHRTKGTCIGLLEGGRVNEWVVTEKLGDALKSKLLSRVVQRKSCYQRVNSKEVMVGVYILGCALYGLIYG
HTWLHFYLFLQATAFFVSGFGFVGT

>AT5G03760_CslA9MELGDTTSVIPDSFMGYRDDITMQMSMVLDQIRAPLIVPALRLGVYICLTMSVMLFVERVYMG
IVISLVKLFGRKPDKRFKYEPIKDDIELGNSAYPMVLIQIPMFNEREVYQLSIGAACGLSWPSDRIVIQVLDDSTDPTIKDLV
EMECSRWASKGVNIKYEIRDNRNGYKAGALKEGMKKSYVKSCDYVAIFDADFQPEADFLWRTVPYLLHNPKLALVQAR
WKFVNSDECLMTRMQEMSLDYHFTVEQEVGSSTYAFFGFNGTAGIWRISALNEAGGWKDRTTVEDMDLAVRASLK
GWKFLYLGSLKVKNELPSTFKAYRYQQHRWSCGPANLFRKMAFEIMTNKNVTLWKKVHVIYSFFVVRKLVAHIVTFIFY
CVILPATVLVPEVTVPKWGAVYIPSVITLLNAVGTPRSLHLMVFWILFENVMSLHRTKATFIGLLEGGRVNEWIVTEKLG
DVKAKSATKTSKKVIRFRFGDRIHVLELGVGMYLLFVGCYDAFFGKNHYYLYLFAQAIAFFIAGFGQIGTIVPNH

>AT5G16190_CslA11MQEDLELGNQNFPMVLVQIPMYNEREVFKLSIGAACRLIWPLDRLIVQVLDDSTDPTIMEMV
STECGKWATKGINIKCERRDNRNGYKAGALKQGMRHSYVKTCTYIAIFDADFQPEPDYLERTVPFLIHNPELALVQARW
KFVNAKKCLMTRMQEMSLNYHFTAEQESGSTRHAFFGFNGTAGVWRLAAMEEAGGWKDRTTVEDMDLAVRVGLH
GWKFVFVNDVSVKSELPSQFKAFRFQQHRWSCGPANLFRKMTMEIIRNKRVTIWKKLYVIYSFFFVRKIIVHFFTFFFYCF

ILPTSVFFPEVNIPTWSTVYFPFMITLFNAIATPRSFYLVIFWVLFENVMAMHRTKGTFIGLLEGGRVNEWVVTEKLGDA
LETKLLPQVRKPRNGFLERINSKEMMVGIYILCCASYNLVFGKTVLYIYLYMQALAFIIAGIGFIGT

>AT5G22740_CslA2MDGVSPKFVLPETFDGVRMEITGQLGMIWELVKAPVIVPLLQLAVYICLLMSVMLLCERVYMGI
VIVLVKLFWKKPDKRYKFEPIHDDEELGSSNFPVVLVQIPMFNEREVYKLSIGAACGLSWPSDRLVIQVLDDSTDPTVKQ
MVEVECQRWASKGINIRYQIRENRVGYKAGALKEGLKRSYVKHCEYVVIFDADFQPEPDFLRRSIPFLMHNPNIALVQA
RWRFVNSDECLLTRMQEMSLDYHFTVEQEVGSSTHAFFGFNGTAGIWRIAAINEAGGWKDRTTVEDMDLAVRASLR
GWKFLYLGDLQVKSELPSTFRAFRFQQHRWSCGPANLFRKMVMEIVRNKKVRFWKKVYVIYSFFFVRKIIAHWVTFCF
YCVVLPLTILVPEVKVPIWGSVYIPSIITILNSVGTPRSIHLLFYWILFENVMSLHRTKATLIGLFEAGRANEWVVTAKLGSG
QSAKGNTKGIKRFPRIFKLPDRLNTLELGFAAFLFVCGCYDFVHGKNNYFIYLFLQTMSFFISGLGWIGTYVPS

>AT2G32530_CslB1MADSSSSLPPLCEKISYKNYFLRVVDLTILGFLFSLLLYRILLMNQNNSVWVVAFLCESFFSFIWLLI
TSIKWSPASYKSYPERLDERVHDLPSVDMFVTTADPVREPPILVANTLLSLLAVNYPANKLACYVSDDGCSPLTYFSLKEA
SKFAKIWVPFCKKYNIKVRAPFRYFLNPPAATESSEFSKDWEITKREYEKLSRRVEDATGDSHWLDAEDDFEDFSNTKPN
DHSTIVKVVWENKGGVGVENEVPHFVYISREKRPNYLHHYKAGAMNFLVRVSGLMTNAPYMLNVDCDMYANEADV
VRQAMCIFLQKSMNSNHCAFVQFPQEFYDSNADELTVLQSYLGRGIAGIQGPTYAGSGCFHTRRVMYGLSIDDLEDDG
SLSSLATRKYLAEENLAREFGNSNEMVTSVVEALQRKPNPQNTLANSLEAAQEVGHCHFEYQTSWGKTIGWLYESTAE
DANTSIGIHSRGWTSSYISPKPPAFLGAMPPGGPEAMLQQRRWATGLLEVLFNKQSPLIGMFCRKIRFRQSLAYLYIFT
WGLRSIPELIYCLLPAYCLLHNAALFPKGVYLGIVVTLVGMHCLYSLWEFMSLGFSVQSWFASQSFWRIKTTCSWLFSIP
DIILKLLGISKTVFIVTKKTMPKTMSGSGSEKSQREVDCPNQDSGKFEFDGSLYFLPGTFILLVNLAALAGCSVGLQRHRG
GGSGLAEACGCILVVILFLPFLKGMFEKGKYGIPWSTLSKAAFLAVLFVVFSVGN

>AT2G32540_CslB2MAESSSPLPPLCERISHKSYFLRAVDLTILGLLLSLLLYRILHVNQKDTVWIVAFLCETCFTFVWLLIT
NIKWSPADYKTYPERLDERVHELPPVDMFVTTADPVREPPLIVVNTVLSLLAVNYPANKLACYVSDDGCSPLTYFSLKEA
SKFAKIWVPFCKKYNVRVRAPFMYFRNSPEAAEGSEFSKDWEMTKREYEKLSQKVEDATGSSHWLDAEDDFEAFLNTK
SNDHSTIVKVVWENKGGVGDEKEVPHVVYISREKRPNHFHHYKAGAMNFLVRVSGLMTNAPYMLNVDCDMYVNEA
DVVRQAMCIFLQKSMDSNHCAFVQYPQDFYDSNVGELTVLQLYLGRGIAGIQGPQYAGSGCFHTRRVMYGLSLDDLG
DDGSLSSIATRKYLAEESLTREFGNSKEMVKSVVDALQRKPFPQKNLKDSLETAQEMGHCHYEYQTSWGKNIGWLYDS
TTEDVNTSIGIHSRGWTSSYIFPDPPAFLGCMPQGGPEVMVQQRRWATGLLEILFNKQSPLIGMFCRKIRFRQSLAYLY
VFSWGLRSIPELFYCLLPAYCLLHNSALFPKGVYLGIIITLVGIHCLYTLWEFMNLGFSIQSWYVTQSFGRIKTTCSWLFSVL
DVILKLLGISKTVFIVTKKTMPETKSGSGSKKSQREVDCPNQDSGKFEFDGSLYFLPGTFIVLVNLAALAGCLVGLQSRGG
GGSGLAEACGCILVVILFLPFLKGMFEKGKYGIPFSTLSKAAFLAALFVVLSVGN

>AT2G32610_CslB3MADSSFSLPPLCERISYTNYFLRAVYLTVLGLFFSLLLHRIRHTSEYDNVWLVAFFCESCFFLVCLLIT
CLKWSPADTKPFPDRLDERVHDLPSVDMFVPTADPVREPPIMVVDTVLSLLAVNYPANKLACYVSDDGCSPLTYFSLKE
ASKFAKIWVPFCKKYNTRVRAPSRYFLKPISVATEDYEFNRDWEKTKREYEKLRRKVEDATGDSHMLDVEDDFEAFSNT
KPNDHSTLVKVVWENKGGVGDEKEIPHIIYISREKRPNYVHNQKCGAMNFLARVSGLMTNAPYILNVDCDMYANDAD
VVRQAMCILLQESLNMKHCAFVQFRQEFYDSSTELIVVLQSHLGRGIAGIQGPIYIGSGCVHTRRVMYGLSPDDFEVDG
SLSSVATREFLVKDSLARRFGNSKEMMKSVVDAIQRNPNPQNILTNSIEAAREVGHCQYEYQTSWGNTIGWLYDSVAE
DLNTSIGIHSRGWTSSYISPDTPAFLGSMPAGVPEALLQQRRWATGWIEILFNKQSPLRGLFSKKIRFRQRLAYLCIITCLR
SIPELIYCLLPAYCLLHNSTLFPKGLYLGITVTLVGIHCLYTLWEFMSLGYSVQSWLVSQSVWRIVATSSWLFSIFDITLKLLG
ISETVFIITKKTVAGTKSALGSGPSQGEDVGPNSDLFKFEFDGSLCFLPGTFIVLVNIAALAVFSVGLQRSSYSHEGGGSGL
AEACGCVLVMMLFLPFLMGLFKKGKYGTPLSTLSIAGFLAVLFVVFSV

>AT2G32620_CslB4MADSSCSLPPLYENISYKSYILRAVDLTILGLLFSLLLHRILYMSQNGIIWLVAFLCESCFSFVWLLST
CTKWSPAETKPYPDRLDERVYDLPSVDMFVPTADPVREPPIMVVNTVLSLLAVNYPANKLACYVSDDGCSPLTYFSLKE
ASKFAKIWVPFCKKYNLKVRAPFRYFLNPFAATEGSEFSRDWEMTKREYEKLCRKVEDATGDSHLLGTDNELEAFSNTK
PNDHSTIIKVVWENKGGVGDEKEVPHIVYISREKRPNYLHHYKAGAMNFLARVSGLMTNAPYMLNVDCDMYANEAD
VVRQAMCIFLQKSQNQNHCAFVQFPQEFYDSNTIKLTVIKSYMGRGIAGIQGPINVGSGCFHSRRVMYGLSPDELEDN
GSLSSVATRELLAEDSLSSGFGNSKEMVTSVVEALQRKPNPQNILTNSIEAAQEVGHCDYESQTSWGKTIGWLYDSMSE
DMNTSIGIHSRGWTSSYIAPDPPAFLGSMPPGGLEAMIQQRRWATGSIEVLFNKQSPLLGLFCRKLRFRQRVAYLCVSIC
VRSIPELIYCLLPAYCLLHNSALFPKGLCLGITMLLAGMHCLYTLWEFMCLGHSIQSWYVSQSFWRIVATSSWLFSIFDIIL
KLLGLSKNVFLVSKKTMPVETMSGSGIGPSQREDDGPNSGKTEFDGSLYFLPGTFIVLVNLAALVGVFVGLQRSSYSHGG
GGSGLGEACACILVVMLFFPFLKGLFAKGKYGIPLSTLSKAGFLAVSFVVFSVGN

>AT4G15290_CslB5MADSSSSLHPLCERISHKSYVLRAVDLTILGLLYSLLLYRILHISENDNVWLLAFFCESCFSLVWLIFT
CLKWSPAEDIPYINTLNERVHDLPSLDMFVPTADTVRESPIITVNTVLSLLAVNYPANKLACYVSDDGCSPLTYFSLKEASK
FVKIWAPFCKKYNVRVRAPFRYFLNPLVATDDSVFSKDWKMMKREYVKLCRKVEDATGDSHWLDADDDFEAFSNTKP
NDHSTIVKVVWENKGGVGDEKEVPHLVYISREKRPNYLHHYKTGAMNFLLRVSGLMTNAPYTLNVDCDMYANEPDV
VRQAMCVFLQNSKNSNHCAFVQFPQKFYDSYTNELAVLQSILGRGVAGIQGPFYIGTGCFHTRRVMYGLSSDDLEDNG
NISQVATREFLAEDSLVRKYGNSKELVKSVVDALQRKSNPQKSLANLIEAAQEVGHCHYEYQTSWGNLGWMYDSVAE
DINTSVGIHLRGWTSSFISPDPPAFIGSTPTLGLEAIVQQRRWATGAIEVLFNKQSPFMGMFHGKIKFRQRLAYFWALM
CLRSIPELIYCLLPAYCLLHDSALFPKGPCLCTIVTLVGMHCLYSLWQFMSLGFSVQSWYVVQSLWRIIATSSWLFSIQDIIL
KLLGISQIGFVIAKKTIPETKSVYESKPSQGEDDVPKLNLGKFEFDSSGLFIPGTFIMLVNLAALAGYLVRLQRSSCSHGGG
GSGLAEACGCILVVMLFLPFLKGLFEHGKYSIPLSTLSKAAFLTVLFVFFCVGK

>AT4G15320_CslB6MADSSSSLLPLCERISHKSYILRIVDLTILVLLFSLLWYRILHMCENNTIWLVAFLCESCFSFMWLIIT
CIKWSPAEDKPYPNRLDERVHDLPSVDMFVPTADPVREPPIIVVNTVLSLLAVNYPANKLACYVSDDGCSPLTYFSLKEA
SKFVKIWAPFCKKYNVRVRAPFRYFLNPLVATDDSVFSKDWKMMKIYKVFYYVYFCINMKREYVKLCRKVEDATGDSH
WLDADDDFEAFSNTKPNDHSTIVKVLLKLFLKTTVRVFVQFSKVMYILKLIIVVWENKGGVGDEKEVPHLVYISREKRPN
YLHHYKTGAMNFLVNDFYLTHLSFFDILIYLKINVNDCRAVSFCYYDKNMMSLIYNFKQLRVSGLMTNAPYMLNVDCD
MYANEPDVVRQAMCVFLQNSKNSNHCAFVQFPQNFYDSYTNELVVLQHYMKRGVAGIQGPIYIGSGCFHTRRVMYG
LSSDDLEDDGSLSSVASREFLSEDSLVRKYGSSKELVKSVVDALQRKSNPQKSLANLVEAAQEVGHCHYEYQTSWGNLG
WLYDSVAEDTNTSIGIHLRGWTSSFISPDPPAFLGSTPSVGPEAIVQHRRWATGSIEVLFNKQSPLIGFRRKIKFRQRLAYF
WVLMCIRSIPELVYCLLPAYCLLNNSALFPKGPCLGIIVTLVGMHCLYTLWQFMILGFSVKSCWLFSIQDIILKLLGISKIGFI
VAKKNMPETRSGYESKSKPSQGEDDGLKLELAGFLVRLQRSSYSHGGGGGSALAETCGCAMIVFGENDGSQEGLELPK
LNSKRGISSIVEKTFVACILPFVRNHKVNEKKNHHINIDRPNLL

>AT2G24630_CslC8MAPRFDFSDLWAKETRRGTPVVVKMENPNYSIVEVEEPDSAFQPMEKSRGKNAKQVTWVLLL
KAHKAVGCLTWVATVFWSLLGSVKRRLSFTHPLGSERLGRDGWLFSAIKLFLVASLAILAFELVAYYRGWHYFKNPNLHI
PTSKLEIQSLLHLFYVGWLSLRADYIAPPIKALSKFCIVLFLVQSVDRLILCLGCLWIKFKKIKPRIDEEHFRNDDFEGSGSEYP
MVLVQIPMCNEREVYEQSISAVCQLDWPKDRLLVQVLDDSDDESIQELIRDEVTKWSQKGVNIIYRHRLVRTGYKAGNL
KSAMSCDYVEAYEFVAIFDADFQPNSDFLKLTVPHFKEKPELGLVQARWAFVNKDENLLTRLQNINLCFHFEVEQQVN
GVFLNFFGFNGTAGVWRIKALEESGGWLERTTVEDMDIAVRAHLHGWKFIYLNDVKVLCEVPESYEAYKKQQHRWHS
GPMQLFRLCLRSILTSKIAMWKKANLILLFFLLRKLILPFYSFTLFCVILPITMFVPEAELPIWVICYVPIFMSLLNILPAPKSFP
FIVPYLLFENTMSVTKFNAMVSGLFQLGSSYEWIVTKKAGRSSESDLLALTDKESEKMPNQILRGVSDSELLEISQVEEQK
KQPVSVKKTNKIFHKELALAFLLLTAAVRSLLASQGVHFYFLLFQGLTFLLVGLDLIGEQMS

>AT3G07330_CslC6MSRSQNEEFQQWWNKQRDRNNHDVLYAGDDEAFLTVEIRTPATVDPDKDRIRTRTVRQLSRL
YLLKFKQLASSFLWIGNSFLYLVRTANRRIANDNPPSVSSSARLYRLIKGFLVVVVLLLCFELAAYFKGWHFTPPSVASAEV
AVEVVYAWWLEIRASYLAPPLQSLTNVCIVLFLIQSVDRLVLVLGCFWIKLRRIKPVASMEYPTKLVGEGVRLEDYPMVIV
QIPMCNEKEVYQQSIGAVCMLDWPRERMLVQVLDDSSELDVQQLIKAEVQKWQQRGVRIVYRHRLIRTGYKAGNLK
AAMNCEYVKDYEFVAIFDADFQPPADFLKKTVPHFKGNEELALVQTRWAFVNKDENLLTRLQNINLSFHFEVEQQVNG
VFINFFGFNGTAGVWRIKALEDCGGWLERTTVEDMDIAVRAHLCGWKFIYLNDVKCLCELPESYEAYKKQQYRWHSG
PMQLFRLCFFDILRSKVSAAKKANMIFLFFLLRKLILPFYSFTLFCVILPLTMFFPEANLPSWVVCYIPGIMSILNIIPAPRSFP
FIVPYLLFENTMSVTKFGAMISGLFKFDSSYEWVVTKKLGRSSEADLVAYAESGSLVESTTIQRSSSDSGLTELSKLGAAKK
AGKTKRNRLYRTEIALAFILLAASVRSLLSAQGIHFYFLLFQGITFVIVGLDLIGEQVS

>AT3G28180_CslC4MAPNSVAVTMEKPDNFSLLEINGSDPSSFPDKRKSISPKQFSWFLLLKAHRLISCLSWLVSSVKKR
IAFSAKNINEEEDPKSRGKQMYRFIKACLVISIIALSIEIVAHFKKWNLDLINRPSWEVYGLVEWSYMAWLSFRSDYIAPLV
ISLSRFCTVLFLIQSLDRLVLCLGCFWIKFKKIEPKLTEESIDLEDPSSFPMVLIQIPMCNEREVYEQSIGAASQLDWPKDRILI
QVLDDSDDPNLQLLIKEEVSVWAEKGVNIIYRHRLIRTGYKAGNLKSAMTCDYVKDYEFVTIFDADFTPNPDFLKKTVPH
FKGNPELGLVQARWSFVNKDENLLTRLQNINLCFHFEVEQQVNGVFLNFFGFNGTAGVWRIKALEESGGWLERTTVE
DMDIAVRAHLNGWKFIYLNDVEVTCELPESYEAYKKQQHRWHSGPMQLFRLCLPSIIKSKISVWKKANLIFLFFLLRKLIL
PFYSFTLFCIILPLTMFIPEAELPLWIICYVPIFISLLNILPSPKSFPFLVPYLLFENTMSITKFNAMISGLFQFGSAYEWVVTKK
TGRSSESDLLAFAEKEEKLHRRNSESGLELLSKLKEQETNLVGQETVKKSLGGLMRPKNKKKTNMVFKKELGLAFLLLTAA
ARSFLSAHGLHFYFLLFQGLSFLVVGLDLIGEQIS

>AT4G07960_CslC12MAPKFEWWAKGNNNNTRKGTPVVVKMENPNNWSMVELESPSHDDFLVRTHEKSRNKNA
RQLTWVLLLKAHRAAGCLTSLGSALFALGTAVRRRIAAGRTDIEISSSGVGSLQKQNHTKKSKLFYSCLKVFLWLSLILLGF
EIAAYFKGWSFGTSKLQLQFIFNKGFFDWVYTRWVLLRVEYLAPPLQFLANGCIVLFLVQSLDRLILCLGCFWIRFKKIKPV
PKPDSISDLESGDNGAFLPMVLVQIPMCNEKEVYQQSIAAVCNLDWPKGKILIQILDDSDDPITQSLIKEEVHKWQKLGA
RIVYRHRVNREGYKAGNLKSAMNCSYVKDYEFVAIFDADFQPLPDFLKKTIPHFKDNEEIGLVQARWSFVNKEENLLTRL
QNINLAFHFEVEQQVNSVFLNFFGFNGTAGVWRIKALEDSGGWLERTTVEDMDIAVRAHLHGWKFVFLNDVECQCEL
PESYEAYRKQQHRWHSGPMQLFRLCLPAVIKSKISIGKKFNLIFLFFLLRKLILPFYSFTLFCIILPMTMFVPEAELPAWVVC
YIPATMSFLNILPAPKSFPFIVPYLLFENTMSVTKFNAMVSGLFQLGSAYEWVVTKKSGRSSEGDLAALVEKDEKTTKHQ
RGVSAPETEAEKKAEKTKRKKKKHNRIYMKELSLAFLLLTAATRSLLSAQGIHFYFLLFQGISFLLVGLDLIGEQVE

>AT4G31590_CslC5MAPRLDFSDWWAKDTRKGTPVVVKMENPNYSVVEIDGPDSAFRPVEKSRGKNAKQVTWVLL
LKAHRAVGCLTWLATVFWSLLGAIKKRLSFTHPLGSEKLGRDRWLFTAIKLFLAVSLVILGFEIVAYFRGWHYFQSPSLHIP
TSTLEIQSLFHLVYVGWLTLRADYIAPPIKALSKFCIVLFLIQSVDRLVLCLGCFWIKYKKIKPRFDEEPFRNDDAEGSGSEYP
MVLVQIPMCNEREVYEQSISAVCQLDWPKDRILVQVLDDSNDESIQQLIKAEVAKWSQKGVNIIYRHRLVRTGYKAGN
LKSAMSCDYVEAYEYVAIFDADFQPTPDFLKLTVPHFKDNPELGLVQARWTFVNKDENLLTRLQNINLCFHFEVEQQVN
GVFLNFFGFNGTAGVWRIKALEESGGWLERTTVEDMDIAVRAHLHGWKFIYLNDVKVLCEVPESYEAYKKQQHRWHS
GPMQLFRLCLGSILTSKIAIWKKANLILLFFLLRKLILPFYSFTLFCIILPLTMFVPEAELPVWVICYIPVFMSFLNLLPSPKSFP
FIVPYLLFENTMSVTKFNAMVSGLFQLGSSYEWIVTKKAGRSSESDLLSITEKETPTKKSQLLRGVSDSELLELSQLEEQKQ
AVSKKPVKKTNKIYHKELALAFLLLTAALRSLLAAQGVHFYFLLFQGVTFLLVGLDLIGEQMS

>AT1G02730_CslD5MVKSAASQSPSPVTITVTPCKGSGDRSLGLTSPIPRASVITNQNSPLSSRATRRTSISSGNRRSNG
DEGRYCSMSVEDLTAETTNSECVLSYTVHIPPTPDHQTVFASQESEEDEMLKGNSNQKSFLSGTIFTGGFKSVTRGHVID
CSMDRADPEKKSGQICWLKGCDEKVVHGRCECGFRICRDCYFDCITSGGGNCPGCKEPYRDINDDPETEEEDEEDEAK

PLPQMGESKLDKRLSVVKSFKAQNQAGDFDHTRWLFETKGTYGYGNAVWPKDGYGIGSGGGGNGYETPPEFGERSK
RPLTRKVSVSAAIISPYRLLIALRLVALGLFLTWRVRHPNREAMWLWGMSTTCELWFALSWLLDQLPKLCPVNRLTDLG
VLKERFESPNLRNPKGRSDLPGIDVFVSTADPEKEPPLVTANTILSILAVDYPVEKLACYLSDDGGALLTFEALAQTASFAS
TWVPFCRKHNIEPRNPEAYFGQKRNFLKNKVRLDFVRERRRVKREYDEFKVRINSLPEAIRRRSDAYNVHEELRAKKKQ
MEMMMGNNPQETVIVPKATWMSDGSHWPGTWSSGETDNSRGDHAGIIQAMLAPPNAEPVYGAEADAENLIDTT
DVDIRLPMLVYVSREKRPGYDHNKKAGAMNALVRTSAIMSNGPFILNLDCDHYIYNSMALREGMCFMLDRGGDRICY
VQFPQRFEGIDPNDRYANHNTVFFDVSMRALDGLQGPMYVGTGCIFRRTALYGFSPPRATEHHGWLGRRKVKISLRR
PKAMMKKDDEVSLPINGEYNEEENDDGDIESLLLPKRFGNSNSFVASIPVAEYQGRLIQDLQGKGKNSRPAGSLAVPRE
PLDAATVAEAISVISCFYEDKTEWGKRVGWIYGSVTEDVVTGYRMHNRGWRSIYCVTKRDAFRGTAPINLTDRLHQVL
RWATGSVEIFFSRNNAIFATRRMKFLQRVAYFNVGMYPFTSLFLIVYCILPAISLFSGQFIVQSLDITFLIYLLSITLTLCMLSL
LEIKWSGITLHEWWRNEQFWVIGGTSAHPAAVLQGLLKVIAGVDISFTLTSKSSAPEDGDDEFADLYVVKWSFLMVPPL
TIMMVNMIAIAVGLARTLYSPFPQWSKLVGGVFFSFWVLCHLYPFAKGLMGRRGRVPTIVFVWSGLLSIIVSLLWVYIN
PPSGKQDYMQFQFP

>AT1G32180_CslD6MMDGESPLRHPRISHVSNSGSDFGSSSDYNKYLVQIPPTPDNNPGPASLSIVLLEIDSNQESVPS
VSGDIVSGSSGKDNEPDLTDVRINVGEEEEDDTLLSKISYSLTRVVKISPIIIALYRILIVVRVVSLALFLFWRIRNPNNKALW
LWLLSVICELWFAFSWLLDQIPKLFPVNHATDIEALKATFETPNPDNPTGKSDLPGIDVFVSTADAEKEPPLVTANTILSIL
SVDYPVEKLSVYISDDGGSLVTFEAIAEAASFAKIWVPFCRKHKIEPRNPESYFGLKRDPYKDKVRHDFVRERRYVKRAYD
EFKVRVNALPHSIRRRSDAFNSKEEIKALEKWKHWKVKVEEDQIKEPRPALVAPKATWMSDGTHWPGTWAVSGPHH
SRGDHASVIQVLLDPPGDEPVEGKGGEGRALDLEGVDIRLPMLVYVSREKRPGYDHNKKAGAMNALVRASAIMSNGP
FILNLDCDHYVYNSRAFRDGICFMMDHDGDRVSYVQFPQRFEGIDPSDRYANKNTVFFDINLRALDGIQGPMYVGTG
CLFRRTALYGFNPPDVFVVEEEPSGSYCFPLIKKRSPATVASEPEYYTDEEDRFDIGLIRKQFGSSSMLVNSVKVAEFEGRP
LATVHSSRLGRPPGSLTGSRKPLDFATVNEAVNVISCWYEDKTEWGFNVGWIYGSVTEDVVTGFRMHEKGWRSFYCV
TEPDAFRGSAPINLTDRLHQVLRWATGSVEIFFSRNNAIFAGPKLKLLQRIAYLNVGIYPFTSIFILTYCFLPPLSLFSGHFVV
ETLTGSFLIYLLIITLSLCGLAVLEVKWSGISLEEWWRNEQFWLIGGTSAHLVAVLQGILKVIAGVEISFTLTSKSSTGGDDE
DDEFADLYLFKWTALMIPPLTIIILNIVAILFAVCRTVFSANPQWSNLLGGTFFASWVLLHMYPFAKGLMGRGGKTPTVV
YVWSGLIAICLSLLYITIKNSEIDGGSFMLV

>AT2G33100_CslD1MASSPPKKTLNSQSSSLSRPPQAVKFGRRTSSGRIVSLSRDDDMDVSGDYSGQNDYINYTVLM
PPTPDNQPAGSSGSTSESKGDANRGGGGGDGPKMGNKLERRLSVMKSNNKSMLLRSQTGDFDHNRWLFESKGKYGI
GNAFWSEEDDTYDGGVSKSDFLDKPWKPLTRKVQIPAKILSPYRLLIVIRLVIVFFFLWWRITNPNEDAMWLWGLSIVC
EIWFAFSWILDILPKLNPINRATDLAALHDKFEQPSPSNPTGRSDLPGVDVFVSTADPEKEPPLVTANTLLSILAVDYPIEKL
SAYISDDGGAILTFEAMAEAVRFAEYWVPFCRKHDIEPRNPDSYFSIKKDPTKNKKRQDFVKDRRWIKREYDEFKVRING
LPEQIKKRAEQFNMREELKEKRIAREKNGGVLPPDGVEVVKATWMADGTHWPGTWFEPKPDHSKGDHAGILQIMSK
VPDLEPVMGGPNEGALDFTGIDIRVPMFAYVSREKRPGFDHNKKAGAMNGMVRASAILSNGAFILNLDCDHYIYNSK
AIKEGMCFMMDRGGDRICYIQFPQRFEGIDPSDRYANHNTVFFDGNMRALDGLQGPVYVGTGCMFRRYALYGFNPP
RANEYSGVFGQEKAPAMHVRTQSQASQTSQASDLESDTQPLNDDPDLGLPKKFGNSTMFTDTIPVAEYQGRPLADH
MSVKNGRPPGALLLPRPPLDAPTVAEAIAVISCWYEDNTEWGDRIGWIYGSVTEDVVTGYRMHNRGWRSVYCITKRD
AFRGTAPINLTDRLHQVLRWATGSVEIFFSKNNAMFATRRLKFLQRVAYLNVGIYPFTSIFLVVYCFLPALCLFSGKFIVQS
LDIHFLSYLLCITVTLTLISLLEVKWSGIGLEEWWRNEQFWLIGGTSAHLAAVVQGLLKVIAGIEISFTLTSKASGEDEDDIF
ADLYIVKWTGLFIMPLTIIIVNLVAIVIGASRTIYSVIPQWGKLMGGIFFSLWVLTHMYPFAKGLMGRRGKVPTIVYVWS
GLVSITVSLLWITISPPDDVSGSGGISV

>AT3G03050_CslD3MASNNHFMNSRSNLSTNSDAAEAERHQQPVSNSVTFARRTPSGRYVNYSRDDLDSELGSVDL
TGYSVHIPPTPDNQPMDPSISQKVEEQYVSNSLFTGGFNSVTRAHLMEKVIDTETSHPQMAGAKGSSCAVPGCDVKV
MSDERGQDLLPCECDFKICRDCFMDAVKTGGMCPGCKEPYRNTDLADFADNNKQQRPMLPPPAGGSKMDRRLSLM
KSTKSGLMRSQTGDFDHNRWLFETSGTYGFGNAFWTKDGNFGSDKDGNGHGMGPQDLMSRPWRPLTRKLQIPAA
VISPYRLLILIRIVVLALFLMWRIKHKNPDAIWLWGMSVVCELWFALSWLLDQLPKLCPINRATDLNVLKEKFETPTPSNP
TGKSDLPGLDMFVSTADPEKEPPLVTSNTILSILAADYPVEKLACYVSDDGGALLTFEAMAEAASFANMWVPFCRKHNI
EPRNPDSYFSLKRDPYKNKVKADFVKDRRRVKREYDEFKVRINSLPDSIRRRSDAYHAREEIKAMKLQRQNRDEEIVEPV
KIPKATWMADGTHWPGTWINSGPDHSRSDHAGIIQVMLKPPSDEPLHGVSEGFLDLTDVDIRLPLLVYVSREKRPGYD
HNKKAGAMNALVRASAIMSNGPFILNLDCDHYIYNSQALREGMCFMMDRGGDRLCYVQFPQRFEGIDPSDRYANHN
TVFFDVNMRALDGLMGPVYVGTGCLFRRIALYGFDPPRAKEHHPGFCSCCFSRKKKKSRVPEENRSLRMGGDSDDDEE
MNLSLVPKKFGNSTFLIDSIPVAEFQGRPLADHPAVQNGRPPGALTIPRELLDASTVAEAIAVISCWYEDKTEWGSRIGW
IYGSVTEDVVTGYRMHNRGWKSVYCVTKRDAFRGTAPINLTDRLHQVLRWATGSVEIFFSRNNAFFASPRMKILQRIAY
LNVGIYPFTSFFLIVYCFLPALSLFSGQFIVQTLNVTFLVYLLIISITLCLLALLEIKWSGISLEEWWRNEQFWLIGGTSAHLAA
VIQGLLKVVAGIEISFTLTSKSGGEDVDDEFADLYIVKWTSLMIPPITIMMVNLIAIAVGFSRTIYSVIPQWSKLIGGVFFSF
WVLAHLYPFAKGLMGRRGRTPTIVYVWSGLVAITISLLWVAINPPAGSTQIGGSFTFP

>AT4G38190_CslD4MASTPPQTSKKVRNNSGSGQTVKFARRTSSGRYVSLSRDNIELSGELSGDYSNYTVHIPPTPDN
QPMATKAEEQYVSNSLFTGGFNSVTRAHLMDKVIDSDVTHPQMAGAKGSSCAMPACDGNVMKDERGKDVMPCEC
RFKICRDCFMDAQKETGLCPGCKEQYKIGDLDDDTPDYSSGALPLPAPGKDQRGNNNNMSMMKRNQNGEFDHNR
WLFETQGTYGYGNAYWPQDEMYGDDMDEGMRGGMVETADKPWRPLSRRIPIPAAIISPYRLLIVIRFVVLCFFLTWRI
RNPNEDAIWLWLMSIICELWFGFSWILDQIPKLCPINRSTDLEVLRDKFDMPSPSNPTGRSDLPGIDLFVSTADPEKEPPL
VTANTILSILAVDYPVEKVSCYLSDDGGALLSFEAMAEAASFADLWVPFCRKHNIEPRNPDSYFSLKIDPTKNKSRIDFVK
DRRKIKREYDEFKVRINGLPDSIRRRSDAFNAREEMKALKQMRESGGDPTEPVKVPKATWMADGTHWPGTWAASTR
EHSKGDHAGILQVMLKPPSSDPLIGNSDDKVIDFSDTDTRLPMFVYVSREKRPGYDHNKKAGAMNALVRASAILSNGP
FILNLDCDHYIYNCKAVREGMCFMMDRGGEDICYIQFPQRFEGIDPSDRYANNNTVFFDGNMRALDGVQGPVYVGT
GTMFRRFALYGFDPPNPDKLLEKKESETEALTTSDFDPDLDVTQLPKRFGNSTLLAESIPIAEFQGRPLADHPAVKYGRPP
GALRVPRDPLDATTVAESVSVISCWYEDKTEWGDRVGWIYGSVTEDVVTGYRMHNRGWRSVYCITKRDSFRGSAPIN
LTDRLHQVLRWATGSVEIFFSRNNAILASKRLKFLQRLAYLNVGIYPFTSLFLILYCFLPAFSLFSGQFIVRTLSISFLVYLLMIT
ICLIGLAVLEVKWSGIGLEEWWRNEQWWLISGTSSHLYAVVQGVLKVIAGIEISFTLTTKSGGDDNEDIYADLYIVKWSSL
MIPPIVIAMVNIIAIVVAFIRTIYQAVPQWSKLIGGAFFSFWVLAHLYPFAKGLMGRRGKTPTIVFVWAGLIAITISLLWTA
INPNTGPAAAAEGVGGGGFQFP

>AT1G55850_CslE1MVNKDDRIRPVHEADGEPLFETRRRTGRVIAYRFFSASVFVCICLIWFYRIGEIGDNRTVLDRLIW
FVMFIVEIWFGLYWVVTQSSRWNPVWRFPFSDRLSRRYGSDLPRLDVFVCTADPVIEPPLLVVNTVLSVTALDYPPEKL
AVYLSDDGGSELTFYALTEAAEFAKTWVPFCKKFNVEPTSPAAYLSSKANCLDSAAEEVAKLYREMAARIETAARLGRIPE
EARVKYGDGFSQWDADATRRNHGTILQVLVDGREGNTIAIPTLVYLSREKRPQHHHNFKAGAMNALLRVSSKITCGKII
LNLDCDMYANNSKSTRDALCILLDEKEGKEIAFVQFPQCFDNVTRNDLYGSMMRVGIDVEFLGLDGNGGPLYIGTGCF
HRRDVICGRKYGEEEEEEEESERIHENLEPEMIKALASCTYEENTQWGKEMGVKYGCPVEDVITGLTIQCRGWKSAYLNP
EKQAFLGVAPTNLHQMLVQQRRWSEGDFQIMLSKYSPVWYGKGKISLGLILGYCCYCLWAPSSLPVLIYSVLTSLCLFKG
IPLFPKVSSSWFIPFGYVTVAATAYSLAEFLWCGGTFRGWWNEQRMWLYRRTSSFLFGFMDTIKKLLGVSESAFVITAK
VAEEEAAERYKEEVMEFGVESPMFLVLGTLGMLNLFCFAAAVARLVSGDGGDLKTMGMQFVITGVLVVINWPLYKG
MLLRQDKGKMPMSVTVKSVVLALSACTCLAFL

>AT4G23990_CslG1MEPHRKHSVGDTTLHTCHPCRRTIPYRIYAVFHTCGIIALMYHHVHSLLTANTTLITSLLLLSDIVL
AFMWATTTSLRYKPVRRTEYPEKYAAEPEDFPKLDVFICTADPYKEPPMMVVNTALSVMAYEYPSDKISVYVSDDGGSS
LTLFALMEAAKFSKHWLPFCKKNNVQDRSPEVYFSSKLRSRSDEAENIKMMYEDMKSRVEHVVESGKVETAFITCDQF
RGVFDLWTDKFTRHDHPTIIQVLQNSENDMDDTKKYIMPNLIYVSREKSKVSSHHFKAGALNTLLRVSGVMTNSPIILTL
DCDMYSNDPATPVRALCYLTDPKIKTGLGFVQFPQTFQGISKNDIYACAYKRLFEINMIGFDGLMGPNHVGTGCFFNRR
GFYGAPSNLILPEIDELKPNRIVDKPINAQDVLALAHRVAGCIYELNTNWGSKIGFRYGSLVEDYYTGYRLHCEGWRSVFC
RPKRAAFCGDSPKSLIDVVSQQKRWAIGLLEVAISRYSPITYGVKSMGLVTGVGYCQYACWAFWSLPLIVYGFLPQLALL
YQSSVFPKSSDPWFWLYIVLFLGAYGQDLLDFVLEGGTYGGWWNDQRMWSIRGFSSHLFGFIEFTLKTLNLSTHGFNV
TSKANDDEEQSKRYEKEIFEFGPSSSMFLPLTTVAIVNLLAFVWGLYGLFAWGEGLVLELMLASFAVVNCLPIYEAMVLRI
DDGKLPKRVCFVAGILTFVLIVSGYVFLK

>AT4G24000_CslG2MEPQRKHSTALHTCHPCRRTIPYRIYAVFHTCGIIALMYHHVHSIVNANNTLITCLLLLSDIVLAF
MWATTTSLRLNPIHRTEYPEKYAAKPEDFPKLDVFICTADPYKEPPMMVVNTALSVMAYEYPSHKISVYVSDDGGSSLT
LFALMEAAKFSKHWLPFCKNNNVQDRSPEVYFSSKSHSSSDEAENLKMMYEDMKSRVEHVVESGKVETAFIACDQFSC
VFDLWTDKFTRHDHPTIIMVLQHNETEMMPNLIYVSREKSKVSPHHFKAGALNTLLRVSAVMTNSPIILTLDCDMYSN
NPTTPLHALCYLSDPKINFDLGFVQFPQKFQGVNKNDIYASELKRPFDINTVGFDGLMGPVHMGTGCFFNRRAFYGPP
TTLILPEIETFGPNRIADKPIKAQDILALAHDVAGCNYECNTNWGSKIGFRYGSLVEDYFTGFMLHCEGWRSIFCSPTKAA
FYGDSPKCLTDVIGQQIRWSVGLLEVAFSRYNPLTYGIKPLSLLMSLGYCHYAFWPFWCIPLVVYGILPQVALIHGVSVFP
KASDPWFWLYIILFLGGYAQDLSDFLLEGGTYRKWWNDQRMWMVRGLSSFFFGFTEFTLKTLNLSTQGYNVTSKSND
DNEQMKRYEQEIFDFGPSSSMFLPITTVAIMNLLAFMRGLYGIFTWGEGPVLELMLASFAVVNCLPIYEAMVLRIDDGK
LPKRICFLAGLLSFVLTGSGYFFLK

>AT4G24010_CslG3METHRKNSVVGNILHTCHPCRRTIPYRIYAIFHTCGIIALMYHHVHSLVTANNTLITCLLLLSDIVL
AFMWATTTSLRLNPVHRTECPEKYAAKPEDFPKLDVFICTADPYKEPPMMVVNTALSVMAYEYPSDKISVYVSDDGGS
SLTFFALIEAAKFSKQWLPFCKKNNVQDRSPEVYFSSESHSRSDEAENLKTNILKCEVEQMMYEDMKSRVEHVVESGKV
ETAFITCDQFRGVFDLWTDKFSRHDHPTIIQVLQNSETDMDNTRKYIMPNLIYVSREKSKVSPHHFKAGALNTLLRVSGV
MTNSPIILTLDCDMYSNDPATLVRALCYLTDPEIKSGLGYVQFPQKFLGISKNDIYACENKRLFIINMVGFDGLMGPTHV
GTGCFFNRRAFYGPPYMLILPEINELKPYRIADKSIKAQDVLSLAHNVAGCIYEYNTNWGSKIGFRYGSLVEDYYTGFMLH
CEGWRSVFCNPKKAAFYGDSPKCLVDLVGQQIRWAVGLFEMSFSKYSPITYGIKSLDLLMGLGYCNSPFKPFWSIPLTVY
GLLPQLALISGVSVFPKASDPWFWLYIILFFGAYAQDLSDFLLEGGTYRKWWNDQRMLMIKGLSSFFFGFIEFILKTLNLS
TPKFNVTSKANDDDEQRKRYEQEIFDFGTSSSMFLPLTTVAIVNLLAFVWGLYGILFCGGELYLELMLVSFAVVNCLPIYG
AMVLRKDDGKLSKRTCFLAGNLHVGSYCVKLLRPQVTSPLRLIHNNNTSGWFKRKKHNMNESV

>OS02G09930_OsCslA1MEVNGGGAAGLPEAWSQVRAPVIVPLLRLAVAVCLTMSVLLFLERMYMAVVISGVKILRR
RPDRRYRCDPIPDDDPELGTSAFPVVLIQIPMFNEREVYQLSIGAVCGLSWPSDRLVVQVLDDSTDPVIKEMVRIECER
WAHKGVNITYQIRENRKGYKAGALKEGMKHGYVRECEYVAIFDADFQPDPDFLRRTIPFLVHNSDIALVQARWRFVNA
DECLMTRMQEMSLDYHFTVEQEVSSSVCAFFGFNGTAGVWRVSAVNEAGGWKDRTTVEDMDLAIRASLKGWKFVY
LGDVQVKSELPSTFKAFRFQQHRWSCGPANLFRKMLMEIKVTIWKKIHVIYNFFLIRKIIAHIVTFAFYCLIIPATIFVPEVRI
PKWGCVYIPTIITLLNSVGTPRSFHLLFFWILFENVMSLHRTKATLIGLLEAGRANEWVVTEKLGNALKMKSSSKSSAKKS
FMRVWDRLNVTELGVAAFLFSCGWYDLAFGKDHFFIYLFFQGAAFFIVGIGYVGTIVPQS

>DAA01745_OsCslA10MEGQWGRWRLAAAAAASSSGDQIAAAWAVVRARAVAPVLQFAVWACMAMSVMLVLE
VAYMSLVSLVAVKLLRRVPERRYKWEPITTGSGGVGGGDGEDEEAATGGREAAAFPMVLVQIPMYNEKEVYKLSIGAA

CALTWPPDRIIIQVLDDSTDPAIKDLVELECKDWARKEINIKYEIRDNRKGYKAGALKKGMEHIYTQQCDFVAIFDADFQ
PESDFLLKTIPFLVHNPKIGLVQTRWEFVNYDVCLMTRIQKMSLDYHFKVEQESGSSMHSFFGFNGKIQYQNYLHELFM
PGPNIADLPTGTAGVWRVSAINEAGGWKDRTTVEDMDLAVRASLKGWQFLYVGDIRVKSELPSTFKAYRHQQHRWT
CGAANLFRKMXQRKLPKTRQAFFNXTEWNMLRDCHAIXFFTRLYVIQGVSVWKKLHLLYSFFFVRRVVAPILTFLFYCVV
IPLSVMVPEVSIPVWGMVYIPTAITIMNAIRNPGSIHLMPFWILFENVMAMHRMRAALTGLLETMNVNQWVVTEKV
GDHVKDKLEVPLLEPLKPTDCVERIYIPELMVAFYLLVCASYDLVLGAKHYYLYIYLQAFAFIALGFGFAGTSTPCS

>OS08G33740_OsCslA11MSSSGGGGVAEEVARLWGELPVRVVWAAVAAQWAAAAAAARAAVVVPAVRALVAVS
LAMTVMILAEKLFVAAVCLAVRAFRLRPDRRYKWLPIGAAAAAASSEDDEESGLVAAAAAFPMVLVQIPMFNEREVYK
LSIGAACSLDWPSDRVVIQVLDDSTDLVVKVFIVIYFTDISSRIIRSTSSLVIKDLVEKECQKWQGKGVNIKYEVRGNRKGY
KAGALKEGLKHDYVKECEYIAMFDADFQPESDFLLRTVPFLVHNSEIALVQTRWKFVNANECLLTRFQEMSLDYHFKYE
QEAGSSVYSFFGFNGTAGVWRIAAIDDAGGWKDRTTVEDMDLAVRATLQGWKFVYVGDVKVKSELPSTFKAYRFQQ
HRWSCGPANLFKKMMVEILENKKVSFWNKIHLWYDFFFVGKIAAHTVTFIYYCFVIPVSVWLPEIEIPLWGVVYVPTVIT
LCKAVGTPSSFHLVILWVLFENVMSLHRIKAAVTGILEAGRVNEWVVTEKLGDANKTKPDTNGSDAVKVIDVELTTPLIP
KLKKRRTRFWDKYHYSEIFVGICIILSGFYDVLYAKKGYYIFLFIQGLAFLIVGFDYIGVCPP

>OS10G26630_OsCslA2MSAMLVAEAAWMGLASLAAAAARRLRGYGYRWEPMAAPPDVEAPAPAPAEFPMVLV
QIPMYNEKEVYKLSIGAACALTWPPDRIIIQVLDDSTDPFVKFSLVQELVELECKEWASKKINIKYEVRNNRKGYKAGALR
KGMEHTYAQLCDFVAIFDADFEPESDFLLKTMPYLLHNPKIALVQTRWEFVNYNVCLMTRIQKMSLDYHFKVEQESGS
FMHAFFGFNGTAGVWRVSAINQSGGWKDRTTVEDMDLAVRASLKGWEFLYVGDIRVKSELPSTFQAYRHQQHRWT
CGAANLFRKMAWEIITNKEVSMWKKYHLLYSFFFVRRAIAPILTFLFYCIVIPLSAMVPEVTIPVWGLVYIPTAITIMNAIR
NPGSVHLMPFWILFENVMAMHRMRAALSGLLETARANDWVVTEKVGDQVKDELDVPLLEPLKPTECAERIYIPELLLA
LYLLICASYDFVLGNHKYYIYIYLQAVAFTVMGFGFVGTRTPCS

>OS06G12460_OsCslA3MAMAGADGPTAGAAAAVRWRGGESLLLLLLRWPSSAELVAAWGAARASAVAPALAAAS
AACLALSAMLLADAVLMAAACFARRRPDRRYRATPLGAGAGADDDDDDEEAGRVAYPMVLVQIPMYNEREVYKLSIG
AACGLSWPSDRLIVQVLDDSTDPTVKTWYDRLRKTLVQQAHPAQADMDVHQSTKRKNKELMTRVPILECDSNHGLAS
IISSYLIAVGLVELECKSWGNKGKNVKYEVRNTRKGYKAGALKEGLLRDYVQQCNYVAIFDADFQPEPDFLLRTIPYLVRN
PQIGLVQAHWEFGTAGVWRISALEEAGGWKDRTTVEDMDLAVRAGLKGWKFVYLADVKVKSELPSNLKTYRHQQHR
WTCGAANLFRKVGAEILFTKEVPFWWKFYLLYSFFFVRKVVAHVVPFMLYCVVIPFSVLIPEVTVPVWGVVYVPTTITLL
HAIRNTSSIHFIPFWILFENVMSFHRTKAMFIGLLELGGVNEWVVTEKLGNGSNTKPASQILERPPCRFWDRWTMSEIL
FSIFLFFCATYNLAYGGDYYFVYIYLQAIAFLVVGIGFCGTISSNS

>OS03G07350_OsCslA4MEGQWGRWRLAAAAAASSSGDQIAAAWAVVRARAVAPVLQFAVWACMAMSVMLVL
EVAYMSLVSLVAVKLLRRVPERRYKWEPITTGSGGVGGGDGEDEEAATGGREAAAFPMVLVQIPMYNEKEVYKLSIGA
ACALTWPPDRIIIQVLDDSTDPAIKDLVELECKDWARKEINIKYEIRDNRKGYKAGALKKGMEHIYTQQCDFVAIFDADF
QPESDFLLKTIPFLVHNPKIGLVQTRWEFVNYDVCLMTRIQKMSLDYHFKVEQESGSSMHSFFGFNGTAGVWRVSAIN
EAGGWKDRTTVEDMDLAVRASLKGWQFLYVGDIRVKSELPSTFKAYRHQQHRWTCGAANLFRKMATEIAKNKGVSV
WKKLHLLYSFFFVRRVVAPILTFLFYCVVIPLSVMVPEVSIPVWGMVYIPTAITIMNAIRNPGSIHLMPFWILFENVMAM
HRMRAALTGLLETMNVNQWVVTEKVGDHVKDKLEVPLLEPLKPTDCVERIYIPELMVAFYLLVCASYDLVLGAKHYYLYI
YLQAFAFIALGFGFAGTSTPCS

>OS03G26050_OsCslA5MEAGEAAGAVLFLLAAAVSLLAAVSTGALDFTYLVTVVGEGSSTSPGSGGGAWWREAWV
GARSRAVAPALQVGVWACMVMSVMLVVEATYNSAVSVAARLVGWRPERWFKWEPLGGGAGAGDEEKGEAAAAA
YPMVMVQIPMYNELEVYKLSIGAVCGLKWPKERLIIQVLDDSTDAFIKNLVELECEDWASKGLNIKYATRSGRKGFKAG
ALKKGMEWDYAKQCEYVAIFDADFQPEPDFLLRTVPFLMHNQNVALVQARWVFVNDRVSLLTRIQKTFLDYHFKAEQ
EAGSATFAFFSFNGTAGVWRTEAINDAGGWKDRTTVEDMDLAVRATLKGWKFIYLGDLRVKSELPSTYKAYCRQQFR
WSCGGANLFRKMIWDVLVAKKVSSLKKIYILYSFFLVRRVVAPAVAFILYNVIIPVSVMIPELFLPIWGVAYIPTALLIVTAIR
NPENLHTVPLWILFESVMSMHRLRAAVAGLLQLQEFNQWIVTKKVGNNAFDENNETPLLQKSRKRLINRVNLPEIGLS
VFLIFCASYNLVFHGKNSFYINLYLQGLAFFLLGLNCVGTLPDHCCF

>AAL25127_OsCslA6MQGSSTSILHFVPSDPTSTSVLDFLSPTPRGTSPVHDRRLHAGDLALRAGGDRLLVADTVAAVV
ESLVQAWRQVRMELLVPLLRGAVVACMVMSVIVLAEKVFLGVVSAVVKLLRRRPARLYRCDPVVVEDDDEAGRASFP
MVLVQIPMYNEKEVYQLSIGAACRLTWPADRLIVQVLDDSTDAIVKELVRKECERWGKKGINVKYETRKDRAGYKAGN
LREGMRRGYVQGCEFVAMLDADFQPPPDFLLKTVPFLVHNPRLALVQTRWEFVNANDCLLTRMQEMSMDYHFKVE
QEAGSSLCNFFGYNGTAGVWRRQVIDESGGWEDRTTAEDMDLALRAGLLGWEFVYVGSIKVKSELPSTLKAYRSQQH
RWSCGPALLFKKMFWEILAAKKVSFWKKLYMTYDFFIARRIISTFFTFFFFSVLLPMKVFFPEVQIPLWELILIPTAIILLHSV
GTPRSIHLIILWFLFENVMALHRLKATLIGFFEAGRANEWIVTQKLGNIQKLKSIVRVTKNCRFKDRFHCLELFIGGFLLTSA
CYDYLYRDDIFYIFLLSQSIIYFAIGFEFMGVSVSS

>OS07G43710_OsCslA7MVEAGEIGGAAVFALAAAAALSAASSLGAVDFRRPLAAVGGGGAFEWDGVVPWLIGVLG
GGDEAAAGGVSVGVAAWYEVWVRVRGGVIAPTLQVAVWVCMVMSVMLVVEATFNSAVSLGVKAIGWRPEWRFK
WEPLAGADEEKGRGEYPMVMVQIPMYNELEVYKLSIGAACELKWPKDKLIVQVLDDSTDPFIKNLVELECESWASKGV
NIKYVTRSSRKGFKAGALKKGMECDYTKQCEYIAIFDADFQPEPNFLLRTVPFLMHNPNVALVQARWAFVNDTTSLLTR
VQKMFFDYHFKVEQEAGSATFAFFSFNGTAGVWRTTAINEAGGWKDRTTVEDMDLAVRASLNGWKFIYVGDIRVKS
ELPSTYGAYCRQQFRWACGGANLFRKIAMDVLVAKDISLLKKFYMLYSFFLVRRVVAPMVACVLYNIIVPLSVMIPELFIP
IWGVAYIPMALLIITTIRNPRNLHIMPFWILFESVMTVLRMRAALTGLMELSGFNKWTVTKKIGSSVEDTQVPLLPKTRK
RLRDRINLPEIGFSVFLIFCASYNLIFHGKTSYYFNLYLQGLAFLLLGFNFTGNFACCQ

>Os09g39920_
OsCslA8MSIMLFLERLYMALVVAALWLIRRRRRRSNRREQDDDGAENDQLLQDPEAANSPMVLVQIPMFNEKQVYR
LSIGAACGMTWPSDKLVIQVLDDSTDPAIREMVEGECGRWAGKGVSIRYENRRNRSGYKAGAMREGLRKAYARECEL
VAIFDADFQPDADFLLRTVPVLVADPGVALVQARWRFVNADECLLTRIQEMSLDYHFRVEQEVGSACHGFFGFNGTA
GVWRVRALEEAGGWKERTTVEDMDLALVAHLLTFSFYCVVIPACVLAGSDHVRLPKYVALYVPAAITLLNAACTPRSCH
LLIFWILFENVMSMHRTKATLIGLLEATRANEWVVTDKRGNANPKHQQPANTTTRPGRKTTTSSSRTSFFNNDVHVAEI
LLGACLLYCALYDIAYGRDSFYIYLLLQSAAAFIVGFGYVGT

>OS06G42020_OsCslA9MWEQVKAPVVVPLLRLSVAACLAMSVMLFVEKVYMSVVLVGVHLFGRRPDRRYRCDPIV
AAGADNDDPELADANAAFPMVLIQIPMYNEREVYKLSIGAACGLSWPSDRVIVQVLDDSTDPVIKEMVQVECKRWES
KGVRIKYEIRDNRVGYKAGALREGMKHGYVRDCDYVAIFDADFQPDPDFLARTIPFLVHNPDIALVQARWKFVNANEC
LMTRMQEMSLDYHFKVEQEVGSSTHAFFGFNGTAGVWRISAMNEAGGWKDRTTVEDMDLAVRAGLKGWKFVYLG
DLMVKSELPSTFKAFRYQQHRWSCGPANLFRKMLVEIATNKKVTLWKKIYVIYNFFLVRKIIGHIVTFVFYCLVVPATVLIP
EVEIPRWGYVYLPSIVTILNSIGTPRSLHLLIFWVLFENVMSLHRTKATLIGLLETGRVNEWVVTEKLGDALKLKLPGKAFR
RPRMRIGDRVNALELGFSAYLSFCGCYDIAYGKGYYSLFLFLQSITFFIIGVGYVGTIVPH

>OS01G56130_OsCslC1MARWWGGEGRGGSGTPVVVKMESPEWAISEVEAGAAAPGSPAAGGKAGRGKNARQIT
WVLLLKAHRAAGKLTGAASAALSVAAAARRRVAAGRTDSDDAAAAPPGESPALRARFHGFLRAFLLLSVLLLAVDVAA
HAQGWHAVVPDLLAVEGLFAAAYASWLRVRLEYLAPGLQFLANACVVLFLIQSADRLILCLGCLWIKLKGIKPVPKASGG
GGGGKGSDDVEAGADEFPMVLVQIPMCNEKEVYQQSIGAVCNLDWPRSNFLVQVLDDSDDAATSALIKEEVEKWQR
EGVRILYRHRVIRDGYKAGNLKSAMNCSYVKDYEFVVIFDADFQPQADFLKRTVPHFKGNEDVGLVQARWSFVNKDE
NLLTRLQNINLCFHFEVEQQVNGVFLNFFGFNGTAGVWRIKALEDSGGWMERTTVEDMDIAVRAHLKGWKFLYINDV
ECQCELPESYEAYRKQQHRWHSGPMQLFRLCFVDIIKSKIGVWKKFNLIFLFFLLRKLILPFYSFTLFCIILPMTMFVPEAEL
PAWVVCYIPATMSLLNILPAPKSFPFIVPYLLFENTMSVTKFNAMISGLFQLGSAYEWVVTKKSGRSSEGDLVSLVEKQP
KQQRVGSAPNLDSLAKESHPKKDSKKKKHNRIYQKELALSFLLLTAAARSLLSVQGIHFYFLLFQGVSFLVVGLDLIGEQVE

>OS07G03260_OsCslC10MAPWSGFWAASRPALAAAAAGGTPVVVKMDNPNWSISEIDADGGEFLAGGRRRGRG
KNAKQITWVLLLKAHRAAGCLAWLASAAVALGAAARRRVAAGRTDDADAETPAPRSRLYAFIRASLLLSVFLLAVELAA
HANGRGRVLAASVDSFHSSWVRFRAAYVAPPLQLLADACVVLFLVQSADRLVQCLGCLYIHLNRIKPKPISSPAAAAAAL
PDLEDPDAGDYYPMVLVQIPMCNEKEVYQQSIAAVCNLDWPRSNILVQVLDDSDDPITQSLIKEEVEKWRQNGARIVY
RHRVLREGYKAGNLKSAMSCSYVKDYEYVAIFDADFQPYPDFLKRTVPHFKDNEELGLVQARWSFVNKDENLLTRLQNI
NLCFHFEVEQQVNGIFINFFGFNGTAGVWRIKALEDSGGWMERTTVEDMDIAVRAHLNGWKFVFLNDVECQCELPES
YEAYRKQQHRWHSGPMQLFRLCLPDIIRCKIAFWKKANLIFLFFLLRKLILPFYSFTLFCIILPMTMFIPEAELPDWVVCYIP
ALMSFLNILPAPKSFPFIIPYLLFENTMSVTKFNAMISGLFQLGSAYEWVVTKKSGRSSEGDLIALAPKELKQQKILDLTAIK
EQSMLKQSSPRNEAKKKYNRIYKKELALSLLLLTAAARSLLSKQGIHFYFLMFQGLSFLLVGLDLIGEDVK

>OS09G25900_OsCSLC2MAPPGVGVGVAYLWGKGRGGRKGTPVVVTMESPNYSVVEVDGPDAEAELRTAAVAM
DKGGGRGRSRSRTARQLTWVLLLRARRAAGRLASFAAAAARRFRRSPADAADELGRGRGRLMYGFIRGFLALSLLALA
VELAAYWNGWRLRRPELHVPEAVEIEGWAHSAYISWMSFRADYIRRPIEFLSKACILLFVIQSMDRLVLCLGCFWIKLRKI
KPRIEGDPFREGSGYQHPMVLVQIPMCNEKEVYEQSISAACQLDWPREKFLIQVLDDSSDESIQLLIKAEVSKWSHQGV
NIVYRHRVLRTGYKAGNLKSAMSCDYVKDYEFVAIFDADFQPTPDFLKKTIPHFEGNPELGLVQARWSFVNKDENLLTR
LQNINLCFHFEVEQQVNGVFLNFFGFNGTAGVWRIQALEESGGWLERTTVEDMDIAVRAHLNGWKFIFLNDVKVLCE
LPESYEAYRKQQHRWHSGPMHLFRLCLPDILTAKISSWKKANLILLFFLLRKLILPFYSFTLFCVILPLTMFVPEAELPVWVI
CYVPVCMSFLNILPSPRSFPFIVPYLLFENTMSVTKFNAMVSGLFKLGSSYEWIVTKKSGRSSESDLSTAAERDTKDLTLPR
LQKQISESELIELKMQKERQEKAPLGAKKANKVYKKELALSLLLLTAATRSLLSAQGIHFYFLLFQGVSFLFVGLDLIGEQID

>OS08G15420_OsCslC3MAPPPNTYSESWWGGKEERGTPVVVKMDNPYSLVEIDGPGMAAPSEKARGKNAKQLT
WVLLLRAHRAVGCVAWLAAGFWAVLGAVNRRVRRSRDADAEPDAEASGRGRAMLRFLRGFLLLSLAMLAFETVAHL
KGWHFPRSAAGLPEKYLRRLPEHLQHLPEHLRRHLPEHLRMPEKEEIEGWLHRAYVAWLAFRIDYIAWAIQKLSGFCIAL
FMVQSVDRLVLCLGCFWIKLRGIKPVADTSISNDDIEATAGDGGGYFPMVLIQMPMCNEKEVYETSISHVCQIDWPRE
RMLVQVLDDSDDETCQMLIKAEVTKWSQRGVNIIYRHRLNRTGYKAGNLKSAMSCDYVRDYEFVAIFDADFQPNPDF
LKLTVPHFKGNPELGLVQARWSFVNKDENLLTRLQNINLCFHFEVEQQVNGVYLSFFGFNGTAGVWRIKALEDSGGW
MERTTVEDMDIAVRAHLNGWKFIFLNDVKVLCELPESYQAYRKQQHRWHSGPMQLFRLCLPAVFKSKISTWKKANLV
MLFFLLRKLILPFYSFTLFCVILPLTMFVPEAELPIWVICYVPVIMSVLNILPAPKSFPFVIPYLLFENTMSVTKFNAMVSGLF
QLGSSYEWVVTKKAGRTSSESDILALAEAADADARPPPAKLHRGVSEGGLKEWAKLHKEQEDATAAAAAAAAPGTPVK
KSKAAKAPNRIFKKELALAFLLLTAATRSLLSAQGLHFYFLLFQGVTFLAVGLDLIGEQVS

>OS05G43530_OsCslC7MAPSWWGRSGGGGVGNGGGTPVVVKMENPNWSISEVEAAEVAPGSPAGAGKAGRGK
NARQITWVLLLKAHRAAGRLTGAASAALAVASAARRRVASGRTDADAAPGESTALRARSYGCIRVSLVLSLLLLAVEVAA

YLQGWHLEEVASLLAVDGLFAASYAGWMRLRLDYLAPPLQFLTNACVALFMVQSIDRLVLCLGCFWIRFKGIKPVPQA
AAAGKPDVEAGAGDYPMVLVQMPMCNEREVYQQSIGAVCNLDWPKSNFLVQVLDDSDDATTSALIKEEVEKWQRE
GVRIIYRHRVIRDGYKAGNLKSAMNCSYVKDYEFVVIFDADFQPQADFLKRTVPHFKGKDDVGLVQARWSFVNKDENL
LTRLQNVNLCFHFEVEQQVNGAFLNFFGFNGTAGVWRIKALEDSGGWMERTTVEDMDIAVRAHLKGWKFVFLNDV
ECQCELPESYEAYRKQQHRWHSGPMQLFRLCFVDIIKSKIGFWKKFNLIFLFFLLRKLILPFYSFTLFCVILPMTMFVPEAEL
PAWVVCYIPATMSILNILPAPKSFPFIVPYLLFENTMSVTKFNAMISGLFQLGSAYEWVVTKKSGRSSEGDLVGLVEKHSK
QQRVGSAPNLDALTKEESNPKKDSKKKKHNRIYRKELALSFLLLTAAARSLLSAQGIHFYFLLFQGVSFLVVGLDLIGEQVE

>OS03G56060_OsCslC9MVLVQIPMCNEKEVYQQSIAAVCNLDWPRSNFLVQVLDDSDDPTTQTLIREEVLKWQQN
GARIVYRHRVLRDGYKAGNLKSAMSCSYVKDYEFVAIFDADFQPNPDFLKRTVPHFKDNDELGLVQARWSFVNKDENL
LTRLQNINLCFHFEVEQQVNGIFLNFFGFNGTAGVWRIKALDDSGGWMERTTVEDMDIAVRAHLRGWKFIFLNDVEC
QCELPESYEAYRKQQHRWHSGPMQLFRLCLPDIIKCKIVFWKKANLIFLFFLLRKLILPFYSFTLFCIILPMTMFVPEAELPD
WVVCYIPALMSLLNILPSPKSFPFIIPYLLFENTMSVTKFNAMISGLFQLGNAYEWVVTKKSGRSSEGDLISLAPKELKHQK
TESAPNLDAIAKEQSAPRKDVKKKHNRIYKKELALSLLLLTAAARSLLSKQGIHFYFLLFQGISFLLVGLDLIGEQIE

>OS10G42750_OsCslD1MASKGILKNGGKPPTAPSSAAPTVVFGRRTDSGRFISYSRDDLDSEISSVDFQDYHVHIPMT
PDNQPMDPAAGDEQQYVSSSLFTGGFNSVTRAHVMEKQASSARATVSACMVQGCGSKIMRNGRGADILPCECDFKI
CVDCFTDAVKGGGGVCPGCKEPYKHAEWEEVVSASNHDAINRALSLPHGHGHGPKMERRLSLVKQNGGAPGEFDH
NRWLFETKGTYGYGNAIWPEDDGVAGHPKELMSKPWRPLTRKLRIQAAVISPYRLLVLIRLVALGLFLMWRIKHQNED
AIWLWGMSIVCELWFALSWVLDQLPKLCPINRATDLSVLKDKFETPTPSNPTGKSDLPGIDIFVSTADPEKEPVLVTANTI
LSILAADYPVDKLACYVSDDGGALLTFEAMAEAASFANLWVPFCRKHEIEPRNPDSYFNLKRDPFKNKVKGDFVKDRRR
VKREYDEFKVRVNGLPDAIRRRSDAYHAREEIQAMNLQREKMKAGGDEQQLEPIKIPKATWMADGTHWPGTWLQA
SPEHARGDHAGIIQVMLKPPSPSPSSSGGDMEKRVDLSGVDTRLPMLVYVSREKRPGYDHNKKAGAMNALVRASAIM
SNGPFILNLDCDHYVYNSKAFREGMCFMMDRGGDRLCYVQFPQRFEGIDPSDRYANHNTVFFDVNMRALDGLQGPV
YVGTGCLFRRIALYGFDPPRSKDHTTPWSCCLPRRRRTRSQPQPQEEEEETMALRMDMDGAMNMASFPKKFGNSSFL
IDSIPVAEFQGRPLADHPSVKNGRPPGALTIPRETLDASIVAEAISVVSCWYEEKTEWGTRVGWIYGSVTEDVVTGYRM
HNRGWKSVYCVTHRDAFRGTAPINLTDRLHQVLRWATGSVEIFFSRNNALFASSKMKVLQRIAYLNVGIYPFTSVFLIVY
CFLPALSLFSGQFIVQTLNVTFLTYLLIITITLCLLAMLEIKWSGIALEEWWRNEQFWLIGGTSAHLAAVLQGLLKVIAGIEIS
FTLTSKQLGDDVDDEFAELYAVKWTSLMIPPLTIIMINLVAIAVGFSRTIYSTIPQWSKLLGGVFFSFWVLAHLYPFAKGL
MGRRGRTPTIVYVWSGLVAITISLLWIAIKPPSAQANSQLGGSFSFP

>OS06G02180_OsCslD2MASSGGGGLRHSNSSRLSRMSYSGEDGRAQAPGGGGDRPMVTFARRTHSGRYVSYSRD
DLDSELGNSGDMSPESGQEFLNYHVTIPATPDNQPMDPAISARVEEQYVSNSLFTGGFNSVTRAHLMDKVIESEASHP
QMAGAKGSSCAINGCDAKVMSDERGDDILPCECDFKICADCFADAVKNGGACPGCKDPYKATELDDVVGARPTLSLP
PPPGGLPASRMERRLSIMRSQKAMTRSQTGDWDHNRWLFETKGTYGYGNAIWPKENEVDNGGGGGGGGGLGGG
DGQPAEFTSKPWRPLTRKLKIPAGVLSPYRLLILIRMAVLGLFLAWRIKHKNEDAMWLWGMSVVCELWFGLSWLLDQ
LPKLCPVNRATDLAVLKDKFETPTPSNPNGRSDLPGLDIFVSTADPEKEPPLVTANTILSILAADYPVEKLSCYVSDDGGAL
LTFEAMAEAASFANMWVPFCRKHDIEPRNPESYFNLKRDPYKNKVRSDFVKDRRRVKREYDEFKVRINSLPDSIRRRSD
AYHAREEIKAMKRQREAALDDVVEAVKIPKATWMADGTHWPGTWIQPSAEHARGDHAGIIQVMLKPPSDDPLYGTS
GEEGRPLDFTEVDIRLPMLVYVSREKRPGYDHNKKAGAMNALVRSSAVMSNGPFILNLDCDHYVYNSQAFREGMCF
MMDRGGDRIGYVQFPQRFEGIDPSDRYANHNTVFFDVNMRALDGIMGPVYVGTGCLFRRIALYGFDPPRSKEHSGCC
SCCFPQRRKVKTSTVASEERQALRMADFDDEEMNMSQFPKKFGNSNFLINSIPIAEFQGRPLADHPGVKNGRPPGALT
VPRDLLDASTVAEAISVISCWYEDKTEWGQRVGWIYGSVTEDVVTGYRMHNRGWKSVYCVTKRDAFRGTAPINLTDR

LHQVLRWATGSVEIFFSRNNALLASRKMKFLQRIAYLNVGIYPFTSIFLIVYCFLPALSLFSGQFIVRTLNVTFLTYLLVITLT
MCMLAVLEIKWSGISLEEWWRNEQFWLIGGTSAHLAAVLQGLLKVIAGIEISFTLTSKSGGDEADDEFADLYIVKWTSL
MIPPIVIMMVNLIAIAVGFSRTIYSEIPQWSKLLGGVFFSFWVLAHLYPFAKGLMGRRGRTPTIVFVWSGLLAITISLLWV
AINPPSQNSQIGGSFTFP

>OS08G25710_OsCslD3MSTGPGKKAIRNAGGVGGGAGPSAGGPRGPAGQAVKFARRTSSGRYVSLSREDIDMEGE
LAADYTNYTVQIPPTPDNQPMLNGAEPASVAMKAEEQYVSNSLFTGGFNSATRAHLMDKVIESSVSHPQMAGAKGS
RCAMPACDGSAMRNERGEDVDPCECHFKICRDCYLDAQKDGCICPGCKEHYKIGEYADDDPHDGKLHLPGPGGGGN
KSLLARNQNGEFDHNRWLFESSGTYGYGNAFWPKGGMYDDDLDDDVDKLGGDGGGGGGGGPLPEQKPFKPLTRKI
PMPTSVISPYRIFIVIRMFVLLFYLTWRIRNPNMEALWLWGMSIVCELWFAFSWLLDMLPKVNPVNRSTDLAVLKEKFE
TPSPSNPHGRSDLPGLDVFVSTADPEKEPVLTTATTILSILAVDYPVEKLACYVSDDGGALLTFEAMAEAASFANVWVPF
CKKHDIEPRNPDSYFSVKGDPTKGKRRNDFVKDRRRVKREFDEFKVRINGLPDSIRRRSDAFNAREDMKMLKHLRETGA
DPSEQPKVKKATWMADGSHWPGTWAASAPDHAKGNHAGILQVMLKPPSPDPLYGMHDDDQMIDFSDVDIRLPML
VYMSREKRPGYDHNKKAGAMNALVRCSAVMSNGPFMLNFDCDHYINNAQAVREAMCFFMDRGGERIAYIQFPQRF
EGIDPSDRYANNNTVFFDGNMRALDGLQGPMYVGTGCMFRRFAVYGFDPPRTAEYTGWLFTKKKVTTFKDPESDTQ
TLKAEDFDAELTSHLVPRRFGNSSPFMASIPVAEFQARPLADHPAVLHGRPSGALTVPRPPLDPPTVAEAVSVISCWYED
KTEWGDRVGWIYGSVTEDVVTGYRMHNRGWRSVYCITKRDAFLGTAPINLTDRLHQVLRWATGSVEIFFSRNNAFLA
SRKLMLLQRISYLNVGIYPFTSIFLLVYCFIPALSLFSGFFIVQKLDIAFLCYLLTMTITLVALGILEGLLKVMAGIEISFTLTAKA
AADDNEDIYADLYIVKWSSLLIPPITIGMVNIIAIAFAFARTIYSDNPRWGKFIGGGFFSFWVLAHLNPFAKGLMGRRGKT
PTIVFVWSGLLSITVSLLWVAISPPEANSNGGARGGGFQFP

>OS12G36890_OsCslD4MSRRLSLPAGAPVTVAVSPVRSPGGDAVVRRGSGLTSPVPRHSLGSSTATLQVSPVRRSGG
SRYLGASRDGGADESAEFVHYTVHIPPTPDRATASVASEAEAAAEAEEVHRPQRSYISGTIFTGGLNCATRGHVLNFSGE
GGATAASRAAASGNMSCKMRGCDMPAFLNGGRPPCDCGFMICKECYAECAAGNCPGCKEAFSAGSDTDESDSVTD
DDDDEAVSSSEERDQLPLTSMARKFSVVHSMKVPGAAANGNGKPAEFDHARWLFETKGTYGYGNALWPKDGHAHS
GAGFVAADEPPNFGARCRRPLTRKTSVSQAILSPYRLLIAIRLVALGFFLAWRIRHPNPEAVWLWAMSVACEVWFAFS
WLLDSLPKLCPVHRAADLAVLAERFESPTARNPKGRSDLPGIDVFVTSADPEKEPPLVTANTILSILAADYPVEKLACYLSD
DGGALLSFEALAETASFARTWVPFCRKHGVEPRCPEAYFGQKRDFLKNKVRVDFVRERRKVKREYDEFKVRVNSLPEAI
RRRSDAYNAGEELRARRRQQEEAAAAAAAGNGELGAAAVETAAVKATWMSDGSHWPGTWTCPAADHARGDHAGI
IQAMLAPPTSEPVMGGEAAECGGLIDTTGVDVRLPMLVYVSREKRPGYDHNKKAGAMNALVRTSAIMSNGPFILNLD
CDHYVHNSSALREGMCFMLDRGGDRVCFVQFPQRFEGVDPSDRYANHNLVFFDVSMRAMDGLQGPMYVGTGCVF
RRTALYGFSPPRATEHHGWLGRRKIKLFLTKKKSMGKKTDRAEDDTEMMLPPIEDDDGGADIEASAMLPKRFGGSATF
VASIPVAEYQGRLLQDTPGCHHGRPAGALAVPREPLDAATVAEAIGVISCFYEEKTEWGRRIGWIYGSVTEDVVTGYRM
HNRGWRSVYCVTPRRDAFRGTAPINLTDRLHQVLRWATGSVEIFFSRNNALFASPRMKLLQRVAYFNAGMYPFTSVFL
LAYCLLPAVSLFSGKFIVQRLSATFLAFLLVITLTLCLLALLEIKWSGITLHEWWRNEQFWVIGGTSAHPAAVLQGLLKVIA
GVDISFTLTSKPGNGGGDGGVGGEGNDDEAFAELYEVRWSYLMVPPVTIMMVNAVAIAVAAARTLYSEFPQWSKLLG
GAFFSFWVLCHLYPFAKGLLGRRGRVPTIVFVWSGLISMIISLLWVYINPPAGARERIGGGGFSFP

>OS06G22980_OsCslD5MSVDYANYTVLMPPTPDNQPSGGAPPAAPSAGGARPGDLPLPPYGSSSSSRLVNRRGGG
DDGAKMDRRLSTARVPAPSSNKSLLVRSQTGDFDHNRWLFETKGTYGIGNAYWPQDNVYGDDGGGGAVKMEDLVE
KPWKPLSRKVPIPPGILSPYRLLVLVRFVALFLFLVWRVTNPNMDALWLWGISIVCEFWFAFSWLLDQMPKLNPINRAA
DLAALKEKFESPSPTNPTGRSDLPGLDVFISTADPYKEPTLVTANTLLSILATEYPVEKLFVYISDDGGALLTFESMAEACAF
AKVWVPFCRKHSIEPRNPDSYFTQKGDPTKGKKRPDFVKDRRWIKREYDEFKIRVNSLPDLIRRRANALNARERKLARD

KQAAGDADALASVKAATWMADGTHWPGTWLDPSPDHAKGDHASIVQVMIKNPHHDVVYGEAGDHPYLDMTDVD
MRIPMFAYLSREKRAGYDHNKKAGAMNAMVRASAILSNGPFMLNFDCDHYIYNCQAIREAMCYMLDRGGDRICYIQ
FPQRFEGIDPSDRYANHNTVFFDGNMRALDGLQGPMYVGTGCLFRRYAIYGFNPPRAIEYRGTYGQTKVPIDPRQGSE
AMPGAGGGRSGGGSVGGDHELQALSTAHPDHEAPQKFGKSKMFIESIAVAEYQGRPLQDHPSVLNGRPPGALLMPR
PPLDAATVAESVSVISCWYEDNTEWGQRVGWIYGSVTEDVVTGYRMHNRGWRSVYCITRRDAFRGTAPINLTDRLHQ
VLRWATGSVEIFFSKNNAVLASRRLKFLQRMAYLNVGIYPFTSLFLIMYCLLPALSLFSGQFIVATLDPTFLSYLLLITITLML
LCLLEVKWSGIGLEEWWRNEQFWVIGGTSAHLAAVLQGLLKVVAGIEISFTLTAKAAAEDDDDPFAELYLIKWTSLFIPP
LAVIGINIIALVVGVSRTVYAEIPQYSKLLGGGFFSFWVLAHYYPFAKGLMGRRGRTPTIVYVWAGLISITVSLLWITISPPD
DSVAQGGIDV

>OS09G30120_OsCslE1MAYNYPSEKISVYLSDDGGSILTFYALWEASIFAKKWLPFCKRYNIEPRSPAAYFSESKVHHN
LCIPKEWALIKNLYEEMRERIDTATMSGKIPEEMKLKHKGFDEWNSDFTLKNHQPIVQILIDGKNRNAIDDDRNVLPTM
VYVAREKRPQYHHNFKAGALNALIRVSSVISDSPVILNVDCDMYSNNSDSIRDALCFFLDEEMGQKIGFVQYPQIFNNM
TQNDIYGNSFNVSYHVEMCGLDSVGGCLYIGTGCFHRREILCGRIFSKDYKENWNRGIKERGKENINEIEEKATSLVTCTY
EHRTQWGNDIGVKYGFPAEDIITGLAIHCRGWESAFINPKRAAFLGLAPSTLAQNILQHKRWSEGNLTIFLSKYCSFLFG
HGKIKLQLQMGYCICGLWAANSLPTLYYVVIPSLGLVKGTPLFPQIMSPWATPFIYVFCVKTLYGLYEALLSGDTLKGWW
NGQRMWMVKSITSYLYGFIDTIRKCVGMSKMSFEVTAKVSGHDEAKRYEQEILEFGSSSPEYVIIATVALLNFVCLVGGL
SQIMAGVWNMPWNVFLPQAILCGMIVIINMPIYEAMFLRKDNGRIPTAVTLASIGFVMLAFLVPIV

>OS02G49330_OsCslE2MATAAALALHTQFRPPRSPRRLRQHLALPSGVLIRSPVRASAASASASAPAQREAAAAGVP
WGCEIESLESAVSLERWLTDSGLPEQRLGIQRVDVGERGLVALKNIRKGEKLLFVPPSLVITADSEWGCPEVGNVLKRNS
VPDWPLIATYLISEASLESSSRWSSYIAALPRQPYSLLYWTRPELDAYLVASPIRERAIQRITDVVGTYNDLRDRIFSKHSDL
FPEEVYNLETFRWSFGILFSRLVRLPSMDGRVALVPWADMLNHSPEVETFLDYDKSSGGIVFTTDRSYQPGEQVFISYGK
KSSGELLLSYGFVPKEGTNPNDSVELLVSLNKSDKCYKEKLQALKRNGLSEFESFPLRVTGWPVELMAYAFLVVSPPEMS
QRFEEMAVAASNKSPSKPGLNYPELEEQALQFILDCCESNIAKYTKFLEGSSGSLQLSTNSKQANRTLLLKQLARDLCISER
RILYRTQYVVFVADEAQTVEEKEIDKSDYNLSRQATDARRLEVVGGSMAGSGGGVVSGGRQRGPPLFATEKPGRMAM
AAYRVSAATVFAGVLLIWLYRATHLPPGGGDGVRRWAWLGMLAAELWFGFYWVLTLSVRWCPVYRRTFKDRLAQRI
LIANSYSEDELPSVDIFVCTADPTAEPPMLVISTVLSVMAYDYLPEKLNIYLSDDAGSVLTFYVLCEASEFAKHWIPFCKKYK
VEPRSPAAYFAKVASPPDGCGPKEWFTMKELYKDMTDRVNSVVNSGRIPEVPRCHSRGFSQWNENFTSSDHPSIVQIR
VSSVISNSPIIMNVDCDMYSNNSESIRDALCFFLDEEQGQDIGFVQYPQNFENVVHNDIYGHPINVVNELDHPCLDGW
GGMCYYGTGCFHRREALCGRIYSQEYKEDWTRVAGRTEDANELEEMGRSLVTCTYEHNTIWGIEKGVRYGCPLEDVTT
GLQIQCRGWRSVYYNPKRKGFLGMTPTSLGQILVLYKRWTEGFLQISLSRYSPFLLGHGKIKLGLQMGYSVCGFWAVNS
FPTLYYVTIPSLCFLNGISLFPEKTSPWFIPFAYVMVAAYSCSLAESLQCGDSAVEWWNAQRMWLIRRITSYLLATIDTFR
RILGISESGFNLTVKVTDLQALERYKKGMMEFGSFSAMFVILTTVALLNLACMVGVKCTASTSTCEMGAI

>OS09G30130_OsCslE6METTTTERRRLFATEKVGGRAVYRLQAATVAAGILLVLYYRATRVPAAGEGRAAWLGMAA
AELWFAVYWVITQSVRWCPVRRRTFKNRLAERYKENLPGVDVFVCTADPHAEPPSLVISTILSVMAYNYPSEKISVYLSD
DGGSILTFYALWEASMFAKKWLPFCRRYNIEPRSPAAYFSESEGHHNLCSPKEWSFIKNLYEEMRERIDSAVMSGKIPEEI
KLKHKGFDEWNSEMTSKNHQPIVQVLIDGKSQNAVDDDGNVLPTLVYMAREKSPQYHHNFKAGALNALIRVSALISDS
PVILNVDCDMYSNNSDSIRDALCFFLDEEMSHKIGFVQYPQNYNNMTKNNIYGNSLNVINHVEMRGLDSAGGCLYIGT
GCFHRREILCGKKFSKDYKEDWGRGIKERGHENIDEIEEKAKSLATCTYELRTQWGNEIGVKYGCPVEDVITGLAIHCRG
WESVYMEPQRAAFVGVAPATLAQTILQHKRWSEGNFTIFLSKHNTFLFGHGKISLQLQMGYCIYGLWAANSLPTIYYV
MIPALGLVKGTPLFPEIMSPWATPFIYVFCVKTLYSLYEALLSGDTLKGWWNGQRMWMVKRITSYLYGFIDTIRKLLGLS

KMSFEITAKVSDGDEAKRYEQEILEFGSSSPEFVIIATVALLNFVCLVAGLSKIMAGVWNVFLPQVILCGLIVITNIPIYEAM
FVRKDKGRIPLPVTLASIGFVMLAFLLPIV

>OS07G36700_OsCslF1MSAAAAVTSWTNGCWSPAATRVNDGGKDDVWVAVDEADVSGARGSDGGGRPPLFQT
YKVKGSILHPYRFLILARLIAIVAFFAWRIRHKNRDGAWLWTMSMVGDVWFGFSWVLNQLPKQSPIKRVPDIAALADR
HSGDLPGVDVFVTTVDPVDEPILYTVNTILSILAADYPVDRYACYLSDDGGTLVHYEAMVEVAKFAELWVPFCRKHCVE
PRSPENYFAMKTQAYKGGVPGELMSDHRRVRREYEEFKVRIDSLSSTIRQRSDVYNAKHAGENATWMADGTHWPGT
WFEPADNHQRGKHAGIVQVLLNHPSCKPRLGLAASAENPVDFSGVDVRLPMLVYISREKRPGYNHQKKAGAMNVML
RVSALLSNAPFVINFDGDHYVNNSQAFRAPMCFMLDGRGRGGENTAFVQFPQRFDDVDPTDRYANHNRVFFDGTM
LSLNGLQGPSYLGTGTMFRRVALYGVEPPRWGAAASQIKAMDIANKFGSSTSFVGTMLDGANQERSITPLAVLDESVA
GDLAALTACAYEDGTSWGRDVGWVYNIATEDVVTGFRMHRQGWRSVYASVEPAAFRGTAPINLTERLYQILRWSGG
SLEMFFSHSNALLAGRRLHPLQRVAYLNMSTYPIVTVFIFFYNLFPVMWLISEQYYIQRPFGEYLLYLVAVIAMIHVIGMF
EVKWAGITLLDWCRNEQFYMIGSTGVYPTAVLYMALKLVTGKGIYFRLTSKQTAASSGDKFADLYTVRWVPLLIPTIVIM
VVNVAAVGVAVGKAAAWGPLTEPGWLAVLGMVFNVWILVLLYPFALGVMGQWGKRPAVLFVAMAMAVAAVAA
MYVAFGAPYQAELSGVAASLGKVAAASLTGPSG

>OS07G36690_OsCslF2MAATAASTMSAAAAVTRRINAALRVDATSGDVAAGADGQNGRRSPVAKRVNDGGGGK
DDVWVAVDEKDVCGARGGDGAARPPLFRTYKVKGSILHPYRFLILLRLIAIVAFFAWRVRHKNRDGVWLWTMSMVG
DVWFGFSWVLNQLPKLSPIKRVPDLAALADRHSGDLPGVDVFVTTVDPVDEPILYTVNTILSILAADYPVDRYACYLSDD
GGTLVHYEAMVEVAKFAELWVPFCRKHCVEPRSPENYFAMKTQAYKGGVPGELMSDHRRVRREYEEFKVRIDSLSSTI
RQRSDVYNAKHAGENATWMADGTHWPGTWFEPADNHQRGKHAGIVQVLLNHPSCKPRLGLAASAENPVDFSGVD
VRLPMLVYISREKRPGYNHQKKAGAMNVMLRVSALLSNAPFVINFDGDHYVNNSQAFRAPMCFMLDGRGRGGENT
AFVQFPQRFDDVDPTDRYANHNRVFFDGTMLSLNGLQGPSYLGTGTMFRRVALYGVEPPRWGAAASQIKAMDIANK
FGSSTSFVGTMLDGANQERSITPLAVLDESVAGDLAALTACAYEDGTSWGRDVGWVYNIATEDVVTGFRMHRQGWR
SVYASVEPAAFRGTAPINLTERLYQILRWSGGSLEMFFSHSNALLAGRRLHPLQRVAYLNMSTYPIVTVFIFFYNLFPVM
WLISEQYYIQRPFGEYLLYLVAVIAMIHVIGMFEVKWAGITLLDWCRNEQFYMIGSTGVYPTAVLYMALKLVTGKGIYFR
LTSKQTTASSGDKFADLYTVRWVPLLIPTIVIIVVNVAAVGVAVGKAAAWGPLTEPGWLAVLGMVFNVWILVLLYPFAL
GVMGQWGKRPAVLFVAMAMAVAAVAAMYVAFGAPYQAELSGGAASLGKAAAASLTGPSG

>OS07G36750_OsCslF3MASPASVAGGGEDSNGCSSLIDPLLVSRTSSIGGAERKAAGGGGGGAKGKHWAAADKGE
RRAAKECGGEDGRRPLLFRSYRVKGSLLHPYRALIFARLIAVLLFFGWRIRHNNSDIMWFWTMSVAGDVWFGFSWLLN
QLPKFNPVKTIPDLTALRQYCDLADGSYRLPGIDVFVTTADPIDEPVLYTMNCVLSILAADYPVDRSACYLSDDSGALILYE
ALVETAKFATLWVPFCRKHCIEPRSPESYFELEAPSYTGSAPEEFKNDSRIVHLEYDEFKVRLEALPETIRKRSDVYNSMKT
DQGAPNATWMANGTQWPGTWIEPIENHRKGHHAGIVKVVLDHPIRGHNLSLKDSTGNNLNFNATDVRIPMLVYVS
RGKNPNYDHNKKAGALNAQLRASALLSNAQFIINFDCDHYINNSQAFRAAICFMLDQREGDNTAFVQFPQRFDNVDP
KDRYGNHNRVFFDGTMLALNGLQGPSYLGTGCMFRRLALYGIDPPHWRQDNITPEASKFGNSILLLESVLEALNQDRF
ATPSPVNDIFVNELEMVVSASFDKETDWGKGVGYIYDIATEDIVTGFRIHGQGWRSMYCTMEHDAFCGTAPINLTERL
HQIVRWSGGSLEMFFSHNNPLIGGRRLQPLQRVSYLNMTIYPVTSLFILLYAISPVMWLIPDEVYIQRPFTRYVVYLLVIIL
MIHMIGWLEIKWAGITWLDYWRNEQFFMIGSTSAYPTAVLHMVVNLLTKKGIHFRVTSKQTTADTNDKFADLYEMR
WVPMLIPTMVVLVANIGAIGVAIGKTAVYMGVWTIAQKRHAAMGLLFNMWVMFLLYPFALAIMGRWAKRSIILVVL
LPIIFVIVALVYYVATHILLANIIPF

>OS07G36740_OsCslF4MSAAAVTRRINAGGLRVEVTNGNGAAGVYVAAAAAPCSPAAKRVNDGGGKDDVWVAV
DEADVSGPSGGDGVRPTLFRTYKVKGSILHPYRFLILVRLIAIVAFFAWRVRHKNRDGAWLWTMSMAGDVWFGFSW
ALNQLPKLNPIKRVADLAALADRQQHGTSGGGELPGVDVFVTTVDPVDEPILYTVNSILSILAADYPVDRYACYLSDDGG
TLVHYEAMVEVAKFAELWVPFCRKHCVEPRAPESYFAMKTQAYRGGVAGELMSDRRRVRREYEEFKVRIDSLFSTIRKR
SDAYNRAKDGKDDGENATWMADGTHWPGTWFEPAENHRKGQHAGIVQVLLNHPTSKPRFGVAASVDNPLDFSGV
DVRLPMLVYISREKRPGYNHQKKAGAMNALLRVSALLSNAPFIINFDCDHYVNNSQAFRAPMCFMLDRRGGGDDVAF
VQFPQRFDDVDPTDRYANHNRVFFDGTTLSLNGLQGPSYLGTGTMFRRAALYGLEPPRWGAAGSQIKAMDNANKFG
ASSTLVSSMLDGANQERSITPPVAIDGSVARDLAAVTACGYDLGTSWGRDAGWVYDIATEDVATGFRMHQQGWRSV
YTSMEPAAFRGTAPINLTERLYQILRWSGGSLEMFFSHSNALLAGRRLHPLQRIAYLNMSTYPIVTVFIFFYNLFPVMWLI
SEQYYIQQPFGEYLLYLVAIIAMIHVIGMFEVKWSGITVLDWCRNEQFYMIGSTGVYPTAVLYMALKLFTGKGIHFRLTS
KQTTASSGDKFADLYTVRWVPLLIPTIVVLAVNVGAVGVAVGKAAAWGLLTEQGRFAVLGMVFNVWILALLYPFALGI
MGQRGKRPAVLFVATVMAVAAVAIMYAAFGAPYQAGLSGVAASLGKAASLTGPSG

>OS08G06380_OsCslF6MAPAVAGGGGRRNNEGVNGNAAAPACVCGFPVCACAGAAAVASAASSADMDIVAAGQ
IGAVNDESWVAVDLSDSDDAPAAGDVQGALDDRPVFRTEKIKGVLLHPYRVLIFVRLIAFTLFVIWRIEHKNPDAMWL
WVTSIAGEFWFGFSWLLDQLPKLNPINRVPDLAVLRRRFDHADGTSSLPGLDIFVTTADPIKEPILSTANSILSILAADYPV
DRNTCYLSDDSGMLLTYEAMAEAAKFATLWVPFCRKHAIEPRGPESYFELKSHPYMGRAQEEFVNDRRRVRKEYDDFK
ARINGLEHDIKQRSDSYNAAAGVKDGEPRATWMADGSQWEGTWIEQSENHRKGDHAGIVLVLLNHPSHARQLGPP
ASADNPLDFSGVDVRLPMLVYVAREKRPGCNHQKKAGAMNALTRASAVLSNSPFILNLDCDHYINNSQALRAGICFML
GRDSDTVAFVQFPQRFEGVDPTDLYANHNRIFFDGTLRALDGLQGPIYVGTGCLFRRITLYGFEPPRINVGGPCFPRLGG
MFAKNRYQKPGFEMTKPGAKPVAPPPAATVAKGKHGFLPMPKKAYGKSDAFADTIPRASHPSPYAAEAAVAADEAAI
AEAVMVTAAAYEKKTGWGSDIGWVYGTVTEDVVTGYRMHIKGWRSRYCSIYPHAFIGTAPINLTERLFQVLRWSTGSL
EIFFSRNNPLFGSTFLHPLQRVAYINITTYPFTALFLIFYTTVPALSFVTGHFIVQRPTTMFYVYLAIVLGTLLILAVLEVKWA
GVTVFEWFRNGQFWMTASCSAYLAAVLQVVTKVVFRRDISFKLTSKLPAGDEKKDPYADLYVVRWTWLMITPIIIILVNI
IGSAVAFAKVLDGEWTHWLKVAGGVFFNFWVLFHLYPFAKGILGKHGKTPVVVLVWWAFTFVITAVLYINIPHIHGPG
RHGAASPSHGHHSAHGTKKYDFTYAWP

>OS10G20260_OsCslF7MPPSAGLATESLPAATCPAKKDAYAAAASPESETKLAAGDERAPLVRTTRISTTTIKLYRLTIF
VRIAIFVLFFKWRITYAARAISSTDAGGIGMSKAATFWTASIAGELWFAFMWVLDQLPKTMPVRRAVDVTALNDDTLL
PAMDVFVTTADPDKEPPLATANTVLSILAAGYPAGKVTCYVSDDAGAEVTRGAVVEAARFAALWVPFCRKHGVEPRN
PEAYFNGGEGGGGGGKARVVARGSYKGRAWPELVRDRRRVRREYEEMRLRIDALQAADARRRRCGAADDHAGVVQ
VLIDSAGSAPQLGVADGSKLIDLASVDVRLPALVYVCREKRRGRAHHRKAGAMNALLRASAVLSNAPFILNLDCDHYVN
NSQALRAGICFMIERRGGGAEDAGDVAFVQFPQRFDGVDPGDRYANHNRVFFDCTELGLDGLQGPIYVGTGCLFRRV
ALYGVDPPRWRSPGGGVAADPAKFGESAPFLASVRAEQSHSRDDGDAIAEASALVSCAYEDGTAWGRDVGWVYGTV
TEDVATGFCMHRRGWRSAYYAAAPDAFRGTAPINLADRLHQVLRWAAGSLEIFFSRNNALLAGGRRRLHPLQRAAYL
NTTVYPFTSLFLMAYCLFPAIPLIAGGGGWNAAPTPTYVAFLAALMVTLAAVAVLETRWSGIALGEWWRNEQFWMVS
ATSAYLAAVAQVALKVATGKEISFKLTSKHLASSATPVAGKDRQYAELYAVRWTALMAPTAAALAVNVASMAAAGGG
GRWWWWDAPSAAAAAAAALPVAFNVWVVVHLYPFALGLMGRRSKAVRPILFLFAVVAYLAVRFLCLLLQFHTA

>OS07G36630_OsCslF8MAANGGGGGAGGCSNGGGGGAVNGAAANGGGGGGGGSKGATTRRAKVSPMDRYW
VPTDEKEMAAAVADGGEDGRRPLLFRTFTVRGILLHPYRLLTLVRLVAIVLFFIWRIRHPYADGMFFWWISVIGDFWFG
VSWLLNQVAKLKPIRRVPDLNLLQQQFDLPDGNSNLPGLDVFINTVDPINEPMIYTMNAILSILAADYPVDKHACYLSD
DGGSIIHYDGLLETAKFAALWVPFCRKHSIEPRAPESYFAVKSRPYAGSAPEDFLSDHRYMRREYDEFKVRLDALFTVIPK

RSDAYNQAHAEEGVKATWMADGTEWPGTWIDPSENHKKGNHAGIVQVMLNHPSNQPQLGLPASTDSPVDFSNVD
VRLPMLVYIAREKRPGYDHQKKAGAMNVQLRVSALLTNAPFIINFDGDHYVNNSKAFRAGICFMLDRREGDNTAFVQ
FPQRFDDVDPTDRYCNHNRVFFDATLLGLNGIQGPSYVGTGCMFRRVALYGVDPPRWRPDDGNIVDSSKKFGNLDSFI
SSIPIAANQERSIISPPALEESILQELSDAMACAYEDGTDWGKDVGWVYNIATEDVVTGFRLHRTGWRSMYCRMEPDA
FRGTAPINLTERLYQILRWSGGSLEMFFSHNCPLLAGRRLNFMQRIAYINMTGYPVTSVFLLFYLLFPVIWIFRGIFYIQKP
FPTYVLYLVIVIFMSEMIGMVEIKWAGLTLLDWIRNEQFYIIGATAVYPLAVLHIVLKCFGLKGVSFKLTAKQVASSTSEKF
AELYDVQWAPLLFPTIVVIAVNICAIGAAIGKALFGGWSLMQMGDASLGLVFNVWILLLIYPFALGIMGRWSKRPYILFV
LIVISFVIIALADIAIQAMRSGSVRLHFRRSGGANFPTSWGF

>OS07G36610_OsCslF9MSLLPPSCSAFLPSSSLYLRLAAELVLSAMLLTLVRLIAVVLFLAWRLKHRDSDAMWLWWISI
AGDFWFGVTWLLNQASKLNPVKRVPDLSLLRRRFDDGGLPGIDVFINTVDPVDEPMLYTMNSILSILATDYPADRHAA
YLSDDGASLAHYEGLIETARFAALWVPFCRKHRVEPRAPESYFAAKAAPYAGPALPEEFFGDRRLVRREYEEFKARLDALF
TDIPQRSEASVGNANTKGAKATLMADGTPWPGTWTEPAENHKKGQHAGIVKVMLSHPGEEPQLGMPASSGHPLDF
SAVDVRLPILVYIAREKRPGYDHQKKAGAMNAQLRVSALLSNAPFIFNFDGDHYINNSQAFRAALCFMLDCRHGDDTA
FVQFPQRFDDVDPTDRYCNHNRVFFDATLLGLNGVQGPSYVGTGCMFRRVALYGADPPRWRPEDDDAKALGCPGRY
GNSMPFINTIPAAASQERSIASPAAASLDETAAMAEVEEVMTCAYEDGTEWGDGVGWVYDIATEDVVTGFRLHRKG
WRSMYCAMEPDAFRGTAPINLTERLYQILRWSGGSLEMFFSRNCPLLAGCRLRPMQRVAYANMTAYPVSALFMVVY
DLLPVIWLSHHGEFHIQKPFSTYVAYLVAVIAMIEVIGLVEIKWAGLTLLDWWRNEQFYMIGATGVYLAAVLHIVLKRLL
GLKGVRFKLTAKQLAGGARERFAELYDVHWSPLLAPTVVVMAVNVTAIGAAAGKAVVGGWTPAQVAGASAGLVFNV
WVLVLLYPFALGIMGRWSKRPCALFALLVAACAAVAAGFVAVHAVLAAGSAAPSWLGWSRGATAILPSSWRLKRGF

>OS10G20090_CSLH1MEAAARGNKKLQERVPIRRTAWRLADLAILFLLLALLLHRVLHDSGAPWRRAALACEAWFTF
MWLLNVNAKWSPVRFDTFPENLAERIDELPAVDMFVTTADPVLEPPLVTVNTVLSLLALDYPAAGEKLACYVSDDGCS
PLTCYALREAARFARTWVPFCRRHGVAVRAPFRYFSSTPEFGPADGKFLEDWTFMKSEYEKLVHRIEDADEPSLLRHGG
GEFAEFLDVERGNHPTIIKVLWDNNRSRTGDGFPRLIYVSREKSPNLHHHYKAGAMNALTRVSALMTNAPFMLNLDCD
MFVNNPRVVLHAMCLLLGFDDEISCAFVQTPQKFYGALKDDPFGNQLEVSLMKVGRGIAGLQGIFYCGTGCFHRRKVI
YGMRTGREGTTGYSSNKELHSKFGSSNNFKESARDVIYGNLSTEPIVDISSCVDVAKEVAACNYEIGTCWGQEVGWVY
GSLTEDVLTGQRIHAAGWRSTLMEIEPPAFMGCAPNGGPACLTQLKRWASGFLEILISRNNPILTTTFKSLQFRQCLAYL
HSYVWPVRAPFELCYALLGPYCLLSNQSFLPKTSEDGFYIALALFIAYNTYMFMEFIECGQSARACWNNHRMQRITSAS
AWLLAFLTVILKTLGFSETVFEVTRKDKSTSDGDSNTDEPEPGRFTFDESTVFIPVTALAMLSVIAIAVGAWRVVLVTTEG
LPGGPGISEFISCGWLVLCFMPLLRGLVGSGRYGIPWSIKMKACLLVAIFLLFCKRN

>OS04G35020_OsCslH2MAVVAAAAATGSTTRSGGGGGEGTRSGRKKPPPPPLQERVPLGRRAAWAWRLAGLAVL
LLLLALLALRLLRHHGGAGGDGGVWRVALVCEAWFAALCALNVSAKWSPVRFVTRPENLVAEGRTPSTTAAEYGELPA
VDMLVTTADPALEPPLVTVNTVLSLLALDYPRAGERLACYVSDDGCSPLTCHALREAAGFAAAWVPFCRRYGVAVRAP
FRYFSSSSSPESGGPADRKFLDDWTFMKDEYDKLVRRIKNTDERSLLRHGGGEFFAEFLNVERRNHPTIVKTRVSAVMT
NAPIMLNMDCDMFVNNPQAVLHAMCLLLGFDDEASSGFVQAPQRFYDALKDDPFGNQMECFFKRFISGVQGVQGA
FYAGTGCFHRRKAVYGVPPNFNGAEREDTIGSSSYKELHTRFGNSEELNESARNIIWDLSSKPMVDISSRIEVAKAVSAC
NYDIGTCWGQEVGWVYGSLTEDILTGQRIHAMGWRSVLMVTEPPAFMGSAPIGGPACLTQFKRWATGQSEIIISRNN
PILATMFKRLKFRQCLAYLIVLGWPLRAPFELCYGLLGPYCILTNQSFLPKASEDGFSVPLALFISYNTYNFMEYMACGLSA
RAWWNNHRMQRIISVSAWTLAFLTVLLKSLGLSETVFEVTGKDKSMSDDDDNTDGADPGRFTFDSLPVFIPVTALAML
NIVAVTVGACRVAFGTAEGVPCAPGIGEFMCCGWLVLCFFPFVRGIVWGKGSYGIPWSVKLKASLLVAMFVTFCKRN

>OS04G35030_OsCslH3MAAASGEKEEEEKKLQERAPIRRTAWMLANFVVLFLLLALLVRRATAADAEERGVGGAA
WRVAFACEAWFAFVWLLNMNAKWSPARFDTYPENLAGRCGAAHRPRKSSCISGHLDLMRRQCALMQDRRAAGGR
HVRDDGGPGARAAGGDGEQGALAARRRLLPGRRRRRRRRLACYVSDDGCSPVTYYALREAAGFARTWVPFCRRHG
VAVRAPFRYFASAPEFGPADRKFLDDWTFMKSEYDKLVRRIEDADETTLLRQGGGEFAEFMDAKRTNHRAIVKVIWDN
NSKNRIGEEGGFPHLIYVSREKSPGHHHHYKAGAMNALTRVSAVMTNAPIMLNVDCDMFANDPQVVLHAMCLLLGF
DDEISSGFVQVPQSFYGDLKDDPFGNKLEVIYKKLLGGVAGI

>OS01G54620_OsCesAMMESGVPPCAACGDDAHAACRACSYALCKACLDEDAAEGRTTCARCGGEYGAPDPAHG
QGAVVEEEVEESHEPAAGGVRERVTMASQLSDHQDEGVHARTMSTHARTISSVSGVGSELNDESGKPIWKNRVESW
KEKKKEKKASAKKAAAKAQAPPVEEQIMDEKDLTDAYEPLSRIIPISKNKLTPYRAVIIMRLVVLGLFFHYRITNPVYSAFGL
WMTSVICEIWFGFSWILDQFPKWCPINRETYVDRLIARYGDGEDSGLAPVDFFVSTVDPLKEPPLITANTVLSILAVDYP
VEKISCYVSDDGSAMLTFESLAETAEFARRWVPFCKKYSIEPRAPEFYFSQKIDYLKDKIHPSFVKERRAMKRDYEEYKVRI
NALVAKAQKTPEEGWIMQDGTPWPGNNPRDHPGMIQVFLGETGARDFDGNELPRLVYVSREKRPGYQHHKKAGA
MNALVRVSAVLTNAPYILNLDCDHYVNNSKAVREAMCFMMDPSVGRDVCYVQFPQRFDGIDRSDRYANRNVVFFDV
NMKGLDGLQGPVYVGTGCCFYRQALYGYGPPSLPALPKSSVCSWCCCCCPKKKAEKSEKEMHRDSRREDLESAIFNLR
EIDNYDEYERSMLISQMSFEKSFGLSSVFIESTLMENGGVPESANPSTLIKEAIHVISCGYEEKTEWGKEIGWIYGSVTEDIL
TGFKMHCRGWRSIYCMPIRPAFKGSAPINLSDRLHQVLRWALGSVEIFLSRHCPLWYGYGGGRLKWLQRLSYINTIVYP
FTSLPLIAYCCLPAICLLTGKFIIPTLSNAATIWFLGLFISIIVTSVLELRWSGIGIEDWWRNEQFWVIGGVSAHLFAVFQGIL
KMIAGLDTNFTVTAKATDDTEFGELYVFKWTTVLIPPTSILVLNLVGVVAGFSDALNSGYESWGPLFGKVFFAMWVIM
HLYPFLKGLMGRQNRTPTIVVLWSVLLASVFSLLWVKIDPFIGSSETTTTNSCANFDC

>OS03G59340_OsCesAMDGAKSGKQCHVCQICGDGVGTAADGELFTACDVCGFPVCRPCYEYERKDGSQACPQCK
TKYKRHKGSPPILGDESDDVDADDASDVNYPTSGNQDHKHKIAERMLTWRMNSGRNDDIVHSKYDSGEIGHPKYDSG
EIPRIYIPSLTHSQISGEIPGASPDHMMSPVGNIGRRGHPFPYVNHSPNPSREFSGSLGNVAWKERVDGWKMKDKGAI
PMANGTSIAPSEGRGVGDIDASTDYNMEDALLNDETRQPLSRKVPISSSRINPYRMVIVLRLIVLCIFLHYRITNPVRNAY
PLWLLSVICEIWFALSWILDQFPKWSPINRETYLDRLALRYDREGEPSQLAPVDIFVSTVDPMKEPPLVTANTVLSILAVD
YPVDKVSCYVSDDGAAMLTFDALAETSEFARKWVPFCKKYSIEPRAPEWYFAQKIDYLKDKVQASFVKDRRAMKREYE
EFKVRVNALVAKAQKVPEEGWIMQDGTPWPGNNTRDHPGMIQVFLGHSGGLDTEGNELPRLVYVSREKRPGFQHH
KKAGAMNALVRVSAVLTNGQYLLNLDCDHYINNSKALREAMCFLMDPNLGRRVCYVQFPQRFDGIDRNDRYANRNT
VFFDINLRGLDGLQGPVYVGTGCVFNRTALYGYEPPIKQKRPGYFSSLCGGRKKTKKSKEKSTEKKKSHKHVDSSVPVFN
LEDIEEGIEGSGFDDEKSLLMSQMSLEKRFGQSSVFVASTLMEYGGVPQSATPESLLKEAIHVISCGYEDKSDWGTEIGW
IYGSVTEDILTGFKMHARGWRSIYCMPKRPAFKGSAPINLSDRLNQVLRWALGSVEILFSRHCPIWYGYGGRLKFLERFA
YINTTIYPLTSIPLLLYCILPAICLLTGKFIIPEISNFASIWFISLFLSIFATGILEMRWSGVGIDEWWRNEQFWVIGGISAHLFA
VFQGLLKVLAGIDTSFTVTSKASDEEGDFAELYMFKWTTLLIPPTTILIINLVGVVAGISYAINSGYQSWGPLFGKLFFAFW
VIVHLYPFLKGLMGRQNRTPTIVVVWAILLASIFSLLWVRIDPFTTRVTGPDTQKCGINC

>OS07G10770_OsCesAMDGDADAVKSGRHGSGQACQICGDGVGTTAEGDVFAACDVCGFPVCRPCYEYERKDGT
QACPQCKTKYKRHKGSPAIRGEEGEDTDADDVSDYNYPASGSADQKQKIADRMRSWRMNAGGGGDVGRPKYDSGE
IGLTKYDSGEIPRGYIPSVTNSQISGEIPGASPDHHMMSPTGNIGKRAPFPYVNHSPNPSREFSGSIGNVAWKERVDGW
KLKQDKGAIPMTNGTSIAPSEGRGVGDIDASTDYNMEDALLNDETRQPLSRKVPLPSSRINPYRMVIVLRLVVLSIFLHYR
ITNPVRNAYPLWLLSVICEIWFALSWILDQFPKWFPINRETYLDRLALRYDREGEPSQLAAVDIFVSTVDPMKEPPLVTA
NTVLSILAVDYPVDKVSCYVSDDGAAMLTFDALAETSEFARKWVPFVKKYNIEPRAPEWYFSQKIDYLKDKVHPSFVKD
RRAMKREYEEFKVRINGLVAKAQKVPEEGWIMQDGTPWPGNNTRDHPGMIQVFLGHSGGLDTEGNELPRLVYVSRE

KRPGFQHHKKAGAMNALVRVSAVLTNGQYMLNLDCDHYINNSKALREAMCFLMDPNLGRSVCYVQFPQRFDGIDR
NDRYANRNTVFFDINLRGLDGIQGPVYVGTGCVFNRTALYGYEPPIKQKKKGSFLSSLCGGRKKASKSKKKSSDKKKSNK
HVDSAVPVFNLEDIEEGVEGAGFDDEKSLLMSQMSLEKRFGQSAAFVASTLMEYGGVPQSATPESLLKEAIHVISCGYE
DKTEWGTEIGWIYGSVTEDILTGFKMHARGWRSIYCMPKRPAFKGSAPINLSDRLNQVLRWALGSVEILFSRHCPIWYG
YGGRLKFLERFAYINTTIYPLTSIPLLIYCVLPAICLLTGKFIIPEISNFASIWFISLFISIFATGILEMRWSGVGIDEWWRNEQF
WVIGGISAHLFAVFQGLLKVLAGIDTNFTVTSKASDEDGDFAELYMFKWTTLLIPPTTILIINLVGVVAGISYAINSGYQSW
GPLFGKLFFAFWVIVHLYPFLKGLMGRQNRTPTIVVVWAILLASIFSLLWVRIDPFTTRVTGPDTQTCGINC

>OS05G08370_OsCesAMAANAGMVAGSRNRNEFVMIRPDGDAPPPAKPGKSVNGQVCQICGDTVGVSATGDVFV
ACNECAFPVCRPCYEYERKEGNQCCPQCKTRYKRHKGSPRVQGDEEEEDVDDLDNEFNYKHGNGKGPEWQIQRQGE
DVDLSSSSRHEQHRIPRLTSGQQISGEIPDASPDRHSIRSGTSSYVDPSVPVPVRIVDPSKDLNSYGINSVDWQERVASW
RNKQDKNMMQVANKYPEARGGDMEGTGSNGEDMQMVDDARLPLSRIVPIPSNQLNLYRIVIILRLIILMFFFQYRVT
HPVRDAYGLWLVSVICEIWFALSWLLDQFPKWYPINRETYLDRLALRYDREGEPSQLAPIDVFVSTVDPLKEPPLITANTV
LSILAVDYPVDKVSCYVSDDGSAMLTFEALSETAEFARKWVPFCKKHNIEPRAPEFYFAQKIDYLKDKIQPSFVKERRAMK
REYEEFKVRINALVAKAQKVPEEGWTMADGTAWPGNNPRDHPGMIQVFLGHSGGLDTDGNELPRLVYVSREKRPGF
QHHKKAGAMNALIRVSAVLTNGAYLLNVDCDHYFNSSKALREAMCFMMDPALGRKTCYVQFPQRFDGIDLHDRYAN
RNIVFFDINMKGLDGIQGPVYVGTGCCFNRQALYGYDPVLTEADLEPNIVVKSCCGGRKKKSKSYMDSKNRMMKRTES
SAPIFNMEDIEEGIEGYEDERSVLMSQKRLEKRFGQSPIFIASTFMTQGGIPPSTNPASLLKEAIHVISCGYEDKTEWGKEI
GWIYGSVTEDILTGFKMHARGWISIYCMPPRPCFKGSAPINLSDRLNQVLRWALGSVEILLSRHCPIWYGYNGRLKLLER
LAYINTIVYPITSIPLIAYCVLPAICLLTNKFIIPEISNYAGMFFILLFASIFATGILELRWSGVGIEDWWRNEQFWVIGGTSAH
LFAVFQGLLKVLAGIDTNFTVTSKASDEDGDFAELYVFKWTSLLIPPTTVLVINLVGMVAGISYAINSGYQSWGPLFGKLF
FSIWVILHLYPFLKGLMGRQNRTPTIVIVWSILLASIFSLLWVKIDPFISPTQKAVALGQCGVNC

>OS07G24190_OsCesAMDEARQPLSRKVPIPSSQINPYRMVIIIRLVVLGFFFHYRVMHPVPDAFALWLISVICEIWFA
MSWILDQFPKWFPIERETYLDRLTLRFDKEGQTSQLAPIDFFVSTVDPLKEPPLVTANTVLSILAVDYPVDKVSCYVSDDG
AAMLTFEALSETSEFAKKWVPFCKKYSIEPRAPEWYFQQKIDYLKDKVAPYFVRERRAMKREYEEFKVRINALVAKAQKV
PEEGWTMQDGTPWPGNNVRDHPGMIQVFLGQSGGHDIEGNELPRLVYVSREKRPGYNHHKKAGAMNALVRVSAV
LTNAPYMLNLDCDHYINNSKAIKEAMCFMMDPLVGKKVCYVQFPQRFDGIDRHDRYANRNVVFFDINMKGLDGIQG
PIYVGTGCVFRRQALYGYDAPKTKKPPSRTCNCWPKWCICCCCFGDRKSKKKTTKPKTEKKKRSFFKRAENQSPAYALG
EIEEGAPGAENEKAGIVNQQKLEKKFGQSSVFVASTLLENGGTLKSASPASLLKEAIHVISCGYEDKTDWGKEIGWIYGSV
TEDILTGFKMHCHGWRSIYCIPKLPAFKGSAPLNLSDRLHQVLRWALGSVEIFFSNHCPLWYGYGGGLKCLERFSYINSIV
YPFTSIPLLAYCTLPAICLLTGKFITPELTNVASLWFMSLFICIFATGILEMRWSGVGIDDWWRNEQFWVIGGVSSHLFAL
FQGLLKVIAGIDTSFTVTSKGGDDEEFSELYTFKWTTLLIPPTTLLLLNFIGVVAGVSNAINNGYESWGPLFGKLFFAFWVI
VHLYPFLKGLVGRQNRTPTIVIVWSILLASIFSLLWVRIDPFLAKNDGPLLEECGLDCN

>OS03G62090_OsCesAMEASAGLVAGSHNRNELVVIRRDGEPGPKPVKHTNGQVCQICGDDVGLTPDGEPFVACN
ECAFPVCRDCYEYERREGTQNCPQCKTRFKRLKGCARVPGDEEEEDVDDLENEFNWRDKTDSQYVAESMLHGHMSY
GRGGDLDGVPQHFQPIPNVPLLTNGEMADDIPPEQHALVPSFMGGGGKRIHPLPYADPNLPVQPRSMDPSKDLAAY
GYGSVAWKERMESWKQKQERLHQMRNDGGGKDWDGDGDDADLPLMDEARQPLSRKIPISSSLVNPYRMIIIIRLVV
LGFFFHYRVMHPVPDAFALWLISVICEIWFAMSWILDQFPKWFPIERETYLDRLTLRFDKEGQQSQLAPVDFFVSTVDP
MKEPPLVTANTVLSILAVDYPVDKVSCYVSDDGAAMLTFEALSETSEFAKKWVPFCKRYSLEPRAPEWYFQQKIDYLKD
KVAPNFVRERRAMKREYEEFKVRINALVAKAQKVPEEGWTMQDGTPWPGNNVRDHPGMIQVFLGQSGGHDVEGN
ELPRLVYVSREKRPGYNHHKKAGAMNALVRVSAVLTNAPYMLNLDCDHYINNSKAIKEAMCFMMDPLVGKKVCYVQ

FPQRFDGIDRHDRYANRNVVFFDINMKGLDGIQGPIYVGTGCVFRRQALYGYDAPKSKKPPSRTCNCWPKWCICCCCF
GNRTNKKKTAKPKTEKKKRLFFKRAENQSPAYALGEIDEGAPGAENEKAGIVNQQKLEKKFGQSSVFVASTLLENGGTLK
SASPASLLKEAIHVISCGYEDKTDWGKEIGWIYGSVTEDILTGFKMHCHGWRSIYCIPKRAAFKGSAPLNLSDRLHQVLR
WALGSIEIFFSNHCPLWYGYGGGLKCLERFSYINSIVYPWTSIPLLAYCTLPAICLLTGKFITPELTNIASLWFMSLFICIFATG
ILEMRWSGVGIDDWWRNEQFWVIGGVSSHLFAVFQGLLKVIAGIDTSFTVTSKGGDDEEFSELYTFKWTTLLIPPTTLLL
LNFIGVVAGVSNAINNGYESWGPLFGKLFFAFWVIVHLYPFLKGLVGRQNRTPTIVIVWSILLASIFSLLWVRIDPFLAKN
DGPLLEECGLDCN

>OS07G14850_OsCesAMEASAGLVAGSHNRNELVVIRRDGGGGGGVGGRRAAEAKAACQICGDDVGEGPDGEPF
VACNECAFPVCRNCYDYERREGSQACPQCKTRFKRLKGCPRVAGDEEEDGVDDLEGEFGLDGREDDPQYIAESMLRA
NMSYGRGGDLQPFQPIPNVPLLTNGQMVDDIPPEQHALVPSYMGGGGGGGKRIHPLPFADPSVPVQPRSMDPSKDL
AAYGYGSVAWKERMEGWKQKQERMQQLRSEGGGDWDGDGDADLPLMDEARQPLSRKVPISSSRINPYRMIIIIRLV
VLGFFFHYRVMHPVNDAFALWLISVICEIWFAMSWILDQFPKWLPIERETYLDRLSLRFDKEGQPSQLAPVDFFVSTVD
PSKEPPLVTANTVLSILSVDYPVEKVSCYVSDDGAAMLTFEALSETSEFAKKWVPFCKKFNIEPRAPEWYFQQKIDYLKDK
VAASFVRERRAMKRDYEEFKVRINALVAKAQKVPEEGWTMQDGSPWPGNNVRDHPGMIQVFLGQSGGRDVEGNE
LPRLVYVSREKRPGYNHHKKAGAMNALVRVSAVLSNAPYLLNLDCDHYINNSKAIREAMCFMMDPLVGKKVCYVQFP
QRFDGIDRHDRYANRNVVFFDINMKGLDGIQGPIYVGTGCVFRRQALYGYDAPKTKKPPSRTCNCWPKWCCCCCCG
NRHTKKKTTKPKPEKKKRLFFKKAENQSPAYALGEIEEGAPGAETDKAGIVNQQKLEKKFGQSSVFVASTLLENGGTLKS
ASPASLLKEAIHVISCGYEDKTDWGKEIGWIYGSITEDILTGFKMHCHGWRSIYCIPKRPAFKGSAPLNLSDRLHQVLRW
ALGSVEIFFSKHCPLWYGYGGGLKFLERFSYINSIVYPWTSIPLLAYCTLPAICLLTGKFITPELTNVASLWFMSLFICIFVTGI
LEMRWSGVAIDDWWRNEQFWVIGGVSSHLFAVFQGLLKVLAGVDTSFTVTSKAGDDEEFSELYTFKWTTLLIPPTTLLL
LNFIGVVAGVSNAINNGYESWGPLFGKLFFAFWVIVHLYPFLKGLVGRQNRTPTIVIVWSILLASIFSLLWVRIDPFLAKN
NGPLLEECGLDCN

>OS09G25490_OsCesAMEASAGLVAGSHNRNELVLIRGHEEPKPLRALSGQVCEICGDEVGRTVDGDLFVACNECGF
PVCRPCYEYERREGTQNCPQCKTRYKRLKGSPRVPGDEDEEDIDDLEHEFNIDDEKQKQLQQDQDGMQNSHITEAML
HGKMSYGRGPDDGDGNSTPLPPIITGARSVPVSGEFPISNSHGHGEFSSSLHKRIHPYPVSEPGSAKWDEKKEVSWKER
MDDWKSKQGIVAGGAPDPDDYDADVPLNDEARQPLSRKVSIASSKVNPYRMVIILRLVVLGFFLRYRILHPVPDAIPLW
LTSIICEIWFAVSWILDQFPKWYPIDRETYLDRLSLRYEREGEPSLLSAVDLFVSTVDPLKEPPLVTANTVLSILAVDYPVDK
VSCYVSDDGASMLTFESLSETAEFARKWVPFCKKFSIEPRAPEFYFSQKVDYLKDKVHPNFVQERRAMKREYEEFKVRIN
ALVAKAQKVPAEGWIMKDGTPWPGNNTRDHPGMIQVFLGHSGGHDTEGNELPRLVYVSREKRPGFQHHKKAGAM
NALIRVSAVLTNAPFMLNLDCDHYINNSKAIREAMCFLMDPQVGRKVCYVQFPQRFDGIDVHDRYANRNTVFFDINM
KGLDGIQGPVYVGTGCVFRRQALYGYNPPKGPKRPKMVTCDCCPCFGRKKRKHGKDGLPEAVAADGGMDSDKEML
MSQMNFEKRFGQSAAFVTSTLMEEGGVPPSSSPAALLKEAIHVISCGYEDKTDWGLELGWIYGSITEDILTGFKMHCRG
WRSVYCMPKRAAFKGSAPINLSDRLNQVLRWALGSVEIFFSRHSPLLYGYKNGNLKWLERFSYINTTIYPFTSLPLLAYCT
LPAVCLLTGKFIMPPISTFASLFFIALFISIFATGILEMRWSGVSIEEWWRNEQFWVIGGVSAHLFAVVQGLLKVLAGIDT
NFTVTSKATGDEDDEFAELYAFKWTTLLIPPTTLLILNIIGVVAGVSDAINNGSEAWGPLFGKLFFAFWVIVHLYPFLKGL
MGRQNRTPTIVVIWSVLLASIFSLLWVRIDPFTIKARGPDVRQCGINC

>OS10G32980_OsCesAMDTASVTGGEHKGKEKTCRVCGEEVAAREDGKPFVACAECGFPVCKPCYEYERSEGTQCC
PQCNTRYKRHKGCPRVEGDEDDGGDMDDFEEEFQIKSPTKQKPPHEPVNFDVYSENGEQPAQKWRPGGPALSSFTG
SVAGKDLEQEREMEGGMEWKDRIDKWKTKQEKRGKLNRDDSDDDDDKNDDEYMLLAEARQPLWRKVPIPSSKINP
YRIVIVLRLVVLCFFLKFRITTPAMDAVPLWLASVICELWFALSWILDQLPKWSPVTRETYLDRLALRYERDGEPCRLAPID

FFVSTVDPLKEPPIITANTVLSILAVDYPVDRVSCYVSDDGASMLLFDTLSETAEFARRWVPFCKKFTIEPRAPEFYFSQKID
YLKDKVQPTFVKERRAMKREYEEFKVRINALVAKAQKKPEEGWVMQDGTPWPGNNTRDHPGMIQVYLGSQGALDV
EGSELPRLVYVSREKRPGYNHHKKAGAMNSLVRVSAVLTNAPFILNLDCDHYVNNSKAVREAMCFLMDKQLGKKLCYV
QFPQRFDGIDRHDRYANRNTVFFDINMKGLDGIQGPVYVGTGTVFNRQALYGYDPPRPEKRPKMTCDCWPSWCCCC
CCFGGGKRGKSHKNKKGGGGGEGGGLDEPRRGLLGFYKKRSKKDKLGGGAASLAGGKKGYRKHQRGFELEEIEEGLEG
YDELERSSLMSQKSFEKRFGQSPVFIASTLVEDGGLPQGAAADPAALIKEAIHVISCGYEEKTEWGKEIGWIYGSVTEDILT
GFKMHCRGWKSVYCTPARAAFKGSAPINLSDRLHQVLRWALGSVEIFMSRHCPLWYAYGGRLKWLERFAYTNTIVYPF
TSIPLLAYCTIPAVCLLTGKFIIPTLNNLASIWFIALFLSIIATGVLELRWSGVSIEDWWRNEQFWVIGGVSAHLFAVFQGLL
KVLGGVDTNFTVTSKAAADETDAFGELYLFKWTTLLVPPTTLIIINMVGIVAGVSDAVNNGYGSWGPLFGKLFFSFWVIL
HLYPFLKGLMGRQNRTPTIVVLWSILLASIFSLVWVRIDPFIPKPKGPVLKPCGVSC

>OS06G39970_OsCesAMDGESPEIMPVECPDPEPASSESGDDHDIPEPLSSRLSVPSGELNLYRAAVALRLVLLAAFFR
YRVTRPVADAHALWVTSVACELWLAASWLIAQLPKLSPANRVTYLDRLASRYEKGGEASRLAGVDVFVAAADAAREPP
LATANTVLSVLAADYPAGGVACYVHDDGADMLVFESLFEAAGFARRWIPFCRRHGVEPRAPELYFARGVDYLRDRAAP
SFVKDRRAMKREYEEFKVRMNHLAARARKVPEEGWIMSDGTPWPGNNSRDHPAMIQVLLGHPGDRDVDGGELPRL
FYVSREKRPGFRHHGKAGAMNALLRVSAVLTNGAYVLNLDCDHCVNNSSALREAMCFMMDPVAGNRTCFVQFALR
DSGGGDSVFFDIEMKCLDGIQGPVYVGSGCCFSRKALYGFEPAAAADDGDDMDTAADWRRMCCFGRGKRMNAMR
RSMSAVPLLDSEDDSDEQEEEEAAGRRRRLRAYRAALERHFGQSPAFIASAFEEQGRRRGGDGGSPDATVAPARSLLKE
AIHVVSCAFEERTRWGKEIGWMYGGGVATGFRMHARGWSSAYCSPARPAFRRYARASPADVLAGASRRAVAAMGIL
LSRRHSPVWAGRRLGLLQRLGYVARASYPLASLPLTVYCALPAVCLLTGKSTFPSDVSYYDGVLLILLLFSVAASVALELRW
SRVPLRAWWRDEKLWMVTATSASLAAVFQGILSACTGIDVAFSTETAASPPKRPAAGNDDGEEEAALASEITMRWTN
LLVAPTSVVVANLAGVVAAVAYGVDHGYYQSWGALGAKLALAGWVVAHLQGFLRGLLAPRDRAPPTIAVLWSVVFVS
VASLLWVHAASFSAPTAAPTTEQPIL

>GRMZM2G103972_P01MAAAVTRRVGLHVEATNGGADDESRRNSSAADHSPVAKRINDAAANAKRNDVWVAA
QEGEMPAAAGNSSQPPLLFRTMKVKGSILHPYRFVILLRLVAIVAFFIWRIRNRNRDGVWLWAMSMVGDVWFGFSW
VLNQLPKLNPIKRVPDLAAIRDQYEQPSASGGESNNKLPGIDVFVTTVDPVDEPILYTVNSVLSILATDYPVEKYACYLSDD
GGTLVHYEAMLEVASFARLWAPFCRKHSVEPRAPESYFGVKRRQPYTGSVQGEFTSDHRRMRREYEEFKVRIDSLFSTV
CQRSQAYNRKHAKDDEAGMVMKATWMADGTQWPGTWIEQAENHRKGHHAGIVKVVLNHPGHKPELGSPASIDN
PFDFSNTDTRLPMLVYMSREKRTGYNHQKKAGAMNAMLRVSALLSNAPFLINFDCDHYVNNSQAFRASMCFMLDPR
DGRNTAFVQFPQRFDGVDPTDRYANHNRVFFDGTMLSLNGLQGPSYLGTGTMFRRAALYGMEPPRWRTTGSVKVID
DDDDHKGKEYGRSTLFRNAVLDDAANQERSITPVFLDDDETTTISSEVASLMTCAYEDGTTWGRDVGWVYNIATEDVV
TGFRMHRQGWRSMYCSVEPAAFRGTAPINLTERLLQVLRWSGGSLEMFFSHSNAFLAGARMHPLQRVAYLNMSTYP
VVTVFILAYNLFPLMWLVSERYYIQRPFGTYVLYLVATIAMIHVIGMFEVRWAGITLLDWCRNEQFYMIGATGVYPTAV
LYMALKLVTGKSIHFRLTSKQTEACSGGDKFADLYVVRWVPLLVPTIAVLAVNVAAVGVAVGKAATWGLLTQQAQHAL
LGMVFNVWILVLLYPFALGVMGRWGKRPAILFGVLVMAIGAVAVVYISFRATYYPTGWSEMETTSALGKAESFF

>GRMZM2G113432_P01MAPKAPANNGAVVVAGGHHGGSTDVGGVAGGATTTTVPTSVAANGNGKQHGTRKQ
RRPAAVVVSPMDKYWTPVDDKEAAEAVDDGGEDGRRPLLFRTYKVKGILLHPYRLLTLLRLIAIILFFIWRIRHPHADGM
WLWWISIVGDFWFGVTWLLNQVAKLNPTKRVPDLSLLRQQFDLPDGNSNLPRLDVFINTVDPINEPMIYTMNSILSILA
VDYPIDRTATYLSDDGGSIIHYEGLLETANFATLWVPFCRKHSIEPRAPESYFAVKSRPYTGNVPDEFADDHRRMSKEYD
EFKVRLDALFTKIPERSDAHNAEAKEGVKATWMADGTQWPGTWFDPAENHKKGQHAGIVKVMLNHPGDEPRFGGP
ASAETPLDFSAVDVRLPMLVYISREKSPSHDHQKKAGAMNVQLRISALLTNAPFIINFDGDHYVNNSQAFRAAMCFML

DRRDGENTAFVQFPQRFDDVDPTDRYCNHNRVFFDATLLGLNGIQGPSYVGTGCMFRRIAVYGIDPPRWRTDAFKLV
DNPSKFGSSMLFINSIPSAANQEWSMASPPAHEESVMEELNNVMKCAYEEGTEFGKEIGWVYNIATEDVVTGFRVHR
TGWRSMYCRMEPDAFRGTAPINLTERLCQILRWSGGSLEMFFSHCPLLAGRRLNLMQRIAYTNMTAYPISSVFLVFYLL
FPVIWIFRGEFYIQKPFPTYVLYLVVIIAMTELIGMVEIKWAGLTLLDWIRNEQFYIIGATAVYPLATLHIVLKLVLRGNGVS
FKLTAKQATSAVNEKYAEMYVVQWTPLLIPTIAVIAVNVGAIGAAIGKAVVGGWSLLQMADASLGLVFNAWILLLIYPF
ALGVMGRWSKRPYILFVLFMIGFAVVAAVVVAIHAARTGSVRFHSGHSGGASFPTSWGF

>GRMZM2G339645_P01MASPAPAGDAVYAANGGLTDPLLVSANGHGAAARKAGHGARGRYWVASDKAERRAA
KESGGEDGRALLFRKYKVKGALLHPYRLLIIIRLVAVLAFFAWRIRHNKSDIMWFWTMSIVGDVWFGFSWLLNQLPKFN
PVKTIPDLAALQRHFGYPDGGASRLPGIDVFVTTADPIDEPILYTMNCVLSILSVDYPVDRLACYLSDDSGALVLYEALAEV
GKFAPLWVPFCRKYSIEPRAPESYFEHVAPPQAGRVTQEFLNDYRRVQMEYDEFKARLDNLPDAIRKRSDVYNSVRDA
GGAQKATWMANGTQWPGTWIDPAENHRKGHHAPIAKVVLNHPSRGQHPITESNPSIATTDERLPMLVYVSREKNP
GYDHNKKAGALNAQLRASALLSNAQLIINFDCDHYINNSQALSSAVCFMLDQRDGDNTAFVQFPQRFDNVDPTDRYG
NHNRVFFDGTMLALNGLQGPSYLGTGCMFRRLALYGIDPPHCRAENITAEASRFGNSTIFLDSVSKALKNDRTITPPPID
DTFLAELERVVTCSYDKGTDWGKGVGYIYDIATEDIVTGFRIHGQGWRSMYCTMEHDAFCGVAPINLTERLHQIVRWS
GGSLEMFFSHNNPFIGGRRIQPLQRVSYLNMTVYPVTSVFILIYALSPVMWLIPDEVYIQRPFTRYVVYLLVIIVMIHMIG
WLEIKWAGVTWLDYWRNEQFFMIGSTSAYPMAVLHMAVNLLTKKGIHFRVTSKQTAADDNDKFADLYDFRWVPML
IPTMAVLICNVGAIGVALGKTVVYIGTWTAAKKMHAALGLLFNIWIMFLLYPFALAIMGRWAKRPIILVVLLPVVFALVA
LLYVGIHILLAGLIPF

>GRMZM2G367267_P01MTMTSPQALSAGAVTVEDDGGLAVPLLADGSGATKTEEECGAEAEGKYWVAADEAER
QAVTDCGAEDGRALLFRTYKLRGAILHPYRALILVRLVAVLLFFVWRIRNSASNVMWFWATSVAGDAWFGFSWLLNQ
LPKFSPVKSVPDLAALRRHYDLLPADDGAASKLPGVDVFVTTADSVDEPVLYTMNSILSILATDYPADRLACYVSDDSGA
LVLYEALVEAAKFARLWVPFCRKHCVEPRAPERYFETEPQGGRASQEFVNDYKRVQMEYDEFKVRLGNLPDTIRKRSGT
GSMRASEGDAQGTWMADGMQWPGTWMDPTEKHRKGHHAGIVKVVLDHHPSRGHHGPRAGAGAGAENKQSAD
DFGAAAGLRLPMLVYVSREKDPNYDHNKKAGALNAQLRVSALLSNAQFVINFDCDHYVNNSQALRAAVCLMLDQRE
GGDTAFVQFPQRFDNVDPTDRYGNHNRVFFDGTMLALNGLQGPSYLGTGCMFRRIALYGVDPPHCRQQQLESVAPE
PASKYGKSTALIHSVSEAMGERERLTTPPPVPPLDVEMVVAASYDGGTDWGKGVGYIYGIATEDIVTGFRIHGKGWRS
MYCTMRRDAFRGTAPINLTERLHQIVRWSGGSLEMFFSRNNPLVGGQRLKLLQRVSYLNMTVYPVTSLFILLYALCPV
MWLVPEEVHIQRPFTRYVVYLLITILMIHMIGWLEMKWSGVAWLDHWRNEQFFMIGSTSAYPIALWHMAKKLLTRK
GIHFRVTSKQTTAGTDDKFADLYEMRWTPMLVPTAFVLVANVGAVGVAMGKALVYMGVWTVAQKTHAALGLLFNV
WIMLLLYPFALAIMGRWAKRPIILLVLLPAVFAVVGTIYVALHFLLANVIPI

>GRMZM2G110145_P01MAAGQQQASGGAKHGCVCGFPVCACAGAAAVASAASSADMDRVAVAATEGQIGAVN
DESWIAVDLSDDGLSADGADPGVALEDRPVFRTEKIKGVLLHPYRVLIFVRLIAFTLFVIWRISHRNPDALWLWVTSIAGE
FWFGFSWLLDQLPKLNPINRVPDLAALRQRFDRAGGGAGGGTSLLPGLDVFVTTADPFKEPILSTANSVLSILAADYPVE
RNTCYLSDDSGMLLTYEAMAEAAKFATVWVPFCRKHGIEPRGPESYFDLKSHPYMGRSQEDFVNDRRRVRKDYDEFK
ARINGLDHDIKQRSDAYNAARGLKDGEPRATWMADGTQWEGTWVEPSENHRKGDHAGIVLVLLNHPSHSRQLGPP
ASADNPLDLSMVDVRLPMLVYVSREKRPGHNHQKKAGAMNALTRCSAVLSNSPFILNLDCDHYINNSQALRAGICFM
LGRDSDTVAFVQFPQRFEGVDPTDLYANHNRIFFDGTLRALDGMQGPIYVGTGCLFRRITLYGFDPPRINVGGPCFPAL
GGMFAKAKYEKPGLELTTTKAAVAKGKHGFLPMPKKSYGKSDAFADTIPMASHPSPFAAASAASVVADEATIAEAVAV
CAAAYEKKTGWGSDIGWVYGTVTEDVVTGYRMHIKGWRSRYCSIYPHAFIGTAPINLTERLFQVLRWSTGSLEIFFSRN
NPLFGSTFLHPLQRVAYINITTYPFTAIFLIFYTTVPALSFVTGHFIVQRPTTMFYVYLAIVLGTLLILAVLEVKWAGVTVFE

WFRNGQFWMTASCSAYLAAVCQVLVKVVFRRDISFKLTSKQPAGDEKKDPYADLYVVRWTWLMVTPIIIILVNIIGSAV
AFAKVLDGEWTHWLKVAGGVFFNFWVLFHLYPFAKGILGRHGKTPVVVLVWWAFTFVITAVLYINIPHIHGPGGKHG
GAIGRHGGDAHHHGKKFDGYYLWP

>GRMZM2G122277_P01MAPGGRRSNGETPTGQQQQQQQADGRRGCACGGFPVCACAGAAAVASAASSADMD
RVAVAATEGQIGAVNDESWVAVDLSDDGLSSAADPGAVALEERPVFRTEKIKGVLLHPYRVLIFVRLIAFTLFVIWRISHR
NPDALWLWVTSIAGEFWFGFSWLLDQLPKLNPINRVPDLGALRQRFDRADGTSRLPGLDIFVTTADPFKEPILSTANSIL
SILAADYPVERNTCYLSDDSGMLLTYEAMAEAAKFATVWVPFCRKHGIEPRGPESYFELKSHPYMGRSQEDFVNDRRR
VRRDYDEFKARINGLENDIRQRSDAYNAARGLKDGEPRATWMADGTQWEGTWVEPSENHRKGDHAGIVLVLLNHPS
HSRQLGPPASADNPLDLSMVDVRLPMLVYVSREKRPGHNHQKKAGAMNALTRCSAVLSNSPFILNLDCDHYINNSQA
LRAGICFMLGRDSDTVAFVQFPQRFEGVDPTDLYANHNRIFFDGTLRALDGMQGPIYVGTGCLFRRITLYGFDPPRINV
GGPCFPSLGGMFAKTKYEKPGLELTTKAAVAKGKHGFLPMPKKSYGKSDAFADTIPMASHPSPFAAAAAVVAEEATIAE
AVAVCAAAYEKKTGWGSDIGWVYGTVTEDVVTGYRMHIKGWRSRYCSIYPHAFIGTAPINLTERLFQVLRWSTGSLEIF
FSRNNPLFGSTFLHPLQRVAYINITTYPFTAIFLIFYTTVPALSFVTGHFIVQRPTTMFYVYLAIVLGTLLILAVLEVKWAGVT
VFEWFRNGQFWMTASCSAYLAAVCQVLVKVVFRRDISFKLTSKQPAGDEKKDPYADLYVVRWTWLMVTPIIIILVNIIG
SAVAFAKVLDGEWTHWLKVAGGVFFNFWVLFHLYPFAKGILGRHGKTPVVVLVWWAFTFVITAVLYINIPHIHGPGGK
HGGAIGKHGAAHHGKKFDGYYLWP

>GRMZM2G164761_P01MSTTYTTKSNGTTSDDNKWPKDHVKKSASVDRPPLVRTTKLSTITIKLYRLMIILRMGIFVL
FFKWRIGTALVMISSTGTDDKSTVLGMWMVSMAGELWFALMWVLDQVPKMQPVRRVVYLAALDEPMLPAMDVF
VTTVDTEKEPPLVTVNTILSILAADYPAEKLTCYVSDDGGALLTRDAVAEAARFSALWVPFCRKHAVEPRNPEAYFSPGA
SNGFKAWRADYKGTAWPELARDRRRVRREYEELRLRIDALQAGGRAAVDAVAADRSCWRRGAAEDHAGAVELLVD
NPGPGSTPRLGVSGTVDGVSNLLDLSSVDVRVPALVYMCREKRRGRVNHGKAGALNALLRASAVLSNAPFILNLDCDH
YVNNSQALRAGVCHMLDGEGGNGNDVAFVQFPQRFDGVDPADRYANHNRVFFDCTELGLDGLQGPIYVGTGCVFR
RSALYGVDPPLWRPQGDDAGKGAANGIETGKLGVSTPFLRSVYAVLTNQSDQWDTVSISSPPCSFDAAAIGEATALVS
CGYEDRTAWGRDIGWIYGTVTEDVATGFCMHRRGWRSSYCATAPDAFRGTAPINLTDRLYQVLRWAAGSLEIFFSRN
NALLAGRRLHPLQRLAYLNTTVYPFTSIFLIAYCGLFPAIPLVTGNGATTGAFFSIIIRPPSATYIAFVAALMLTLAVVAVLEV
RWSGISLGDWWRNQQFWMVSATSAYLAAAVQVALKIAAGKEISFKLTSKQRATSTVASVKDRFAELYAVKWTVLMV
PTAVVLAVNLTSIVAAMEGGSWRDGPMAVFALAFNAYVVVHLYPFALGLMGRWSNTLSPLLLLIAFFTVRLLCIVLYVQ
ML

>GRMZM2G044269_P01MSNPPPKKAIRNPGGGAGGPAGGPRGPAGNTVKFARRTSSGRYVSLSREDIDMEGELAA
DYTNYTVQIPPTPDNQPMMDPASVAMKAEEQYVSNSLFTGGFNSVTRAHLMDKVIESEVTHPQMAGSKGSRCAMP
ACDGKVMRNERGEDIDPCECRFKICRDCYLDAQKEGCLCPGCKEHYKIGEYADDDPNDASSGKHYLPGPGGGMMNN
SKSLLARNQNGEFDHNRWLFESSGTYGYGNAFWPKGGMYDDDLDDEGGPGGGGGDGMLPEQKPFKPLTRKIPMPT
SIISPYRIFIVIRMFVLIFYLTWRVRNPNMEALWLWGMSIVCELWFAFSWLLDMLPKVNPVNRSTDLAVLKEKFETPSPS
NPHGRSDLPGLDVFVSTADPDKEPVLTTATTILSILAADYPVEKLACYVSDDGGALLTFEAMAEAASFANIWVPFCKKHD
IEPRQPDSYFSIKGDPTKGKRRSDFVKDRRKVKREFDEFKVRINGLPDSIRRRSDAFNAREDMKMLKHLRETGADPAEQ
PKVKKATWMADGTHWPGTWAVSAPDHAKGNHAGILQVMLKPPSPDPLYGMHDEEQLIDFSDVDIRLPMLVYMSRE
KRPGYDHNKKAGAMNALVRCSAVMSNGPFILNFDCDHYINYAQAIREAMCFVMDRGGERIAYIQFPQRFEGIDPSDR
YANNNTVFFDGNMRALDGLQGPMYVGTGCMFRRFALYGFDPPRTTEYTGWLFKKKKVTTFGKADQGETDTQSLNSK
GAEDFDAELTSMLVPRRFGNSSALMASIPVAEFQARPLADHPAVLHGRPPGSLTVPRPPLDPPTVAEAVSVISCWYEDK
TEWGDRVGWIYGSVTEDVVSGYRMHNRGWRSVYCIPKRDAFLGTAPINLTDRLHQVLRWATGSVEIFFSRNNAFLAS

RRLMFLQRVAYLNVGIYPFTSIFLLVYCFIPALSLFSGFFIVQTLNVAFLCYLLTITVTLIALGVLEVKWSGIELEDWWRNEQ
FWLISGTSAHLYAVVQGLLKVMAGIEISFTLTAKAAVDDNEDIYADLYVVKWSSLLIPPITIGMINVIAIAFAFARTVYSDN
PRWGKFIGGGFFSFWVLAHLYPFAKGLMGRRGKTPTIVFVWSGLISITISLLWVAISPPEASAGGRGAGFQFP

>GRMZM2G436299_P01MASKGILKNSGSRRMPPQGAGSRPPTAPTSAPTVVFGRRTDSGRFISYSRDDLDSEISSVD
FQDYHVHIPMTPDNQPMDEDDSGTARAEDQRYVSGSLFTGGFNTVTRAHVMDKPDGDAAGRRGGGGAKGPSACM
VEGCDARAMRDARGDDVLPCECDFRVCVDCFTDAVKAGAGVCPGCKEPYKNTEWEGLGAGAAEEVTRALSLPRGPA
GANGHRMERRLSLVKQANVNQSGEFDHNLWLFQTKGTYGYGNAIWPQDEADDDTDGGAPAGHPKELLTKPWRPLT
RKLRIPAAVISPYRLLVLIRLVALAFFLMWRIKHQNEDAIWLWGMSIVCELWFAFSWVLDQLPKLCPINRATDLSVLKEK
FETPTPNNPTGKSDLPGVDIFVSTADPEKEPVLVTANTILSILAADYPVEKLACYLSDDGGALLTFEAMAEAASFANLWVP
FCRKHDIEPRNPDSYFNLRRDPFKNKVKPDFVKDRRRVKREYDEFKVRVNGLPDAIRRRSDAYHAREEIQAMNLQREKL
KGGGDEPPFEPVKIPKATWMADGTHWPGTWLQPSQDHARGDHAGIIQVMLKPPSDMPTTMYDAASKTPLDLAGV
DTRLPMLVYMSREKRPGYDHNKKAGAMNALVRASAIMSNGPFILNLDCDHYIYNSKALREGMCFMMDRGGDRLCYV
QFPQRFEGIDPSDRYANHNTVFFDVNMRALDGLQGPVYVGTGCLFRRIALYGFDPPRSKDHSPGFCSCCLPRRRKPSA
ASREETMALRMGGFDGDSMDLATFPKKFGNSSFLIDSIPVAEFQGRPLADHPSVKNGRPPGALTIPREMLDASIVAEAI
SVVSCWYEEKTEWGIRVGWIYGSVTEDVVTGYRMHNRGWKSVYCVTQRDAFRGTAPINLTDRLHQVLRWATGSVEI
FFSRNNALFASSKMKVLQRIAYLNVGIYPFTSIFLIVYCFLPALSLFSGQFIVQTLNVTFLTYLLIITVTLCLLAMLEIKWSGIAL
EEWWRNEQFWLIGGTSAHLAAVLQGLLKVVAGIEISFTLTSKQVGDDVEDEFAELYIVKWTSLMIPPLTIIMINLVAIAV
GFSRTIYSTIPQWSKLLGGVFFSFWVLAHLYPFAKGLMGRRGRTPTIVYVWSGLVSITISLLWIAIKPPSQAANSQFGGSF
SFP

>GRMZM2G052149_P01MASTGGGGGLRHSNSSRLSRMSFSGGGSDDARAQAAAAPGGDRPMVTFARRTRSGRY
VSYSRDDLDSELGAAGVDMSPDRDEQFASYHVHIPATPDNQPMDPAISARVEEQYVSNSLFTGGFNSVTRAHLMDKV
IDSEASHPQMAGARGSSCAVNGCDANVMSDERGDDILPCECDFKICAECFADAVKNAGAICPGCKEPYKNTELDDVV
GAAADATAGGRPTLSLPPPPGAAASRMERRLSIMRSQKAMTRSQTGDWDHNRWLFETKGTYGYGNAIWPKENEVD
AAGGLGGGGADGQPAEFTTKPWRPLTRKLSIPAGVLSPYRLLILIRMAVLGLFLTWRIKHKNEDAIWLWGMSVVCELW
FGFSWLLDQLPKLCPVNRATDLAVLKDKFETPTPSNPTGRSDLPGLDIFVSTADPEKEPPLVTANTILSILAADYPVEKLSC
YVSDDGGALLTFEAMAEAASFANMWVPFCRKHNIEPRNPDSYFNLKKDPYKNKVRQDFVKDRRRVKREYDEFKVRIN
GLPDSIRRRSDAYHAREEIKAMKRQREAALDDAVEPVKIPKATWMADGTHWPGTWIQPSAEHTRGDHAGIIQVMLK
PPSDDPLYGSTGDEGRPLDFTEVDIRLPMLVYVSREKRPGYDHNKKAGAMNALVRSSAVMSNGPFILNLDCDHYVYNS
QAFREGMCFMMDRGGDRIGYVQFPQRFEGIDPSDRYANHNTVFFDVNMRALDGLMGPVYVGTGCLFRRVALYGFD
PPRSKEHGGCCSCCFPQRRKIKASAAAPEETRALRMADFDEDEMNMSSFPKKFGNSSFLIDSIPIAEFQGRPLADHPGV
KNGRPPGALTVPRDLLDASTVAEAVSVISCWYEDKTEWGHRVGWIYGSVTEDVVTGYRMHNRGWKSVYCVTKRDAF
RGTAPINLTDRLHQVLRWATGSVEIFFSRNNALLASRRMKFLQRIAYLNVGIYPFTSIFLIVYCFLPALSLFSGQFIVKTLNV
TFLTYLLVITLTLCLLAVLEIKWSGISLEEWWRNEQFWLIGGTSAHLAAVLQGLLKVVAGIEISFTLTSKSGGDDVDDEFAD
LYIVKWTSLMIPPIVIMMVNLIGIAVGFSRTIYSEIPQWSKLLGGVFFSFWVLAHLYPFAKGLMGRRGRTPTIVFVWAGL
LSITISLLWVAINPPSQNQQIGGSFTFP

>GRMZM2G061764_P01MHEGLGRWGLDSLKYGCHSNYTVRMPPTPDNQPYSVGGGAPLSVCAGGTKPDDLPLPP
SKLVNRRGGACADDGLAGASGKMDRRLSTARVPAPSKSLLVRSQTGDFDHNRWLFETRGTYGIGNAYWPQDSSAYA
DDEDGGVGSDPVKMEDLVDKPWKPLSRKVPIPPGILSPYRLLVLVRFISLFLFLIWRATNPNLDALWLWGISIVCEFWFA
FSWLLDQMPKLNPINRAVDLSALREKFESPTPSNPTGRSDLPGLDVFISTADPYKEPPLTTANSLLSILGTEYPVEKLFVYIS
DDGGALLTFEAMAEACEFAKVWVPFCRKHSIEPRNPDAYFNQKGDPTKGKKRPDFVKDRRWIKREYDEFKVRINGLAD

LIRRRANAMNARERKIARDKAAAASSDAPVADASTVKATWMADGTHWPGTWLDSAPDHAKGDHASIVQVMIKNP
HYDVVHGDAGSHPYLDFTGVDVRIPMFVYLSREKRPGYDHNKKAGAMNAMVRASAILSNGPFMLNFDCDHYIFNCM
AIREAMCYMLDRGGDRICYIQFPQRFEGIDPSDRYANHNTVFFDGNMRALDGLQGPMYVGTGCLFRRYAIYGFNPPR
TNEYRGIYGQVKVPIDPHGHHAPGAAEELRPLSEHPDHEAPQRFGKSKMFIETIAVAEYQGRPLQDHPSVQNGRPPGA
LLMPRPPLDAATVAESVAMISCWYEDGTEWGQRVGWIYGSVTEDVVTGYRMHNRGWRSVYCITRRDAFRGTAPINL
TDRLHQVLRWATGSVEIFFSKNNALLASQRLKFLQRLSYLNVGIYPFTSLFLIMYCLLPALSLFSGQFIVATLDPTFLCYLLLI
TITLMLLCLLEVKWSGIGLEEWWRNEQFWVIGGTSAHLAAVLQGLLKVIAGIEISFTLTAKAAAEDDDDPFAELYLVKWT
SLFIPPLAVIGINIIALVVGVSRAVYAEIPQYSKLLGGGFFSFWVLAHYYPFAKGLMGRRGRTPTLVYVWAGLISITVSLLWI
TISPPDDRITQGGVDV

>GRMZM2G015886_P01MSRRLSLPAGSQVTVTVSPTRGKAESPGDGVIKRSGGRIGLTSPAPRHSLGGAGSSTIPTV
QLSPVRRSGGSRYASLDGGAGADDSAEFVHYTVHIPPTPERTVAASADSVDAPAPTAYDEDGGAAGVRAQRSYISGTIF
TGGLNQATRGHVLNTSANSAAVAASANMSCKMRGCDMPAFLSSGAGGGPCDCGFMICRECYADCVAAAGNCPGC
KEPYSAGSDTDDDGEDDEAVSSSEERDQLPLTSMAKRFSLIHSMKMPSNNGGGGGKPAEFDHARWLFETKGTYGYG
NALWPKDGHGGGGGGGGFSGFEEPPNFGSRCRRPLTRKTSISQAILSPYRLLIAIRLVALGFFLTWRIRHPNPEAVWLW
ALSVTCEVWFAFSWLLDSLPKLCPIHRAADLDVLAERFELPTARNPKGHSDLPGIDVFVSTADPEKEPPLVTANTILSILAA
DYPVEKLACYLSDDGGALLTFEALAETASFARTWVPFCRKHGVEPRCPEAYFGQKRDFLRNKVRVDFVRERRKVKREYD
EFKVRVNSLPEAIRRRSDAYNAGEELRARRRQQEEAMAAGTILGALPEAAGAVKATWMSDGSQWPGTWLTSAPDHS
RGDHAGIIQAMLAPPTSEPVLGAEPAESGGLIDTTGVDIRLPMLVYVSREKRPGYDHNKKAGAMNALVRTSAIMSNGP
FILNLDCDHYVHNSAALREGMCFMLDRGGDRVCYVQFPQRFEGIDPNDRYANHNLVFFDVAMRAMDGLQGPMYV
GTGCVFRRTALYGFSPPRATEHHGWLGRRKIKLLLRKPTMGKKTDRENNSDKEMMLPPIEDDAFQQLDDIESSALLPRR
FGSSATFVASIPVAEYQGRLLQDTPGAHQGRPAGALAVPREPLDADTVAEAISVISCFYEDKTEWGRRIGWIYGSVTED
VVTGYRMHNRGWRSVYCVTRRDAFRGTAPINLTDRLHQVLRWATGSVEIFFSRNNALFASPRMKFLQRVAYFNVGM
YPFTSIFLLVYCVLPAVSLFSGKFIVQSLNATFLALLLVITITLCLLALLEIKWSGITLHEWWRNEQFWVIGGTSAHPAAVLQ
GLLKVIAGVDISFTLTSKPGTGDDGEEDAFAELYEVRWSFLMVPPVTIMMVNAVAVAVASARTLYSEFPQWSKLLGGA
FFSFWVLCHLYPFAKGLLGRRGRVPTIVFVWSGLISMTISLLWVYISPPAGARELIGGGGFSFP

>GRMZM2G122431_P01MEDGAEKATTPDSSSSQAAPPALSAVYVNRRLVAGNRAMAAVHVALVATVIGQRALALL
SAGTSSASPASRSLQVQHVAMALADLTLLFVWALSQSGLWRPVTRAAFPGRLLAAVPRGALPRVDVLVVTADPDKEPP
LGVVNTVVSAMALDYPGGKLSVYLSDDAGSPLTLLAARKAYAFAARAWVPFCRRHSVQCPWPDRYFAGDDDDADGD
RRREELAEERARVRKLYEKLKADIEAAKNDETISGSWSKDKRQNHDAYVEIIEDGDGVEEIPALVYVAREKRRAWPHHFK
AGALNALLRVSGVVSNAPYVLVLDCDMACNSRASALDAMCFLLDRRPPPDSLAFVQFPQLFHNLSHKDIYANELRYIFG
TRWFGLDGVRGPPLSGSGFYVRRDALYGATPTADFMPDATAVAELKTRFGHSDRLVASLRSPGVPPEAEAMMSLAAL
ASCAYEAGTAWGAGVGFMYQSVVEDYFTGFQRFFARGWTSAYCYPEPRPAFLGSVPTNLNDVLVQNKRWMSGMLA
VGVSRRHSPLACRPLLRASLLQAMAYAYFGFAALCAVPVLCYATLPQLCLLRGVPLFPCPAATAAAFASSLLQHMAEVC
VSRRGRLDLRTWWNEQRFWVLNALTAQLFGCVSAAQELLGARALDFDLTSKAAVDGSLYQDGVFDFTGCSALLLPAT
TLSVLNAAAIVAGTWKMSSSSSSSSGGFHFAPQLFLMCYGAALSYPLLEGMFLRRDPARVPPRITALSVALAAVLLAAM
LG

>GRMZM2G012044_P01MAGSSVRGGSNCPPLFVTEKPTRMARYAYRLFASTVLAGVLLVWLYRATHVPPMSSGAR
WWAWLGLSAAELWFGFYWVLTLSVRWSPVFRRAFPDQLLRRYKEEQLPGVDIFVCTADPTVEPPMLVISTVLSVMAY
DYPKEKLNIYLSDDAGSIITLYALYEASEFAKHWLPFCNKYQVEPRSPAAYFGTEASPPDACDRKEWFSLKEMHKDLAAR
VNSVVNSGKIPEVSKCKLMGFSRWSENASFRDHPSIVQILIDGNKRKATDVDGKVLPTLVYMAREKRPQEHHHFKAGSL

NALIRVSSVISNSPVIMNVDCDMYSNNSGSIRDALCFFQDEQLGQDIAFVQYPQNFENVVQNDIYGNPINTVNELDHP
CLDGWGGMCYYGTGCFHRREALCGRIYSPDYKEDWTRVARKTEDVIDLEGMAESLVTCTYEHNTLWGVEKGVIYGCP
LEDVITGLQIQCRGWRSVYHNPPRKGFLGMAPTSLGQILVQHKRWTEGFLQISLSKYSPFLLGHRKISLGLQMGYSVCG
FWAANSFPTLYYVTIPSLCFLNGISLFPEITSPWFVPFAYVAVAAYSCSLVESLQCGDTAVEWWNAQRMWLFRRITSYLL
AAIDTIRRMLGVTESGFTLTAKVTDPRALERYKKGMMEFGSFSVMFAIITTVALLNLACMMLGVAKVLLRKGAVSLGA
MFVQAVLCALIVAINFPVYEAMFVRKDSGRLPASVSVVSLCIVLPFCILPTKL

>GRMZM2G014558_P01MEERLFATEKHGGRALYRLHAVTVFLGICLLLCYRATHVPAAGSGGRAAWLGMLAAELW
FGFYWVITQSVRWCPIRRRTFHDRLAARFGERLPCVDIFVCTADPRSEPPSLVVATVLSVMAYNYPPAKLNVYLSDDGG
SILTFYALWEASAFAKHWLPFCRRYGVEPRSPAAYFAQSDEKPRHDPPHALQEWTSVKNLYDEMTERIDSAARTGNVP
EETRAKHKGFSEWDTGITSKDHHPIVQILIDGKDKAVADNEGNVLPTLVYVAREKRPQYHHNFKAGAMNALIRVSSVIS
NSPIILNVDCDMYSNNSDTIRDALCFFLDEETGHRIAFVQYPQNYNNLTKNNIYGNSLNVINQVELSGLDAWGGPLYIGT
GCFHRRETLCGRRFTEDYKEDWDRGTKEQQQHRHRVDGETEAKAKSLATCAYEHDDDTRWGDEVGLKYGCSVEDVIT
GLAIHCRGWESVYSNPARAAFVGVAPTTLAQTILQHKRWSEGNFGIFVSRYCPFVFGRRGKTRLPHQMGYSIYGLWAP
NSLPTLYYAVVPSLCLLKGTPLFPELTSPWIAPFVYVAVAKNVYSAWEALWCGDTLRGWWNGQRMWLVRRTTSYLYG
FVDTVRDSLGLSKMGFVVSSKVSDEDEAKRYEQEMMEFGTASPEYVIVAAVALLNLVCLAGMAAALDVFFVQVALCG
VLVLLNVPVYEAMFVRKDRGRMPFPITLASVGFVTLALIVPFF

>GRMZM2G028286_P01MAPWSGFWGGKPGGDAYRGTPVVVKMENPNWSISEISSPEDDDEDILAAAAAAGRRN
KGGRTKNAKQIRWVLLLKAHRAAGCLASLASAAVALGGAARRRVAAGRTDAEAGVVAAAGESPVVRSRFYAFIKAFLV
VSLLLLAVEVAAYFNGWDLAASALALPAIGLESLYASWLRFRATYVAPGIQFLTDACVVLFLIQSADRLIQCLGCFYIHIKRI
KPKPRSLALPDAEDPDAGYYPMVLVQIPMCNEKEVYQQSIAAVCNLDWPKSNFLVQVLDDSDDPLTQTLIREEVAKW
QQQGARIVYRHRVLRDGYKAGNLKSAMSCSYVKEYEFVAIFDADFQPHPDFLKRTVPHFKDNDELGLVQARWSFVNK
DENLLTRLQYINLCFHFEVEQQVNGVFLNFFGFNGTAGVWRIKALEDSGGWLERTTVEDMDIAVRAHLHGWKFIFLND
VECQCELPESYEAYRKQQHRWHSGPMQLFRLCLPDIIKCKIAFWKKANLIFLFFLLRKLILPFYSFTLFCIILPMTMFVPEAE
LPDWVVCYVPALMSLLNILPSPKSFPFIIPYLLFENTMSVTKFNAMISGLFQLGSAYEWVVTKKSGRSSEGDLIALAPPKEP
VKHATRTGSAPNLDAVAKEEQQQQQLAASRKDAAAKKKEKHNRIYKKELALSMLLLTAAARSLLSKQGIHFYFLLFQGV
SFLLVGLDLIGEQVE

>AC183932.3MAPCSGLWGGKAGGDAYRGTPVVVKMENPNWSISEISSPEDDDDDEDILAAAGGRRRKGARTKNAK
QIRWVLLLKAHRAAGCLASLASAAVALGAAARRRVAAGRTDADADADAGVLAVAGESPVVRSGFYAFIRAFLVVSLLLL
AVEVAAYINGWDLAASALALPALGLESLYASWLRFRAAYVAPGIQFLTDACVVLFLVQSADRLILCLGCFYIRVKRIKPEPK
SPALPDAEDPDAGYYPMVLVQIPMCNEKEVYQQSIAAVCNLDWPKSSFLVQVLDDSDDLLTQALIREEVAKWQQQGA
RIVYRHRVLRDGYKAGNLKSAMSCSYVKDYEFVAIFDADFQPQPDFLKRTVLHFKDNDELGLVQARWSFVNKDENLLT
RLQYINLCFHFEVEQQVNGVFLNFFGFNGTAGVWRIKALEDSGGWMERTTVEDMDIAVRAHLHGWKFIFLNDVECQ
CELPESYEAYRKQQHRWHSGPMQLFRLCLPDIIKCKMAFWKKGNLILLFFLLRKLILPLYSFTLFCIILPTAMFVPEAELPD
WVVCYVPALMSLLNVLPSPRSFPFVIPYLLFENTMSVTKFNAMVSGLFQLGSAYEWVVTKKSGSGPRSSEVAGDLVSLA
AAAPTAKKKKKKKHNRIYKKELALSMLLLTAAARSLLSKQGIHFYFLLFQGVSFLLVGLDLIGEQVE

>GRMZM2G074792_P01MRARLDYLAPPLQFLTNACVLLFLVQSVDRLVLCLGCFWIKLKGVRPVPPLPADKEDVEAG
PDGVPMVLVQMPMCNEREVYQQSIGAVCSLDWPRSNFLVQVLDDSDDATTSALIKEEVEKWQREGVRIVYRHRVIRD
GYKAGNLKSAMNCSYVKDYEFVVIFDADFQPQADFLKRTVPHFKGKDDVGLVQARWSFVNKDENLLTRLQNINLCFHF
EVEQQVNGAFLNFFGFNGTAGVWRIKALEESGGWMERTTVEDMDIAVRAHLKGWKFLFLNDVECQCELPESYEAYR

KQQHRWHSGPMQLFRLCFVDIIKSKIGFWKKFNLIFLFFLLRKLILPFYSFTLFCVILPMTMFVPEAELPAWVVCYIPATM
SILNILPSPKSFPFIVPYLLFENTMSVTKFNAMVSGLFQLGSAYEWVVTKKSGRSSEGDLVALVEKHSKQQRVGSAPNLD
ALTKESKGTEEEKNKKKRKKKHNRIYRKELALSFLLLTAAARSLLSAQGVHFYFLLFQGVSFLVVGLDLIGEQVD

>GRMZM2G454081_P04MVQWWGREASGGRGTSVVVKMESPEWTIPAESEVAAGGKAGRGKNARQITWVLLLK
AHRTAGKLTGAASAVLSIAGAAWRRVAAGRTDEDEDEDAGAAPPGESPALRSRFYGFLRASLVLSVLLLAADVAAHLQ
GWHLAVDVPDLLAVEGLFAAGYASWARVRLEYLAPALQFLANACVVLFLVQSADRLILCLGCLWIKHRGIRPVPKAGGK
GSDDVEAGTGNFPMVLVQIPMRNEKEVYQQSIGAVCGLDWPRPNFLVQVLDDSDDAATSTLIKEEVEKWQREGVRIL
YRHRLIRDGYKAGNLKSAMNCSYVKDYEFVVIFDADFQPQPDFLKRTVPHFKGNEDVGLVQARWSFVNKDENLLTRLQ
NINLCFHFEVEQQVNGIFLNFFGFNGTAGVWRIKALEDSGGWMERTTVEDMDIAVRAHLKGWKFLFLNDVECQCELP
ESYEAYRKQQHRWHSGPMQSDSGRKFNLIFLFFLLRKLILPFYSFTLFCIILPMTMFIPEAELPAWVVCYIPATMSLLNILP
APKSFPFIVPYLLFENTMSVTKFNAMVSGLFQLGSAHEWVVTKKSGRSSEGDLVVEKQPRQQRAGSASNLGSLAKEPSS
SLRKDSQRKKHNRIYRKELALSFLLLTAAARSLISVQGIHFYFLLFQGDCAKGNPEKGKKMFCMSASVECLRALFVNARA
QITCGSRTVIFQPTCPAVMVERDRVSGELGKGFRNNPSPFTNGASNSERRLCLENSYSRSCSQGGDRCVNRCRYGCCFR
SANIFIRRLGLHLVSLPSLPVFQSPCLLSLAGSVRMQSDLRTV

>GRMZM2G027794_P01MPQWWGREARGGGGGGGTSVVVKMESPDWAVREPEAARGKAGRGKNARQITWVLL
LKAHRAAGKLTGAASAALSVAAAARRRVAAGRTDADADAGAGAGAAPHGESPALRTRLYGFLRASLVLSMLLLAADV
AAHLQGWHLLAVDVPDLLAVEGLFAAGYASWVRVRLQYLAPALQFLANACVLLFLVQSADRLILCLGCLWIKLRGIKPV
PNAAGKGPDDVEAGTGEFPMVLVQIPMCNEKEVYQQSIGAVCGLDWPRPNFLVQVLDDSDDAATSALIKEEVERWQ
REGVRILYRHRVIRDGYKAGNLKSAMNCSYVKDYEFVVIFDADFQPQPDFLKRTVPHFKGNEDVGLVQARWSFVNKDE
NLLTRLQNINLCFHFEVEQQVNGIFLNFFGFNGTAGVWRIKALEDSGGWMERTTVEDMDIAVRAHLKGWKFLFLNDV
ECQCELPESYEAYRKQQHRWHSGPMQLFRLCFVDIIKSKIGFWKKFNLIFLFFLLRKLILPFYSFTLFCIILPMTMFIPEAELP
AWVVCYIPATMSLLNILPAPKSFPFIVPYLLFENTMSVTKFNAMVSGLFQLGSAYEWVVTKKSGRSSEGDLIALVEKQPK
QQRVGSAPNLESLTKESSSLKRDSKRKKHNRIYRKELALSFLLLTAAARSLLSVQGIHFYFLLFQGVSFLVVGLDLIGEQVE

>GRMZM2G142685_P01MAPPPSSWWGSEEQRGTPVVVKMDNPYSLVEIDGPGMPPSDKARGKNAKQFTWVLLL
RAHRAVGCVAWLAGGFWGVLGAVNRRVRRSRDADDEPDAEASGRGRVMLRFLRAFLLLSLAMLAFETVAHLKGWQ
FPQHLMPGNLQELEEQLQHLPEHLRHLPENLRQLPDHLRVPERQEIQGWLHRAYVAWLEFRVDYIAWAIQKLSTFCILL
FMVQSVDRIVQCLACFWIKIRGIKPRIPASAGGKPRGGTTGRKRVDDVENGDADDDRYFPMVLVQMPMCNEKEVYE
TSISHVCQMDWPRDRLLIQVLDDSDDEVCQMLIKAEVTKWSQRGVNVIYRHRLSRTGYKAGNLKSAMACDYVKDYEF
VAIFDADFQPNPDFLKLTVPHFKENPELGLVQARWSFVNKDENLLTRLQNINLCFHFEVEQQVNGVYLNFFGFNGTAG
VWRIKALEDSGGWMERTTVEDMDIAVRAHLNGWKFIFLNDVKVLCELPESYEAYRKQQHRWHSGPMQLFRLCIPTVF
RSKIPFWKKANLVMLFFLLRKLVLPFYSFTLFCVILPLTMFVPEAELPIWVICYIPVLMSILNILPAPKSFPFIIPYLLFENTMS
VTKFNAMVSGLFQLGSSYEWIVTKKAGRTSSASDILALAEADAHAHAPAPAAKLVRGVSEGGLQEWGRLREQEAAEW
ANKEDAAAALAAAAAPATPKKSSNKAKKPPNRIFKKELALACLLLTAATRSLLSKQGLHFYFLLFQGVTFLAVGLDLIGEQ
VS

>GRMZM2G173759_P01MAPGLRLAYPWGRDVRRGTPVVVTMENPNYSVVEIDGPEAEALRAGVPPMDKGRGRG
RSAKQFTWVLLLRAHRAAGCLASLAAVTWALPSAVAKRFRRAAAAEGVGRGRGWLLYRFIKVLLALSLLALTVELAAYW
KGWHFQRPNLTMPEFHVPEVEDIQGWLHTAYLAWMSFRADYIRRPIEFLSKVCILLFVVQSLDRLVLCIACFWIKLKKIK
PRLEGDPFREGSGYLHPMVLVQIPMCNEKEVYEQSISAVCQLDWPRDKFLIQVLDDSSDESIQMLIKAEVSKWNQQGV
NIVYRHRVLRTGYKAGNLNSAMSCDYVKNFEFVAIFDADFQPSTDFLKKTIPHFDGNPELGLVQARWSFVNKDENLLTR

LQNINLCFHFEVEQQVNGVFLNFFGFNGTAGVWRIQALEESGGWLERTTVEDMDIAVRAHLNGWKFIFLNDVKVLCE
VPESYEAYRKQQHRWHSGPMHLFRLCFPDIITAKISSWKKANLILLFFLLRKLILPFYSFTLFCVILPLTMFVPEAELPVWVI
CYVPICMSFLNILPSPRSFPFIVPYLLFENTMSVTKFNAMVSGLFKLGSSYEWVVTKKSGRSSELDLLTSEEKNRKCTTLPQ
LQNQLPENSELVEINVRKEQREKVPNDAKRGNKIYKKELALSLLLLTAATRSLLSAQGIHFYFLLFQGMSFLAVGLDLIGEQ
IS

>GRMZM2G135286_P01MAPPSSWWGGEDQDGTPVVVKMDNPYSLVEIDGLGVPPADKARGKNAKQFTWVLLL
RAHRAVGCVAWLAGGFWGVLGAVNRRVRRSRDADDEPDAEASGRGRAMLRFLRAFLLLSLAMLAFETVAHLKGWQ
FPQHLPGNLQELEEQLQHLPEHLRHLPENLRQLPDHLRMPERQEIQGWLHRAYVAWLEFRVDYIAWAIQKLSSFCILLF
MVQSVDRIVQCLACFWIKIRGIKPRVPASGGKPRGTTGRKSADAENGFADGDADGYFPMVLVQMPMCNEKEVYETSI
SHVCQIDWPRDRLLIQVLDDSDDEVCRMLIKAEVTKWSQRGVNVIYRHRLSRTGYKAGNLKSAMACDYVKDYEFVAIF
DADFQPNPDFLKLTVPHFKENPELGLVQARWSFVNKDENLLTRLQNINLCFHFEVEQQVNGVYLNFFGFNGTAGVWR
IKALEDSGGWMERTTVEDMDIAVRAHLNGWKFIFLNDVKVLCELPESYQAYRKQQHRWHSGPMQLFRLCIPAVFRSKI
PFWKKANLVMLFFLLRKLVLPFYSFTLFCVILPLTMFVPEAELPIWVICYIPVLMSLLNILPAPKSFPFVIPYLLFENTMSVTK
FNAMVSGLFQLGSSYEWIVTKKAGRTSSASDILALAEADSHAPLPPPAKLVRRVSEGGLQEWSRLREQETAEWANKEE
AAAALAAAAPATPKMTRKTKKPNRIFKKELALACLLLTAATRSLLSKQGLHFYFLLFQGVTFLAVGLDLIGEQVS

>GRMZM2G405567_P02MGAAAAGGHALRAVGDVVSFPATVAAFVEALLQGWAEARAGLLVPLLRAAVLLCTAMS
LIVLAEKVFLGAVSSVAKLRRRRPGRVCRCDPDEEAAAASQAYPMVLVQIPMYNEREVYQLSIEAACRLTWPVDRLIVQ
VLDDSTDSVIKELVKGECERWATEEGINVKYETRKDRAGYKAGNLKEGMRHAYVRACEFVAMFDADFQPPPDFLVRTV
PFLVHNPSLALVQTRWKFVNANDCLLTRMQEMSMDYHFKVEQEAGSSLCNFFGYNGTAGVWRTQAIVESGGWEDR
TTAEDMDLALRAGLLGWEFVYVGSIKVKSELPSTLKAYRSQQHRWSCGPALLFKKMFWQILAAERVSVWKKWYMVY
DFFIARRIVGTFYTFFFFSVLIPLNILLPEAQIPVWELIYIPIAITLLNSVGTPRSIHLVILWVLFENVMALHRFKAILIGFLEADR
ANEWIVTQKLGNLQKLKSIARLTGSYRFKDRFHFLEVFIGLFLLASACFDYLYRDDYVYLFVLPQSIMYFAIGFQFVGLNVS
ED

>GRMZM2G020742_P01MTLDYHFKVEQEAGSSTFGFFGFNGTAGVWRTSSIKEAGGWEDRTTVEDMDLAVRAGL
KGWKFIYVGDVKVKSELPSNLKAYRRQQHRWTCGAANLFRKMGAEIILTKEVSLWRKLYLIYSFFFIRKVVAHVVPFMLY
CVIIPLSVLIPEVTVPVWGVVYIPTTITLLYAIRNPSSIHFIPFWILFENVMSFHRTKATFIGLLELGNVNEWVVTEKLGRTKP
VPQMLEKPRCRFWDRCTISEILVAIFLFFCATYNLVLGDDFYFVYIYLQEIAFLIVGTGFCGT

>GRMZM2G108600_P03MGGVRGHSSAAGVSEEAAAAAAWVWAAADLPVRVDWAGAAAQCASLWAHARALLV
VPAVRLLVALSLAMTVMVLAEKLFVCAVCVAVRAFRLGPHRRYRWEPIAAAAAAVGYPVVLVQIPMYNEREVYKLSIG
AACALEWPPERFVIQVLDDSTDPVVKDLVETECQRWKSKGVNIKYEVRGNRKGYKAGALKEGLKHDYVADCEYIAMFD
ADFQPDSDFLLRTIPFLVHNPEIALVQARWKFVNSDECLLTRFQEMSLDYHFKYEQEAGSSVYSFFGFNGTAGVWRISAI
DDAGGWKDRTTVEDMDLAVRAMLQGWKFLYVGDIKVKSELPSTFKAYRFQQHRWSCGPANLFKKMMVEILENKRV
SLWSKIHLWYDFFFVGKVAAHTVTFIYYCFAIPVSVLFPEIQIPLWGVVYVPTVITLLKALGTPSSFHLVILWVLFENVMSL
HRIKAAVSGLLDAGGRVNEWVVTEKLGDTSKAKPGTNGSDTAVKVIDVKLTEPLVPKLVKRRARFWERYHCSELFVGTC
IILCGFYDLLFANKGYYIFLFLQGTAFLVVGFGYVGTLPPCTA

>GRMZM2G099088_P01MAADLVETECQRWKSKGVNIKYEVRGNRKGYKAGALKEGLKHDYVEDCEYIAMFDADF
QPEPDFLLRAVPFLVHNPEIALVQARWKFVNSGECLLTRFQEMSLDYHFKYEQEAGSSLHSFFGFNGTAGVWRIAAIDD
AGGWKDRTTVEDMDLAVRAMLQGWKFVYVGDIKVKSELPSTFKAYRFQQHRWSCGPANLFKKMMVEILENKKVSL

WSKIYLWYNFFFVGKVAAHTVTFIYYCCAIPVSVLLPEIQIPLWGVVYIPTLITLLKALGTPSSFHLVILWVLFENVMSLHRI
KAAASGLLDAGGRVNEWVVTEKLGDTSKAKPGANGSDDVKVIDVKLTEPLVPKLVKRRARFWERYNCSELFVGTCILLC
GFYDLLFAKKGYYIFLFLQGTAFLVVGFGYVGTLPHCNA

>GRMZM2G010142_P02MEAGEIGGALVFILAAAAAVAAAVSVGAVDFSRPLTAGAPFDFQAAVSWLIGILDGTSSA
AADVDGAWVAVRAGVIAPVLQVAVWACMVMSVMLVVEAVYNSVISLGVKAIGWRPEWRFKWKPLDSADEEKGTA
HFPMVLVQIPMYNELEVYKLSIAAACELQWPKDRIVIQVLDDSTDPFIKNLVELECEHWVNKGVNIKYATRTSRKGFKA
GALKKGMECDYAWQSEYIAIFDADFQPEPDFLLQTVPFLLHNPEVALVQARWSFVNDTTSLLTRVQKMFYDYHFKVEQ
EAGSATFAFFSFNGTAGVWRTGAIRDAGGWKDRTTVEDMDLAVRATLKGWKFVYVGDVRVKSELPSTYKAYCRQQF
RWSSGGANLFRKMAKDVLFAKDISLVKKFYMLYSFFFVRRVVAPTAACILYNVIIPISVTIPELYLPVWGVAYIPMVLTVV
TAIRHPKNLHILPFWILFESVMTLHRMRAAMTGLLELEGFNQWIVTKKVGNDLEDTEVPLLQKTRKRLRDRVNLPEIGFS
VFLFLCASYNLVFHGKTSYYLYMYLQGLAFLLLGFNFTGNCSCYQ

>GRMZM2G115772_P01MAAWAVPALAAAAWALRAAVWACLAASAMLVAEAAYMGLASLASAAAMLWRRPDA
RYRWEPMPGGCDVEAATGADFPMVLVQIPMYNEREVYKLSIDAACALTWPPDRIVIQVLDDSTDPIIKELVELECQDW
ATKKINIKYEVRNNRKGYKAGALKKGMEHIYAKQCEFVAIFDADFQPEPDFLLKTIPFLVHNPKIALVQARWEFVNYDVC
LMTRIQKMSLDYHFKVEQESGSFVYSFFGFNGTAGVWRVSAINQSGGWKDRTTVEDMDLAVRASLKGWEFLYVGDI
RVKSELPSTFKAYRHQQHRWTCGAANLFRKMAWEIITNKEVSIWKKHHLLYSFFFVRRVIAPLVTFLFYCVVIPLSAMVP
GVSIPVWGLVYIPTAITCMNAIRNPWSLHLMPFWILFENVMSMHRMRAAVTGLLETARANDWVVTEKVGDLVKDDL
DVPLLEPVKPTECVERIYFPELLLALHLLICASYDFVLGSHKYYLYIYLQAFAYVVMGFGFVGTKTPCS

>GRMZM2G178880_P03MELEGPWARVLHLHDACEWGRAVRARAVAPALEAAAWACLAMSVMLVLEVCYMSVS
SFVAVNLLRRTPQRRYSWEPMPSGTARGDDEEAAVGDGGGEAYPMVLVQIPMYNEREVYKISIGAACALTWPPDRIII
QVLDDSTDPFIKELVEFECKDWASKKINIKYEIRESRKGYKAGALKKGMEHSYAQECDFVAIFDADFQPDPDFLLRTIPFL
VHNPKIALVQTRWEFVNYNICLLTRIQKMSLDYHFKVEQESGSSVHAFFGFNGTAGVWRVSAIGEAGGWKDRTTVED
MDLAVRASLKGWQFLYVGDIRFFVQVKSELPSTFKAYRHQQHRWTCGAANLFRKMAGDIVISKGATVWKKLHLLYSFF
FVRRVIAPILTFLFYCVVIPLSVMVPEVSIPAWGMFYIPTAITIMTAIRNPW

>GRMZM2G424486_P01MSCVRNEASGQTGSVYRLSIGAACGMSWPSERLVIQVLDDSTNPAIRELVEVECARWAR
KGVRICYENRSNRNGYKAGAMREGLKKHYARDCEFVAIFDADFQPDSDFLRRTVPLLQRDPGVGLVQARWRYVNADE
CILTRIQEMSLNYHFAVEQEVGSACHAFFGFNGTAGVWRVAALADAGGWKERTTVEDMDLAVRASLRGWRFVYVG
DLVVRNELPSTFKAYRYQQHRWSCGPANLFRKVLPEILRSDRVSLGKKFHLLYAFFFVRKVVAHLVTFLFYCVVIPACVLV
QGDVRLPKYVAMYVPALITLLNAACTPRSCHLLIFWILFENVMSMHRSKAAIIGLLEASRANEWVVTDKLGSSKAAAAV
VAKKKKQQLVRSRCCSTRREMHVLELAMGVCLLYCAVYDIVFFGRDHYYMYLLLQSAAAFIVGFGYVGTTTPS

>GRMZM2G105631_P01MDALPEAWSQVRAPVIVPLLRLAVAVCLTMSVLLFLERVYMAVVISGVRLLRLRPDRRYR
CDPLPEDDPELGSSAFPVVLVQIPMFNEREVYQLSIGAVCGLSWPADRLVVQVLDDSTDEVIKEMVRMECERWARKGI
NITYQIREDRKGYKAGALRAGMRHAYVRDCEYVAIFDADFQPDPDYLKRTIPYLVHNPEIALVQARWRFVNADECLMT
RMQEMSLDYHFTVEQEVSSSVCAFFGFNGTAGVWRISAVNEAGGWKDRTTVEDMDLAIRASLKGWKFVYLGDVQV
KSELPSTFKAFRFQQHRWSCGPANLFRKMLMEIVTNKKVTIWKKIHVIYNFFLIRKIIAHIITFSFYCVIIPATIFVPEVRIPK
WGCVYIPSAITLLNSVGTPRSFHLLFFWVAFENVMSLHRTKATLIGLLEAGRANEWVVTAKLGSAMKMKSANKAGLRK
QFMRIWERLHVTELGVAAFLFSCGWYDLAYGRDHFFIYLFFQSVAFFIVGVGYVGTIVPQS
