# Supplementary material for: Genome-wide analysis of the cellulose synthase-like (Csl) gene family in bread wheat (Triticum aestivum L.)
Source: BMC Plant Biol. 2017 Nov 3;17:193. doi: 10.1186/s12870-017-1142-z (PMC5670714; doi:10.1186/s12870-017-1142-z)
Supplement: Supplementary file 2 — List of Csl subfamily genes, their protein sizes (number of amino acids), and multiple protein sequence alignments for different subfamilies. The conserved motifs (D, D, DXD, QXXRW) diagnostic of CSL proteins are highlighted with red boxes for each of the subfamilies. (PDF 465 kb) [file 12870_2017_1142_MOESM2_ESM.pdf]

Fig. S_2: List of *Csl* subfamily genes, their protein sizes (number of amino acids), and multiple protein sequence alignments. The conserved motifs (D, D, DXD, QXXRW) diagnostic of CSL proteins are highlighted with red boxes for each of the subfamilies.

Figure S_2A: *CslA* subfamily

**S.No**	**Gene name with number of splice variants (CslA)**	**No. of amino acids (aa)**
1	TRIAE_CS42_2BS_TGACv1_146583_AA0468630.1	581 aa
2	TRIAE_CS42_2AS_TGACv1_113418_AA0355820.2	580 aa
3	TRIAE_CS42_2DS_TGACv1_177473_AA0578070.1	581 aa
4	TRIAE_CS42_2AS_TGACv1_113300_AA0354190.1	579 aa
5	TRIAE_CS42_2DS_TGACv1_177798_AA0584795.1	881 aa
6	TRIAE_CS42_6BS_TGACv1_513375_AA1639370.1_2_SPLICE	518 aa
7	TRIAE_CS42_6AS_TGACv1_485966_AA1554960.1	518 aa
8	TRIAE_CS42_U_TGACv1_642146_AA2112270.1	522 aa
9	TRIAE_CS42_7BL_TGACv1_579090_AA1903960.1	375 aa
10	TRIAE_CS42_7AL_TGACv1_558725_AA1795700.1	518 aa
11	TRIAE_CS42_6DS_TGACv1_543811_AA1744360.1	531 aa
12	TRIAE_CS42_6AS_TGACv1_487286_AA1569690.1	528 aa
13	TRIAE_CS42_6BS_TGACv1_513376_AA1639390.2_2_SPLICE	528 aa
14	TRIAE_CS42_U_TGACv1_642146_AA2112290.1	512 aa
15	TRIAE_CS42_7BS_TGACv1_592860_AA1945380.1	547 aa
16	TRIAE_CS42_7DS_TGACv1_623146_AA2050070.1	545 aa
17	TRIAE_CS42_7AS_TGACv1_569190_AA1809650.1_3_SPLICE	551 aa
18	TRIAE_CS42_7DL_TGACv1_602617_AA1962870.1_2_SPLICE	555 aa
19	TRIAE_CS42_7AL_TGACv1_557254_AA1778850.1	515 aa
20	TRIAE_CS42_7BL_TGACv1_578444_AA1895100.1	515 aa
21	TRIAE_CS42_3DL_TGACv1_249033_AA0835410.1_2_SPLICE	572 aa
22	TRIAE_CS42_3B_TGACv1_221079_AA0729630.1_2_SPLICE	571 aa
23	TRIAE_CS42_3AL_TGACv1_197519_AA0666560.1	573 aa
24	TRIAE_CS42_3B_TGACv1_220828_AA0720500.1	570 aa
25	TRIAE_CS42_3DS_TGACv1_273022_AA0927600.1	568 aa
26	TRIAE_CS42_2AL_TGACv1_093375_AA0278800.1	527 aa
27	TRIAE_CS42_2BL_TGACv1_129747_AA0394630.1	528 aa
28	TRIAE_CS42_2DL_TGACv1_160461_AA0550770.1	548 aa
29	TRIAE_CS42_1AS_TGACv1_019142_AA0061550.1	515 aa
30	TRIAE_CS42_3AS_TGACv1_210508_AA0674280.1	566 aa
31	TRIAE_CS42_3DS_TGACv1_272005_AA0912960.1	570 aa
32	TRIAE_CS42_3B_TGACv1_223332_AA0780350.1	925 aa

```
TRIAE_CS42_3AS_TGACv --------------------------------------------------------------------------------- 0
TRIAE_CS42_3B_TGACv1 MLLLKIDIAKAFDTVSWEYILELLQRMNFPAHWRDRIALLLSSVSSAYLLKGDPGPAILHQRGLRQGDPLSAILFILVIV 80
TRIAE_CS42_3B_TGACv1 --------------------------------------------------------------------------------- 0
TRIAE_CS42_3DS_TGACv --------------------------------------------------------------------------------- 0
TRIAE_CS42_3DS_TGACv --------------------------------------------------------------------------------- 0
TRIAE_CS42_3DL_TGACv --------------------------------------------------------------------------------- 0
TRIAE_CS42_3B_TGACv1 --------------------------------------------------------------------------------- 0
TRIAE_CS42_3AL_TGACv --------------------------------------------------------------------------------- 0
TRIAE_CS42_6BS_TGACv --------------------------------------------------------------------------------- 0
TRIAE_CS42_6AS_TGACv --------------------------------------------------------------------------------- 0
TRIAE_CS42_7AL_TGACv --------------------------------------------------------------------------------- 0
TRIAE_CS42_U_TGACv1_ --------------------------------------------------------------------------------- 0
TRIAE_CS42_U_TGACv1_ --------------------------------------------------------------------------------- 0
TRIAE_CS42_7BL_TGACv --------------------------------------------------------------------------------- 0
TRIAE_CS42_7AL_TGACv --------------------------------------------------------------------------------- 0
TRIAE_CS42_7BL_TGACv --------------------------------------------------------------------------------- 0
TRIAE_CS42_7DL_TGACv --------------------------------------------------------------------------------- 0
TRIAE_CS42_2AL_TGACv --------------------------------------------------------------------------------- 0
TRIAE_CS42_2DL_TGACv --------------------------------------------------------------------------------- 0
TRIAE_CS42_2BL_TGACv --------------------------------------------------------------------------------- 0
TRIAE_CS42_1AS_TGACv --------------------------------------------------------------------------------- 0
TRIAE_CS42_7DS_TGACv --------------------------------------------------------------------------------- 0
TRIAE_CS42_7AS_TGACv --------------------------------------------------------------------------------- 0
TRIAE_CS42_7BS_TGACv --------------------------------------------------------------------------------- 0
TRIAE_CS42_6DS_TGACv --------------------------------------------------------------------------------- 0
TRIAE_CS42_6BS_TGACv --------------------------------------------------------------------------------- 0
TRIAE_CS42_6AS_TGACv --------------------------------------------------------------------------------- 0
TRIAE_CS42_2BS_TGACv --------------------------------------------------------------------------------- 0
TRIAE_CS42_2DS_TGACv --------------------------------------------------------------------------------- 0
TRIAE_CS42_2AS_TGACv --------------------------------------------------------------------------------- 0
```

```
TRIAE_CS42_2AS_TGACv -------------------------------------------------------------------------------- 0
TRIAE_CS42_2DS_TGACv -------------------------------------------------------------------------------- 0

TRIAE_CS42_3AS_TGACv -------------------------------------------------------------------------------- 0
TRIAE_CS42_3B_TGACv1 PLHRMLEAAQQAGTIAPLPAGAARLRVTLYADDAIFFANPVRQEIDTIMQLLQGFGEAAGLRGNPQKSSAATLNYGSIDL 160
TRIAE_CS42_3B_TGACv1 -------------------------------------------------------------------------------- 0
TRIAE_CS42_3DS_TGACv -------------------------------------------------------------------------------- 0
TRIAE_CS42_3DS_TGACv -------------------------------------------------------------------------------- 0
TRIAE_CS42_3DL_TGACv -------------------------------------------------------------------------------- 0
TRIAE_CS42_3B_TGACv1 -------------------------------------------------------------------------------- 0
TRIAE_CS42_3AL_TGACv -------------------------------------------------------------------------------- 0
TRIAE_CS42_6BS_TGACv -------------------------------------------------------------------------------- 0
TRIAE_CS42_6AS_TGACv -------------------------------------------------------------------------------- 0
TRIAE_CS42_7AL_TGACv -------------------------------------------------------------------------------- 0
TRIAE_CS42_U_TGACv1_ -------------------------------------------------------------------------------- 0
TRIAE_CS42_U_TGACv1_ -------------------------------------------------------------------------------- 0
TRIAE_CS42_7BL_TGACv -------------------------------------------------------------------------------- 0
TRIAE_CS42_7AL_TGACv -------------------------------------------------------------------------------- 0
TRIAE_CS42_7BL_TGACv -------------------------------------------------------------------------------- 0
TRIAE_CS42_7DL_TGACv -------------------------------------------------------------------------------- 0
TRIAE_CS42_2AL_TGACv -------------------------------------------------------------------------------- 0
TRIAE_CS42_2DL_TGACv -------------------------------------------------------------------------------- 0
TRIAE_CS42_2BL_TGACv -------------------------------------------------------------------------------- 0
TRIAE_CS42_1AS_TGACv -------------------------------------------------------------------------------- 0
TRIAE_CS42_7DS_TGACv -------------------------------------------------------------------------------- 0
TRIAE_CS42_7AS_TGACv -------------------------------------------------------------------------------- 0
TRIAE_CS42_7BS_TGACv -------------------------------------------------------------------------------- 0
TRIAE_CS42_6DS_TGACv -------------------------------------------------------------------------------- 0
TRIAE_CS42_6BS_TGACv -------------------------------------------------------------------------------- 0
TRIAE_CS42_6AS_TGACv -------------------------------------------------------------------------------- 0
TRIAE_CS42_2BS_TGACv -------------------------------------------------------------------------------- 0
TRIAE_CS42_2DS_TGACv -------------------------------------------------------------------------------- 0
TRIAE_CS42_2AS_TGACv -------------------------------------------------------------------------------- 0
TRIAE_CS42_2AS_TGACv -------------------------------------------------------------------------------- 0
TRIAE_CS42_2DS_TGACv -------------------------------------------------------------------------------- 0

TRIAE_CS42_3AS_TGACv -------------------------------------------------------------------------------- 0
TRIAE_CS42_3B_TGACv1 IDVLKNFSGTRVGFPIRYLGLPLCIGRLPLCTRVGFPIRYLGWLLGKANSCIAPPLAVASHVLVRCVLSALPAFAMAVLR 240
TRIAE_CS42_3B_TGACv1 -------------------------------------------------------------------------------- 0
TRIAE_CS42_3DS_TGACv -------------------------------------------------------------------------------- 0
TRIAE_CS42_3DS_TGACv -------------------------------------------------------------------------------- 0
TRIAE_CS42_3DL_TGACv -------------------------------------------------------------------------------- 0
TRIAE_CS42_3B_TGACv1 -------------------------------------------------------------------------------- 0
TRIAE_CS42_3AL_TGACv -------------------------------------------------------------------------------- 0
TRIAE_CS42_6BS_TGACv -------------------------------------------------------------------------------- 0
TRIAE_CS42_6AS_TGACv -------------------------------------------------------------------------------- 0
TRIAE_CS42_7AL_TGACv -------------------------------------------------------------------------------- 0
TRIAE_CS42_U_TGACv1_ -------------------------------------------------------------------------------- 0
TRIAE_CS42_U_TGACv1_ -------------------------------------------------------------------------------- 0
TRIAE_CS42_7BL_TGACv -------------------------------------------------------------------------------- 0
TRIAE_CS42_7AL_TGACv -------------------------------------------------------------------------------- 0
TRIAE_CS42_7BL_TGACv -------------------------------------------------------------------------------- 0
TRIAE_CS42_7DL_TGACv -------------------------------------------------------------------------------- 0
TRIAE_CS42_2AL_TGACv -------------------------------------------------------------------------------- 0
TRIAE_CS42_2DL_TGACv -------------------------------------------------------------------------------- 0
TRIAE_CS42_2BL_TGACv -------------------------------------------------------------------------------- 0
TRIAE_CS42_1AS_TGACv -------------------------------------------------------------------------------- 0
TRIAE_CS42_7DS_TGACv -------------------------------------------------------------------------------- 0
TRIAE_CS42_7AS_TGACv -------------------------------------------------------------------------------- 0
TRIAE_CS42_7BS_TGACv -------------------------------------------------------------------------------- 0
TRIAE_CS42_6DS_TGACv -------------------------------------------------------------------------------- 0
TRIAE_CS42_6BS_TGACv -------------------------------------------------------------------------------- 0
TRIAE_CS42_6AS_TGACv -------------------------------------------------------------------------------- 0
TRIAE_CS42_2BS_TGACv -------------------------------------------------------------------------------- 0
TRIAE_CS42_2DS_TGACv -------------------------------------------------------------------------------- 0
TRIAE_CS42_2AS_TGACv -------------------------------------------------------------------------------- 0
TRIAE_CS42_2AS_TGACv -------------------------------------------------------------------------------- 0
TRIAE_CS42_2DS_TGACv --------------------------------MAAWNPETHGSGAIIVGADDCETTVEDEMAAGRDANTKLFHRVANGRK 48

TRIAE_CS42_3AS_TGACv -------------------------------------------------------------------------------- 0
TRIAE_CS42_3B_TGACv1 IPKRFYKDVDKARWRFLWVHDHEVTGGRCKVNWRLVTSPVDHGGLGIPSMERFARALCLRWLWLAWTDPARPWARMGTPC 320
TRIAE_CS42_3B_TGACv1 -------------------------------------------------------------------------------- 0
TRIAE_CS42_3DS_TGACv -------------------------------------------------------------------------------- 0
TRIAE_CS42_3DS_TGACv -------------------------------------------------------------------------------- 0
TRIAE_CS42_3DL_TGACv -------------------------------------------------------------------------------- 0
TRIAE_CS42_3B_TGACv1 -------------------------------------------------------------------------------- 0
TRIAE_CS42_3AL_TGACv -------------------------------------------------------------------------------- 0
TRIAE_CS42_6BS_TGACv -------------------------------------------------------------------------------- 0
TRIAE_CS42_6AS_TGACv -------------------------------------------------------------------------------- 0
TRIAE_CS42_7AL_TGACv -------------------------------------------------------------------------------- 0
TRIAE_CS42_U_TGACv1_ -------------------------------------------------------------------------------- 0
TRIAE_CS42_U_TGACv1_ -------------------------------------------------------------------------------- 0
TRIAE_CS42_7BL_TGACv -------------------------------------------------------------------------------- 0
TRIAE_CS42_7AL_TGACv -------------------------------------------------------------------------------- 0
TRIAE_CS42_7BL_TGACv -------------------------------------------------------------------------------- 0
TRIAE_CS42_7DL_TGACv -------------------------------------------------------------------------------- 0
TRIAE_CS42_2AL_TGACv -------------------------------------------------------------------------------- 0
TRIAE_CS42_2DL_TGACv -------------------------------------------------------------------------------- 0
TRIAE_CS42_2BL_TGACv -------------------------------------------------------------------------------- 0
TRIAE_CS42_1AS_TGACv -------------------------------------------------------------------------------- 0
TRIAE_CS42_7DS_TGACv -------------------------------------------------------------------------------- 0
TRIAE_CS42_7AS_TGACv -------------------------------------------------------------------------------- 0
TRIAE_CS42_7BS_TGACv -------------------------------------------------------------------------------- 0
TRIAE_CS42_6DS_TGACv -------------------------------------------------------------------------------- 0
TRIAE_CS42_6BS_TGACv -------------------------------------------------------------------------------- 0
```

```
TRIAE_CS42_6AS_TGACv -------------------------------------------------------------------------------- 0
TRIAE_CS42_2BS_TGACv -----------------------------------------------------------------------------MEA 3
TRIAE_CS42_2DS_TGACv -----------------------------------------------------------------------------MEA 3
TRIAE_CS42_2AS_TGACv -----------------------------------------------------------------------------MEA 3
TRIAE_CS42_2AS_TGACv -----------------------------------------------------------------------------MEA 3
TRIAE_CS42_2DS_TGACv LKNFIPAISVEGITITDQAAKEEAFFEAYSELLGRCGSREHTLDLDYLGIESINLEDQDLVFQEEEVWKVVRDMPSDRAL 128

TRIAE_CS42_3AS_TGACv ---------------------------------------------------------------------------MAMAA 5
TRIAE_CS42_3B_TGACv1 DDKDRALFASATTVTVGDGNRVLFWHCSWLGEQPVRQDYPNLFRRSTRKNRMMADAIRDDRWIMDLRRSGAGEEVMAMAA 400
TRIAE_CS42_3B_TGACv1 ---------------------------------------------------------------------------MAMAA 5
TRIAE_CS42_3DS_TGACv -----------------------------------------------------------------------------MAA 3
TRIAE_CS42_3DS_TGACv ---------------------------------------------------------------------------MAATA 5
TRIAE_CS42_3DL_TGACv ---------------------------------------------------------------------MAGAGEEFMA- 10
TRIAE_CS42_3B_TGACv1 ---------------------------------------------------------------------MAGAGEEFMA- 10
TRIAE_CS42_3AL_TGACv ---------------------------------------------------------------------MAGAGEEFMAS 11
TRIAE_CS42_6BS_TGACv -------------------------------------------------------------------------------- 0
TRIAE_CS42_6AS_TGACv -------------------------------------------------------------------------------- 0
TRIAE_CS42_7AL_TGACv -------------------------------------------------------------------------------- 0
TRIAE_CS42_U_TGACv1_ -------------------------------------------------------------------------------- 0
TRIAE_CS42_U_TGACv1_ -------------------------------------------------------------------------------- 0
TRIAE_CS42_7BL_TGACv -------------------------------------------------------------------------------- 0
TRIAE_CS42_7AL_TGACv -------------------------------------------------------------------------------- 0
TRIAE_CS42_7BL_TGACv -------------------------------------------------------------------------------- 0
TRIAE_CS42_7DL_TGACv -------------------------------------------------------------------------------- 0
TRIAE_CS42_2AL_TGACv -------------------------------------------------------------------------------- 0
TRIAE_CS42_2DL_TGACv ---------------------------------------------------------------------MEKKKRRSSIS 11
TRIAE_CS42_2BL_TGACv -------------------------------------------------------------------------------- 0
TRIAE_CS42_1AS_TGACv -------------------------------------------------------------------------------- 0
TRIAE_CS42_7DS_TGACv --------------------------------------------------------------MAGDGEGAAAFAAAKAEW 18
TRIAE_CS42_7AS_TGACv --------------------------------------------------------MAGDGEGAGDGEGAAAFAAAKAEW 24
TRIAE_CS42_7BS_TGACv --------------------------------------------------------------MAGDGEGAAAFAVAKAEW 18
TRIAE_CS42_6DS_TGACv -------------------------------------------------------------------------------- 0
TRIAE_CS42_6BS_TGACv -------------------------------------------------------------------------------- 0
TRIAE_CS42_6AS_TGACv -------------------------------------------------------------------------------- 0
TRIAE_CS42_2BS_TGACv AEIGGALLFALAAAAALFSAVSTGAVDFSHPLAVGGRVDFQETISWFIG------------------------------- 52
TRIAE_CS42_2DS_TGACv AEIGGALLFALAAAAALFAAVSTGAVDFSHPPAVGGRVDFQEAISWFIG------------------------------- 52
TRIAE_CS42_2AS_TGACv AEIGGALLFALAAAAALFAAVSTGAIDFSRPLAVGGRVDFQEAISWFIG------------------------------- 52
TRIAE_CS42_2AS_TGACv GEIGGALLFVLAAAAAVLAAVSTGAVDFSHPPAVGGQLDFQETISWFTG------------------------------- 52
TRIAE_CS42_2DS_TGACv GPNGFIGVFFQKAWAIVKRDVMAALNKLFLNNGRGFGRLNQALITLIPKNHEACQIKDFRPICLVHSIPKLASKLLATRL 208

TRIAE_CS42_3AS_TGACv TAWLWVEVPVRVDWPAVAAQCAWAGEQARAFLVVPAVRLLVLLSLAMTVMILLEKLFVAAV-CYAAKAFGHRPESRYQWR 84
TRIAE_CS42_3B_TGACv1 TAGLWAEVPVRLDWATVAAQCALAGEQARAFLVVPAVRLLVLLSLAMTVMILLEKLFVAAV-CYAAKALGHRPERRYKWG 479
TRIAE_CS42_3B_TGACv1 TAGLWAEVPVRLDWATVAAQCSLAGEQARAFLVVPALRLLVLLSLAMTVMILLEKLFVAAV-CYSAKAFRHRPESRYRWR 84
TRIAE_CS42_3DS_TGACv TVGLREEVPVRLDWATVAAQCAWAGEQTRSFLVVPAVRLLVLLSLAMTVMILLEKLFVAAV-CYAAKAFGHRPESRYKWG 82
TRIAE_CS42_3DS_TGACv WLWAEVPVPVRVDWAAVAAQCAWAGQQARALLVVPTVRLLVLLSLAMTVMILLEKLFVAAV-CYAAKAFGHRPESRYRWR 84
TRIAE_CS42_3DL_TGACv --GVWAEVPVRVDWAAVAAQCAWAGAQARAFLVVPAIRLLVVLSLAMTVMILLEKIFVAAV-CFAAKAFGHRPERRYQWR 87
TRIAE_CS42_3B_TGACv1 --AVWAGLPVRVDWAAVAAQCAWAGMQARAFLVVPAIRLLVLLSLAMTVMILLEKVFVAAV-CFAAKAFGHRPERRYQWR 87
TRIAE_CS42_3AL_TGACv AAGVWAELPVRVDWAAVAAQCAWAGAQARAFLVVPAIRLLLVLSLTMTVMILLEKIFVAAV-CFAAKAFGHKPERRYQWR 90
TRIAE_CS42_6BS_TGACv --------------MDAAVGLPDAWSQVRAPVIVPLLKLAVAVCLLMSVLLFLERLYMAVV-IVGVKLLGRRPERRYKCD 65
TRIAE_CS42_6AS_TGACv --------------MDAAVGLPDAWSQVRAPVIVPLLKLAVALCLLMSVLLFLERLYMAVV-IVGVKLLGRRPERRYKCD 65
TRIAE_CS42_7AL_TGACv -----------MSTLPGVWQIAAAWEQVRGPVIVPLLRASVLLCLAMSAMLFAEKVYMAVV-VLAVRLLGRRPERQWKWE 68
TRIAE_CS42_U_TGACv1_ -----------MSTLPGAWHVAAAWEQVRGPVIVPLLRASVLLCLAMSAMLFAEKVYMAVV-VLAVRLLGRRPERQYQWE 68
TRIAE_CS42_U_TGACv1_ --------MAAALLPGTRITFSGAWQQVRGPVIVPLLRASVLLCVAMSAMLLAEKVYMAVV-VLALRLLGRRPELQYRWE 71
TRIAE_CS42_7BL_TGACv -----------MSTLPRVWQIAAAWEQVRGPVIVPLLRVSVLLCLAMSAMLFAEKVYMAVV-VLAVRLLGRRPEQQYRWE 68
TRIAE_CS42_7AL_TGACv --------------MEAAEQIAVVWKQVRGPVIAPLLRASVMVCLAMCVILFVEKVYMAVV-IVAMRLIGRHPERQWRWE 65
TRIAE_CS42_7BL_TGACv --------------MEAAEQIAVVWKQVRGPVIVPLLRASVMVCLAMCVILFVEKVYMAVV-IVAMRLIGRRPERQWRWE 65
TRIAE_CS42_7DL_TGACv --------------MEAAEQIAVVWKQVRGPVIVPLLRASVMVCLAMCVILFVEKVYMAVV-IVAMRLIGRRPERQWRWE 65
TRIAE_CS42_2AL_TGACv -----------MKGVSMLTMARAAWAAVRHAVVVPLLQLAVYLCAAMSLMLFAERLYMGLV-VAALWLRRRRRQRRNPGR 68
TRIAE_CS42_2DL_TGACv FLLSFGGGRRRMKGVSMLTMARAAWAVVRYAVVVPLLQLAVYLCAAMSLMLFAERLYMGLV-VAALWLRRRRRQRRSPSR 90
TRIAE_CS42_2BL_TGACv -----------MRGVSMLTMARAAWAAVRYAVVVPLLQLAVYLCAAMSLMLFAERLYMGLV-VAALWLRRRRRQRRNPSR 68
TRIAE_CS42_1AS_TGACv --------------MSMLPMARAAWLVLRYAVVVPLLQLAIYLCVVMSLMLFADRLYMGLV-VAVLWLYRRCRNRNQRNK 65
TRIAE_CS42_7DS_TGACv LDGSGGLPLLRWWRASGGGELLGRWDAVRAGAVAPALAAVSGACLAMSAMLLAEAVFMAAA-SLVR----RRPERRYSAG 93
TRIAE_CS42_7AS_TGACv LGGSGGLPLLRWWRASGGGELLRGWDAVRAGAVAPALAAVSGACLAMSAMLLAEAVFMAAA-SLVR----RRPERRYSAG 99
TRIAE_CS42_7BS_TGACv LAGSGGLPLLRWWRASGGGELLRGWDAVRAGAVAPALAAVSGACLAMSAMLLAEAVFMAAA-SLVR----RRPERRYSAG 93
TRIAE_CS42_6DS_TGACv ------------MAPLGADAAAAAWAAVRARAVAPALTAAVWACLAMSAMLLLEAACMSLVSLVAVRLLRLRPERRFKWE 68
TRIAE_CS42_6BS_TGACv ------------MAPLGADAAAAAWAAVRARAVSPALTAAVWACLAMSAMLLLEAACMSLVSLVAVRLLRLRPQRRFKWE 68
TRIAE_CS42_6AS_TGACv ------------MAPLSAGAAAAAWAAVRARAVAPALTAAVWACLAMSAMLLLEAACMSLVSLVAVRLLRLRPERRFKWE 68
TRIAE_CS42_2BS_TGACv -IFDGSSSSSSAAGGVSLAEVYELWVRVRGRVIAPALQVAVWACMVMSVMLVVEALYNCVV-SLGVKAVGWRPEWRFKWE 130
TRIAE_CS42_2DS_TGACv -VFDGSSSSSSAAGGVSLAEVYELWVRVRGRVIAPALQVAVWACMVMSVMLVVEALYNCVV-SLGVKAVGWRPEWRFKWE 130
TRIAE_CS42_2AS_TGACv -VFDGSSSSS-AAGGVSLAEVYELWVRVRGRVIAPALQVAVWACMVMSVMLVVEALYNCVV-SLGVKAVGWRPEWRFKWE 129
TRIAE_CS42_2AS_TGACv -VYNG-ASYSSGAGAVSLAEVHELWVRVRGRVIAPALQVTVWACMVMSVMLAVEALYNCVV-SLGVKAIGWRPEWRFKWE 129
TRIAE_CS42_2DS_TGACv CPRMGELVHAKQSAFIKGRNIHDNFLQVRQLARKLYKRKTKSVMLKLDISRAFDSLSWPFL-FEVLRVKGFSRTWRFWIA 287

TRIAE_CS42_3AS_TGACv PIAASACKTGGDDEEDGIVVVG----SAAFPVVLVQIPMYNEREVYKVSIGAACALEWPSDRMVIQVLDDSTDPVVKDLV 160
TRIAE_CS42_3B_TGACv1 PVAASACKTGGDDEEDGIVGVGSGSGSAAFPVVLVQIHMYNER---------------------------------EDLV 526
TRIAE_CS42_3B_TGACv1 PITASACKTGGDDEEDGIVVVGSGSGRAAFPVVLVQIPMYNEREVYKVSIGAACALEWPSDRMVIQVLDDSTDPVVKELV 164
TRIAE_CS42_3DS_TGACv PIAASACKTGGDDEEDGIVVVGSGSGSGAFPVVLVQIPMYNEREVYKVSIGAACALEWPSDRMVIQVLDDSTDPVVKELV 162
TRIAE_CS42_3DS_TGACv PIAASACKAGGGDEEDGIVIVGSSSGSAAFPVVLVQIPMYNEREVYKVSIGAACALEWPSDRMVIQVLDDSTDPVVKDLV 164
TRIAE_CS42_3DL_TGACv PIAAGAAAAARGDEE--AGLVGGGGGSAAFPVVLVQIPMYNEREVYKLSIGAACALEWPSDRVVIQVLDDSTDPAVKDLV 165
TRIAE_CS42_3B_TGACv1 PIAAGAAAAARGDEE--AGVGGGG--SAAFPVVLVQIPMYNEREVYKLSIGAACALEWPAERVVIQVLDDSTDPVVKDLV 163
TRIAE_CS42_3AL_TGACv PIAASACKTGGVDEE--ASVGGGS---SAFPVVLVQIPMYNEREVYKLSIGAACALEWPSDRVVIQVLDDSTDPAVKDLV 165
TRIAE_CS42_6BS_TGACv PICEDDDPE---------------LGSAAFPIVLVQIPMFNEREVYQLSIGAVCGLSWPSDRLVVQVLDDSTDPLIKEMV 130
TRIAE_CS42_6AS_TGACv PICEDDDPE---------------LGSAAFPVVLVQIPMFNEREVYQLSIGAVCGLSWPSDRLVVQVLDDSTDPLVKEMV 130
TRIAE_CS42_7AL_TGACv PVGE-DDPE---------------LGSAAYPMVLVQIPMYNEREVYQLSIGAACGLSWPSDRIVVQVLDDSTDPVIKELV 132
TRIAE_CS42_U_TGACv1_ PMGD-DDPE---------------LGSAAYPMVLVQIPMYNEREVYQLSIGAACGLSWPSDRIVVQVLDDSTDPVIKELV 132
TRIAE_CS42_U_TGACv1_ PMRDGDDPE---------------LGSAAYPMVLVQIPMYNEREVYQLSIGAACGLSWPSDRIIVQVLDDSTDPVVKELV 136
TRIAE_CS42_7BL_TGACv PVGDGNDPE---------------LGSAAYPMVLVQIPMYNEREVYQLSIGAACGLSWPSDRIIVQVLDDSTDPVIKELV 133
TRIAE_CS42_7AL_TGACv PLRD-DDPE---------------LGNAAYPMVLVQIPMYNEREVYKKSIGAACGLSWPSDRIVIQVLDDSTDPAIKELV 129
TRIAE_CS42_7BL_TGACv PLRD-DDPE---------------LGNAAYPMVLVQIPMYNEREVYKKSIGAVCGLSWPSDRIVIQVLDDSTEPAIKELV 129
TRIAE_CS42_7DL_TGACv PLRD-DDPE---------------LGNAAYPMVLVQIPMYNEREVYKKSIGAACGLSWPSDRIVIQVLDDSTDPAIKELV 129
TRIAE_CS42_2AL_TGACv NKGGDDDVG-----------DLESGAAEDLPVVLVQIPMFNEKQVYRLSIGAACGLWWPADKLVIQVLDDSTDAGIRAMV 137
TRIAE_CS42_2DL_TGACv NKGGDDDD-------------LESGAAEDLPLVLVQIPMFNEKQVYRLSIGAACGLWWPADKLVIQVLDDSTDAGIRAMV 157
TRIAE_CS42_2BL_TGACv NKGDDDGGAG----------DLESGGGEDLPMVLVQIPMFNEKQVYRLSIGAACGLWWPADKLVIQVLDDSTDAGIRALV 138
TRIAE_CS42_1AS_TGACv GDDDNLESD-----------------DADRPMVLVQIPMFNEKQVFRLSIGAACGLWWPADKLVIQVLDDSTDAGIRSLV 128
TRIAE_CS42_7DS_TGACv PLGAQDGEDE-------------ERGLLGYPMVLVQIPMYNEREVYKLSIGAACGLSWPSDRVIVQVLDDSTDPTIKDLV 160
```

```
TRIAE_CS42_7AS_TGACv PLGAQDGEDE-------------ERGLLGYPMVLVQIPMYNEREVYKLSIGAACGLSWPSDRVIVQVLDDSTDPTIKDLV 166
TRIAE_CS42_7BS_TGACv PLGAQDGEDED-----------EERGLLGYPMVLVQIPMYNEREVYKLSIGAACGLSWPSDRVIVQVLDDSTDPTIKDLV 162
TRIAE_CS42_6DS_TGACv PMAGALEGGEADVED-----PPASAGRREFPMVLVQIPMYNEKEVYKLSIGAVCALTWPPDRIIIQVLDDSTDPIIKELV 143
TRIAE_CS42_6BS_TGACv PMPGALPGAEADAED-----PPG---RREFPMVLVQIPMYNEKEVYKLSIGAVCALTWPPDRIIIQVLDDSTDPIIKELV 140
TRIAE_CS42_6AS_TGACv PMTGALEGGEADVED-----PAG---RREFPMVLVQIPMYNEKEVYKLSIGAVCALTWPPDRIIIQVLDDSTDPIIKELV 140
TRIAE_CS42_2BS_TGACv PLAGDDEEKGG----------------AHYPMVLVQIPMYNELEVYKLSIGAACELQWPKDRIIVQVLDDSTDPFIKNLV 194
TRIAE_CS42_2DS_TGACv PLAGDDEEKGG----------------AHYPMVLVQIPMYNELEVYKLSIGAACELQWPKDRIIVQVLDDSTDPFIKNLV 194
TRIAE_CS42_2AS_TGACv PLAGDDEEKGG----------------AHYPVVLVQIPMYNELEVYKLSIGAACELQWPKDRIIVQVLDDSTDPFIKNLV 193
TRIAE_CS42_2AS_TGACv PLAGD-EEKGS----------------AHYPMVLVQIPMYNELEVYKLSIGAACELKWPKDRMIVQVLDNSTDPLIKNLV 192
TRIAE_CS42_2DS_TGACv TLLTTASSRVV----------------VNGCVGKKFMHACGLRQGDSISPLLFVIAMDVLSAMILKARETNAVSKIPGCA 351

TRIAE_CS42_3AS_TGACv KIECQRWKSKGVNIRYEVRQNRKGYKAGALKEGLMRD---------------------------------------YVRE 201
TRIAE_CS42_3B_TGACv1 KIECQRWKSKGVNIRYEVRQNRKGYKAGALKEGLIRD---------------------------------------YVRE 567
TRIAE_CS42_3B_TGACv1 KTECQRWKGKGVNIRYEVRGNRKGYKAGALKQGLMRD---------------------------------------YVRE 205
TRIAE_CS42_3DS_TGACv KTECQRWKGKGVNIRYEVRGNRKGYKAGALKQGLMRD---------------------------------------YVRE 203
TRIAE_CS42_3DS_TGACv KIECQRWKSKGVNIRYEVRENRKGYKAGALKQGLMRD---------------------------------------YVRE 205
TRIAE_CS42_3DL_TGACv EIECQRWKGKGVNIKYEVRGNRKGYKAGALKEGLKHD---------------------------------------YVQE 206
TRIAE_CS42_3B_TGACv1 EIECQRWKGKGVNIKYEVRGNRKGYKAGALKEGLKHD---------------------------------------YVQE 204
TRIAE_CS42_3AL_TGACv EIECQRWKGKGVNIKYEVRGNRKGYKAGALKEGLKHD---------------------------------------YVQE 206
TRIAE_CS42_6BS_TGACv RMECERWAHKGINITYQIREDRKGYKAGALKAGMKHG---------------------------------------YVRE 171
TRIAE_CS42_6AS_TGACv RMECERWAHKGINITYQIREDRKGYKAGALKAGMKHG---------------------------------------YVRE 171
TRIAE_CS42_7AL_TGACv QVECRRWARKGVNIKYEIRDNRRGYKAGALKEGMKHG---------------------------------------YVKD 173
TRIAE_CS42_U_TGACv1_ RVECRRWARKGVNIKYEIRDNRRGYKAGALKEGMKHG---------------------------------------YVKD 173
TRIAE_CS42_U_TGACv1_ QVECQRWARKGVNIKYETRNNRRGYKAGALKEAMKHG---------------------------------------YVKD 177
TRIAE_CS42_7BL_TGACv QVECRRWARKGVNIKYEIRDNRRGYKAGALKEGMKHG---------------------------------------YVKD 174
TRIAE_CS42_7AL_TGACv QAECHRWANKGVNIKYEIRDNRRGYKAGALKEGMKHG---------------------------------------YVKD 170
TRIAE_CS42_7BL_TGACv QVECQRWANKGVNIKYEIRDNRRGYKAGALKEGMKHG---------------------------------------YVKD 170
TRIAE_CS42_7DL_TGACv QVECQRWANKGVNIKYEIRDNRRGYKAGALKEGMKHG---------------------------------------YVKD 170
TRIAE_CS42_2AL_TGACv EAECRRWAGKGVHIRYENRSNRSGYKAGAMREGLKKG---------------------------------------YAKD 178
TRIAE_CS42_2DL_TGACv EAECRRWAGKGVQIRYENRSNRSGYKAGAMREGLKKG---------------------------------------YAKD 198
TRIAE_CS42_2BL_TGACv EAECRRWAGKGVQIRYENRSNRSGYKAGAMREGLKKG---------------------------------------YARD 179
TRIAE_CS42_1AS_TGACv EAECRRWAGKGVHIRYENRSNRSGYKAGAMRDGLKKQ---------------------------------------YVKD 169
TRIAE_CS42_7DS_TGACv ELECKIWAKKGKNVKYEVRNNREGYKAGALKEGMLHA---------------------------------------YVQQ 201
TRIAE_CS42_7AS_TGACv ELECKIWAKKGKNVKYEVRNNREGYKAGALKEGMLHA---------------------------------------YVQQ 207
TRIAE_CS42_7BS_TGACv ELECKIWAKKGKNVKYEVRNNREGYKAGALKEGMLHA---------------------------------------YVQQ 203
TRIAE_CS42_6DS_TGACv ELECQEWASKKIDIKYEVRNNRKGYKAGALKKGMEHV---------------------------------------YAQQ 184
TRIAE_CS42_6BS_TGACv ELECQEWASKKIDIKYEVRNNRKGYKAGALKKGMEHV---------------------------------------YAQQ 181
TRIAE_CS42_6AS_TGACv ELECQEWASKKIDIKYEVRNNRKGYKAGALKKGMEHV---------------------------------------YAQQ 181
TRIAE_CS42_2BS_TGACv ELECESWAVKGLNIKYATRSSRKGFKAGALKKGMECD---------------------------------------YAKQ 235
TRIAE_CS42_2DS_TGACv ELECESWAVKGLNIKYATRSSRKGFKAGALKKGMECD---------------------------------------YAKQ 235
TRIAE_CS42_2AS_TGACv ELECESWSVKGLNIKYATRSSRKGFKAGALKKGMEYD---------------------------------------YAKQ 234
TRIAE_CS42_2AS_TGACv ELECETWVTKGLNIKYAPRSGQKGFKAGALKKGMECD---------------------------------------YARQ 233
TRIAE_CS42_2DS_TGACv PIQRLSLYVDDVVMFIKPSWTDLWFVQEALRVFGEASGLKVNFSKSSAVMIRSEEEEEVLVRKAMPWKMETFPIKYLGLQ 431

TRIAE_CS42_3AS_TGACv CEFIAMFDADIQPESDFLLRTVPFLVHN---------------------------------------------------- 229
TRIAE_CS42_3B_TGACv1 CEFIAMFDADIQPESDFLLRTVPFLVHN---------------------------------------------------- 595
TRIAE_CS42_3B_TGACv1 CEFIAMFDADIQPESDFLLRTVPFLVHN---------------------------------------------------- 233
TRIAE_CS42_3DS_TGACv CKFIAMFDADIQPESDFLLRTVPFLVHN---------------------------------------------------- 231
TRIAE_CS42_3DS_TGACv CEFIAMFDADIQPESDFLLRTVPFLVHN---------------------------------------------------- 233
TRIAE_CS42_3DL_TGACv CEFIAMFDADIQPESDFLLRTVPFLVHN---------------------------------------------------- 234
TRIAE_CS42_3B_TGACv1 CEFIAMFDADIQPESDFLLRTVPFLVHN---------------------------------------------------- 232
TRIAE_CS42_3AL_TGACv CEFIAMFDADIQPESDFLLRTVPFLVHN---------------------------------------------------- 234
TRIAE_CS42_6BS_TGACv CEYMVIFDADIQPDPDFLHRTIPYLHHN---------------------------------------------------- 199
TRIAE_CS42_6AS_TGACv CEYMVIFDADIQPDPDFLHRTIPYLHHN---------------------------------------------------- 199
TRIAE_CS42_7AL_TGACv CDLVAIFDADIQPEPDFLWRAVPFLVHN---------------------------------------------------- 201
TRIAE_CS42_U_TGACv1_ CDLVAIFDADI------LWRAVPFLVHN---------------------------------------------------- 195
TRIAE_CS42_U_TGACv1_ CDLVAIFDADIQPEPDFLSRSVPFLVHN---------------------------------------------------- 205
TRIAE_CS42_7BL_TGACv CDLVAIFDADIQPEPDFLSRSVPFLVHN---------------------------------------------------- 202
TRIAE_CS42_7AL_TGACv CDYVVIFDADIQPEPDYLSRAMPFLVHN---------------------------------------------------- 198
TRIAE_CS42_7BL_TGACv CDFVVIFDADIQPEPDYLSRAMPFLIHN---------------------------------------------------- 198
TRIAE_CS42_7DL_TGACv CDFVVIFDADIQPEPDYLSRAMPFLIHN---------------------------------------------------- 198
TRIAE_CS42_2AL_TGACv CELVAVFDADIQPDADFLRRTVPVLQAD---------------------------------------------------- 206
TRIAE_CS42_2DL_TGACv CELVAVFDADIQPDADFLRRTVPVLQAD---------------------------------------------------- 226
TRIAE_CS42_2BL_TGACv CELVAVFDADIQPDADFLRRTVPVLQAD---------------------------------------------------- 207
TRIAE_CS42_1AS_TGACv CEFVAVFDADIQPDADFLRHTVPVLEAD---------------------------------------------------- 197
TRIAE_CS42_7DS_TGACv CDFLAVFDADIQPEPDFLMRTIPYLARN---------------------------------------------------- 229
TRIAE_CS42_7AS_TGACv CDFLAVFDADIQPEPDFLMRTIPYLARN---------------------------------------------------- 235
TRIAE_CS42_7BS_TGACv CDFLAVFDADIQPEPDFLMRTIPYLSRN---------------------------------------------------- 231
TRIAE_CS42_6DS_TGACv CEFVAIFDADIQPESDFLLKTIPFLVHN---------------------------------------------------- 212
TRIAE_CS42_6BS_TGACv CEFVAIFDADIQPESDFLLKTIPFLVHN---------------------------------------------------- 209
TRIAE_CS42_6AS_TGACv CEFVAIFDADIQPESDFLLKTIPFLVHN---------------------------------------------------- 209
TRIAE_CS42_2BS_TGACv CEYVAIFDADIQPEPDFLLRTVPFFVHN---------------------------------------------------- 263
TRIAE_CS42_2DS_TGACv CEYVAIFDADIQPEPDFLLRTVPFFVHN---------------------------------------------------- 263
TRIAE_CS42_2AS_TGACv CEYVAIFDADIQPEPDFLLRTVPFFVHN---------------------------------------------------- 262
TRIAE_CS42_2AS_TGACv CEYVAIFDADIQPEPDFLLRTIPFFVHN---------------------------------------------------- 261
TRIAE_CS42_2DS_TGACv LGIKQLTRSEVQPVVDQALKMMPGWQRGPVTRPGRLPLVNQVVRARPIHHLIVAEAPKRALDRVDKGCRAFFWAGSEEIQ 511

TRIAE_CS42_3AS_TGACv ----------------------------------------------------PDIALVQTRWKFVNSDECLLTRFQEMSL 257
TRIAE_CS42_3B_TGACv1 ----------------------------------------------------PDIALVQTRWKFVNSDECLLTRFLEMSL 623
TRIAE_CS42_3B_TGACv1 ----------------------------------------------------PDIALVQTRWKFVNSDKCLLTRFQEMSL 261
TRIAE_CS42_3DS_TGACv ----------------------------------------------------PDIALVQTRWKFVNSDKCLLTRFQEMSL 259
TRIAE_CS42_3DS_TGACv ----------------------------------------------------PDIALVQTRWKFVNSDECLLTRFQEMSL 261
TRIAE_CS42_3DL_TGACv ----------------------------------------------------PDIALVQTRWKFVNSDECLLTRFQEMSL 262
TRIAE_CS42_3B_TGACv1 ----------------------------------------------------PDIALVQTRWKFVNSDECLLTRFQEMSL 260
TRIAE_CS42_3AL_TGACv ----------------------------------------------------PDIALVQTRWKFVNSDECLLTRFQEMSL 262
TRIAE_CS42_6BS_TGACv ----------------------------------------------------PEIALVQARWRFVNADECLMTRMQEMSL 227
TRIAE_CS42_6AS_TGACv ----------------------------------------------------PEIALVQARWRFVNADECLMTRMQEMSL 227
TRIAE_CS42_7AL_TGACv ----------------------------------------------------PDIALVQARWKFVNADECLMTRMQEMSL 229
TRIAE_CS42_U_TGACv1_ ----------------------------------------------------PDIALVQARWKFVNADECLMTRMQEMSL 223
TRIAE_CS42_U_TGACv1_ ----------------------------------------------------PDIALVQARWKFVNADECLMTRMQEMSL 233
TRIAE_CS42_7BL_TGACv ----------------------------------------------------PDIALVQARWKFVNADECLMTRMQEMSL 230
TRIAE_CS42_7AL_TGACv ----------------------------------------------------PEIALVQARWVFVNANECLMTRMQEMSL 226
TRIAE_CS42_7BL_TGACv ----------------------------------------------------PEIALVQARWVFVNANECLMTRMQEMSL 226
TRIAE_CS42_7DL_TGACv ----------------------------------------------------PEIALIQARWVFVNANECLMTRMQEMSL 226
TRIAE_CS42_2AL_TGACv ----------------------------------------------------PAVALVQARWRFVNADECILTRIQEMSL 234
```

```
TRIAE_CS42_2DL_TGACv ----------------------------------------------------PSVALVQARWRFVNADECILTRIQEMSL 254
TRIAE_CS42_2BL_TGACv ----------------------------------------------------PAVALVQARWRFVNADECILTRIQEMSL 235
TRIAE_CS42_1AS_TGACv ----------------------------------------------------PAVALVQARWRFVNADECILTRMQEMSL 225
TRIAE_CS42_7DS_TGACv ----------------------------------------------------PQIALVQARWEFVNPNECLMTRIQKMTL 257
TRIAE_CS42_7AS_TGACv ----------------------------------------------------PQIALVQARWEFVNPNECLMTRIQKMTL 263
TRIAE_CS42_7BS_TGACv ----------------------------------------------------PQIALVQARWEFVNPNECLMTRIQKMTL 259
TRIAE_CS42_6DS_TGACv ----------------------------------------------------PKIALVQTRWKFVNYDACLMTRIQKMSL 240
TRIAE_CS42_6BS_TGACv ----------------------------------------------------PKIALVQTRWKFVNYDACLMTRIQKMSL 237
TRIAE_CS42_6AS_TGACv ----------------------------------------------------PKIALVQTRWKFVNYDACLMTRIQKMSL 237
TRIAE_CS42_2BS_TGACv ----------------------------------------------------PEVALVQARWSFVNDTASLLTRVQKMFF 291
TRIAE_CS42_2DS_TGACv ----------------------------------------------------PEVALVQARWSFVNDTASLLTRVQKMFF 291
TRIAE_CS42_2AS_TGACv ----------------------------------------------------PEVALVQARWSFVNDNASLLTRVQKMFF 290
TRIAE_CS42_2AS_TGACv ----------------------------------------------------PKVALVQARWSFVNGTVSLLTRIQKMFF 289
TRIAE_CS42_2DS_TGACv GGQCAVAWRGVYRPKQMGGLGVVDLHKHGIALRLSLSQTSFSEQSRSSCTIQKLLLFKLSGPSNLNGTVSLLTRIQKMFF 591

TRIAE_CS42_3AS_TGACv DYHFKFEQEAGSIVYSFFGFNGTAGVWRISAINDAGGWKERTTVEDMDLAVRTALLGLKFVYVGAVKVKSELPSTFKAYR 337
TRIAE_CS42_3B_TGACv1 DYHFKFEQEAGSIVYSFFGFNGTAGVWRISAINDAGGWKERTMVEDMDLVVRTALLGLKFVYIGAVKVKSELPSTFKAYR 703
TRIAE_CS42_3B_TGACv1 DYHFKFEQEAGSIVYSFFGFNGTAGVWRISAINDAGGWEDRTTVEDMDLAVRTSLLGWKFVYVGAVKVKSELPSTFKAYR 341
TRIAE_CS42_3DS_TGACv DYHFKFEQEAGSIVYSFFGFNGTAGVWRISAINDAGGWEDRTTVEDMDLAVRTALLGWKFVYVGAVKVGSELPSTFKAYR 339
TRIAE_CS42_3DS_TGACv DYHFKFEQEAGSIVYSFFGFNGTAGVWRISAINDAGGWNDRTTVEDMDLAVRTALLGWKFVYVGDVKVRSELPSTFKAYR 341
TRIAE_CS42_3DL_TGACv DYHFKFEQEAGSIVYSFFGFNGTAGVWRISAIDDAGGWKDRTTVEDMDLAVRTALKGWKFVYVGAVKVRSELPSTFKAYR 342
TRIAE_CS42_3B_TGACv1 DYHFKFEQEAGSIVYSFFGFNGTAGVWRIAAIDDAGGWKDRTTVEDMDLAVRTALKGWKFVYVGAVKVRSELPSTFKAYR 340
TRIAE_CS42_3AL_TGACv DYHFKFEQEAGSIVYSFFGFNGTAGVWRISAIDDAGGWKDRTTVEDMDLAVRTALKGWKFVYVGAVKVRSELPSTFKAYR 342
TRIAE_CS42_6BS_TGACv DYHFKVEQEVSSSVCAFFGFNGTAGVWRIAAVNEAGGWKDRTTVEDMDLAIRASLKGWKFVYLGDVQVKSELPSTFKAFR 307
TRIAE_CS42_6AS_TGACv DYHFKVEQEVSSSVCAFFGFNGTAGVWRIAAVNEAGGWKDRTTVEDMDLAIRASLKGWKFVYLGDVQVKSELPSTFKAFR 307
TRIAE_CS42_7AL_TGACv DYHFRVEQEVGSSTYAFFGFNGTAGVWRISALNEAGGWKDRTTVEDMDLAVRASLKGWKFVYLGDLKVKNELPSTFKAFR 309
TRIAE_CS42_U_TGACv1_ DYHFKVEQEVGSSTYAFFGFNGTAGVWRISALNEAGGWKDRTTVEDMDLAVRASLKGWKFVYLGDLKVKNELPSTFKAFR 303
TRIAE_CS42_U_TGACv1_ DYHFKVEQEVGSSTYAFFGFNGTAGVWRISALNEAGGWKDRTTVEDMDLAVRASLKGWKFVYLGDLKVKNELPSTFKAFR 313
TRIAE_CS42_7BL_TGACv DYHFKVEQEVGSSTYAFFGFNGTAGVWRISALNEAGGWKDRTTVEDMDLAVRASLKGWKFVYIGDLKVKNELPSTFKAFR 310
TRIAE_CS42_7AL_TGACv DYHFKVEQEVGSSAYAFFGFNGTAGVWRISALNEAGGWKDRTTVEDMDLAVRASLKGWKFVCLGDLRVKSELPSTFKAFR 306
TRIAE_CS42_7BL_TGACv DYHFKVEQEVGSSAYAFFGFNGTAGVWRISALNEAGGWKDRTTVEDMDLAVRASLKGWKFVYLGDLRVKSELPSTFKAFR 306
TRIAE_CS42_7DL_TGACv DYHFKVEQEVGSSAYAFFGFNGTAGVWRISALNEAGGWKDRTTVEDMDLAVRASLKGWKFVYLGDLRVKSELPSTFKAFR 306
TRIAE_CS42_2AL_TGACv DYHFSVEQEVGSACHGFFGFNGTAGVWRVQALADAGGWKDRTTVEDMDLAVRASMRGWRFVYAGDVQVRNELPSSFKAYR 314
TRIAE_CS42_2DL_TGACv DYHFSVEQEVGSACHGFFGFNGTAGVWRVQALADAGGWKDRTTVEDMDLAVRASMRGWRFVYAGDVQVRNELPSSFKAYR 334
TRIAE_CS42_2BL_TGACv DYHFSVEQEVGSACHGFFGFNGTAGVWRVQALADAGGWKDRTTVEDMDLAVRASMRGWRFVYAGDVQVRNELPSSFKAYR 315
TRIAE_CS42_1AS_TGACv DYHFSVEQEVGSAFHGFFSFNGTAGVWRLHALADAGGWKDRTTVEDMDLAVRASMRGWRFVYAGDVQVRNELPSSFKAYR 305
TRIAE_CS42_7DS_TGACv DYHFKVEQEAGSSTFAFFGFNGTAGVWRISAIKEAGGWDDRTTVEDMDLAVRAGLKGWKFVYVGDVKVKSELPSNLKAYR 337
TRIAE_CS42_7AS_TGACv DYHFKVEQEAGSSTFAFFGFNGTAGVWRISAIKEAGGWDDRTTVEDMDLAVRAGLKGWKFVYVGDVKVKSELPSNLKAYR 343
TRIAE_CS42_7BS_TGACv DYHFKVEQEAGSSTFAFFGFNGTAGVWRISAIKEAGGWDDRTTVEDMDLAVRAGLKGWKFVYVGDVKVKSELPSNLKAYR 339
TRIAE_CS42_6DS_TGACv DYHFKVEQESGSFMHAFFGFNGTAGVWRVSAINESGGWKDRTTVEDMDLAVRACLKEWEFLYVGDIRVKSELPSTFKAYR 320
TRIAE_CS42_6BS_TGACv DYHFKVEQESGSFMHAFFGFNGTAGVWRVSAINESGGWKDRTTVEDMDLAVRACLKEWEFLYVGDIRVKSELPSTFKAYR 317
TRIAE_CS42_6AS_TGACv DYHFKVEQESGSFMHAFFGFNGTAGVWRVSAINESGGWKDRTTVEDMDLAVRACLKEWEFLYVGDIRVKSELPSTFKAYR 317
TRIAE_CS42_2BS_TGACv DYHFKVEQEAGSATFSFFSFNGTAGVWRTSAIKEAGGWKDRTTVEDMDLAVRATLKGWKFVYVGDIRVKSELPSTYKAYC 371
TRIAE_CS42_2DS_TGACv DYHFKVEQEAGSATFSFFSFNGTAGVWRTAAIKEAGGWKDRTTVEDMDLAVRATLKGWKFVYVGDIRVKSELPSTYKAYC 371
TRIAE_CS42_2AS_TGACv DYHFKVEQEAGSATFSFFSFNGTAGVWRAAAIKEAGGWKDRTTVEDMDLAVRATLKGWKFVYVGDIRVKSELPSTYKAYC 370
TRIAE_CS42_2AS_TGACv DYHFKVEQEAGSATFAFFSFNGTAGVWRTAAIKEAGGWKDRTTVEDMDLAIRATLKGWKFIYVGDIRVKSELPSSYKAYC 369
TRIAE_CS42_2DS_TGACv DYHFKVEQEAGSATFAFFSFNGTAGVWRTAAIKEAGGWKDRTTVEDMDLAIRATLKGWKFIYVGDIRVKSELPSSYKAYC 671

TRIAE_CS42_3AS_TGACv FQQHRWSCGPANLFKKVLVEILHNKKVSFWCKLHLLYDFFFVGKITAHTVTFIYYCFAIPVSVFFP--EIQIPLWCVVYV 415
TRIAE_CS42_3B_TGACv1 FQQHRWSCGPANLFKKMLVEILQNKKVSFWSKLHLLYDFFFVGKIIAHIVTFIYYCFATPVSVFFP--EIQIPLWGVVYV 781
TRIAE_CS42_3B_TGACv1 FQQHRWSCGPANLFKKILLDILKNKKVSFWSKLHLLYDFFFVGKIAAHTVTFIYYCFAIPVSVFFP--EIQIPLWGVVYV 419
TRIAE_CS42_3DS_TGACv FQQHRWSCGPANLFKKMLLDILRKKKVSFWSKLHLLYDFFFVGKIAAHMVTFIYYCFAIPVSVFFP--EIQIPLWGVVYV 417
TRIAE_CS42_3DS_TGACv FQQHRWSCGPANLFKKMLVDILENKKVSFWSKLHLLYDFFFVGKIAAHTLTFIYYCFAIPVSVFFP--EIQIPLWGVVYV 419
TRIAE_CS42_3DL_TGACv FQQHRWSCGPANLFKKMLVEILESKKVSFWSKLHLLYDFFFVGKIAAHTVTFIYYCFAIPLSVFFP--EIQIPLWGVVYV 420
TRIAE_CS42_3B_TGACv1 FQQHRWSCGPANLFKKMLVEILESKKVSFWSKLHLLYDFFFVGKIAAHTVTFIYYCFAIPLSVFFP--EIQIPLWGVVYV 418
TRIAE_CS42_3AL_TGACv FQQHRWSCGPANLFKKMLVEILENKKVSFWSKLHLLYDFFFVGKIAAHTVTFIYYCFAIPLSVFFP--EIQIPLWGVVYV 420
TRIAE_CS42_6BS_TGACv FQQHRWSCGPANLFRKMLLEIVTNKKVTIWKKFHVIYNFFLVRKIVAHIVTFTFYCIIIPTTIFVP--EVHIPKWGCVYI 385
TRIAE_CS42_6AS_TGACv FQQHRWSCGPANLFRKMLVEIVTNKKVTIWKKFHVIYNFFLVRKIVAHIVTFTFYCIIIPTTIFVP--EVHIPKWGCVYI 385
TRIAE_CS42_7AL_TGACv YQQHRWSCGPANLFRKMVMEIIRNKKVTLWKKIHVVYSFFLVRKVVAHIVTFVFYCLVIPATVLVP--EVEVPKWGCVYI 387
TRIAE_CS42_U_TGACv1_ YQQHRWSCGPANLFRKMVMEIIRNKKVTLWKKIHVVYSFFLVRKVVAHIVTFVFYCLVIPATVLVP--EVEVPKWGCVYI 381
TRIAE_CS42_U_TGACv1_ YQQHRWSCGPANLFRKMVMEIIKNKKVTLWKKIHVVYNFFFLRKVVAHIMTFVFYCLVIPATVLVP--EVEVPKWGCVYI 391
TRIAE_CS42_7BL_TGACv YQQHRWSCGPANLFRKMVMEIVRNKKVTLWKKIHVIYNFFLVRKVVAHIVTFVFYCVVERIDMVD--------------- 375
TRIAE_CS42_7AL_TGACv YQQHRWSCGPANLFRKMLMEIVKNQKVTLWKKIYVIYNFFLVRKIIGHILTSVFYCLVIPATVFVP--EVEIPRWGYFYI 384
TRIAE_CS42_7BL_TGACv YQQHRWSCGPANLFRKMLMEIVKNQKVTLWKKIYVIYNFFFVRKIIGHILTSVFYCLVIPATVFVP--EVEIPRWGYFYI 384
TRIAE_CS42_7DL_TGACv YQQHRWSCGPANLFRKMLMEIVKNQKVTLWKKIYVIYNFFFVRKIIGHILTSVFYCLVIPATVFVP--EIEIPRWGYFYI 384
TRIAE_CS42_2AL_TGACv YQQHRWSCGPANLMRKMFWEIVASRQVSAWKKVHVLYGFFFVRKVVAHLVTFLFYCVVIPAYVLVGGQDVRLPKYVAMYV 394
TRIAE_CS42_2DL_TGACv YQQHRWSCGPANLMRKMFWEIVASRQVSAWKKVHVLYGFFFVRKVVAHLVTFLFYCVVIPAYVLVGGQDVRLPKYVAMYV 414
TRIAE_CS42_2BL_TGACv YQQHRWSCGPANLMRKMFWEIVASRQVSAWKKVHVLYGFFFVRKVVAHLVTFLFYCVVIPAYVLVGGQDVRLPKYVAMYV 395
TRIAE_CS42_1AS_TGACv YQQHRWSCGPPNLMRKMFWEIVANKQVSAWKKLHVLYGFFFVRKVVAHLATFLFCCVVIPVYVLVGGQDVWLPQYVPMYV 385
TRIAE_CS42_7DS_TGACv RQQHRWTCGAANLFRKMGAEILLTKEVSLWWKLYLLYSFFLVRKVVAHVVPFVLYCVVIPFSVLIP--EIKIPAWGVVYI 415
TRIAE_CS42_7AS_TGACv RQQHRWTCGAANLFRKMGAEILLTKEVSLWWKLYLLYSFFLVRKVVAHVLPFVLYCVVIPFSVLIP--EIKIPAWGVVYI 421
TRIAE_CS42_7BS_TGACv RQQHRWTCGAANLFRKMGAEILLTKEVSLWWKLYLLYSFFLVRKVVAHVVPFVLYCVVIPFSVLIP--EIKIPAWGVVYI 417
TRIAE_CS42_6DS_TGACv HQQHRWTCGAANLFRKMGWEIVTNKGVSIWKKWHLLYSFLFVRRVIAPILTFLFYCVVIPLSAMVP--EVHIPVWGLVYI 398
TRIAE_CS42_6BS_TGACv HQQHRWTCGAANLFRKMGWEIVTNKGVSIWKKWHLLYSFLFVRRVIAPILTFLFYCVVIPLSAMVP--EVHIPVWGLVYI 395
TRIAE_CS42_6AS_TGACv HQQHRWTCGAANLFRKMGWEIVTNKGVSIWKKWHLLYSFLFVRRVIAPILTFLFYCVVIPLSAMVP--EVHIPVWGLVYI 395
TRIAE_CS42_2BS_TGACv RQQFRWSCGGAHLFRKVAKDILTAKDVSLIKKFHMLYSFFLVRRVVAPTVACILYNIILPISVMIP--ELFLPVWGIAYI 449
TRIAE_CS42_2DS_TGACv RQQFRWSCGGAHLFRKVAKDILTAKDVSLIKKFHMLYSFFLVRRVMAPTVACILYNIILPISVMIP--ELFLPVWGIAYI 449
TRIAE_CS42_2AS_TGACv RQQFRWSCGGAHLFRKVAKDILTAKDVSLIKKFHMLYSFFLVRRVVAPTLACILYNIILPISVMIP--ELFLPIWGIAYI 448
TRIAE_CS42_2AS_TGACv RQQFRWACGGANLFRKVAIDILTSKDVSVVKKFYMLYSFLFVRRVVAPAVACILSNIIVPLSVMIP--ELYLPVWGVAYI 447
TRIAE_CS42_2DS_TGACv RQQFRWACGGANLFRKVAVDILTSKDVSVIKKFYMLYSFLFVRRVVAPAVACILSNIIVPLSVMIP--ELYLPVWGVAYI 749

TRIAE_CS42_3AS_TGACv PTVITLCKALGSPSSFHLVILWVLFDNVMSLHRIKATITGLLDARRVNEWVVTEKLGDANKTEPAVEGLNDVQVIDVELS 495
TRIAE_CS42_3B_TGACv1 PTVITLCKALGSPSSFHLVILWVLFDNVMSLHRIKATITGLLDARRVNEWVVTEKLGDTNKTEPAMEGLNDVQVIDVELS 861
TRIAE_CS42_3B_TGACv1 PTVITLCKALGSPSSFHLVILWVLFDNVMSLHRIKATITGLLDTRRVNEWVVTEKLGDANKTEPAMERLDDVQVIDVELS 499
TRIAE_CS42_3DS_TGACv PTVITLCKALGSPSSFHLVILWVLFDNVMSLHRIKATITGLLDTRRVNEWVVTEKLGDANKTEPAMEGLDDVQVIDVELS 497
TRIAE_CS42_3DS_TGACv PTVITLCKSLGSPSSFHLVILWVLFENVMSLHRIKATITGLLDTRRVNEWVVTEKLGDANKTEPAMEGLDDVQVIDVELS 499
TRIAE_CS42_3DL_TGACv PTVITLCKALGSPSSFHLVILWVLFENVMSLHRIRAAITGLLDAGRVNEWVVTEKLGDANKTKPATEVLDAVKVIDVELT 500
TRIAE_CS42_3B_TGACv1 PTVITLCKALGSPSSFHLVILWVLFENVMSLHRIRAAVTGLLDAGRVNEWVVTEKLGDANKTKPAMEVLDAVKVIDVELT 498
TRIAE_CS42_3AL_TGACv PTVITLCKALGSPSSFHLVILWVLFENVMSLHRIKAAVTGLLDAGRVNEWVVTEKLGDANKTKPAMEALDAVKVIDVELA 500
TRIAE_CS42_6BS_TGACv PTIITLLNSVGTPRSFHLLFFWILFENVMSLHRTKATLIGLLEAGRANEWVVTEKLG-----------------SAMKMK 448
TRIAE_CS42_6AS_TGACv PTIITLLNSVGTPRSFHLLFFWILFENVMSLHRTKATLIGLLEAGRANEWVVTEKLG-----------------SAMKMK 448
TRIAE_CS42_7AL_TGACv PTIITLLNAVGTPRSVHLVVFWVLFENVMSLHRAKATFIGLLEAGTVNEWVVTEKLG-----------------DTLKAK 450
TRIAE_CS42_U_TGACv1_ PTIITLLNAVGTPRSVHLVVFWVLFENVMSLHRAKATFIGLLEVGTVNEWVVTEKLG-----------------DTLKAK 444
TRIAE_CS42_U_TGACv1_ PAIITLLSVVGTPRSVHLVIFWALFENVMSLHRTKATFIGLLEAHTVNEWVVTEKLG-----------------DTVKTK 454
TRIAE_CS42_7BL_TGACv -------------------------------------------------------------------------------- 375
```

```
TRIAE_CS42_7AL_TGACv PTVITLLNAVGTPRSFHLVIFWVLFENVMSLHRTKATFSGLLELGRVNEWVVTEKLG-----------------DILKMK 447
TRIAE_CS42_7BL_TGACv PTVITLLNAVGTPRSFHLVIFWVLFENVMSLHRTKATFSGLLELGRVNEWVVTEKLG-----------------DVLKMK 447
TRIAE_CS42_7DL_TGACv PTIITLLNAVGTPRSFHLVIFWVLFENVMSLHRTKATFSGLLELGRVNEWVVTEKLG-----------------DVLKMK 447
TRIAE_CS42_2AL_TGACv PAIITLLNAVCTPRSWHLLVFWILFENVMSMHRSKATIIGLVEASRANEWVVTEKLGSV------------TS-TPAATT 461
TRIAE_CS42_2DL_TGACv PAIITLLNAVCTPRSWHLLVFWILFENVMSMHRSKATIIGLVEASRANEWVVTEKLGSV------------TSSTPAATT 482
TRIAE_CS42_2BL_TGACv PAIITLLNAVCTPRSWHLLVFWILFENVMSMHRSKATIIGLVEASRANEWVVTEKLGSV------------TS-TPAAAT 462
TRIAE_CS42_1AS_TGACv AAVLTLLNAVCTPRSCHLLVFWILFENVMSIHRCKATIIGLLEASRANEWVVTEKLGGS------------TTSTPAAAT 453
TRIAE_CS42_7DS_TGACv PTAITVLYAVRNPSSIHFIPFWILFENVMSFHRTKATFIGLLELGSVNEWVVTEKLG------------------SASNT 477
TRIAE_CS42_7AS_TGACv PTAITILYAVRNPSSIHFIPFWILFENVMSFHRTKATFIGLLELGSVNEWVVTEKLG------------------SVSNT 483
TRIAE_CS42_7BS_TGACv PTAITILYAVRNPSSIHFIPFWILFENVMSFHRTKATFIGLLELGSVNEWVVTEKLG------------------SVSNT 479
TRIAE_CS42_6DS_TGACv PTAITVMNAIRNPGSLHLMPFWILFENVMSMHRMRAALTGLLETAHVNDWVVTEKVG-----------------DLVKDD 461
TRIAE_CS42_6BS_TGACv PTAITIMNAIRNPGSLHLMPFWILFENVMSMHRMRAALTGLLETAHVNDWVVTEKVG-----------------DLVKDD 458
TRIAE_CS42_6AS_TGACv PTAITIMNAIRNPGSLHLMPFWILFENVMSMHRMRAALTGLLETAHVNDWVVTEKVG-----------------DVVKDD 458
TRIAE_CS42_2BS_TGACv PTVLLVVTAIRHPKNLHILPFWILFESVMTMHRMRAALSGLFELSEFNEWVVTKKTG-------------------NNFE 510
TRIAE_CS42_2DS_TGACv PTVLLVVTAIRHPKNLHILPFWILFESVMTMHRMRAALSGLFELSEFNEWVVTKKTG-------------------NNFE 510
TRIAE_CS42_2AS_TGACv PTVLLVVTAIRHPKNLHILPFWILFESVMTMHRMRAALSGLFELSEFNEWVVTKKTG-------------------NNFE 509
TRIAE_CS42_2AS_TGACv PAVLLVVTAIRNPKNIHLLPFWILFESVMTIHRTRAALVGLFEFSEFNEWVVTKKTG-------------------NNFE 508
TRIAE_CS42_2DS_TGACv PTVLLVVTAIRNPKNIHLLPFWILFESVMTIHRTRAALVGLFELTEFDEWLVTKKTG-------------------NNFE 810

TRIAE_CS42_3AS_TGACv TPLVPKLEKRRTRLWDKYNCSEIFVGTCIIICGCYDVLYA-NKGYYIYLFIQGLAFLVIGFEYIGTRPPNTE-------- 566
TRIAE_CS42_3B_TGACv1 TPLVPKLEKR-------YNCSEIFVGTCIIICGCYDVLYA-NKGCYIYLFIQGVAFLVIGFEYIGTRPPGAE-------- 925
TRIAE_CS42_3B_TGACv1 TPLVPKLEKRRTRLWHKYNCSEIFVGTFIIICGCYDVLYA-KKGYYIYLFIQGLAFLVIGFEYIGTRPPSTE-------- 570
TRIAE_CS42_3DS_TGACv TPLVPKLEKRRTRLWDKYNCSEIFVGTCIIICGCYDVLYA-KKGYYIYLFIQGLAFLVIGFEYIGTRPPSIE-------- 568
TRIAE_CS42_3DS_TGACv TPLVPKLEKRRTRLWDKYNCSEIPVGTCIIICGCYDVLYA-KKGYYIYLFIQGLAFLVVGFEYIGTRPPSAE-------- 570
TRIAE_CS42_3DL_TGACv TPLVPKLKKRRIRLWDKYNCSEIFVGTCIVICGFYDLFYA-NKGYYIYLFIQGLAFLVVGFEYIGTRPPTPSA------- 572
TRIAE_CS42_3B_TGACv1 TPLVPKLKKRRIRLWDKYNCSEIFVGTCIIISGFYDLFYA-NKGYYIYLFIQGLAFLVVGFEYIGTRPPTPSAG------ 571
TRIAE_CS42_3AL_TGACv TPLVPKLKKRRIKLWDKYNCSEIFVGTSIIICGFYDLFYA-NKGYYIYLFIQGLAFLVVGFEYIGTRPPTPSAE------ 573
TRIAE_CS42_6BS_TGACv SANKASARKSFMRMWERLNVPELGVGAFLFSCGWYDVAFG-KDNFFIYLFFQSMAFFVVGVGYVGTIVPPS--------- 518
TRIAE_CS42_6AS_TGACv SANKASARKSFMRMWERLNVPELGVGAFLFSCGWYDVAFG-KDNFFIYLFFQSMAFFVVGVGYVGTIVPQS--------- 518
TRIAE_CS42_7AL_TGACv MPSKALK-KLRMRIGERLHLWELGVAAYLFLCGCYDISFG-NNRYFIFLFMQSIAFFIVGVGYVGTFVAQ---------- 518
TRIAE_CS42_U_TGACv1_ MPSKALR-KLRMRIGERLHLWELGVAAYLFLCGCYDISFG-NNRYFIFLFMQSIAFFIVGVGYVGTFVAQ---------- 512
TRIAE_CS42_U_TGACv1_ MPSKALK-KLRIGIGERLHLWELGVAAYLFICGCYSISFG-NNHYFIFLLMQSIAFFIVGVGYVGTFVTQ---------- 522
TRIAE_CS42_7BL_TGACv -------------------------------------------------------------------------------- 375
TRIAE_CS42_7AL_TGACv VQSKVTK-KLRMRIRERLQLLELGVAAYIFFCGSYDLLFG-KRYYYVFLFMQSIAFFVVGVGFVGTLVPN---------- 515
TRIAE_CS42_7BL_TGACv VQSKVTK-KLRMRIRERLQLLELGVAAYIFFCGSYDLLFG-KRYYYIFLFMQSIAFFVVGVGFVGTLVPN---------- 515
TRIAE_CS42_7DL_TGACv VQSKVTK-KLRMRIRERYIIIIGLLMCISQLSYLNFNMES-GCSFWSLVLQPISSFVEVTTFCLAKDITISFSSCNPSLS 525
TRIAE_CS42_2AL_TGACv TMATNKGAMKKKKSQSSILAPEIVMGLCLLYCAVYDIFFG-HDHFYVYLLMQSAAAFVIGFGYVGSQ------------- 527
TRIAE_CS42_2DL_TGACv TMATNKGATKKKKSQSSILAPEIVMGLCLLYCAVYDIVFG-HDHFYVYLLMQSAAAFVIGFGYVGSQ------------- 548
TRIAE_CS42_2BL_TGACv TMAANKGAMKKKKSQSSILAPEIVMGLCLLYCAVYDIVFG-HDHFYVYLLMQSAAAFVIGFGYVGSQ------------- 528
TRIAE_CS42_1AS_TGACv TTMVAK----KKKSSSSFLAPEIVMGLFLLYCALYDIVFG-HDHFYVYLLMQSAAAFVIGFGYVGSQ------------- 515
TRIAE_CS42_7DS_TGACv KPVPQILERPRCRFWDRWTVSELLFAVFLFVCATYNLVYG-SDFYFIYIYLQAITFIIVGTGFCGTSNS----------- 545
TRIAE_CS42_7AS_TGACv KPVPQILERPRCRFWDRWTVSELLFAVFLFVCATYNLVYG-SDFYFIYIYLQAITFIIVGTGFCGTSNS----------- 551
TRIAE_CS42_7BS_TGACv KPVPQILEKPRCRFWDRWTVSELLFAVFLFVCATYNLVYG-SDFYFIYIYLQAITFIIVGTGFCGTSNS----------- 547
TRIAE_CS42_6DS_TGACv FDVPLLEPLKPTECVERIYIPELLLALYLLICASYDYVLG-SQTYFMYIYLQALAFIVLGFGFVGMKTPCS--------- 531
TRIAE_CS42_6BS_TGACv FDVPLLEPLKPTECVERIYIPELLLALYLLICASYDYVLG-SQTYFMYIYLQALAFIVLGFGFVGTKTPCS--------- 528
TRIAE_CS42_6AS_TGACv FEVPLLEPLKPTECVERIYIPELLLALYLLICASYDYVLG-SQTYFMYIYLQALAFIVLGFGFVGMKTPCS--------- 528
TRIAE_CS42_2BS_TGACv DSEVPLLQKTRKRLRDRVNFREIVFSAFLFFCASYNLVFTGKTSYYFNLYLQGLAFVCLGLNFTGTCSCCQ--------- 581
TRIAE_CS42_2DS_TGACv DNEVPLLQKTRKRLRDRVNFREIVFSAFLFFCASYNLVFPGKTSYYFNLYLQGLAFVCLGLNFTGTCSCCQ--------- 581
TRIAE_CS42_2AS_TGACv DNEVPLLQKTRKRLRDRVNFREIVFSAFLFFCASYNLVFPGKTRYYFNLYLQGLAFVCLGLNFTGTCSCCQ--------- 580
TRIAE_CS42_2AS_TGACv DNKVPLLQKTRKRLRDRVNFPEILFSAFLFFCASYNLVFPGKTSYYFNLYLQGLAFAFLGLNFSGTCTCFQ--------- 579
TRIAE_CS42_2DS_TGACv DNKVPLLQKTRKRLRDRVNFPEILFSAFLFFCASYNLVFPGKTSYYFNLYFQGLAFAFLGLNFTGTCTCFQ--------- 881

TRIAE_CS42_3AS_TGACv ------------------------------ 566
TRIAE_CS42_3B_TGACv1 ------------------------------ 925
TRIAE_CS42_3B_TGACv1 ------------------------------ 570
TRIAE_CS42_3DS_TGACv ------------------------------ 568
TRIAE_CS42_3DS_TGACv ------------------------------ 570
TRIAE_CS42_3DL_TGACv ------------------------------ 572
TRIAE_CS42_3B_TGACv1 ------------------------------ 571
TRIAE_CS42_3AL_TGACv ------------------------------ 573
TRIAE_CS42_6BS_TGACv ------------------------------ 518
TRIAE_CS42_6AS_TGACv ------------------------------ 518
TRIAE_CS42_7AL_TGACv ------------------------------ 518
TRIAE_CS42_U_TGACv1_ ------------------------------ 512
TRIAE_CS42_U_TGACv1_ ------------------------------ 522
TRIAE_CS42_7BL_TGACv ------------------------------ 375
TRIAE_CS42_7AL_TGACv ------------------------------ 515
TRIAE_CS42_7BL_TGACv ------------------------------ 515
TRIAE_CS42_7DL_TGACv LSSVWDLLGHLSPTDNALRSWCNVVRKDSV 555
TRIAE_CS42_2AL_TGACv ------------------------------ 527
TRIAE_CS42_2DL_TGACv ------------------------------ 548
TRIAE_CS42_2BL_TGACv ------------------------------ 528
TRIAE_CS42_1AS_TGACv ------------------------------ 515
TRIAE_CS42_7DS_TGACv ------------------------------ 545
TRIAE_CS42_7AS_TGACv ------------------------------ 551
TRIAE_CS42_7BS_TGACv ------------------------------ 547
TRIAE_CS42_6DS_TGACv ------------------------------ 531
TRIAE_CS42_6BS_TGACv ------------------------------ 528
TRIAE_CS42_6AS_TGACv ------------------------------ 528
TRIAE_CS42_2BS_TGACv ------------------------------ 581
TRIAE_CS42_2DS_TGACv ------------------------------ 581
TRIAE_CS42_2AS_TGACv ------------------------------ 580
TRIAE_CS42_2AS_TGACv ------------------------------ 579
TRIAE_CS42_2DS_TGACv ------------------------------ 881
```

Fig. S_2B: *CslC* subfamily.

S.No	Gene name with number of splice variants (CslC)	No. of amino acids (aa)
1	TRIAE_CS42_1DL_TGACv1_062162_AA0209740.1	690 aa
2	TRIAE_CS42_1BL_TGACv1_030501_AA0092480.1	656 aa
3	TRIAE_CS42_5BL_TGACv1_404820_AA1311790.1_3_SPLICE	712 aa
4	TRIAE_CS42_5DL_TGACv1_435778_AA1454840.1_2_SPLICE	708 a
5	TRIAE_CS42_5AL_TGACv1_374268_AA1195590.3_3_SPLICE	703 aa
6	TRIAE_CS42_1DL_TGACv1_061928_AA0205730.1	702 aa
7	TRIAE_CS42_1BL_TGACv1_030750_AA0099830.1	702 aa
8	TRIAE_CS42_1AL_TGACv1_001272_AA0028090.1	702 aa
9	TRIAE_CS42_3DL_TGACv1_251593_AA0882850.1_3_SPLICE	704 aa
10	TRIAE_CS42_3AL_TGACv1_197197_AA0665370.1_3_SPLICE	704 aa
11	TRIAE_CS42_3DS_TGACv1_271926_AA0910940.1	758 aa
12	TRIAE_CS42_3B_TGACv1_220758_AA0718310.2	751 aa
13	TRIAE_CS42_3AS_TGACv1_211225_AA0686890.2	750 aa

```
TRIAE_CS42_1DL_TGACv ---MAPSFWGREAR--LSDGGGGTPVVVKMENPNWSISEMEQEAVPGSPAGLAAGK-------AGRGKNARQITWVLLLK 68
TRIAE_CS42_1BL_TGACv ---MAPSFWGREAR--LSDGGGGTPVVVKMENPNWSISEMEQEAVPGSPAGLAAGK-------AGRGKNARQITWVLLLK 68
TRIAE_CS42_1AL_TGACv ---MAPSFWGREAR--LSDGGGGTPVVVKMENPNWSISEMEQEPVPGSPAGLAAGK-------AGRGKNARQITWVLLLK 68
TRIAE_CS42_3DL_TGACv ----MAPWWGQEARGGVSGGVTGTPVVVKMQTPDWAISEVPPPGSP-----AAGGK-------DGRGKNARQITWVLLLK 64
TRIAE_CS42_3AL_TGACv ----MAPWWGQEARGGVSGGVTGTPVVVKMQTPDWAISEVPPPGSP-----AAGGK-------DGRGKNARQITWVLLLK 64
TRIAE_CS42_1DL_TGACv MAPWNGLWGGRAAIAGGN-AYRDMPVIVKMENPNWSISEINGGGDNGEDFLARVGG------QRRRVKNTKQITWVFRLK 73
TRIAE_CS42_1BL_TGACv -----------------------------MENPNWSISEINIDDDNSEDFLARVGG------QRRRVKNTKQITWVFRLK 45
TRIAE_CS42_5BL_TGACv MAPWTGLWGARAGAGAGAGAYRGTPVVVKMENPNWSISEISPEDAEDEDFLVSGAGAARRSRKGGRGKNAKQITWVLLLK 80
TRIAE_CS42_5DL_TGACv MAPWTGLWGARAGAGAG--AYRGTPVVVKMENPNWSISEISPEDAEDEDFLVSGAGAARR-RKGGRGKNAKQITWVLLLK 77
TRIAE_CS42_5AL_TGACv MAPWTGLWGARAGAGAYR----GTPVVVKMENPNWSISEISPEDAEDEDFLVS----GAARRKGGRGKNAKQITWVLLLK 72
TRIAE_CS42_3DS_TGACv ----------MASSWWGDKEEHGTPVVVKMDNPYSLVEIDGPGMDSSEK--------------ARRSKNAKQFKWVLLLR 56
TRIAE_CS42_3B_TGACv1 ----------MASSWWGDKEEHGTPVVVKMDNPYSLVEIDGPGMDSSEK--------------ARRSKNAKQFKWVLLLR 56
TRIAE_CS42_3AS_TGACv ----------MASSWWGDKEEHGTPVVVKMDNPYSLVEIDGPGMDSSEK--------------ARRSKNAKQFKWVLLLR 56

TRIAE_CS42_1DL_TGACv AHRAAGRLTGAASAALAVAAAARRRVAAGRTDGDAAPG--------ESTALRARFYGCLRLFVVLSMLLLAVEVAAYLQG 140
TRIAE_CS42_1BL_TGACv AHRAAGRLTGAASAALAVAAAARRRVAAGRTDGDAAPG--------ESTALRARFYGCLRLFVVLSMLLLAVEVAAYLQG 140
TRIAE_CS42_1AL_TGACv AHRAAGRLTGAASAALAVAAAARRRVAAGRTDGDAAPG--------ESTALRARFYGCLRVFVVLSMLLLAVEVAAYLQG 140
TRIAE_CS42_3DL_TGACv AHRAAGKLTGAATAALSVAAAARRRVAAGRTDSDADNAPPGLG---GSPALRTRLYGFIRASLLLSVLLLAADVAAHAQG 141
TRIAE_CS42_3AL_TGACv AHRAAGKLTGAATAALSVAAAARRRVAAGRTDSDADADGAPPGPGAGRPALRTRLYGFIRASLLLSLLLLAADVAAHAQG 144
TRIAE_CS42_1DL_TGACv AHRAAGCLARLTSAAVALGGAARRRVVAGRTDSDAADGECEDVEERDPASRRSRFYTLIKACLMMSVFLLVVELAAYSN- 152
TRIAE_CS42_1BL_TGACv AHRAAGCLSWLTSAAFALGGATRRRVVAGRTDSNATDGECKDVEEWAPASRRSRFYTLIKACLMMFVCLLIVELAAYSN- 124
TRIAE_CS42_5BL_TGACv AHRAAGCLASLASAAVTLGAAARRRVADGRTDADAGAPG-SAGES---PVLRSRFYAFIRAFLLLSLLLLAVELAARLHG 156
TRIAE_CS42_5DL_TGACv AHRAAGCLASLASAAVTLGAAARRRVADGRTDADAGATPGSAGES---PVLRSRFYAFIRAFLLLSLLLLAVELAARFHG 154
TRIAE_CS42_5AL_TGACv AHRAAGCLASLASAAVTLGAAARRRVADGRTDADAGAPG-PARES---PVLRSRFYAFIRAFLLLSLLLLAVELAARFHR 148
TRIAE_CS42_3DS_TGACv AHRAVGCVAWLAGGFWGLLGAVNRRVRRSRDADAEPDAEASGRGR--------HMLGFLRAFLLLSLAMLAFETAAYLKG 128
TRIAE_CS42_3B_TGACv1 AHRAVGCVAWLAGGFWGLLGAVNRRVRRSRDADAEPDAEASGRGR--------HMLGFLRAFLLLSLAMLAFETAAYLKG 128
TRIAE_CS42_3AS_TGACv AHRAVGCVAWLAGGFWGLLGAVNRRVRRSRDADAEPDAEASGRGR--------HMLGFLRAFLLLSLAMLAFETAAYLKG 128

TRIAE_CS42_1DL_TGACv W---------------------------------HLQMPEMPEMPGQLAMDGLLAVDGLAASAYAGWMRVRLQYIAPPLQ 187
TRIAE_CS42_1BL_TGACv W---------------------------------HLQMPEMPEMPGQLAMDGLLAVDGLAAAAYAGWMRVRLQYIAPPLQ 187
TRIAE_CS42_1AL_TGACv W---------------------------------HLQMPQMPEMPGQLAMDGLLAVDGLAAAAYAGWMRVRLQYIAPPLQ 187
TRIAE_CS42_3DL_TGACv W---------------------------------HLAA-----------LPDLEAVEGLFAAGYAAWMRARAAYLGPALQ 177
TRIAE_CS42_3AL_TGACv W---------------------------------HLAA-----------LPDLEAVEGLFAAGYAAWMRARAAYLGPALQ 180
TRIAE_CS42_1DL_TGACv ---------------------------------------------------GRVNLAIFINSFNTSWIRFRATYVAPPLQ 181
TRIAE_CS42_1BL_TGACv ---------------------------------------------------GKGNLAVFINSFNTSWIRFRAAYIAPPLQ 153
TRIAE_CS42_5BL_TGACv WDLAA---------------------------------------------SALALPIIGVESLYASWLRLRAAYLAPLLQ 191
TRIAE_CS42_5DL_TGACv WDLAA---------------------------------------------SALALPIIGVESLYASWLRLRAAYLAPLLQ 189
TRIAE_CS42_5AL_TGACv WDLAA---------------------------------------------SALALPIIGVESLYASWLRLRAAYLAPLLQ 183
TRIAE_CS42_3DS_TGACv WHYFPRDLPEHYLRQLPEHLQNLPEHLRHLPENLRHLPENLRHLPDGLRMPEQQEIQGWLHRAYVAWLAFRIDYIAWAIE 208
TRIAE_CS42_3B_TGACv1 WHYFPRDLPEHYLRQLPEHLQ-------NLPENLRHLPENLRHLPDGLRMPEQQEIQGWLHRAYVAWLAFRIDYIAWAIE 201
TRIAE_CS42_3AS_TGACv WHYFPRDLPEHYLRQLPEHLQN-------LPEHLRHLPENLRHLPDGLRMPEQQEIQGWLHRAYVAWLAFRIDYIAWAIE 201

TRIAE_CS42_1DL_TGACv FLTNSCVVLFMIQSVDRLVLCLGCLWIKLRGIKP---VPIAADKD--------------DVEAGDEDFPMVLVQMPMCNE 250
TRIAE_CS42_1BL_TGACv FLTNSCVVLFMIQSVDRLILCLGCLWIKLRGIKP---VPIAADKD--------------DVEAGEEDFPMVLVQMPMCNE 250
TRIAE_CS42_1AL_TGACv FLTNSCVVLFMIQSVDRLVLCLGCLWIKLRGIKP---VPIAADKD--------------DVEAGDEDFPMVLVQMPMCNE 250
TRIAE_CS42_3DL_TGACv FLTNACVVLFMIQSADRLILCLGCFWIKLRGIRP---VPNAAAAAGNGNGKGSDDVEAGAQEE--GDFPMVLVQIPMCNE 252
TRIAE_CS42_3AL_TGACv FLTNACVVLFMIQSADRLILCLGCFWIKLRGIRP---VPNAATAG---NGKGSDDVEAGAQEEEEGEFPMVLVQIPMCNE 254
```

```
TRIAE_CS42_1DL_TGACv LLADACVVLFLVQSADRLFQSLGCFYILVKRIKPKPLSPALA----------------DAEDPDAGYYPMVLVQIPMCNE 245
TRIAE_CS42_1BL_TGACv LLANACVVLFLVQSADRVFQSLGCFYILVKRIKPKPLFLALS----------------DAEDPDAGYYPMVLVQIPMCNE 217
TRIAE_CS42_5BL_TGACv FLTDACVVLFLIQSADRLIQCLGSFYITVKRIKPTLKSPALP----------------DAEDPDAGYYPMVLVQIPMCNE 255
TRIAE_CS42_5DL_TGACv FLTDACVVLFLIQSADRLIQCLGSFYITVKRIKPRLKSPALP----------------DAEDPDAGYYPMVLVQIPMCNE 253
TRIAE_CS42_5AL_TGACv FLTDACVVLFLIQSADRLIQCLGSFYITVKRIKPRLRSPALP----------------DAEDPDAGYYPMVLVQIPMCNE 247
TRIAE_CS42_3DS_TGACv KLSGFCIVLFMVQSIDRILLCLGCFWIKLRGIKPGLKAAANKRGSK-----YADDDDLEDGDDLGAYFPMVLLQMPMCNE 283
TRIAE_CS42_3B_TGACv1 KLSGFCIVLFMVQSIDRILLCLGCFWIKLRGIKPGLKAAASKRGSK-----YADENDLEDGDDLGAYFPMVLLQMPMCNE 276
TRIAE_CS42_3AS_TGACv KLSGFCIVLFMVQSIDRILLCLGCFWIKVRGIKPGLVATK-KRGNK-----YADDNDLEDGDDLGAYFPMVLLQMPMCNE 275

TRIAE_CS42_1DL_TGACv REVYQQSIGAICALDWPRSNFLVQVLDDSDDATTSALIKEEVEKWQREGVRIVYRHRVIRDGYKAGNLKSAMNCSYVKDY 330
TRIAE_CS42_1BL_TGACv REVYQQSIGAICALDWPRSNFLVQVLDDSDDATTSALIKEEVEKWQREGVRIVYRHRVIRDGYKAGNLKSAMNCSYVKDY 330
TRIAE_CS42_1AL_TGACv REVYQQSIGAICALDWPRSNFLVQVLDDSDDATTSALIKEEVEKWQREGVRIVYRHRVIRDGYKAGNLKSAMNCSYVKDY 330
TRIAE_CS42_3DL_TGACv KEVYQQSIGAVCNLDWPRSNFLVQVLDDSDDAATSALIREEVEKWQREGVRVLYRHRVIRDGYKAGNLKSAMNCSYVKDY 332
TRIAE_CS42_3AL_TGACv KEVYQQSIGAVCNLDWPRSNFLVQVLDDSDDAATSALIREEVEKWQREGVRIIYRHRVIRDGYKAGNLKSAMNCSYVKDY 334
TRIAE_CS42_1DL_TGACv KEVYRQSIAAVCNLDWPRSNFLVQVLDDSDDVATQALIKEEVEKWRHSGAHIVYRHRVLREGYKAGNLKSAMSCSYVKDY 325
TRIAE_CS42_1BL_TGACv KEVYRQSIAAVCNLDWPRSNFLVQVLDDSDDVTTQALIKDEVEKWRHSGAHIVYRHRVLREGYKAGNLKSAMSCSYVKDY 297
TRIAE_CS42_5BL_TGACv KEVYQQSIAAVCNLDWPRSNFLVQVLDDSDDPTTQSLIREEVAKWQQTGARIIYRHRVLRDGYKAGNLKSAMACSYVKDY 335
TRIAE_CS42_5DL_TGACv KEVYQQSIAAVCNLDWPRSNFLVQVLDDSDDPTTQSLIREEVAKWQQTGARIIYRHRVLRDGYKAGNLKSAMACSYVKDY 333
TRIAE_CS42_5AL_TGACv KEVYQQSIAAVCNLDWPRSNFLVQVLDDSDDPTTQSLIREEVAKWQQTGARIIYRHRVLRDGYKAGNLKSAMACSYVKDY 327
TRIAE_CS42_3DS_TGACv KEVYETSISHVCQIDWPRDRMLVQVLDDSDDETCQMLIRAEVTKWNQRGVNIIYRHRLSRTGYKAGNLKSAMSCEYVKDY 363
TRIAE_CS42_3B_TGACv1 KEVYETSISHVCQIDWPRDRMLVQVLDDSDDETCQMLIRAEVTKWSQRGVNIIYRHRLSRTGYKAGNLKSAMSCEYVKDY 356
TRIAE_CS42_3AS_TGACv KEVYETSISHVCQIDWPRDRMLVQVLDDSDDETCQMLIRAEVTKWNQRGVNIIYRHRLSRTGYKAGNLKSAMSCEYVKDY 355

TRIAE_CS42_1DL_TGACv EYVVIFDADFQPQADFLKRAMPHFKGKDDVGLVQARWSFVNNDENLLTRLQNINLCFHFEVEQQVNGAFLNFFGFNGTAG 410
TRIAE_CS42_1BL_TGACv EYVVIFDADFQPQADFLKRAMPHFKGKDDVGLVQARWSFVNNDENLLTRLQNINLCFHFEVEQQVNGAFLNFFGFNGTAG 410
TRIAE_CS42_1AL_TGACv EYVVIFDADFQPQADFLKRAMPHFKGKDDVGLVQARWSFVNNDENLLTRLQNINLCFHFEVEQQVNGAFLNFFGFNGTAG 410
TRIAE_CS42_3DL_TGACv EFVVIFDADFQPQPDFLKLTVPHFKGKEDVGLVQARWSFVNEDENLLTRLQNINLCFHFEVEQQVNGAFLNFFGFNGTAG 412
TRIAE_CS42_3AL_TGACv EFVVIFDADFQPQPDFLKLTVPHFKGKEDVGLVQARWSFVNEDENLLTRLQNINLCFHFEVEQQVNGAFLNFFGFNGTAG 414
TRIAE_CS42_1DL_TGACv EYVAIFDADFQPYPDFLKRTVPHFKDNEDLGLVQARWSFVNEDENLLTRLQNINLCFHFEVEQQVNGVFINFFGFNGTAG 405
TRIAE_CS42_1BL_TGACv EYVAIFDADFQPYPDFLKRTVPHFKDNEDLGLVQARWSFVNEDENLLTRLQNINLCFHFEVEQQVNGVFINFFGFNGTAG 377
TRIAE_CS42_5BL_TGACv EFVAIFDADFQPNPDFLKRTVPHFKDNDELGLVQARWSFVNEDENLLTRLQNINLCFHFEVEQQVNGVFLNFFGFNGTAG 415
TRIAE_CS42_5DL_TGACv EFVAIFDADFQPNPDFLKRTVPHFKDNDELGLVQARWSFVNEDENLLTRLQNINLCFHFEVEQQVNGVFLNFFGFNGTAG 413
TRIAE_CS42_5AL_TGACv EFVAIFDADFQPNPDFLKRTVPHFKDNDELGLVQARWSFVNEDENLLTRLQNINLCFHFEVEQQVNGVFLNFFGFNGTAG 407
TRIAE_CS42_3DS_TGACv EFVAIFDADFQPNPDFLKLTVPHFKGNPELGLVQARWSFVNEDENLLTRLQNINLCFHFEVEQQVNGIYLNFFGFNGTAG 443
TRIAE_CS42_3B_TGACv1 EFVAIFDADFQPNPDFLKLTVPHFKGNPELGLVQARWSFVNEDENLLTRLQNINLCFHFEVEQQVNGIYLNFFGFNGTAG 436
TRIAE_CS42_3AS_TGACv EFVAIFDADFQPNPDFLKLTVPHFKGNPELGLVQARWSFVNEDENLLTRLQNINLCFHFEVEQQVNGIYLNFFGFNGTAG 435

TRIAE_CS42_1DL_TGACv VWRIKALEDSGGWMERTTVEDMDIAVRAHLKGWKFLYLNDVECQCELPESYEAYRKQQHRWHSGPMQLFRLCFVDIIKSK 490
TRIAE_CS42_1BL_TGACv VWRIKALEDSGGWMERTTVEDMDIAVRAHLKGWKFLYLNDVECQCELPESYEAYRKQQHRWHSGPMQLFRLCFVDIIKSK 490
TRIAE_CS42_1AL_TGACv VWRIKALEDSGGWMERTTVEDMDIAVRAHLKGWKFLYLNDVECQCELPESYEAYRKQQHRWHSGPMQLFRLCFVDIIKSK 490
TRIAE_CS42_3DL_TGACv VWRIKALEDSGGWMERTTVEDMDIAVRAHLKGWKFLYLNDVECQCELPESYEAYRKQQHRWHSGPMQLFRLCFVDIIKSK 492
TRIAE_CS42_3AL_TGACv VWRIKALEDSGGWMERTTVEDMDIAVRAHLKGWKFLYLNDVECQCELPESYEAYRKQQHRWHSGPMQLFRLCFVDIIKSK 494
TRIAE_CS42_1DL_TGACv VWRIKAVEDSGGWMERTTVEDMDIAVRAHLKGWKFVFLNDVECQCELPETYEAYRKQQHRWHSGPMQLFRLCLPDIIRCK 485
TRIAE_CS42_1BL_TGACv VWRIKAVEDSGGWMERTTVEDMDIAG------WKFVFLNDVECQCELPETYEAYRKQQHRWHSGPMQLFRLCLPDIIRCK 451
TRIAE_CS42_5BL_TGACv VWRIKALEESGGWMERTTVEDMDIAVRAHLHGWKFIFLNDVECQCELPESYEAYRKQQHRWHSGPMQLFRLCIPDIIKSK 495
TRIAE_CS42_5DL_TGACv VWRIKALEESGGWMERTTVEDMDIAVRAHLHGWKFIFLNDVECQCELPESYEAYRKQQHRWHSGPMQLFRLCIPDIIKSK 493
TRIAE_CS42_5AL_TGACv VWRIKALEESGGWMERTTVEDMDIAVRAHLHGWKFIFLNDVECQCELPESYEAYRKQQHRWHSGPMQLFRLCIPDIIKSK 487
TRIAE_CS42_3DS_TGACv VWRIEALEDSGGWMERTTVEDMDIAVRAHLQGWKFIYLNDVKVLCELPESYQAYRKQQHRWHSGPMQLFRLCLPAIIKSK 523
TRIAE_CS42_3B_TGACv1 VWRIEALEDSGGWMERTTVEDMDIAVRAHLQGWKFIYLNDVKVLCELPESYQAYRKQQHRWHSGPMQLFRLCLPAIIKSK 516
TRIAE_CS42_3AS_TGACv VWRIEALEDSGGWMERTTVEDMDIAVRAHLQGWKFIYLNDVKVLCELPESYQAYRKQQHRWHSGPMQLFRLCLPAIIKSK 515

TRIAE_CS42_1DL_TGACv IGFWKKCNLIFLFFLLRKLILPFYSFTLFCVILPMTMFVPEAELPAWVVCYIPATMSIMSILPSPKSFPFIVPYLLFENT 570
TRIAE_CS42_1BL_TGACv IGFWKKCNLIFLFFLLRKLILPFYSFTLFCVILPMTMFVPEAELPAWVVCYIPATMSIMSILPSPKSFPFIVPYLLFENT 570
TRIAE_CS42_1AL_TGACv IGFWKKCNLIFLFFLLRKLILPFYSFTLFCVILPMTMFVPEAELPAWVVCYIPATMSIMSILPSPKSFPFIVPYLLFENT 570
TRIAE_CS42_3DL_TGACv IGFWKKFNLIFLFFLLRKLILPFYSFTLFCVILPMTMFAPEAELPAWVVCYIPATMSLLNILPAPKSFPFIVPYLLFENT 572
TRIAE_CS42_3AL_TGACv IGFWKKFNLIFLFFLLRKLILPFYSFTLFCVILPMTMFAPEAELPAWVVCYIPATMSLLNILPAPKSFPFIVPYLLFENT 574
TRIAE_CS42_1DL_TGACv IVFWKKANLIFLFFLLRKLILPFYSFTLFCIILPMTMFVPEAELPDWVVCYIPVLMSFLNIAPAPKSFPFIIPYLLFENT 565
TRIAE_CS42_1BL_TGACv IVFWKKANLIFLFFLLRKLILPFYSFTLFCIILPMTMFVPEAELPDWVVCYIPVLMSFLNIAPAPKSFPFIIPYLLFENT 531
TRIAE_CS42_5BL_TGACv ISVWKKFNLIFLFFLLRKLILPFYSFTLFCIILPMTMFVPEAELPDWVVCYIPALMSLLNILPSPKSFPFIIPHLLFENT 575
TRIAE_CS42_5DL_TGACv ISVWKKFNLIFLFFLLRKLILPFYSFTLFCIILPMTMFVPEAELPDWVVCYIPALMSLLNILPSPKSFPFIIPYLLFENT 573
TRIAE_CS42_5AL_TGACv ISVWKKFNLIFLFFLLRKLILPFYSFTLFCIILPMTMFVPEAELPDWVVCYIPALMSLLNILPSPKSVPFIIPYLLFENT 567
TRIAE_CS42_3DS_TGACv IPLWKKANLVMLFFLLRKLILPFYSFTLFCVILPLTMFVPEAELPIWVICYVPMIMSVLNILPAPKSFPFVIPYLLFENT 603
TRIAE_CS42_3B_TGACv1 IPLWKKANLVMLFFLLRKLILPFYSFTLFCVILPLTMFVPEAELPIWVICYVPMIMSVLNILPAPKSFPFVIPYLLFENT 596
TRIAE_CS42_3AS_TGACv IPLWKKANLVMLFFLLRKLILPFYSFTLFCVILPLTMFVPEAELPIWVICYVPMIMSVLNILPAPKSFPFVIPYLLFENT 595

TRIAE_CS42_1DL_TGACv MSVTKFNAMISGLFQLGSAYEWVVTKKSGRSSEGDLVALVEKHTVQQQQRVG-----------------------SAPDL 627
TRIAE_CS42_1BL_TGACv MSVTKFNAMISGLFQLGSAYEWVVTKKSGRSSEGDLVALVEKHTVQQQQRVG-----------------------SAPDL 627
TRIAE_CS42_1AL_TGACv MSVTKFNAMISGLFQLGSAYEWVVTKKSGRSSEGDLVALVEKHTAQQQQRVG-----------------------SAPDL 627
TRIAE_CS42_3DL_TGACv MSVTKFNAMISGLFQLGSAYEWVVTKKSGRSSEGDLVALVENEKQSKQLRVG-----------------------SAPNL 629
TRIAE_CS42_3AL_TGACv MSVTKFNAMISGLFQLGSAYEWVVTKKSGRSSEGDLVALVENEKQSKQQRVG-----------------------SAPNL 631
TRIAE_CS42_1DL_TGACv MSVTKFNAMISGLFQLGSTYEWVVTKKSGRSLEGDLISLAPKGLKQLKYG---------------------------- 615
TRIAE_CS42_1BL_TGACv MSVTKFNAMISGLFQLGSTYEWVVTKKSGRSLEGDLISLAPKGLKQLKYG---------------------------- 581
TRIAE_CS42_5BL_TGACv MSVTKFNAMISGLFQLGSAYEWVVTKKSGRSSEGDLISLAAAAPPRELRHHPKTGS------------------APSLEA 637
TRIAE_CS42_5DL_TGACv MSVTKFNAMISGLFQLGSAYEWVVTKKSGRSSEGDLISLAAAPP-RELRHHPKTGS------------------APSMEA 634
TRIAE_CS42_5AL_TGACv MSVTKFNAMISGLFQLGSAYEWVVTKKSGRSSEGDLISLAAAAPPRELRQQQKTGS------------------APSLEA 629
TRIAE_CS42_3DS_TGACv MSVTKFNAMVSGLFQLGSSYEWVVTKKAGRTSSESDIFAMAEETDTATRPAPRLVRGVSEAGLEAWAKTHQLDNKDLQLK 683
TRIAE_CS42_3B_TGACv1 MSVTKFNAMVSGLFQLGSSYEWVVTKKAGRTSSESDIFAMAEETNTATRPAPRLVRGVSEAGLEAWAKTHQLDNKDLQLK 676
TRIAE_CS42_3AS_TGACv MSVTKFNAMVSGLFQLGSSYEWVVTKKAGRTSSESDIFAMAEKTDTATRPAPRLVRGVSEAGLEAWAKTHQLDNKDLQLK 675

TRIAE_CS42_1DL_TGACv AGLAAKDSSLPKKDAPKKKQKHNRIYRKELALSFLLLTAAARSVLSAQGIHFYFLLFQGVSFLVMGLDLIGEQVE 702
TRIAE_CS42_1BL_TGACv AGLAAKDSSLPKKDAPKKKQKHNRIYRKELALSFLLLTAAARSVLSAQGIHFYFLLFQGVSFLVMGLDLIGEQVE 702
```

```
TRIAE_CS42_1AL_TGACv AGLAAKDSSLPKKDAPKKKQKHNRIYRKELALSFLLLTAAARSVLSAQGIHFYFLLFQGVSFLVMGLDLIGEQVE 702
TRIAE_CS42_3DL_TGACv DSLAAKEELYPKAEPKPKKKKHNRLYRKELALSFLLLTAAARSLLSVQGIHFYFLLFQGVSFLVVGLDLIGEQVE 704
TRIAE_CS42_3AL_TGACv DSLAAKEELYPKSEP--KKKKHNRLYRKELALSFLLLTAAARSLLSVQGIHFYFLLFQGVSFLVVGLDLIGEQVE 704
TRIAE_CS42_1DL_TGACv SVPAINVAIKEQSKAKKESKKYNRIYKKELAMSLLLLSAAARSLLSKQGIHFYFLLFQGISFLLVGLDLIGQDIK 690
TRIAE_CS42_1BL_TGACv SVPAINVAIKEKLKAKKESKKYNRIYKKELAMSLLLLSAAIRSLLSKQGIHFYFLLFQGISFLLVGLDLIGQDIK 656
TRIAE_CS42_5BL_TGACv LMVLKEQQPSPKKEGKKQQKKHNRIYKKELALSLLLLTAAARSLLTKQGIHFYFLLFQGISFLLVGLDLIGEQVE 712
TRIAE_CS42_5DL_TGACv LMVLKEQ-PSPKKEGKKQQKKHNRIYKKELALSLLLLTAAARSLLTKQGIHFYFLLFQGISFLLVGLDLIGEQVE 708
TRIAE_CS42_5AL_TGACv LMVLKEEQASPRKEGKKQ-KKHNRIYKKELALSLLLLTAAARSLLTKQGIHFYFLLFQGISFLLVGLDLIGEQVE 703
TRIAE_CS42_3DS_TGACv AQAEEVTSLAAAIKKTSKAKPPNRIFKKELALAFLLLIAATRSLLSAQGLHFYFLLFQGVTFLVVGLDLIGEQVS 758
TRIAE_CS42_3B_TGACv1 AEAEEVTSLAAAIKKTSKAKPPNRIFKKELALAFLLLIAATRSLLSAQGLHFYFLLFQGVTFLVVGLDLIGEQVS 751
TRIAE_CS42_3AS_TGACv AEAEEVTSLAAAIKKTSKAKPPNRIFKKELALAFLLLIAATRSLLSAQGLHFYFLLFQGVTFLVVGLDLIGEQVS 750
```

Fig. S_2C: *CslD* subfamily.

S.No	Gene name with number of splice variants (CslD)	No. of amino acids (aa)
1	TRIAE_CS42_2BS_TGACv1_148683_AA0494520.1	1121 aa
2	TRIAE_CS42_2DS_TGACv1_177279_AA0572180.1	1120 aa
3	TRIAE_CS42_2AS_TGACv1_114244_AA0365360.1	1120 aa
4	TRIAE_CS42_1BL_TGACv1_030586_AA0094860.1	1189 aa
5	TRIAE_CS42_1AL_TGACv1_001700_AA0034150.2	1146 aa
6	TRIAE_CS42_1DL_TGACv1_063091_AA0223780.1	1014 aa
7	TRIAE_CS42_1BS_TGACv1_049706_AA0160220.1	330 aa
8	TRIAE_CS42_5BS_TGACv1_425241_AA1392650.1	1022 aa
9	TRIAE_CS42_5DS_TGACv1_457675_AA1488780.1	989 aa
10	TRIAE_CS42_7BL_TGACv1_577301_AA1871610.1	994 aa
11	TRIAE_CS42_7AL_TGACv1_559436_AA1799630.1	993 aa
12	TRIAE_CS42_7DL_TGACv1_603510_AA1985050.1	994 aa

```
TRIAE_CS42_1BL_TGACv --------------------MGSKGILKNSGSSRMPPHGPSKPPTAPTSAPQVVFGRRTESGRFISYSRDDLDS-EISSV 59
TRIAE_CS42_1DL_TGACv --------------------MGSKGILKNSGSSRVPPHGPSKPPTAPTSAPQVVFGRRTESGRFISYSRDDLDS-EISSV 59
TRIAE_CS42_1AL_TGACv --------------------MGSKGILKNSGSSRVPPHGPSKPPTAPTSAPQVVFGRRTESGRFISYSRDDLDS-EISSV 59
TRIAE_CS42_2DS_TGACv -----------------------------MSKAPRNPGGGSAGAPKSSSGQPVKFARRTPSGRYLSLSREDIDMEGEMGP 51
TRIAE_CS42_2AS_TGACv -----------------------------MSKAPRNPGGGSAGAPKSSSGQPVKFARRTPSGRYLSLSREDIDMEGEMGP 51
TRIAE_CS42_2BS_TGACv -----------------------------MSKAPRNPGGGSAGAPKSSSGQPVKFARRTPSGRYLSLSREDIDMEGEMGP 51
TRIAE_CS42_7BL_TGACv -----------------------------------------------------------------------------MAS 3
TRIAE_CS42_7DL_TGACv -----------------------------------------------------------------------------MAS 3
TRIAE_CS42_7AL_TGACv -----------------------------------------------------------------------------MAS 3
TRIAE_CS42_1BS_TGACv -------------------------------------------------------------------------------- 0
TRIAE_CS42_5BS_TGACv MSRRLSLPAGSPVTVTVSPTKGKGAGGGSPGDGVVRRGSGLTSPVPRHSIGSSTATLQVSPVRRSGGSRYASRDGADASA 80
TRIAE_CS42_5DS_TGACv MSRRLSLPASSPVTVTVSPTRGKGAGGGSPGDGVVRRGSGLTSPVPRHSIGSSTATLQVSPVRRSGGSRYASRDGADASA 80

TRIAE_CS42_1BL_TGACv DFQDYHVHIPMTPDNQPMEED------GTKADEQYVSSSLFTGGFNSVTRAHVMD--KQGPDSDIGRSGPKGSICMVEGC 131
TRIAE_CS42_1DL_TGACv DFQDYHVHIPMTPDNQPMEED------GTKADEQYVSSSLFTGGFNSVTRAHVMD--KQGPDSDMGRSGPKGSICMVEGC 131
TRIAE_CS42_1AL_TGACv DFQDYHVHIPMTPDNQPMEED------GTKADEQYVSSSLFTGGFNSVTRAHVMD--KQGPDSDMGRSGPKGSICMVEGC 131
TRIAE_CS42_2DS_TGACv DYANYTVHIPPTPDNQPMKDGAERTAVAMKAEEQYVSNSLFTGGFNSVTRAHLMDRVIDSDVKHPQMAGARPARCAMPAC 131
TRIAE_CS42_2AS_TGACv DYANYTVHIPPTPDNQPMKDGAEPTAVAMKAEEQYVSNSLFTGGFNSVTRAHLMDRVIDSDVKHPQMAGAKATRCAMPAC 131
TRIAE_CS42_2BS_TGACv DYANYTVHIPPTPDNQPMKDGSEPTAVAMKAEEQYVSNSLFTGGFNSVTRAHLMDRVIDSDVKHPQMAGAKATRCAMPAC 131
TRIAE_CS42_7BL_TGACv DHTNYTVFMPPTPDNQPGAAPAPASGGSTKPDNLPLP--RYTSGSKLVNRRSGDDGAAGGAKMDRGLS------------ 69
TRIAE_CS42_7DL_TGACv DHTNYTVFMPPTPDNQPGAAPTPASGGSTKPENLPLP--RYTSGSKLVNRRSGDDGAAGGAKMDRWLS------------ 69
TRIAE_CS42_7AL_TGACv DHTNYTVFMPPTPDNQPGAASAPASGGPTKPDNLPLP--RSS-GSKLVNRRSGDDGAAGGGKMDRRLS------------ 68
TRIAE_CS42_1BS_TGACv ------------------------------------------------------------------------MSCKMRGC 8
TRIAE_CS42_5BS_TGACv EFVHYTVHIPPTPDRTTASASTDVPAAEEEGEVLPQRSYVSGTIFTGGLNCATRAHVLSNSADGARPAASANMSCKMRGC 160
TRIAE_CS42_5DS_TGACv EFVHYTVHIPPTPDRNTASASTDAPVAEEEGEVLPQRSYVSGTIFTGGLNCTTRAHVLSNSADGARPAASVNMSCKMRGC 160

TRIAE_CS42_1BL_TGACv DSKIMRNGRGEDILPCECDFKICVDCFTDAVKGGGGVCPGCKELYKHTEWEEVLSNSSNELTRALSLPHGPGGKMERRLS 211
TRIAE_CS42_1DL_TGACv DSKIMRNGRGEDILPCECDFKICVDCFTDAVKGGRGVCPGCKELYKHTEWEEVLSNSSNELTRALSLPHGPGGKMERRLS 211
TRIAE_CS42_1AL_TGACv DSKIMRNGRGEDILPCECDFKICVDCFTDAVKGGGGVCPGCKELYKHTEWEEVLSNSSNELTRALSLPHGPGGKMERRLS 211
TRIAE_CS42_2DS_TGACv DGKVMRNERGEEIEPCECRFKICRDCYLDAQKDGCLCPG----------------------CKEHYKIGDYADDDTHDVS 189
TRIAE_CS42_2AS_TGACv DGKVMRNERGEEIDPCECRFKICRDCYLDAQKDGCLCPG----------------------CKEHYKIGDYADDDPHDVS 189
TRIAE_CS42_2BS_TGACv DGKVMRNERGEEVDPCECRFKICRDCYLDAQKDGCLCPG----------------------CKEHYKIGDYADDDPHDVS 189
TRIAE_CS42_7BL_TGACv --------------------------------------------------------------------------TEHVAS 75
TRIAE_CS42_7DL_TGACv --------------------------------------------------------------------------TEQVAS 75
TRIAE_CS42_7AL_TGACv --------------------------------------------------------------------------PVQVAS 74
TRIAE_CS42_1BS_TGACv DMLALAATRP----------MICEECYMDCVAASGNCPGCKEAYSAGSDTDDSVDEDDDDAISSSEERDQMPMTSMSKRF 78
TRIAE_CS42_5BS_TGACv DMPAFLNAGRGGHPPCDCGFMICEECYMDCVAAAGNCPGCKEAYSAGSDTDDSVDEDDDDAISSSEERDQMPMTSMSKRF 240
TRIAE_CS42_5DS_TGACv DMPAFLNAGRGGRPPCDCGFMICEECYMDCVAAAGNCPGCKEAYSAGSDTDDSVDEDDDDAISSSEERDQMPMTSMSKRF 240

TRIAE_CS42_1BL_TGACv LVKQGTMNNQS----------GEFDHNRWLFETKGTYGYGNAIWPD-------DNVDDDGRNGVPGHPKELMSKPWRPLT 274
TRIAE_CS42_1DL_TGACv LVKQGTMNNQS----------GEFDHNRWLFETKGTYGYGNAIWPD-------DNVDDDGRNGVPGHPKELMSKPWRPLT 274
TRIAE_CS42_1AL_TGACv LVKQGTMNNQS----------GEFDHNRWLFETKGTYGYGNAIWPD-------DNVDDDGRNGVPGHPKELMSKPWRPLT 274
TRIAE_CS42_2DS_TGACv AGKSLLARNQN----------GEFDHNRWLFESSGTYGYGNAFMPKG--GMYEDDLDEDGAAGDD-GMQDMNQKPFKPLT 256
TRIAE_CS42_2AS_TGACv SGKSLLARNQN----------GEFDHNRWLFESSGTYGYGNAFMPKG--GMYEDDLDEDGVGGDG-GMQDMNQKPFKPLT 256
TRIAE_CS42_2BS_TGACv AGKSLLARNQN----------GEFDHNRWLFESSGTYGYGNAFMPKG--GMYEDDLDEDGAGGDGGMPADLSQKPFKPLT 257
TRIAE_CS42_7BL_TGACv PSKSLLVRSQT----------GEFDHNRWLFETQGTYGIGNAYWPQ------DENDDGAGMGGGSVKMEDLVDKPWKPLS 139
TRIAE_CS42_7DL_TGACv PSKSLLVRSQT----------GEFDHNRWLFETQGTYGIGNAYWPQ------DDNDDGAGMGGGSVKMEDLVDKPWKPLS 139
TRIAE_CS42_7AL_TGACv PSKSLLVRSQT----------GEFDHNRWLFETQGTYGIGNAYWPQ------EDNDDGAGMGGGSVKMEDLVDKPWKPLS 138
TRIAE_CS42_1BS_TGACv SMVHSIKMPMSSSND----KPADFDHARWLFETKGTYSYGNALWPENEHGGGGNNAGATFGFVGIEEPPNF--------- 145
TRIAE_CS42_5BS_TGACv SMVHSIKMPMPSSNG----KPADFDHARWLFETKGTYGYGNALWPKNEHGGGGNNAGATSGFVGIEEPPNFGARCRRPLT 316
TRIAE_CS42_5DS_TGACv SMVHSIKMPMPSSNGNGGGKPADFDHARWLFETKGTYGYGNALWPKNEHGGGGNTAGATSGFVGIEEPPNFGARCRRPLT 320

TRIAE_CS42_1BL_TGACv RKLQIPAAVISPYRLLVLIRLVALAFFLMWRIKHQNDDAIWLWGMSIVCELWFAFSWVLDQLPKLCPINRATDLSVLKEK 354
TRIAE_CS42_1DL_TGACv RKLQIPAAVISPYRLLVLIRLVALAFFLMWRIKHQNDDAIWLWGMSIVCELWFALSWVLDQLPKLCPINRATDLSVLKEK 354
```

```
TRIAE_CS42_1AL_TGACv RKLQIPAAVISPYRLLVLIRLVALAFFLMWRIKHQNDDAIWLWGMSIVCELWFALSWVLDQLPKLCPINRATDLSVLKEK 354
TRIAE_CS42_2DS_TGACv RKIPMPASIISPYRIFIVIRFFVLIFYLTWRIRNPNMEALWLWGMSIVCELWFAFSWLLDMLPKVNPINRSTDLAVLKEK 336
TRIAE_CS42_2AS_TGACv RKIPMPTSIISPYRIFIVIRFFVLIFYLTWRIRNPNMEALWLWGMSIVCELWFAFSWLLDMLPKVNPINRSTDLAVLKEK 336
TRIAE_CS42_2BS_TGACv RKIPMPTSIISPYRIFIVIRFFVLIFYLTWRIRNPNMEALWLWGMSIVCELWFAFSWLLDMLPKVNPINRSTDLAVLKEK 337
TRIAE_CS42_7BL_TGACv RKVAIPPGILSPYRLLVLVRFVALFLFLIWRATNPNPDAMWLWGISIVCEYWFALSWLLDQMPKLNPINRAADLAALREK 219
TRIAE_CS42_7DL_TGACv RKVAIPPGILSPYRLLVLVRFVALFLFLVWRATNPNPDAMWLWGISIVCEYWFALSWLLDQMPKLNPINRAADLAALREK 219
TRIAE_CS42_7AL_TGACv RKVAIPPGILSPYRLLVLVRFVALFLFLVWRATNPNPDAMWLWGISIVCEYWFALSWLLDQMPKLNPINRAADLAALREK 218
TRIAE_CS42_1BS_TGACv -------------------------------------------------------------------------------- 145
TRIAE_CS42_5BS_TGACv RKTSVSQAILSPYRMLIAIRLVALGFFLAWRIRHPNPDAMWLWALSVTCEVWFAFSWLLDSLPKLCPVNRSCDLDVLADR 396
TRIAE_CS42_5DS_TGACv RKTSVSQAILSPYRMLIAIRLVALGFFLAWRIRHPNPDAMWLWALSVTCEVWFAFSWLLDSLPKLCPVNRSCDLDVLADR 400

TRIAE_CS42_1BL_TGACv FETPTPSNPTGKSDLPGIDIFVSTADPEKEPVLVTANTILSILAVDYPVDKLACYVSDDGGALLTFEAMAEAASFANFWV 434
TRIAE_CS42_1DL_TGACv FETPTPSNPTGKSDLPGIDIFVSTADPEKEPVLVTANTILSILAVDYPVDKLACYVSDDGGALLTFEAMAEAASFANFWV 434
TRIAE_CS42_1AL_TGACv FETPTPSNPTGKSDLPGIDIFVSTADPEKEPVLVTANTILSILAVDYPVDKLACYVSDDGGALLTFEAMAEAASFANFWV 434
TRIAE_CS42_2DS_TGACv FETPSPSNPHGRSDLPGLDVFVSTADPEKEPVLTTANTILSILAVDYPVEKLACYVSDDGGALLTFEAMAEAASFANIWV 416
TRIAE_CS42_2AS_TGACv FETPSPSNPHGRSDLPGLDIFVSTADPEKEPVLTTANTILSILAVDYPVEKLACYVSDDGGALLTFEAMAEAASFANIWV 416
TRIAE_CS42_2BS_TGACv FETHSPSNPHGRSDLPGLDVFVSTADPEKEPVLTTANTILSILAVDYPVEKLACYVSDDGGALLTFEAMAEAASFANIWV 417
TRIAE_CS42_7BL_TGACv FESKTPSNPTGRSDLPGLDVFISTADPYKEPPLVTANTLLSILATDYPVEKLFVYISDDGGALLTFEAMAEACAYAKVWV 299
TRIAE_CS42_7DL_TGACv FESKTPSNPTGRSDLPGLDVFISTADPYKEPPLVTANTLLSILATDYPVEKLFVYISDDGGALLTFEAMAEACAYAKVWV 299
TRIAE_CS42_7AL_TGACv FESKTPSNPTGRSDLPGLDVFISTADPYKEPPLVTANTLLSILATDYPVEKLFVYISDDGGALLTFEAMAEACAYAKVWV 298
TRIAE_CS42_1BS_TGACv -------------------------------------------------------------------------------- 145
TRIAE_CS42_5BS_TGACv FELPTARNPKGRSDLPGIDVFVSTADPEKEPPLVTANTILSILAADYPVEKLACYLSDDGGALLTFEALAETASFARTWV 476
TRIAE_CS42_5DS_TGACv FELPTARNPKGRSDLPGIDVFVSTADPEKEPPLVTANTILSILAADYPVEKLACYLSDDGGALLTFEALAETASFARTWV 480

TRIAE_CS42_1BL_TGACv PFCRKHDIEPRNPDSYFNLKRDPFKNKVKADFVKDRRRIKREYDEFKVRVNGLPDSIRRRSDAYHAREEIQAMNLQREKI 514
TRIAE_CS42_1DL_TGACv PFCRKHDIEPRNPDSYFNLKRDPFKNKVKADFVKDRRRIKREYDEFKVRVNGLPDSIRRRSDAYHAREEIQAMNLQREKI 514
TRIAE_CS42_1AL_TGACv PFCRKHDIEPRNPDSYFNLKRDPFKNKVKADFVKDRRRIKREYDEFKVRVNGLPDSIRRRSDAYHAREEIQAMNLQREKI 514
TRIAE_CS42_2DS_TGACv PFCKKHDIEPRNPDSYFALKGDPTKGKRRSDFVKDRRKVKREYDEFKVRINGLPDSIRRRSDAFNAREDMKML----KHL 492
TRIAE_CS42_2AS_TGACv PFCKKHDIEPRNPDSYFALKGDPTKGKRRSDFVKDRRKVKREYDEFKVRINGLPDSIRRRSDAFNAREDMKML----KHL 492
TRIAE_CS42_2BS_TGACv PFCKKHDIEPRNPDSYFALKGDPTKGKRRSDFVKDRRKVKREYDEFKVRINGLPDSIRRRSDAFNAREDMKML----KHL 493
TRIAE_CS42_7BL_TGACv PFCRKHSIEPRNPEAYFTQKGDPTKGKKRPDFVKDRRWIKREYDEYKVRINDLPEAIKRRAKAMNAHERKIAR----ETA 375
TRIAE_CS42_7DL_TGACv PFCRKHSIEPRNPEAYFTQKGDPTKGKKRPDFVKDRRWIKREYDEYKVRINDLPEAIKRRAKAMNAHERKIAR----ETA 375
TRIAE_CS42_7AL_TGACv PFCRKHSIEPRNPEAYFTQKGDPTKGKKRPDFVKDRRWIKREYDEYKVRINDLPEAIKRRAKAMNAHERKIAR----ETA 374
TRIAE_CS42_1BS_TGACv -------------------------------------------------------------------------------- 145
TRIAE_CS42_5BS_TGACv PFCRKHGVEPRCPESYFGQKRDFLKNKVRLDFVRERRKVKREYDEFKVRVNSLTEAIRRRSDAYNAGEELRARRRLQEEA 556
TRIAE_CS42_5DS_TGACv PFCRKHGVEPRCPESYFGQKRDFLKNKVRLDFVRERRKVKREYDEFKVRVNSLTEAIRRRSDAYNAGEELRARRRLQEEA 560

TRIAE_CS42_1BL_TGACv KAGGDEQFEPV----KIPKATWMADSTHWPGTWIHSSQDHARGDHAGIIQVMLKPPSDMPMYG--NIEK-SPLDFSEVDT 587
TRIAE_CS42_1DL_TGACv KAGGDEQFEPV----KIPKATWMADSTHWPGTWIHSSQDHARGDHAGIIQVMLKPPSDMPMYG--NIEK-SPLDFSEVDT 587
TRIAE_CS42_1AL_TGACv KAGGDEQFEPV----KIPKATWMADSTHWPGTWIHPSQDHARGDHAGIIQVMLKPPSDMPMYG--NIEK-SPLDFSGVDT 587
TRIAE_CS42_2DS_TGACv RETGADPSEQP----KVKKATWMADGTHWPGTWAVSSPDHAKGNHAGILQVMLRPPSPDPLYG--MHDEDQLIDYSDVDT 566
TRIAE_CS42_2AS_TGACv RETGADPSEQP----KVKKATWMADGTHWPGTWAVSSPDHAKGNHAGILQVMLRPPSPDPLYG--MHDEDQLIDYSDVDT 566
TRIAE_CS42_2BS_TGACv RETGADPSEQP----KVKKATWMADGTHWPGTWAVSSPDHAKGNHAGILQVMLRPPSPDPLYG--MHDEDQLVDYSDVDT 567
TRIAE_CS42_7BL_TGACv AAS---SDAAP----PPVKATWMADGTHWPGTWLDSAPDHGKGDHASIVQVMIKNPHHDVVYG--EADDHAYLDFTNVDV 446
TRIAE_CS42_7DL_TGACv AAS---SDAAP----PPVKATWMADGTHWPGTWLDSAPDHGKGDHASIIQVMIKNPHHDVVYG--DADDHAYLDFTNVDV 446
TRIAE_CS42_7AL_TGACv AAS---SDAAP----PPVKATWMADGTHWPGTWLDSAPDHGKGDHASIVQVMIKNPHHDVVYG--DADDHAYLDFTNVDV 445
TRIAE_CS42_1BS_TGACv -------------------ATWMSDGSQWASTWLAGATDHARGNHAGIIQ------------------------------ 176
TRIAE_CS42_5BS_TGACv VAAGGALGTAPLAETGAVKATWMSDGSQWPGTWLTGATDHARGNHAGIIQAMLAPPTSEPVLGGEPAESGALIDTTGVDI 636
TRIAE_CS42_5DS_TGACv VAAGGALGAAPLAETGAVKGTWMSDGSQWPGTWLTGATDHARGDHAGIIQAMLAPPTSEPVLGGVPAESGALIDTTGVDI 640

TRIAE_CS42_1BL_TGACv RLPMLVYMSREKRPGYDHNKKAGAMNALVRASAIMSNGPFILNLDCDHYVYNSKAFREGMCFMMDRGGDRLCYVQFPQRF 667
TRIAE_CS42_1DL_TGACv RLPMLVYMSREKRPGYDHNKKAGAMNALVRASAIMSNGPFILNLDCDHYVYNSKAFREGMCFMMDRGGDRLCYVQFPQRF 667
TRIAE_CS42_1AL_TGACv RLPMLVYMSREKRPGYDHNKKAGAMNALVRASAIMSNGPFILNLDCDHYVYNSKAFREGMCFMMDRGGDRLCYVQFPQRF 667
TRIAE_CS42_2DS_TGACv RLPMLVYMSREKRPGYDHNKKAGAMNALVRCSAVMSNAPFILNFDCDHYINNNQAVREAMCFMMDRGGERICYIQFPQRF 646
TRIAE_CS42_2AS_TGACv RLPMLVYMSREKRPGYDHNKKAGAMNALVRCSAVMSNAPFILNFDCDHYINNNQAVREAMCFMMDRGGERICFIQFPQRF 646
TRIAE_CS42_2BS_TGACv RLPMLVYMSREKRPGYDHNKKAGAMNALVRCSAVMSNAPFILNFDCDHYINNTQAIREAMCFMMDRGGERICYIQFPQRF 647
TRIAE_CS42_7BL_TGACv RIPMFVYLSREKRPGYDHNKKAGAMNAMVRASAVLSNGPFMLNFDCDHYVYNCQAIREAMCYMLDRGGDRICYIQFPQRF 526
TRIAE_CS42_7DL_TGACv RIPMFVYLSREKRPGYDHNKKAGAMNAMVRASAVLSNGPFMLNFDCDHYVYNCQAIREAMCYMLDRGGDRICYIQFPQRF 526
TRIAE_CS42_7AL_TGACv RIPMFVYLSREKRPGYDHNKKAGAMNAMVRASAVLSNGPFMLNFDCDHYVYNCQAIREAMCYMLDRGGDRICYIQFPQRF 525
TRIAE_CS42_1BS_TGACv ------------RPGYNHNKKAGAMNALVRTSAIMSNGPFILNLDCDHYVHNSAALREGMCYMLDCRGDRVCYVQFPQRF 244
TRIAE_CS42_5BS_TGACv RLPMLVYVSREKRPGYDHNKKAGAMNALVRTSAIMSNGPFILNLDCDHYVHNSAALREGMCYMLDRGGDRVCYVQFPQRF 716
TRIAE_CS42_5DS_TGACv RLPMLVYVSREKRPGYDHNKKAGAMNALVRTSAIMSNGPFILNLDCDHYVHNSAALREGMCYMLDRGGDRVCYVQFPQRF 720

TRIAE_CS42_1BL_TGACv EGIDPSDRYANHNTVFFDINMRALDGLQGPVYVGTGCLFRRIALYGFDPPRSKDHSPGFCGCCLPRRRKASASNANPEET 747
TRIAE_CS42_1DL_TGACv EGIDPSDRYANHNTVFFDINMRALDGLQGPVYVGTGCLFRRIALYGFDPPRSKDHSPGFCGCCLPRRRKASASNANPEET 747
TRIAE_CS42_1AL_TGACv EGIDPSDRYANHNTVFFDINMRALDGLQGPVYVGTGCLFRRIALYGFDPPRSKDHSPGFCGCCLPRRRKASASNANPEET 747
TRIAE_CS42_2DS_TGACv EGIDPSDRYANHNTVFFDGNMRALDGLQGPMYVGTGCMFRRFALYGFDPPRTAEYTG------WLFKKKKVTNFKDPESD 720
TRIAE_CS42_2AS_TGACv EGIDPSDRYANHNTVFFDGNMRALDGLQGPMYVGTGCMFRRFALYGFDPPRTAEYTG------WLFKKKKVTNFKDPDSD 720
TRIAE_CS42_2BS_TGACv EGIDPSDRYANHNTVFFDGNMRALDGLQGPMYVGTGCMFRRFALYGFDPPRTAEYTG------WLFKKKKVTNFKDPESD 721
TRIAE_CS42_7BL_TGACv EGIDPSDRYANHNTVFFDGNMRALDGLQGPMYVGTGCLFRRYAIYGFNPPRAVEYHG------LVG-QTRVPIDPHARSG 599
TRIAE_CS42_7DL_TGACv EGIDPSDRYANHNTVFFDGNMRALDGLQGPMYVGTGCLFRRYAIYGFNPPRAVEYHG------LVG-QTRVPIDPHARSG 599
TRIAE_CS42_7AL_TGACv EGIDPSDRYANHNTVFFDGNMRALDGLQGPMYVGTGCLFRRYAIYGFNPPRAVEYHG------LVG-QTRVPIDPNARSG 598
TRIAE_CS42_1BS_TGACv EGIDPNDRYANHNLVFFDVAMRAMDGLQGPMYVGG-CIFRRIALYGFSPPRATKHHGWLG-RKIKLFLRKPTMGKKTDRE 322
TRIAE_CS42_5BS_TGACv EGIDPNDRYANHNLVFFDVAMRAMDGLQGPMYVGTGCIFRRTALYGFSPPRATEHHGWLGRKKIKLFLRKPTMGKKTDRE 796
TRIAE_CS42_5DS_TGACv EGIDPNDRYANHNLVFFDVAMRAMDGLQGPMYVGTGCIFRRTALYGFSPPRATEHHGWLGRKKIKLFLRKPTTGKKTDRE 800

TRIAE_CS42_1BL_TGACv MALRMGDFDGDS---------MNLATFPKKFGNSSFLIDSIPVAEFQGRPLADHPSVKNGRPPGALTIPREILDASIVAE 818
TRIAE_CS42_1DL_TGACv MALRMGDFDGDS---------MNLATFPKKFGNSSFLIDSIPVAEFQGRPLADHPSVKNGRPPGALTIPREILDASIVAE 818
TRIAE_CS42_1AL_TGACv MALRMGDFDGDS---------MNLATFPKKFGNSSFLIDSIPVAEFQGRPLADHPSVKNGRPPGALTIPREILDASIVAE 818
TRIAE_CS42_2DS_TGACv TQKLKAEDFDAE---------LTAQLVPRRFGNSSAMLASIPIAEFQARPIADHPAVLHGRPPGTLTVPRPPLDPPTVAE 791
TRIAE_CS42_2AS_TGACv TQQLKAEDFDAE---------LTAQLVPRRFGNSSAMLASIPIAEFQARPIADHPAVLHGRPPGTLTVPRPPLDPPTVAE 791
```

```
TRIAE_CS42_2BS_TGACv TQQLKAEDFDAE---------LTAQLVPRRFGNSSAMLASIPIAEFQARPIADHPAVLHGRPPGTLTVPRPPLDPPTVAE 792
TRIAE_CS42_7BL_TGACv DGVADELRPLSD---------HPDHEAPQRFGKSKMFIESIAVAEYQGRPLADHPSVRNGRPAGALLMPRPPLDAATVAE 670
TRIAE_CS42_7DL_TGACv DGIADELRPLSD---------HPDHEAPQRFGKSKMFIESIAVAEYQGRPLADHPSVRNGRPAGALLMPRPPLDAATVAE 670
TRIAE_CS42_7AL_TGACv DGVADELRPLSD---------HPDHEAPQRFGKSKMFIESIAVAEYQGRPLADHPSVRNGRPPGALLMPRPPLDAATVAE 669
TRIAE_CS42_1BS_TGACv LVMAILQK------------------------------------------------------------------------ 330
TRIAE_CS42_5BS_TGACv SEHESMLPPIEDDDHNQLGDGVRGDLLLPQQRTVRHPADEAPAARGLLQRGHVPVHLHVPHRLLRAPGRLPLHRQVHRPA 876
TRIAE_CS42_5DS_TGACv SEHESMLPPIEDDDHNQLGDIESSALMPKRFGSSATFVSSIPVAEYQGRLLQDMPGVHQGRPAGALAVPREPLDAATVGE 880

TRIAE_CS42_1BL_TGACv AISVVSCWYEEKTEWGTRVGWIYGSVTEDVVTGYRMHNRGWKSVYCVTQRDAFRGTAPINLTDRLHQVLRWATGSVEIFF 898
TRIAE_CS42_1DL_TGACv AISVVSCWYEEKTEWGTRVGWIYGSVTEDVVTGYRMHNRGWKSVYCVTQRDAFRGTAPINLTDRLHQVLRWATGSVEIFF 898
TRIAE_CS42_1AL_TGACv AISVVSCWYEEKTEWGTRVGWIYGSVTEDVVTGYRMHNRGWKSVYCVTQRDAFRGTAPINLTDRLHQVLRWATGSVEIFF 898
TRIAE_CS42_2DS_TGACv AVSVISCWYEDKTEWGDRVGWIYGSVTEDVVTGYRMHNRGWRSVYWISKRDAFLGTAPINMTDRLHQVLRWATGSVEIFF 871
TRIAE_CS42_2AS_TGACv AVSVISCWYEDKTEWGDRVGWIYGSVTEDVVTGYRMHNRGWRSVYWISKRDAFLGTAPINMTDRLHQVLRWATGSVEIFF 871
TRIAE_CS42_2BS_TGACv AVSVISCWYEDKTEWGDRVGWIYGSVTEDVVTGYRMHNRGWRSVYWISKRDAFLGTAPINMTDRLHQVLRWATGSVEIFF 872
TRIAE_CS42_7BL_TGACv AVSVISCWYEDNTEWGLRVGWIYGSVTEDVVTGYRMHNRGWRSVYCITKRDAFRGTAPINLTDRLHQVLRWATGSVEIFF 750
TRIAE_CS42_7DL_TGACv AVSVISCWYEDNTEWGLRVGWIYGSVTEDVVTGYRMQNRGWRSVYCITKRDAFRGTAPINLTDRLHQVLRWATGSVEIFF 750
TRIAE_CS42_7AL_TGACv AVSVISCWYEDNTEWGLRVGWIYGSVTEDVVTGYRMHNRGWRSVYCITKRDAFRGTAPINLTDRLHQVLRWATGSVEIFF 749
TRIAE_CS42_1BS_TGACv -------------------------------------------------------------------------------- 330
TRIAE_CS42_5BS_TGACv PERHVPRLPAHHHHHAVPAGAAGDQVVRDHAARVVAQRAVLGDRRHQRAPGCGAAGPPQGDRRRGHLLHAHVQAGRRRRR 956
TRIAE_CS42_5DS_TGACv AISVISCFYEEKTEWGRRIGWIYGSVTEDVVTGYRMHNRGWRSVYCVTRRDAFRGTAPINLTDRLHQVLRWATGSVEIFF 960

TRIAE_CS42_1BL_TGACv SRNNALFASSKMKVLQRIAYLNVGIYPFTSIFLIVYCFLPALSLFSGQFIVQTLNVTFLTYLLIITITLCLLAMLEIKWS 978
TRIAE_CS42_1DL_TGACv SRNNALFASSKMKVLQRIAYLNVGIYPFTSIFLIVYCFLPALSLFSGQFIVQTLNVTFLTYLLIITVTLCLLAMLEIKWS 978
TRIAE_CS42_1AL_TGACv SRNNALFASSKMKVLQRIAYLNVGIYPFTSIFLIVYCFLPALSLFSGQFIVQTLNVTFLTYLLIITVTLCLLAMLEIKWS 978
TRIAE_CS42_2DS_TGACv SRNNAFLASRKLMFLQRVAYLNVGIYPFTSIFLLTYCFIPALSLFSGFFIVQTLNVAFLFYLLTITVTLIALGILEVKWS 951
TRIAE_CS42_2AS_TGACv SRNNAFLASRKLMFLQRVAYLNVGIYPFTSIFLLTYCFIPALSLFSGFFIVQTLNVAFLFYLLTITITLIALGILEVKWS 951
TRIAE_CS42_2BS_TGACv SRNNAFLASRKLMFLQRVAYLNVGIYPFTSIFLLTYCFIPALSLFSGFFIVQTLNVAFLFYLLTITVTLIALGILEVKWS 952
TRIAE_CS42_7BL_TGACv SKNNAMLASRRLMFLQRMSYINVGIYPFTSLFLIMYCLLPALSLFSGQFIVATLDPTFLCYLLLITVTLVLLCLLEVKWS 830
TRIAE_CS42_7DL_TGACv SKNNAMLASRRLMFLQRMSYINVGIYPFTSLFLIMYCLLPALSLFSGQFIVATLDPTFLCYLLLITVTLVLLCLLEVKWS 830
TRIAE_CS42_7AL_TGACv SKNNALLASRRLMFLQRMSYINVGIYPFTSLFLIMYCLLPALSLFSGQFIVATLDPTFLCYLLLITITLVLLCLLEVKWS 829
TRIAE_CS42_1BS_TGACv -------------------------------------------------------------------------------- 330
TRIAE_CS42_5BS_TGACv GGGHVRGAVRGAVELPDGAPRDHHDAERGGAGGGDGEDAVQRVPAVEQAAGRRLLQLLGAVPPLPL-------------- 1022
TRIAE_CS42_5DS_TGACv SRNNALFATRRMKLLQRVAYFNVGMASSR--------------------------------------------------- 989

TRIAE_CS42_1BL_TGACv GIALEEWWRNEQFWLIGGTSAHLAAVMQGLLKVVAGIEISFTLTSKQVGDDIDDEFAELYEVKWTSLMIPPLTIIMVNLV 1058
TRIAE_CS42_1DL_TGACv GIALEEWWRNEQFWLIGGTSAHLAAVMQGLLKNSTK-------------------------------------------- 1014
TRIAE_CS42_1AL_TGACv GIALEEWWRNEQFWLIGGTSAHLAAVMQGLLKVVAGIEISFTLTSKQVGDDIDDEFAELYEVKWTSLMIP---------- 1048
TRIAE_CS42_2DS_TGACv GIELEDWWRNEQFWLISGISAHLYAVVQGLLKVMAGIEISFTLTAKAAAEDNEDIYADLYVVKWSSLLIP---------- 1021
TRIAE_CS42_2AS_TGACv GIELEDWWRNEQFWLISGISAHLYAVVQGLLKVMAGIEISFTLTAKAAADDNEDIYADLYVVKWSSLLIP---------- 1021
TRIAE_CS42_2BS_TGACv GIELEDWWRNEQFWLISGISAHLYAVVQGLLKVMAGIEISFTLTAKAAAEDNEDIYADLYVVKWSSLLIP---------- 1022
TRIAE_CS42_7BL_TGACv GIGLEEWWRNEQFWVIGGTSAHLAAVLQGLLKVAAGIEISFTLTAKAAAEDDDDPFAELYLIKWTSLFIP---------- 900
TRIAE_CS42_7DL_TGACv GIGLEEWWRNEQFWVIGGTSAHLAAVLQGLLKVAAGIEISFTLTAKAAAEDDDDPFAELYLIKWTSLFIP---------- 900
TRIAE_CS42_7AL_TGACv GIGLEEWWRNEQFWVIGGTSAHLAAVLQGLLKVAAGIEISFTLTAKAAAEDDDDPFAELYLIKWTSLFIP---------- 899
TRIAE_CS42_1BS_TGACv -------------------------------------------------------------------------------- 330
TRIAE_CS42_5BS_TGACv -------------------------------------------------------------------------------- 1022
TRIAE_CS42_5DS_TGACv -------------------------------------------------------------------------------- 989

TRIAE_CS42_1BL_TGACv AIAVGFSRTIYSTDIDDEFAELYEVKWTSLMIPPLTIIMVNLVAIAVGFSRTIYSTIPQWSKLLGGVFFSFWVLAHLYPF 1138
TRIAE_CS42_1DL_TGACv -------------------------------------------------------------------------------- 1014
TRIAE_CS42_1AL_TGACv ---------------------------------PLTIIMVNLVAIAVGFSRTIYSTIPQWSKLLGGVFFSFWVLAHLYPF 1095
TRIAE_CS42_2DS_TGACv ---------------------------------PITIGMLNIIAIAFAFARTIYSDNPRWGKFIGGGFFSFWVLAHLNPF 1068
TRIAE_CS42_2AS_TGACv ---------------------------------PITIGMLNIIAIAFAFARTIYSENPRWGKFIGGGFFSFWVLAHLNPF 1068
TRIAE_CS42_2BS_TGACv ---------------------------------PITIGMLNIIAIAFAFARTIYSDNPRWGKFIGGGFFSFWVLAHLNPF 1069
TRIAE_CS42_7BL_TGACv ---------------------------------PLAIIGINIIAMVVGVSRCVYAEIPQYSKLLGGGFFSFWVLAHYYPF 947
TRIAE_CS42_7DL_TGACv ---------------------------------PLAIIGINIIAMVVGVSRCVYAEIPQYSKLLGGGFFSFWVLAHYYPF 947
TRIAE_CS42_7AL_TGACv ---------------------------------PLAIIGINIIAMVVGVSRCVYAEIPQYSKLLGGGFFSFWVLAHYYPF 946
TRIAE_CS42_1BS_TGACv -------------------------------------------------------------------------------- 330
TRIAE_CS42_5BS_TGACv -------------------------------------------------------------------------------- 1022
TRIAE_CS42_5DS_TGACv -------------------------------------------------------------------------------- 989

TRIAE_CS42_1BL_TGACv AKGLMGRRGRTPTIVYVWAGLVSITISLLWIAINPPSSAANQQLGGSFSFP- 1189
TRIAE_CS42_1DL_TGACv ---------------------------------------------------- 1014
TRIAE_CS42_1AL_TGACv AKGLMGRRGRTPTIVYVWAGLVSITISLLWIAINPPSTAANQQLGGSFSFP- 1146
TRIAE_CS42_2DS_TGACv AKGLMGRRGKTPTIIFVWSGLISITISLLWVALSPPEANSTGGARGGGFQFP 1120
TRIAE_CS42_2AS_TGACv AKGLMGRRGKTPTIIFVWSGLISITISLLWVALSPPEANSTGGARSGGFQFP 1120
TRIAE_CS42_2BS_TGACv AKGLMGRRGKTPTIIFVWSGLISITISLLWVALSPPEANSTGGARGGGFQFP 1121
TRIAE_CS42_7BL_TGACv AKGLMGRRGRTPTIVYVWAGLISITVSLLWITISPPDDRVSQSGIEV----- 994
TRIAE_CS42_7DL_TGACv AKGLMGRRGRTPTIVYVWAGLISITVSLLWITISPPDDRVSQSGIEV----- 994
TRIAE_CS42_7AL_TGACv AKGLMGRRGRTPTIVYVWAGLISITVSLLWITISPPDDRVSQSGIEV----- 993
TRIAE_CS42_1BS_TGACv ---------------------------------------------------- 330
TRIAE_CS42_5BS_TGACv ---------------------------------------------------- 1022
TRIAE_CS42_5DS_TGACv ---------------------------------------------------- 989
```

Fig. S_2D: *CslE* subfamily.

S.No	Gene name with number of splice variants (CslE)	No. of amino acids (aa)
1	TRIAE_CS42_6DL_TGACv1_526558_AA1687090.1	738 aa
2	TRIAE_CS42_6AL_TGACv1_471004_AA1500600.1_3_SPLICE	737 aa
3	TRIAE_CS42_6BL_TGACv1_499967_AA1596110.2	736 aa
4	TRIAE_CS42_U_TGACv1_683314_AA2158770.1	446 aa
5	TRIAE_CS42_5DL_TGACv1_433536_AA1415840.1	756 aa
6	TRIAE_CS42_5BL_TGACv1_406235_AA1342610.1	728 aa
7	TRIAE_CS42_5AL_TGACv1_376126_AA1232370.2	728 aa
8	TRIAE_CS42_5DL_TGACv1_433536_AA1415830.1_2_SPLICE	728 aa
9	TRIAE_CS42_5BL_TGACv1_406235_AA1342600.1_2_SPLICE	734 aa
10	TRIAE_CS42_6DS_TGACv1_543277_AA1737920.1	725 aa

```
Color Align Conservation results
TRIAE_CS42_5DL_TGACv MVAIGRRTGQQHGHWRLAAESPPYLGPRDGEEHEAVRDGDSRGPGGVQAPRRHGGRRILLLLYYRATRVPAAGEGRAAWL 80
TRIAE_CS42_5BL_TGACv ----------------------------MERSRRLFETETHGGRAAYRLHAVTVAAGILLVLYYRATHVPAAGEGRATWL 52
TRIAE_CS42_U_TGACv1_ -------------------------------------------------------------------------------- 0
TRIAE_CS42_5AL_TGACv -----------------------------MERTRLFETETHGGRAAYRLHAVTVAAGILLLLYYRATRVPAAGEGRAAWL 51
TRIAE_CS42_6DS_TGACv ------------------------------MERRLFETVRHGGRALYRLHAVTVAASTLLVLYYRATRVPGSGGRRAAWL 50
TRIAE_CS42_5DL_TGACv ----------------------------MERTRRLFETETHGGRAAYRLHAVTVAAGILLLLYYRATHVPAAGEGRAAWL 52
TRIAE_CS42_5BL_TGACv ----------------------------MERSRRLFETETHGGRAVYRLHAVTVAAGILLLLYYRATRVPAAGEGRAAWL 52
TRIAE_CS42_6AL_TGACv --------------------MAGSSVSGGGGRPPLFATEKPKRVLAYRVYAGTIFAGILLIWFYRATHIPARGSSSLGWR 60
TRIAE_CS42_6BL_TGACv ---------------------MAGSSVSGGGRPPLFATEKPKRVLAYRLYAGTIFAGILLIWFYRATHIPERGDSSLGWR 59
TRIAE_CS42_6DL_TGACv -------------------MAGSSVSGGGGGRPPLFATEKPKRVLAYRLYAGTIFAGILLIWFYRATHIPARGSSSLGWR 61

TRIAE_CS42_5DL_TGACv G---MLAAELWYAAYWVVTQSVRWSPVRRRPFIDRLAARHG-ETLPCVDIFVCTADPYSEPPSLVVSTILSLMAYNYPPE 156
TRIAE_CS42_5BL_TGACv G---MLAAELWYAAYWVVTQSVRWSPVRRRPFIDRLAARHG-ERLPCVDIFVCTADPYSEPPSLVVSTILSLMAYNYPPE 128
TRIAE_CS42_U_TGACv1_ -------------------------------------------------------------------------------- 0
TRIAE_CS42_5AL_TGACv G---MLAAELWYAAYWVVTQSVRWSPVRRRPFRDRLAARHG-ERLPSVDIFVCTADPYSEPPSLVVSTILSLMAYNYPPE 127
TRIAE_CS42_6DS_TGACv G---MLAAELWYAAYWVVTQSVRWSPVRRCTFRDRLTARYG-DRLPGVDIFVCTADPLSEPPSLVISTILSVMAYNYLAE 126
TRIAE_CS42_5DL_TGACv G---MLAAELWYAAYWAVTQSVRWSPVRRLPFIDRLAARYG-ERLPCVDIFVCTADPHSEPPSLVISTVLSLMAYNYPAE 128
TRIAE_CS42_5BL_TGACv G---MLAAELCYAAYWVVTQSVRWSPLHRRPCRDRLAARYG-ERLPCVDIFVCTADPHSEPPSLVISTVLSLMAYNYPAE 128
TRIAE_CS42_6AL_TGACv AGLGLLVAEILFGLYWVLTLSVRWNPVRRTTFKDRLSERYDDDQLPGVDIFVCTADPALEPPMLVISTVLSVMAYDYPPE 140
TRIAE_CS42_6BL_TGACv AGLGLLVAELLFGLYWVLTLSVRWNPVRRTTFKDRLSERYDDDQLPGVDIFVCTADPALEPPMLVISTVLSVMAYDYPPE 139
TRIAE_CS42_6DL_TGACv AGLGLLVAELWFGLYWVLTLSVRWNPVRRATFKDRLSERYDDDQLPGVDIFVCTADPALEPPMLVISTVLSVMAYDYPPE 141

TRIAE_CS42_5DL_TGACv KLSVYLSDDGGSILTFYGMWEASLFAKHWLPFCKRYNIEPRSPAAYFSESDGHQELCNPKEWSLIKDMFDKMTERIDTVV 236
TRIAE_CS42_5BL_TGACv KLSVYLSDDGGSILTLYGMWEASLFAKHWLPFCKRYNIEPRSPAAYFSESDGHQELCTPKEWSLIKDMFDKMTERIDTAV 208
TRIAE_CS42_U_TGACv1_ -------------------------------------------------------------------------------- 0
TRIAE_CS42_5AL_TGACv KLSVYLSDDGGSILTYYGMWEASLFAKHWLPFCKRYNIEPRSPAAYFSQSDGHQELCTPKEWSLIKDMFDEMTERIDTAV 207
TRIAE_CS42_6DS_TGACv KLSVYLSDDGGSVLTFYAMWEASLFAKHWLPFCKRYNIEPRSPAAYFSES--YQDLCTPKEWSFIKDMYDEMTERIDTAV 204
TRIAE_CS42_5DL_TGACv KISVYLSDDGGSVLTFYALWEASLFAKHWIPFCKRYNIEPRSPAAYFSESDGHQDLCSPKEWSLIREMYEDMTERIDTAV 208
TRIAE_CS42_5BL_TGACv KISVYLSDDGGSILTFYALWEASLFAKHWIPFCKRYNIEPRSPATYFSESDGHQDMCTPKEWSLIREMYEDMTERIDTAA 208
TRIAE_CS42_6AL_TGACv KLNIYLSDDAGSAVTFYALHEASEFAKHWIPFCKNYKVEPRSPAAYFAEGATPHDACSPQELLRMKELYKDLTDRVNSVV 220
TRIAE_CS42_6BL_TGACv KLNIYLSDDAGSAVTFYALHEASEFAKHWIPFCKNYKVEPMSPAAYFAEGATPHDACSPQELLRMKELYKDLTDRVNSVV 219
TRIAE_CS42_6DL_TGACv KLNIYLSDDAGSAVTFYALHEASEFAKHWIPFCKNYKVEPRSPAAYFAKGATPHDACSPQEFLRMKELYKDLTDRMNSVV 221

TRIAE_CS42_5DL_TGACv MSGKVPEEIKASHKGFYEWNQEITSKNHQPIVQILIDSKDQNAVDNEGKVLPTLVYMAREKRPQHHHNFKAGAMNALIRV 316
TRIAE_CS42_5BL_TGACv MSGKVPEEIKARHKGFYEWNQEISSKNHQPIVQILIDGKDQNAVDNEGKVLPTLVYMAREKRPQHHHNFKAGAMNALIRV 288
TRIAE_CS42_U_TGACv1_ -----------------------------------------------------------------------------MQIRV 5
TRIAE_CS42_5AL_TGACv MSGKVPEEIKARQKGFHEWNQEITSKNHQPIVQILIDGKDQNAVDNEGNVLPTLVYMAREKRPQHHHNFKAGAMNALIRV 287
TRIAE_CS42_6DS_TGACv ISRKIPEEIRSNHKGFYEWNPEITSKNHQPIVQVLIDGKDQKGVDSEGNVLPTLVYMAREKRPQHHHNFKAGAMNALIRV 284
TRIAE_CS42_5DL_TGACv LSGKISEEVKANHKGFHEWDQENTSKNHQPIVQILIEGKDKNANDDEGNVLPTLVYMAREKRPQHHHNFKAGAMNALIRV 288
TRIAE_CS42_5BL_TGACv LSGKISEEVKENHKGFHEWDQENTSKNHQPIVQILIEGKDKNANDDEGNVLPTLVYMAREKRPQHHHNFKAGAMNALIRV 288
TRIAE_CS42_6AL_TGACv HSGKIPEVPECNHRGSSEWNEMITSGDHPSIVQILIDRNKRKAVDVDGNALPKLVYMSREKRPQEQHHFKAGSLNALIRV 300
TRIAE_CS42_6BL_TGACv HSGKIPEVPECNHRGFSVWNETITSGDHPSIVQILIDRNKRKAVDVDGNALPKLVYMAREKRPQEQHHFKAGSLNALIRV 299
TRIAE_CS42_6DL_TGACv HSGKIPEVPECNHRGFSEWNETITSGDHPSVVQILIDRNKRKAVDVDGNALPKLVYMAREKRPQEQHHFKAGSLNALIRV 301

TRIAE_CS42_5DL_TGACv SSVISNSPIIMNVDCDMYSNNNDAVRDALCFFLDEEMGHKIGFVQYPQNYYNLSKNNIYGNSLHVINEVEMGGMDSLGGP 396
TRIAE_CS42_5BL_TGACv SSVISNSPIIMNVDCDMYSNNNDAVRDALCFFLDEEMGHKIGFVQYPQNYNNLSKNNIYGNSLHVINEVEMGGMDSLGGP 368
TRIAE_CS42_U_TGACv1_ SSVISNSPIIMNVDCDMYSNNNDAVRDALCFFLDEEMGHKIGFVQYPQNYNNLSKNDIYGNSLHVINEVEMGGMDSLGGP 85
TRIAE_CS42_5AL_TGACv SSVISNSPIIMNVDCDMYSNNNDAVRDALCFFLDEEMGHKIGFVQYPQNYNNLSKNDIYGNSLQVINEVEMAGMDSLGGP 367
TRIAE_CS42_6DS_TGACv SSVISNSPVIMNVDCDMYSNNNDTIRDALCFFLDEEMGHKIGFVQYPQNYNNMTKNNLYGNSLHVINEVEMGGMDSLGGP 364
TRIAE_CS42_5DL_TGACv SSVISNSPIIMNVDCDMYSNNCDTIRDALCFFLDEEMGHKIGFVQFPQNYNNLTKNNIYGNSHQVTNQVLMGGMDSVGGP 368
TRIAE_CS42_5BL_TGACv SSVISNSPIIMNVDCDMYSNNYDTIRDALCFFLDEEMGHKIGFVQFPQNYNNLTKNNIYGNSHQVTNQVLMGGMDSVGGP 368
TRIAE_CS42_6AL_TGACv SSVISNSSVILNVDCDMYSNNSESIRDALCFFLDEEQGQDIGFVQYPQNFDNVVHNDIYGNPINVVNELDNPCLDCWGGM 380
TRIAE_CS42_6BL_TGACv SSVISSSPVILNVDCDMYSNNSESIRDALCFFLDEEQGQDIGFVQYPQNFDNVVHNDIYGNPINVVNELDNTCLDCWGGM 379
```

```
TRIAE_CS42_6DL_TGACv SSVISNSPVILNVDCDMYSNNSESIRDALCFFLDEEQGQDIGFVQYPQNFDNVVHNDSYGNPINVVNELDNPCLDGWGGM 381

TRIAE_CS42_5DL_TGACv MYIGTGCFHRREILCGRKFTEDYQEDWNAGIKDKLQES-IDETEEKAKSLAACTYEHGTQWADEIGVKYGCVVEDVNTGL 475
TRIAE_CS42_5BL_TGACv MYIGTGCFHRREILCGRKFTEDYQEDWNAGIKDKLQES-IDETEEKAKSLAACTYEHGTQWGDEIGVKYGCVVEDVNTGF 447
TRIAE_CS42_U_TGACv1_ LYIGTGCFHRREILCGRKFTKDYQEDWNAGIKDKLQES-IDETEEKAKSLAACTYEHGTQWGDEIGVKYGCAVEDVITGL 164
TRIAE_CS42_5AL_TGACv LYIGTGCFHRREILCGRKFTKDYQEDWNAGIKDKLQES-IDETEEKAKSLAACTYEHGTQWGDEIGVKYGCAVEDVITGL 446
TRIAE_CS42_6DS_TGACv LYVGTGCFHRREILRGKRFTKDYQEDWNGGIKGRIQDTSIGEIEEKAKSLATCTYEHDTQWGDEIGLKYGCPVEDVITGL 444
TRIAE_CS42_5DL_TGACv MYVGTGCFHRREILCGRRFTEDYKEDWNGGIKDKTQES-IVEIEEKAKSLAASTYEHDTQWGDEIGIKYGYPAEDIVTGL 447
TRIAE_CS42_5BL_TGACv MYVGTGCFHRREILCGRRFTKDYKEDWDGGIKDKTQES-IDEIEEKAKSLAASTYEHDTQWGDEIGIKYGYPAEDIVTGL 447
TRIAE_CS42_6AL_TGACv CYYGTGCFHRRETLSGQIYSKDYKEDWDRGVGIAENAD---ELEETSKSLVTCTYEHNTPWGIEKGVRYGCPLEDVITGL 457
TRIAE_CS42_6BL_TGACv CYYGTGCFHRRETLSGQIYSKDYKEDWARGVGIAENAD---ELEETSKSLVTCTYEHNTPWGIEKGVRYGCPLEDVITGL 456
TRIAE_CS42_6DL_TGACv CYYGTGCFHRRETLSGQIYSKDYKEDWARGVGIAENAD---ELEETSKSLVTCTYEHNTPWGIEKGVRYGCPLEDVITGL 458

TRIAE_CS42_5DL_TGACv AIHCRGWDSVYNNPKRPAFMGVGPTTLAQTIIQHKRWSEGIFSIFLSKYNVFLFAHGKTKLRHQMGYHIYGLWAPNSLAT 555
TRIAE_CS42_5BL_TGACv AIHCRGWESVYNNPKKPAFMGVGPTTLAQTIIQHKRWSEGIFSIFLSKYNVFLFAHGKTKLQHQMGYHIYGLWAPNSLAT 527
TRIAE_CS42_U_TGACv1_ AIHCRGWESVYNNPKKPAFMGVGPTTLAQTIIQHKRWSEGNLSIFLSKYNVFLFGHGKTKLRHQMGYHIYGLWAPNSLAT 244
TRIAE_CS42_5AL_TGACv AIHCRGWESVYNNPKKPAFMGVGPTTLAQTIIQHKRWSEGNLSIFLSKYNVFLFGHGKTKLRHQMGYHIYGLWAPNSLAT 526
TRIAE_CS42_6DS_TGACv AIHCRGWESVYSNPTRPAFMGLGPTTLAQTLIQHKRWSEGSFSIFLSKYCPFLFGHGKTNLRHQMGYCIYGLWAPNSLPT 524
TRIAE_CS42_5DL_TGACv GIHCRGWKSVHSNPPRPAFLGVAPTTLAQTLIQHKRWSEGSFSIFLSKYCPFMFGHGKIKLRHQMGYSIYGLWAPNSIPT 527
TRIAE_CS42_5BL_TGACv EIHCRGWKSVHSNPPRPAFLGVAPTTLAQTLIQHKRWSEGSFSIFLSKYCPFMFGHGKIKLRHQMGYSIYGLWAPNSIPT 527
TRIAE_CS42_6AL_TGACv QIQCRGWRSVYYNPARKGFLGMAPTSLGQILVQHKRWSEGFLQISLSNYSPFLLGHGKIKLGLQMGYSVCGFWALNSFPT 537
TRIAE_CS42_6BL_TGACv QIQCRGWRSVYYNPARKGFLGMAPTSLGQILVQHKRWSEGFLQISLSNYSPFLLGHGKIKLGLQMGYSVCGFWALNSFPT 536
TRIAE_CS42_6DL_TGACv QIQCHGWRSVYYNPARKGFLGKAPTSLGQILVQHKRWSEGFLQMSLSNYSPFLLGHGKIKLGLQMGYSVCGFWALNSFPT 538

TRIAE_CS42_5DL_TGACv LYYVIIPSLALLKGTSLFPEITSPWIAPFVYVFCVKNMYSLYEALLSGDTLKGWWNGQRMWLVKRITSYLFGVLDNLRKL 635
TRIAE_CS42_5BL_TGACv LYYVIIPSLALLKGTSLFPEITSPWIAPFVYVFCVKNMYSLYEALSSGDTLKGWWNGQRMWLVKRITSYLFGVLDNLRKL 607
TRIAE_CS42_U_TGACv1_ LYYVIIPSLALLKGTPLFPEITSPWIAPFVYVFCVKNMYSLYEALSSGDTLKGWWNGQRMWLVKRITSYLFGVLDNLRKL 324
TRIAE_CS42_5AL_TGACv LYYVIIPSLALLKGTPLFPEITSPWIAPFVYVFCVKNMYSLYEALSSGDTLKGWWNGQRMWLVKRITSYLFGVLDNLWKL 606
TRIAE_CS42_6DS_TGACv LYYVVIPSLALLKGIPLFPKITSPWIAPFIYAFCVKNMYSLYEALSCGDTLKGWWNGQRMWLVKRITSFLFGAIDTVRKL 604
TRIAE_CS42_5DL_TGACv LYYVIIPSLALLKGISLFPEITSPWMSPFIYVLCVKNMYSLYEALSCGDTLKGWWNEQRMWMVRRITSYLYGLTDTVRKL 607
TRIAE_CS42_5BL_TGACv LYYVIIPSLALLKGISLFPEITSPWISPFIYVVCVKNMYSLYEALSCGDTLKGWWNEQRMWMVRRITSYLYGLTDTVRKL 607
TRIAE_CS42_6AL_TGACv FYYVIIPSLCFLSGVSVFPEITSPWCIPFIYVVVASYSWSLMESLQCGDTAVEWWNAQRMWLMRRTTSYLLAAIDTIGGM 617
TRIAE_CS42_6BL_TGACv FYYVIIPSLCFLSGVSVFPEITSPWCIPFIYVVAASYSWSLMESLQCGDTAVEWWNAQRMWLMRRTTSYLLAAIDTIGGM 616
TRIAE_CS42_6DL_TGACv FYYVIIPSLCFLSGVSVFPEITSPWCIPFIYVVVASYSWSLMESLQCGDTAVEWWNAQRMWLMRRTTSYLLAAIDTIGGM 618

TRIAE_CS42_5DL_TGACv LGLSKINFVVTPKVSDEDESKRYEQEIMEFGSSDPEYVIIAAIALLNLVCLMGGLSKVMKGGWN-VHLDALFPQLILCGM 714
TRIAE_CS42_5BL_TGACv LGLSKMNFVVTPKVSDEDESKRYEQEIMEFGSSDPEYVIIGTITLLNLVCLLGGLSKVMKGGWN-EHLDALFPQLILCGM 686
TRIAE_CS42_U_TGACv1_ LGLSKMNFVVSPKVSDEDESKRYEQEIMEFGSSDPEYVIIGTITLLNLVCLLGGLSKVMKVGWNNIHLDALFPQLILCGM 404
TRIAE_CS42_5AL_TGACv LGLSKMNFVVSPKVSDEDESKRYEQEIMEFGSSDPEYVIIGTITLLNLVCLLGGLSKVMKVGWNNIHLDALFPQLILCGM 686
TRIAE_CS42_6DS_TGACv LGLSKMTFVVTPKVSIEDESKRYEEEIMEFGSSTPEYVIIATIALLNLVCLLGGLSQIMTGAWN-IHLDAFSPQLILCGM 683
TRIAE_CS42_5DL_TGACv LGLSKMTFAVTSKVSEESESKRYEQEIMEFGSSAPEYVIIATVALLNLNCLVVGLCQIMTGGWN-ILLNVFSPQLILCGM 686
TRIAE_CS42_5BL_TGACv LGLSKMTFAVTSKVSEENESKRYEQEIMEFGSSAPEYVIIATVALLNLICLVGGLSQILTGGWN-ILLNVFSPQLILCGM 686
TRIAE_CS42_6AL_TGACv LGVSESGFELTVKVDESQALERYKKGKMEFGPISGMFVIITTIALFNLVCLVVGLGRALLREGT-AGLGPLFLQAVLCVA 696
TRIAE_CS42_6BL_TGACv LGVSESGFELTVKVDESQALERYKKGKMEFGPISGMFVIITTIALFNLVCLVLGLGRVVLREGA-AGLGPLFLQAVLCVA 695
TRIAE_CS42_6DL_TGACv LGVSESGFELTVKVDESQALERYKKGKMEFGPISGMFVIITTIALFNLVCLVLGLGRVLLRGGA-EGLGPLFLQAVLCVA 697

TRIAE_CS42_5DL_TGACv LVITSIPFYEAMFLRKDKGRIPFPVTLASIGFVMLALLAKIV------ 756
TRIAE_CS42_5BL_TGACv VVITSIPFYEAMFLRKDKGRIPFPVTLASIGFVMLALLATIV------ 728
TRIAE_CS42_U_TGACv1_ VVITSIPFYEAMFLRKDKGRIPFPVTLASIGFVMLALLPAIV------ 446
TRIAE_CS42_5AL_TGACv VVITSIPFYEAMFLRKDKGRIPFPVTLASIGFVMLALLPAIV------ 728
TRIAE_CS42_6DS_TGACv LVITNMPFYEAMFLRNDKGKIPFTVTLASFGFVMLDFLVPIV------ 725
TRIAE_CS42_5DL_TGACv LVITNIPFYEAMFVRKDKGRIPFSVTLASIGFAILALLVPIV------ 728
TRIAE_CS42_5BL_TGACv LVITNIPFYEAMFVRKDKGRMPFSVTLASISFAMLALLVPIVYNSRYI 734
TRIAE_CS42_6AL_TGACv IVVINAPVYEALFIRRDSGSLPYFVTLVSLCFVSSLCLQAI------- 737
TRIAE_CS42_6BL_TGACv IVVINAPVYEALFIRRDSGSLPYFVTLVSLCFVSSLCLQAI------- 736
TRIAE_CS42_6DL_TGACv IVVINAPVYEALFIRRDSGGLPYFVTLVSLCFVSSLCLQAI------- 738
```

Fig. S_2E: *CslF* subfamily.

S.No	Gene name with number of splice variants (CslF)	No. of amino acids (aa)
1	TRIAE_CS42_2DL_TGACv1_159781_AA0542640.1	845 aa
2	TRIAE_CS42_7BL_TGACv1_580651_AA1914920.1	614 aa
3	TRIAE_CS42_7AL_TGACv1_557532_AA1782680.1	837 aa
4	TRIAE_CS42_7DL_TGACv1_602590_AA1961740.1	835 aa
5	TRIAE_CS42_2AL_TGACv1_094713_AA0301960.1	865 aa
6	TRIAE_CS42_2DL_TGACv1_160109_AA0546890.1	862 aa
7	TRIAE_CS42_2BL_TGACv1_130934_AA0420130.1	663 aa
8	TRIAE_CS42_2DS_TGACv1_178985_AA0603230.1	870 aa
9	TRIAE_CS42_2AS_TGACv1_112790_AA0345230.1	878 aa
10	TRIAE_CS42_2BS_TGACv1_148027_AA0489970.1	877 aa
11	TRIAE_CS42_2AS_TGACv1_113659_AA0359050.1	847 aa
12	TRIAE_CS42_2DS_TGACv1_177641_AA0581710.2_2_SPLICE	847 aa
13	TRIAE_CS42_2BS_TGACv1_148608_AA0494060.1	851 aa
14	TRIAE_CS42_U_TGACv1_641498_AA2096480.1	857 aa
15	TRIAE_CS42_2BS_TGACv1_146146_AA0456710.1	754 aa
16	TRIAE_CS42_2DS_TGACv1_179076_AA0604160.1_2_SPLICE	783 aa
17	TRIAE_CS42_2AS_TGACv1_112322_AA0335290.1	878 aa
18	TRIAE_CS42_2BS_TGACv1_147667_AA0486240.1_2_SPLICE	877 aa
19	TRIAE_CS42_2DS_TGACv1_177329_AA0573830.1	875 aa
20	TRIAE_CS42_2BS_TGACv1_148916_AA0495580.1	701 aa
21	TRIAE_CS42_2DS_TGACv1_178471_AA0596060.1	701 aa
22	TRIAE_CS42_2AS_TGACv1_112322_AA0335280.1	897 aa
23	TRIAE_CS42_5BL_TGACv1_409916_AA1366600.2_2_SPLICE	815 aa
24	TRIAE_CS42_5DL_TGACv1_433902_AA1424880.1	808 aa
25	TRIAE_CS42_5AL_TGACv1_374191_AA1193100.1_2_SPLICE	807 aa
26	TRIAE_CS42_7BL_TGACv1_577473_AA1876170.1	941 aa
27	TRIAE_CS42_7AL_TGACv1_555973_AA1751470.1	899 aa
28	TRIAE_CS42_7DL_TGACv1_607937_AA2011180.1	498 aa
29	TRIAE_CS42_1BS_TGACv1_049866_AA0163180.1	856 aa

```
Color Align Conservation results
TRIAE_CS42_5DL_TGACv -----------------------------------------------------------MSMTYITKKHDYAATLDEKEP 21
TRIAE_CS42_5AL_TGACv -----------------------------------------------------------MSMTYITKKHDYVASLDGKES 21
TRIAE_CS42_5BL_TGACv -----------------------------------------------------------MSMTYISKKHDYAATLDEKEQ 21
TRIAE_CS42_2AS_TGACv -----------MTTSPATHDGAATGLSEPLLPNRNGVHAGALVVTPVVANGHGGGDKLKGDLKAKDKYWKDVDQPDDVAA 69
TRIAE_CS42_2BS_TGACv -----------MTTSPATAAGAATGLSEPLLSNGNGVHAGALVVTPVVANGHGG-DKLKGDLKAKDKYWKDVDQPDDVAA 68
TRIAE_CS42_2DS_TGACv -----------MTTSPATDAGAATGLSEPLLSNRNGVHAGALVVTPVAANGHGG--------KAKDKYWKDVDQPGDMAV 61
TRIAE_CS42_2AS_TGACv ------------MASAAGAGGANAGLADPLLAS------------------------AKKPVGAKGKHWVAADK-DQRRA 43
TRIAE_CS42_2DS_TGACv ------------MASAAGAGGANAGLADPLLAS------------------------AKKPVGAKGKHWVAADK-DQRRA 43
TRIAE_CS42_2BS_TGACv ------------MASAVGAGGANAGLADPLLASRD--------------------GGAKKPVGAKGKHWVAADK-DQRRA 47
TRIAE_CS42_2DL_TGACv -------------------------------MAAAVTRRSNALRVDVPGGEAVAVSVAADSPVAKRGLGAKDDVWVAADE 49
TRIAE_CS42_2BL_TGACv -------------------------------------------------------------------------------- 0
TRIAE_CS42_2AL_TGACv ------------------------------MMAAAVTRRSNALRVDVPDGDAVAVSVVADSPVAKRGLGAKEDVWVAVDE 50
TRIAE_CS42_2DL_TGACv -------------------------------MAAAVTRRVNALRVEVPDG-----NADTANAPAAKRILDAKDDVWVSAD 44
TRIAE_CS42_2BS_TGACv --------------------------MAAAVTRRANALRVEAPDGNTESGRASLAADSPVAKRAVDAKDDVWVAADEGEA 54
TRIAE_CS42_2DS_TGACv --------------------------MAAAVTRRANALRAEAPDGNAESGRASLAADSPAAKRAVDAKDDVWVAADEGDT 54
TRIAE_CS42_7AL_TGACv --------------------------------------------MPLRVEALVATDTASAAAAEGRRAKDDVWVAEEGDM 36
TRIAE_CS42_7DL_TGACv --------------------------------------------MALRVEALVATDTAAAEGR--RAKDDVWVAAEEGDM 34
TRIAE_CS42_7BL_TGACv ----------------------------------------------------MATDTVADAAEGRRARDDVWVAAEEGDM 28
TRIAE_CS42_U_TGACv1_ ------------------------------MPSPAAVGGGRLADPLLAAD-------VVVGAKDKYWVPADEREILASQK 43
TRIAE_CS42_1BS_TGACv ------------------------------MASPAAGGGGRLADPLLATD-------VVVGPKDKYWVPADEREILASHR 43
TRIAE_CS42_2AS_TGACv --------------------------MVSPATGGGRGGNAGLAEPLLATNDDSDGAKHVFGAKAKHWVPADEKEMAASRE 54
TRIAE_CS42_2BS_TGACv --------------------------MVSPATSGGRGGNAGLADPLLATNDDSDGARHVFGAKAKYWAPADEKEMTASRE 54
TRIAE_CS42_2DS_TGACv ----------------------------MVSPAASGGGNAGLADPLLATNDNSEGARHVFGAKAKYWVPADEKEIAASRE 52
TRIAE_CS42_2BS_TGACv -------------------------------------------------------------------------------- 0
TRIAE_CS42_2DS_TGACv -------------------------------------------------------------------------------- 0
TRIAE_CS42_2AS_TGACv ----MGSLAAANGAGHASNGAGVADQALALENGTGNGHKAGVANRATPPLQANGGSKVAKKISPKDKYWVAADEGEMAAA 76
TRIAE_CS42_7AL_TGACv MAPAVAGGGRVRSNEPAAAAAPAAASGKPCVCGFQVCACTGSAAVASAASSLDMDIVAMGQIGAVNDESWVGVELGEDGE 80
TRIAE_CS42_7DL_TGACv -------------------------------------------------------------------------------- 0
TRIAE_CS42_7BL_TGACv MAPAVAGGGRVRSNEP----AAAAASDKPCVCGFQVCACTGSAAVASAASSLDMDIVAMGQIGAVNDESWVGVELGEDGE 76

TRIAE_CS42_5DL_TGACv SEDQKSASVKNLLVRTTKLTTVTIKLYRLMVFVRLTIFVLFFKWRVSTALTVISDGTTTARAMWTMSIAGELWFALMWVL 101
TRIAE_CS42_5AL_TGACv PEHEKSASVERLLVRTTKLTTVTIKLYRLVVFVRMIIFVLFFKWRSSTALAMISDGTTTVRAMWTMSIAGELWFALMWVL 101
```

```
TRIAE_CS42_5BL_TGACv PKDQKSASVESLLVRTTKLTTVTIKLYRIMVFVRMAIFVLFFKWRISTALAMISDGATTVRAMWTMPIAGELWFALMWVL 101
TRIAE_CS42_2AS_TGACv APDLENGGGRPLLFSNRRVKNIILYPYRVLILIRVIAVILFVGWRIK-------HNNSDVMWFWVMSVVADVWFSLSWLS 142
TRIAE_CS42_2BS_TGACv APDLENGGGRPLLFSNRRVKNIILYPYRVLILIRVIAVILFVGWRIK-------HNNSDVMWFWVMSVVADVWFSLSWLS 141
TRIAE_CS42_2DS_TGACv APDLENGGGRPLLFSNRRVKNIILYPYRVLILIRVIAVILFVGWRIK-------NNNSDVMWFWVISVVADVWFSLSWLS 134
TRIAE_CS42_2AS_TGACv AKESGGEDGRPLLFRTYKVKGTLLHPYRALIFIRLIAVLLFFVWRIK-------HNKSDVMWFWTMSVVGDVWFGFSWLL 116
TRIAE_CS42_2DS_TGACv AKESGGEDGRPLLFRTYKVKGTLLHPYRALIFIRLIAVLLFFVWRIK-------HNKSDIMWFWTLSVVGDVWFGFSWLL 116
TRIAE_CS42_2BS_TGACv AKESGGEEGRPLLFRTYKVKGTLLHPYRALIFIRLIAVLLFFVWRIK-------HNKSDIMWFWTMSVVGDVWFGFSWLL 120
TRIAE_CS42_2DL_TGACv GGIMSGDGNRPLLFRTMKVKGSILHPYRFLMLVRLVAVVAFFKWHVE-------HKNQDSVWLWTASMTADPWFGFSWLL 122
TRIAE_CS42_2BL_TGACv -------------------------------------------------------------------------------- 0
TRIAE_CS42_2AL_TGACv GG-MSGDGNRPLLFRTMKVKGSILHPYRFLMLMRLVAVVIFFKWRME-------HKNHDGVWLWTVSMTADVWFGFSWLL 122
TRIAE_CS42_2DL_TGACv DGTSAGNGNQPLLFRTMKVKGSILHPYRFLILVRLVAVAAFFAWRLE-------HRNHDGTWLWATSMVADAWFGFSWLL 117
TRIAE_CS42_2BS_TGACv SGSIAGDGNRTPLFRTFKVKGSILHPYRFMILVRLVAIVAFFAWRVK-------HKNHDGVWLWATSMVADVWFGFSWLL 127
TRIAE_CS42_2DS_TGACv SGAIAGDGNRPPLFRTFKVKGSILHPYRFMILVRLVAIVAFFAWRVK-------HKNHDGVWLWATSMVADVWFGFSWLL 127
TRIAE_CS42_7AL_TGACv SGASAG---RPLLFRTMKVKGSILHPYRFLILVRLVAIVAFFAWRVE-------HRNHDGTWLWATSMVADAWFGFSWLL 106
TRIAE_CS42_7DL_TGACv SGASAG---RPLLFRTMKVKGSILHPYRFLILVRLVAIIAFFAWRVE-------HRNHDGMWLWATSMVADAWFGFSWLL 104
TRIAE_CS42_7BL_TGACv PEASAG---RPLLFRTMKVKGSILHPYRFLILVRLVAIVAFFAWRVE-------HRNHDGVWLWATSMVADAWFGFSWLL 98
TRIAE_CS42_U_TGACv1_ SGAG-EDGRAPLLYRTFRVKGPLINLYRLLTLVRVIVVILFFTWRMR-------HRDSDAMWLWWISVVGDLWFGVTWLL 115
TRIAE_CS42_1BS_TGACv SGAGGDDGRAPLLYRTFRVKGPLINLYRLLTLVRVIVVILFFTWRMR-------HRDSDAMWLWWISVVGDLWFGVTWLL 116
TRIAE_CS42_2AS_TGACv CGGE---DGRPLLYRTFKVRGFLVNTYRFLNLARLTAVIVFFAWRVQ-------HPDSDAMWLWWISVVGDFWFGLSWWL 124
TRIAE_CS42_2BS_TGACv CSGE---DGRPLLYRTFKVKGFLVNTYRFLNLARLTAVIVFFAWRVQ-------HPDSDAMWLWWISVVGDFWFGLSWWL 124
TRIAE_CS42_2DS_TGACv CGGE---DGRPLLYRTFKVKGMLVNTYRFLNLARLTAVIVFFAWRVQ-------HPDSDAMWLWWISVVGDFWFGLSWWL 122
TRIAE_CS42_2BS_TGACv -------------------------------------------------------------------------------- 0
TRIAE_CS42_2DS_TGACv -------------------------------------------------------------------------------- 0
TRIAE_CS42_2AS_TGACv IADGGEDGRRPLLYRTFKVKGILLHPYRLLSLIRLVAIVLFFVWRVR-------HPYADGMWLWWISMVGDLWFGVTWLL 149
TRIAE_CS42_7AL_TGACv TDESGVAVDDRPVFRTEKIKGVLLHPYRVLIFVRLIAFTLFVIWRIS-------HKNPDAMWLWVTSICGEFWFGFSWLL 153
TRIAE_CS42_7DL_TGACv -------------------------------------------------------------------------------- 0
TRIAE_CS42_7BL_TGACv TDESGAAVDDRPVFRTEKIKGVLLHPYRVLIFVRLIAFTLFVIWRIS-------HKNPDAMWLWVTSICGEFWFGFSWLL 149

TRIAE_CS42_5DL_TGACv DQLPKMQPVRRTVYVTALE--------EPRLPTMDVFVTTTDPEKEPPLVTVNTILSILAADYPPDKLTCYVSDDGGALL 173
TRIAE_CS42_5AL_TGACv DQLPKMQPVRRTVYATALE--------ESLLPAMDVFVTTADPEKEPPLVTVNTILSILAADYPPDKLTCYVSDDGGALL 173
TRIAE_CS42_5BL_TGACv DQLPKMQPVRRTVFATALE--------EPLLPTMDVFVTTADPEKEPPLVTVNTILSILAADYPPDKLTCYVSDDGGALL 173
TRIAE_CS42_2AS_TGACv YQLPKYNPIKMIPDLATLRKQFDTPGRSSQLPGIDVIVTTASATDEPILYTMNGVLSILAADYHIGRCNCYLSDDSGSLV 222
TRIAE_CS42_2BS_TGACv YQLPKYNPIKMIPDLATLRKQFDTPGSSSQLPGIDVIVTTASATDEPILYTMNCVLSILAADYHIGRCNCYLSDDSGSLV 221
TRIAE_CS42_2DS_TGACv YQLPKYNPIKMIPDLATLRKQFDTPGRSSQLPGIDVIVTTASATDEPILYTMNCVLSILAADYHIGRCNCYLSDDSGSLV 214
TRIAE_CS42_2AS_TGACv NQLPKFNPVKTIPDMVALRRQYDLPDGTSTLPGIDVFVTTADPIDEPILYTMNCVLSILASDYPVDRCACYLSDDSGALI 196
TRIAE_CS42_2DS_TGACv NQLPKFNPVKTIPDMVALKRQYDLPDGTSTLPGIDVFVTTADPIDEPILYTMNCVLSILASDYPVDRCACYLSDDSGALI 196
TRIAE_CS42_2BS_TGACv NQLPKFNPVKTIPDMVALRRQYDLPDGTSTLPGIDVFVTTADPIDEPILYTMNCVLSILASDYPVDRCACYLSDDSGALI 200
TRIAE_CS42_2DL_TGACv NQLPKLNPIKRVP---DLADRHD----DATLPRIDVFVTTVDPVDEPVLYTVNTILSILAADYPIDNYACYISDDGGTLV 195
TRIAE_CS42_2BL_TGACv -------------------------------------------------------------------------------- 0
TRIAE_CS42_2AL_TGACv NQLPKLNPIKRVPDLAALADRHD----DATLPGIDVFVTTVDPVDEPVLYTVNTILSILAADYPVDNYACYLSDDGGTLV 198
TRIAE_CS42_2DL_TGACv NQLTKLNPIKRVPDLATLADQHG----EAILPGIDVFVTTADPVDEPVLYTVNTVLSILAADYPIDKYACYLSDDGGTLV 193
TRIAE_CS42_2BS_TGACv NQLPKLNPVKRVPDLAALADHSG----DANLPGIDIFVTTVDPVDEPLLYTVNTILSILATDYPVDKYACYLSDDGGTLV 203
TRIAE_CS42_2DS_TGACv NQLPKLNPVKRVPDLAALADHSG----DANLPGIDIFVTTVDPVDEPLLYTVNTILSILATDYPVDKYACYLSDDGGTLV 203
TRIAE_CS42_7AL_TGACv NQLPKLNPIKRVPDLVALADRHG----EAILPGIDVFVTTVDPVDEPVLYTVNTILSILAADYPVDKYACYLSDDGGTLV 182
TRIAE_CS42_7DL_TGACv NQLPKLNPIKRVPDLAALADLHG----EAVLPGIDVFVTTVDPVDEPVMYTVNTILSILAADYPVDKYACYLSDDGGSLV 180
TRIAE_CS42_7BL_TGACv NQLPKLNPIKRVPDLAALADRHG----EAVLPGIDVFVTTVDPVDEPVMYTVNTILSILAADYPVDKYACYLSDDGGTLV 174
TRIAE_CS42_U_TGACv1_ NQITKLRPRKCVPSISVLRDHLDQPDGGSDLPLLDVFINTVDPVDEPMLYTMNSILSILATDYPVEKYATYFSDDGGSLV 195
TRIAE_CS42_1BS_TGACv NQITKLRPRKCVPSISVLREQLDQPDGGSDLPLLDVFINTVDPVDEPMLYTMNSILSILATDYPVDKYATYFSDDGGSLV 196
TRIAE_CS42_2AS_TGACv NQVPKLNPTICIPTIPLLRQQFDLPDGGSNLPVLDVFISTVDPVEEPMLHTMNSILSILATDYPVDKYATYLSDDGGSLL 204
TRIAE_CS42_2BS_TGACv NQVPKLNPTICIPTIPLLRQQFDLPDGGSNLPVLDVFISTVDPVEEPMLHTMNSILSILATDYPVDKYATYLSDDGGSLL 204
TRIAE_CS42_2DS_TGACv NQVPKLNPTICIPTIPLLRQQFDLPDGGSNLPVLDVFISTVDPVEEPMLHTMNSILSILATDYPVDKYATYLSDDGGSLL 202
TRIAE_CS42_2BS_TGACv ----------------------------------------------MIYTMNSIISILAADYPVDKHACYLSDDGGSII 33
TRIAE_CS42_2DS_TGACv ----------------------------------------------MIYTMNSIISILAADYPVDKHACYLSDDGGSII 33
TRIAE_CS42_2AS_TGACv NQVAKLNPVKRVPNLTLLEQQFDLPDGNSNLPCLDVFINTVDPINEPMIYTMNSIISILAADYPVDKHACYLSDDGGSII 229
TRIAE_CS42_7AL_TGACv DQLPKLNPINRVPDLAVLRQRFDRPDGTSTLPGLDIFVTTADPIKEPILSTANSVLSILAADYPVDRNTCYVSDDSGMLL 233
TRIAE_CS42_7DL_TGACv -------------------------------------------------------------------------------- 0
TRIAE_CS42_7BL_TGACv DQLPKLNPINRVPDLAVLRQRFDRPDGTSTLPGLDIFVTTADPIKEPILSTANSVLSILAADYPVDRNTCYVSDDSGMLL 229

TRIAE_CS42_5DL_TGACv TREAVAHAACFARLWVPFCRKHGVEPRNPEAYFCPGVKARVVSRADYMGRSWPELARDRRRVRREYEELRLRIDALHAGD 253
TRIAE_CS42_5AL_TGACv TREAVAQAAWFARLWVPFCRKHGVEPRNPEAYFCPGVKARVVSRADYRAKSWPELARDRRRVRREYEELRLRIDALHAGD 253
TRIAE_CS42_5BL_TGACv TREAVAHAARFARLWVPFCRKHGVEPRNPEAYFCPGVKARVVSRAAYMGRSWPELARDRRRVRREYEELRLRIDALHAGD 253
TRIAE_CS42_2AS_TGACv LYEALVETAKFAALWVPFCRKHQIEPRAPESYFELEGT-------LCGGASHKEFIQDYKHVRTQYDEFKKHLDMLPNTI 295
TRIAE_CS42_2BS_TGACv LYEALVETAKFAALWVPFCRKHQTEPRAPARYFELEGP-------LCGGASHKEFIQDYKHVRMQYEEFKKHLDMLPNTI 294
TRIAE_CS42_2DS_TGACv LYEALVETAKFAALWVPFCRKHQIDPRAPESYFELEGP-------LCGGASHKEFIQDYKHVCTQYEEFKKHLDMLPNTI 287
TRIAE_CS42_2AS_TGACv QYEALVETAKFATLWVPFCRKHCIEPRAPESFFEQEAP-------LYTGSAPEEFKNDHNSVYIEYDEFKECLDSLSSAI 269
TRIAE_CS42_2DS_TGACv QYEALVETAKFATLWVPFCRKHCIEPRAPESYFELEAP-------LYTGSAPEDFKNDHSSVHREYDEFKEHLDSISSAI 269
TRIAE_CS42_2BS_TGACv QYEALIETAKFATLWVPFCRKHCIEPRAPESYFELEAP-------LYTGSASEEFKNDHSSVHREYDEFKEHLDSLSSAI 273
TRIAE_CS42_2DL_TGACv HYEAMVQVASFAALWVPFCRKHCVEPRSPESYFGIKTR-------SYIGGMAGEFMRDHRRVRREYEEFKVRIDSLSTTI 268
TRIAE_CS42_2BL_TGACv ----MVQVASFAALWVLFCRKHCVEPRSPESYFGMKTR-------SYAGGMAGEFMRDHRRVRREYEEFKVRIDSLSTTI 69
TRIAE_CS42_2AL_TGACv HYEAMVQVASFAALWVPFCRKHCVEPRSPESYFGIKTH-------SYAGGMAGEFMRDRRRVRREYEEFKVRIDSLSTTI 271
TRIAE_CS42_2DL_TGACv HYEAMTQVASFAALWAPFCRKHCVEPRSPENYFGMKAQ-------PYAGSMPGDFTRDRRRVRREYDEFMVRIDSLSTTI 266
TRIAE_CS42_2BS_TGACv HYEAMIEVANFAVLWVPFCRKYCVEPRSPENYFGMKTQ-------PYAGSMAGEFMRDHRRVRREYDELKVRVDSLSTTI 276
TRIAE_CS42_2DS_TGACv HYEAMIEVANFAVLWVPFCRKYCVEPRSPENYFGMKTQ-------PYAGSMAGEFMRDHRRVRREYDEFKVRVDSLSTTI 276
TRIAE_CS42_7AL_TGACv HYEAMLQVASFAALWVPFCRKHCVEPRSPENYFGMKTR-------PYVGGMAGEFMSDHRRVRREYGEFKVRIDSLSTTI 255
TRIAE_CS42_7DL_TGACv HYEAMIQIVHFAALWVPFCRKHCIEPRSPENYFGMKTR-------PYVGGMAGEFMSDHRRVRREYGEFKVIIDSLSTTI 253
TRIAE_CS42_7BL_TGACv HYEAMLQVASFAALWVPFCRKHCVEPRSPENYFGMKTR-------PYVGGMAGEFMSDHRRVRREYGEFKVRIDSLSSTI 247
TRIAE_CS42_U_TGACv1_ HYEGLQLAAEFAASWVPFCRKHCVEPRAPESYFWAKMRG------EYAGSAPKEFLDDHRRMRAAYEEFKARLDGLSAAI 269
TRIAE_CS42_1BS_TGACv HYEGLQLAAEFAASWVPFCRKHCVEPRAPESYFWAKMRG------EYAGTAPKEFLDDHRRMRAAYEEFKVRLDGLSAAI 270
TRIAE_CS42_2AS_TGACv HYDGLVETAKFAALWVPFCRKHHVEPRAPESYFGMKVR-------PYKGNLPEEFLDDHRRLRREYEEFKTRLDALFTVI 277
TRIAE_CS42_2BS_TGACv HYDGLVETAKFAALWVPFCRKHHVEPRAPESYFGMKIR-------PYTGNLPEEFLDDHRRLRREYEEFKTRLDALFTVI 277
TRIAE_CS42_2DS_TGACv HYDGLVETAKFAALWVPFCRKHHVEPRAPESYFGVKIR-------PYMGNLPEEFLDDHGRLRREYEEFKTRLDALFTLI 275
```

```
TRIAE_CS42_2BS_TGACv HYDGLLETAKFAALWVPFCRKHSIEPRAPESYFSLNTR-------PYTGNAPQDFVNDRRHMCREYDEFKERLDALFTLI 106
TRIAE_CS42_2DS_TGACv HYDGLLETAKFAALWVPFCRKHSIEPRAPESYFSLNTR-------PYTGNAPQDFVNDRRHMCREYDEFKERLDALFTLI 106
TRIAE_CS42_2AS_TGACv HYDGLLETAKFAALWVPFCRKHSIEPRAPESYFSLNTR-------PYTGNAPQDFVNDRRHMCREYDEFKERLDALFTLI 302
TRIAE_CS42_7AL_TGACv TYEALAESSKFATLWVPFCRKHGIEPRGPESYFELKSHP-------YMGRAQDEFVNDRRRVRKEYDEFKARINSLEHDI 306
TRIAE_CS42_7DL_TGACv -------------------------------------------------------------------------------- 0
TRIAE_CS42_7BL_TGACv TYEALAESSKFATLWVPFCRKHGIEPRGPESYFELKSHP-------YMGRAQDEFVNDRRRVRKEYDEFKARINSLEHDI 302

TRIAE_CS42_5DL_TGACv VRPQ---------------------------------QWSRGTAENHAGVVEVLVGPPGSTPELG-----VSDLLDLSSV 295
TRIAE_CS42_5AL_TGACv VRRQ---------------------------------QWSRGTAEDHAGVVEVLVGPPGSTPELG-----VSDLLDLGSV 295
TRIAE_CS42_5BL_TGACv VRRQ---------------------------------PWSRGTPEYHAGVVEVLVGPPGSTPELG-----VSDLLDLTSV 295
TRIAE_CS42_2AS_TGACv RQRSDIYSRTGTK--DEDATVTWMADG-TQWPGTWLDPTEKHRPGHHAGIVKIVQSHPEHVVPLG-VQESNDNPLNFDDV 371
TRIAE_CS42_2BS_TGACv RQRSDIYSKTGTK--DEDAKVTWMADG-TQWPGTWVDPAEKHRAGHHAGIVKIVQSHPEHVVPLG-VQESNDNPLNFDDV 370
TRIAE_CS42_2DS_TGACv RQRADIYSKTGTK--DEDAKVTWMADG-TQWPGTWLDPAEKHRAGHHAGIVKIVQSHPEHVVPLG-VHESNDSSLNFDGV 363
TRIAE_CS42_2AS_TGACv SKRSDAYNSMKTE--EGDANATWMANG-TQWPGSWIDTTEIHRKGHHAGIVKVVLDHSIRGHNLG-SQASTHN-LNFAST 344
TRIAE_CS42_2DS_TGACv SKRSDAYNSMKTE--EGDAKATWMANG-TQWPGSWIDTTEIHRKGHHAGIVKVVLGHSIRGHNLG-SQASTNN-LNFAST 344
TRIAE_CS42_2BS_TGACv SKRSDAYNSMKTG--EGDAKATWMANG-TQWPGSWIDTTEIHRKGHHAGIVKVVLDHSVRGHNLG-SQASTHN-LNFANT 348
TRIAE_CS42_2DL_TGACv RQRS---DAYNS-SNKGGVSATWMADG-THWPGTWVEQAENHRRGQHAGIVQVLLDHPSCKPQLGSPASTD-NPFDFSNI 342
TRIAE_CS42_2BL_TGACv RQRS---DAYNS-NNKGGVSATWMADG-TQWPGTWVEQAENHRRGQHAGIVQVLLDHPSFKPQLGSPASTD-NPFDFSNV 143
TRIAE_CS42_2AL_TGACv RQRS---DAYNS-KNKGGVSATWMADG-TQWPGTWVEQAENHRRGQHAGIVQVLLDHPSCEPQLGSPASTD-NPFDFSNV 345
TRIAE_CS42_2DL_TGACv RQRS---DAYN---NGDGVHATRMADG-APWPGTWIEQAENHRRAQHAGIVQVILEHPGCKPQLGSSASTD-NPFDFNNV 338
TRIAE_CS42_2BS_TGACv RQRS---DAYNSSTKGDGVRATWMADG-TQWPGTWIEQVENHRRGQHAGIVQVILGHPSCKPQLGSPASSD-NPLDFSNV 351
TRIAE_CS42_2DS_TGACv RQRS---DAYNSSKKGDGVRATWMADG-TQWPGTWIEQVENHRRGQHAGIVQVILGHPSCKPQLGSPASAD-NPLDFSNV 351
TRIAE_CS42_7AL_TGACv RRRS---DAYN--KGDDGVHATWMADG-TQWAGTWIEQADNHRRGHHAGIVQVMLDHPSCKPQLGSSVSTN-SPIDLSNV 328
TRIAE_CS42_7DL_TGACv RRRS---DAYN--KRDDGVHATWMADG-TQWAGTWIEQADNHRRGQHAGIVQVMLDHPSCKPQLGSSARTN-NPIDLSNV 326
TRIAE_CS42_7BL_TGACv RRRS---DAYN--KGDDDVHATWMADG-TQWPGTWIEQADNHRRGQHAGIVKVMLDHPSCKPQLGSSASTN-KPVDLSNV 320
TRIAE_CS42_U_TGACv1_ EQRSEACNRANGKDKEECANATWMADGSTQWQGTWIKPAKGHRKGHHPAILQVMLDQPSKDPELGMAASSD-HPLDFSAV 348
TRIAE_CS42_1BS_TGACv EQRSEACNRANG--KEEGADATWMADGSTQWQGTWIKPAKGHRKGHHPAILQVMLDQPSKDPELGMAASSG-HPLDLSAV 347
TRIAE_CS42_2AS_TGACv PQRSEAHGREDAK-GGGGAKATWMADG-TQWPGTWTEPAEGHRKGDHAGIIQVMLSQPSGEPQLGAPASSDDNPLDFSAV 355
TRIAE_CS42_2BS_TGACv PQRSEAHGREDAK-GGG-GKATWMADG-TQWPGTWTEPAEGHRKGDHAGIIQVMLSQPSSEPQLGEPASSDDGPLDFSAV 354
TRIAE_CS42_2DS_TGACv PQRSEAHGREDAK-GGG-GKATWMADG-TQWPGTWTEPAEGHRKGDHAGIIQVMLSQPSSEPQLGEPASSDHSPLDFSAV 352
TRIAE_CS42_2BS_TGACv PKRSDVYNHAAAK---EGAKATWMADG-TQWPGTWIDPAENHKKGQHVGIVKVMLKHPSYEPELGLGASTN-SPLDFSAV 181
TRIAE_CS42_2DS_TGACv PKRSDVYNHAAAK---EGAKATWMADG-TQWPGTWIDPAENHKKGQHVGIVKVMLKHPSYEPELGLGASTN-SPLDFSAV 181
TRIAE_CS42_2AS_TGACv PKRSDVYNHAAAK---EGAKATWMADG-TQWPGTWIDPAENHKKGQHVGIVKVMLKHPSYEPELGLGASTN-SPLDFSAI 377
TRIAE_CS42_7AL_TGACv KQRNDGYNAANAH-REGEPRPTWMADG-TQWQGTWVDASENHRRGDHAGIVLVLLNHPSHRRQTGPPASAD-NPLDFSGV 383
TRIAE_CS42_7DL_TGACv -------------------------------------------------------------------------------- 0
TRIAE_CS42_7BL_TGACv KQRNDGYNAANAH-REGEPRPTWMADG-TQWEGTWVDASENHRRGDHAGIVRVLLNHPSHRRQTGPPASAD-NPLDFSGV 379

TRIAE_CS42_5DL_TGACv DVRVPAVVYMCREKRHGRVHHRKAGAMNALLRTSAVLSNAPFILNLDCDHYVNNSQALRAGVCLMLD-RGGSNVAFVQFP 374
TRIAE_CS42_5AL_TGACv DVRVPAVVYMCREKRHGRVHHRKAGAMNALLRTSAVLSNAPFILNLDCDHYVNNSQALRAGVCLMLD-RGGSNVAFVQFP 374
TRIAE_CS42_5BL_TGACv DVRVPAVVYMCREKRHGRVHHRKAGAMNALLRTSAVLSNAPFILNLDCDHYVSNSQALRAGVCLMLD-RGGSNVAFVQFP 374
TRIAE_CS42_2AS_TGACv DMRLPMLVYVAREKSPGVEHNKKAGALNAELRISALLSNAPFFINFDCDHYINNSEALRAAICFMLDPREGDNTGFVQFP 451
TRIAE_CS42_2BS_TGACv DMRLPMLVYVAREKSPGVEHNKKAGALNAELRISALLSNAPFFINFDCDHYINNSEALRAAVCFMLDPREGDNTGFVQFP 450
TRIAE_CS42_2DS_TGACv DMRLPMLVYVAREKCPGVEHNKKAGALNAELRISALLSNAPFFINFDCDHYINNSEALHAAVCFMLDPREGDNTGFVQFP 443
TRIAE_CS42_2AS_TGACv DVRLPMLVYISRGKNPSYDHNKKAGALNAQLRASALLSNAQFIINFDCDHYINNSQALRAAMCFMLDQRQGDSTAFVQFP 424
TRIAE_CS42_2DS_TGACv DVRLPMLVYISRGKNPSYDHNKKAGALNAQLRASALLSNAQFIINFDCDHYINNSQALRAAMCFMLDQRQGDSTAFVQFP 424
TRIAE_CS42_2BS_TGACv DVRLPMLVYISRGKNPSYDHNKKAGALNAQLRASALLSNAQFIINFDCDHYINNSQALRAAMCFMLDQRQGDSTAFVQFP 428
TRIAE_CS42_2DL_TGACv DTRLPMLVYISREKRPGYDNQKKAGAMNVMLRVSVLLSNAPFVINFDCDHYINNSQALRAPMCFMLDPHDGQNTAFVQFP 422
TRIAE_CS42_2BL_TGACv DTRLPMLVYISREKRPGYDNQKKAGAMNVMLRVSALLSNAPFVINFDCDHYINNSQALRAPMCFMLDPHDGQNTAFVQFP 223
TRIAE_CS42_2AL_TGACv DTRLPMLVYISREKRPGYDNQKKAGAMNVMLRVSALLSNAPFVINFDCDHYINNSQALRAPMCFMLDPRDGQNTAFVQFP 425
TRIAE_CS42_2DL_TGACv DMRLPMLVYISREKRLGYDNQKKAGAMNAMLRISALLSNAPFIINFDCDHYINNSKALRAPMCFMLDPRDGQNTAFVQFP 418
TRIAE_CS42_2BS_TGACv DTRLPMLVYMSREKRPGYNHQKKAGAMNVMLRVSALLSNAPFVVNFDGDHYINNSQALCAPMCFMLDPRDGQNTAFVQFP 431
TRIAE_CS42_2DS_TGACv DTRLPMLVYMSREKRPGYNHQKKAGAMNVMLRVSAMLSNAPFVVNFDGDHYINNSQALRAPMCFMLDPRDGQNTAFVQFP 431
TRIAE_CS42_7AL_TGACv DTRLPMLVYISREKHPGYDNQKKAGAMNVMLRVSALLSNAPFVINFDCDHYINNSRALRAPMCFMLDPRDGQNTAFVQFP 408
TRIAE_CS42_7DL_TGACv DTRLPMLVYISREKHPGYDNQKKAGAMNVMLRVSALLSNAPFVINFDCDHYINNSQALRAPMCFMLDPRDGQNTAFVQFP 406
TRIAE_CS42_7BL_TGACv DTRLPMLVYISREKRPGYDNQKKAGAMNVMLRVSALLSNAPFVINFDCDHYINNSQALRAPMCFMLDPRDGQNTAFVQFP 400
TRIAE_CS42_U_TGACv1_ DARLPMLVYIAREKRPGYDHQKKAGAMNVQLRVSALLSNAPFIINFDGDHYVNNSQAFRAAICFMLDPRDGADTAFVQFP 428
TRIAE_CS42_1BS_TGACv DARLPMLVYIAREKRPGYDHQKKAGAMNVQLRVSALLSNAPFIINFDGDHYVNNSQAFRAAMCFMLDPRDGADTAFVQFP 427
TRIAE_CS42_2AS_TGACv DVRLPMLVYVSREKRPGYDHQKKAGALNVQLRVSALLSNAPFIINFDCDHYINNSQAFRAAMCFMMDRRDGDNVAFVQFP 435
TRIAE_CS42_2BS_TGACv DVRLPMLVYVSREKRPGYDHQKKAGALNVQLRVSALLSNAPFIINFDCDHYINNSQAFRAAMCFMMDRRDGDNVAFVQFP 434
TRIAE_CS42_2DS_TGACv DVRLPMLVYVSREKRPGYDHQKKAGALNVQLRVSALLSNAPFIINFDCDHYINNSQAFRAAMCFMMDRRDGDNVAFVQFP 432
TRIAE_CS42_2BS_TGACv DVRLPMLVYISREKSPSCDHQKKAGAMNVQLRVSALLTNAPFIINFDGDHYVNNSKAFRAGICFMLDRREGDNTAFVQFP 261
TRIAE_CS42_2DS_TGACv DVRLPMLVYISREKSPSCDHQKKAGAMNVQLRVSALLTNAPFIINFDGDHYVNNSKAFRAGICFMLDRREGDNTAFVQFP 261
TRIAE_CS42_2AS_TGACv DVRLPMLVYISREKSPSCDHQKKAGAMNVQLRVSALLTNAPFIINFDGDHYVNNSKAFRAGICFMLDRREGDNTAFVQFP 457
TRIAE_CS42_7AL_TGACv DVRLPMLVYVXXXXX---------------------------------------------XXXXXX-XXXXXXXXXXX 416
TRIAE_CS42_7DL_TGACv ----------------------------------------------------------------MVG-RDSDTVAFVQFP 15
TRIAE_CS42_7BL_TGACv DARLPMLVYVSREKRPGHDHQKKAGAMNALTRASALLSNSPFILNLDCDHYINNSQALRAGICFMVG-RDSDTVAFVQFP 458

TRIAE_CS42_5DL_TGACv QRFDGVDPADRYANHNRVFFDCTELGLDGLQGPIYVGTGCMFRRAALYNADPPLWRPHGGDRDAGK-------------- 440
TRIAE_CS42_5AL_TGACv QRFDGVDPADRYANHNRVFFDCTELGLDGLQGPIYVGTGCMFRRAALYNADPPLWRPHG-DRDAGK-------------- 439
TRIAE_CS42_5BL_TGACv QRFDGVDPADRYANHNRVFFDCTELGLDGLQGPIYVGTGCMFRRAALYNADPPLWRPHGGDRDAGK-------------- 440
TRIAE_CS42_2AS_TGACv QRFDNVDPTDRYGNHNRVFFDGAMYGLNGQQGPTYLGTGCMFRRLALYGIDPPCWRDEDIIVDSN--------------- 516
TRIAE_CS42_2BS_TGACv QRFDNVDPTDRYGNHNRVFFDGAMYGLNGQQGPTYLGTGCMFRRLALYGIDPPCWRAEDMIVDSN--------------- 515
TRIAE_CS42_2DS_TGACv QRFDNVDPTDRYGNHNRVFFDGAMYGLNGQQGPTYLGTGCMFRRLALYGIDPPCWRAEDIIVDSN--------------- 508
TRIAE_CS42_2AS_TGACv QRFDNVDPSDRYGNHNRVFFDGTMLALNGLQGPSYLGTGCMFRRIALYGIDPPEWRHANIVVDDK--------------- 489
TRIAE_CS42_2DS_TGACv QRFDNVDPSDRYGNHNRVFFDGTMLALNGLQGPSYLGTGCMFRRIALYGIDPPEWRHDNIVVDDK--------------- 489
TRIAE_CS42_2BS_TGACv QRFDNVDPSDRYGNHNRVFFDGTMLALNGLQGPSYLGTGCMFRRIALYGIDPPEWRHDNIVVDDK--------------- 493
TRIAE_CS42_2DL_TGACv QRFDDVDPTDRYANHNRVFFDGTMLALNGLQGPTYLGTGTMFRRVSLYGIEPPRYRAENTKLVRK--------------- 487
TRIAE_CS42_2BL_TGACv QRFDDVDPTDRYANHNRVFFDGTMLALNGLQGPTYLGTGTMFRRVALYGIEPPHYRAENTKLVCK--------------- 288
TRIAE_CS42_2AL_TGACv QRFDDVDPTDRYANHNRVFFDGTMLALNGLQGPTYLGTGTMFRRVSLYGIEPPRYRAENTKLVRK--------------- 490
TRIAE_CS42_2DL_TGACv QRFDDVDPTDRYANHNRVFFDGTMLALNGLQGPSYLGTGTMFRRVTLYGMEPPRYRVENIKLVDN--------------- 483
TRIAE_CS42_2BS_TGACv QRFDDVDPTDRYANHNRVFFDGTMLSLNGLQGPSYLGTGTMFRRVALYGMEPPRYRAENIKLAGK--------------- 496
```

```
TRIAE_CS42_2DS_TGACv QRFDDVDPTDRYANHNRVFTDGTMLSLNGLQGPSYLGTGTMFRRVALYGMEPPRYRAENIKLAGK--------------- 496
TRIAE_CS42_7AL_TGACv QRFDNVDPTDRYSNHNRVFTDGTMLSLNGLQGPTYLGTGTMFRRVALYGMEPPRYKAENIKLVGK--------------- 473
TRIAE_CS42_7DL_TGACv QRFDDVDPTDRYSNHNRVFTDGTMLSLNGLQGPTYLGTGTMFRRVALYGMEPPCYRAENIKLVGK--------------- 471
TRIAE_CS42_7BL_TGACv QRFDDVDPTDRYSNHNRVFTDGTMLSLNGLQGPTYLGTGTMFHRVALYGMEPQRYRAENIKLVGK--------------- 465
TRIAE_CS42_U_TGACv1_ QRFDDVDPTDRYCNHNRMFTDATLLGLNGIQGPSFVGTGCMFRRVALYSADPPRWRPDDAKEAKAS-------------- 494
TRIAE_CS42_1BS_TGACv QRFDDVDPTDRYCNHNRMFTDATLLGLNGIQGPSFVGTGCMFRRVALYSADPPRWRPDDAKEAKAS-------------- 493
TRIAE_CS42_2AS_TGACv QRFDDVDPTDRYANHNRMFTDATMLGMNGIQGPSYVGTGSMFRRVALYGADPPRWRPDDVKVLEN--------------- 500
TRIAE_CS42_2BS_TGACv QRFDDVDPTDRYANHNRMFTDATMLGMNGIQGPSYVGTGSMFRRVALYGADPPRWRPDDVKVLEN--------------- 499
TRIAE_CS42_2DS_TGACv QRFDDVDPTDRYANHNRMFTDATMLGMNGIQGPSYVGTGSMFRRVALYGADPPRWRPDDVKVLEN--------------- 497
TRIAE_CS42_2BS_TGACv QRFDDVDPTDRYCNHNRVFTDATLLGLNGIQGPSYVGTGCMFRRVALYGVDPPRWRPDDVKIVDS--------------- 326
TRIAE_CS42_2DS_TGACv QRFDDVDPTDRYCNHNRVFTDATLLGLNGIQGPSYVGTGCMFRRVALYGVDPPRWRPDDVKIVDS--------------- 326
TRIAE_CS42_2AS_TGACv QRFDDVDPTDRYCNHNRVFTDATLLGLNGIQGPSYVGTGCMFRRVALYGVDPPRWRPDNVKIVDS--------------- 522
TRIAE_CS42_7AL_TGACv XXXXXXXXXXXYANHNRIFTDGTLRALDGMQGPIYVGTGCLFRRITVYGFDPPRINVGGPCFPRLAGLFAKTKYEKPGLE 496
TRIAE_CS42_7DL_TGACv QRFEGVDPTDLYANHNRIFTDGTLRALDGMQGPIYVGTGCLFRRITVYGFDPPRINVGGPCFPRLAGLFAKTKYEKPGLE 95
TRIAE_CS42_7BL_TGACv QRFEGVDPTDLYANHNRIFTDGTLRALDGMQGPIYVGTGCLFRRITVYGFDPPRINVGGPCFPRLAGLFAKTKYEKPSLE 538

TRIAE_CS42_5DL_TGACv ------------------DVATEADKFGISTPFLGSVRAALGLNRSEQWNTTTKPPRSFDGAAVGEATALVSCGYEDRTA 502
TRIAE_CS42_5AL_TGACv ------------------DVAAEADKFGISTPFLGSVRAALNLNQSEQWNTTS-PPRSFDGAAVGEATALVSCGYEDRTA 500
TRIAE_CS42_5BL_TGACv ------------------DVATEADKFGISTPFLVSVRAALNLNRSEQWNTTS-PPRSFDGAAVGEATALVSCGYEDRTA 501
TRIAE_CS42_2AS_TGACv -------------------------RFGNSLLFLNSVLAAIKQEEG------VTLQPPLDDSFLEEVTKVVSSSYDDSTD 565
TRIAE_CS42_2BS_TGACv -------------------------RFGNSLPFLNSVLAAIKQEEG------VTLPPPLDDSFLEEMTKVVSSSYDDSTD 564
TRIAE_CS42_2DS_TGACv -------------------------RFGNSLPFLNSVLAAIKQEEG------VTLPPTLDDSFLEEMTKVVSSSYDDSTD 557
TRIAE_CS42_2AS_TGACv -------------------------RFGSSIPFLESVSKAINQER-------STIPPPISETLVAEMERVVSASHDKATG 537
TRIAE_CS42_2DS_TGACv -------------------------RFGSSIPFLESVSKAINQER-------STIPPPISETLVAEMERVVSASHDKATG 537
TRIAE_CS42_2BS_TGACv -------------------------RFGSSIPFLDSVSKAINQER-------STIPPPISETLVAEMERVVSASHDKATG 541
TRIAE_CS42_2DL_TGACv -----------------------TGEFGYSTSFVNSVPDAAIQDR-------SITPVLVDEHLRKDLATLMTCAYEDGSS 537
TRIAE_CS42_2BL_TGACv -----------------------TGEFGYSTSFINSVPDAAIQDR-------SITPVLVDERLSKDLATLMTCAYEDGSS 338
TRIAE_CS42_2AL_TGACv -----------------------AGEFGYSTSFVNSVPDAAIQDR-------SITPVLVDEGLRKDLTTLMTCAYEDGSS 540
TRIAE_CS42_2DL_TGACv -----------------------AHEFGNSTSFTNSMPDGAIQER-------SITPVLVDEGLINDLATLITCAYEDGSS 533
TRIAE_CS42_2BS_TGACv -----------------------VNEFGSSTSFINSMPDGAIQER-------SITPVLVDEALSNDLATLMTCAYEDGSS 546
TRIAE_CS42_2DS_TGACv -----------------------VNEFGSSTSFINSMPDGAIQER-------SITPVLVDEALSNDLATLMTCAYEDGSS 546
TRIAE_CS42_7AL_TGACv -----------------------AAELGNSTPFLKSIPDGAIQER-------SITPVLVDEALTSDLATLMTCAYEDRTS 523
TRIAE_CS42_7DL_TGACv -----------------------AAELGNSTPFLNSIPDGAIQER-------SITPVLVDEGLSNDIATLMTCTYEDGSS 521
TRIAE_CS42_7BL_TGACv -----------------------GAELGKSTPFLNSIPDGAIQDR-------SITPVSVDEGLMSDLATLMTCAYEDRTS 515
TRIAE_CS42_U_TGACv1_ --------------------RYRPNMFGKSTSFINSMPAAANQERS------VPSPATVGE---AELADAMTCAYEDGTE 545
TRIAE_CS42_1BS_TGACv --------------------RYRPNMFGKSTSFINSVPAAANQERS------VPSPATVGE---AELADAMTCAYEDGTE 544
TRIAE_CS42_2AS_TGACv -----------------------PNKFGKSMTFINSIPVAANQERS------VMSPVSLDEPATTELADVMTCAYEDGTE 551
TRIAE_CS42_2BS_TGACv -----------------------PNKFGKSMTFINSIPVAANQERS------VMSPVSLDEPATTELADVMTCAYEDGTE 550
TRIAE_CS42_2DS_TGACv -----------------------PNKFGKSMTFINSIPVAANQERS------VMSPVSLDEPATTELADVMTCAYEDGTE 548
TRIAE_CS42_2BS_TGACv -----------------------STKFGSSASFISSILPAADQERS------IMSPPALEEPVMADLAHVMTCAYEDGTE 377
TRIAE_CS42_2DS_TGACv -----------------------STKFGSSASFISSILPAADQERS------IMSPPALEESVMADLAHVMTCAYEDGTE 377
TRIAE_CS42_2AS_TGACv -----------------------STKFGSSASFISSILPAADQERS------IMSPPALEEPVMADLAHVMTCAYEDGTE 573
TRIAE_CS42_7AL_TGACv MTMAKAKAAPVPAKGKHGFLPLPKKTYGKSDAFVDSIPRASHPSPY----AAAAEGIVADEATIVEAVNVTAAAFEKKTG 572
TRIAE_CS42_7DL_TGACv MTMAKAKAAPVPAKGKHGFLPLPKKTYGKSDAFVDSIPRASHPSPY----AAAAEGIVADEATIVEAVNVTAAAFEKKTG 171
TRIAE_CS42_7BL_TGACv MTMAKAKAAPVPAKGKHGFLPLPKKTYGKSDAFVDSIPRASHPSPY----AAAAEGIVADEATIVEAVNVTAAAFEKKTG 614

TRIAE_CS42_5DL_TGACv WGRDIGWIYGTVTEDVATGFCMHRRGWRSAYCATAPDAFRGTAPINLTDRLHQVLRWAAGSLEIFFSRNNALLAGARLHP 582
TRIAE_CS42_5AL_TGACv WGRDIGWIYGTVTEDVATGFCMHRRGWSSAYCATAPDAFRGTAPINLTDRLHQVLRWAAGSLEIFFSRNNALLAGPRLHP 580
TRIAE_CS42_5BL_TGACv WGRDIGWIYGTVTEDVATGFCMHRRGWRSAYCATAPDAFRGTAPINLTDRLHQVLRWAAGSLEIFFSRNNALLAGARLHP 581
TRIAE_CS42_2AS_TGACv WGRGIGYIYNMATEDIVTGFRIHGQGWRSMYATMEREAFRGTAPINLTERLRQIVRWSGGSLEMFFSHISPLFAGRRLSL 645
TRIAE_CS42_2BS_TGACv WGRGIGYLYNMATEDIVTGFRIHGQGWRSMYVTMEREAFRGTAPINLTERLRQIVRWSGGSLEMFFSHISPLFAGRRLSL 644
TRIAE_CS42_2DS_TGACv WGRGIGYIYNMATEDIVTGFRIHGQGWRSMYATMEREAFRGTAPINLTERLRQIVRWSGGSLEMFFSHISPLFAGRRLSL 637
TRIAE_CS42_2AS_TGACv WGKGVGYIYDIATEDIVTGFRIHGQGWRSMYCTMERDAFCGIAPINLTERLHQIVRWSGGSLEMFFSLNNPLIGGRRIQS 617
TRIAE_CS42_2DS_TGACv WGKGVGYIYDIATEDIVTGFRIHGQGWRSMYCTMERDAFCGIAPINLTERLHQIVRWSGGSLEMFFSLNNPLIGGRRIQA 617
TRIAE_CS42_2BS_TGACv WGKGVGYIYDIATEDIVTGFRIHGQGWRSMYCTMERDAFCGIAPINLTERLHQIVRWSGGSLEMFFSLNNPLIGGRRIQS 621
TRIAE_CS42_2DL_TGACv WGRDAGWVYNIATEDVVTGFRIHRQGWRSMYCSMEPAAFRGTAPINLTERLYQVLRWSGGSLEVFFSHSNALIASRRLHP 617
TRIAE_CS42_2BL_TGACv WGRDAGWVYNIATEDVVTGFRIHRQGWHSMYCSMEPAAFRGTAPINLTERLYQVLRWSGGSLEVFFSHNNALIASRRLHP 418
TRIAE_CS42_2AL_TGACv WGRDAGWVYNIATEDVVTGFRIHRQGWRSMYCSMEPAAFRGTAPINLTERLYQVLRWSGGSLEVFFSHSNALIASRRLHL 620
TRIAE_CS42_2DL_TGACv WGRDIGWVYNIATEDVVTGFRIHRQGWRSMYCSMEPAAFRGTAPINLTERLYQVLRWSGGSLEVFFSHNNALIAGRRLHP 613
TRIAE_CS42_2BS_TGACv WGRDVGWVYNIATEDVVTGFRMHRQGWRSMYCSMEPAAFRGTAPINLTERLYQVLRWSGGSLEMFFSHSNALMAGRRLHP 626
TRIAE_CS42_2DS_TGACv WGRDVGWVYNIATEDVVTGFRMHRQGWRSMYCSMEPAAFRGTAPINLTERLYQVLRWSGGSLEMFFSHSNALMAGRRLHP 626
TRIAE_CS42_7AL_TGACv WGRDVGWVYNIATEDVVTGFRIHRQGWRSMYCSMEPAAFRGTAPINLTERLYQVLRWSGGSLEAFFSHSNALIASRRLHP 603
TRIAE_CS42_7DL_TGACv WGRDVGWVYNIATEDVVTGFRIHRQGWRSMYCSMEPAAFRGTAPINLTERLYQVLRWSGGSLEVFFSHSNALIASRRLNP 601
TRIAE_CS42_7BL_TGACv WGRDVGWVYNIATEDVVTGFRIHRQGWRSMYCSMEPAAFRGTAPINLTERLYQVLGG----------------------- 572
TRIAE_CS42_U_TGACv1_ WGNDVGWVYNIATEDVVTGFRLHRTGWRSTYCAMEPDAFRGTAPINLTERLYQILRWSGGSLEMFFSRFCPLLAGRRLHP 625
TRIAE_CS42_1BS_TGACv WGNDVGWVYNIATEDVVTGFRLHRTGWRSTYCAMEPDAFRGTAPINLTERLYQILRWSGGSLEMFFSRFCPLLAGRRLHP 624
TRIAE_CS42_2AS_TGACv WGDGVGWVYDMATEDAVTGFRLHRTGWRSMYCDMEPPAFCGTAPINMTERMYQILRWSGGSLEVFFSRFCPLLAGRRLHP 631
TRIAE_CS42_2BS_TGACv WGDGVGWVYDMATEDAVTGFRLHRTGWRSMYCDMEPPAFCGTAPINMTERMYQILRWSGGSLEVFFSRFCPLLAGRRLHP 630
TRIAE_CS42_2DS_TGACv WGDGVGWVYDMATEDAVTGFRLHRTGWRSMYCDMEPPAFCGTAPINMTERMYQILRWSGGSLEVFFSRFCPLLAGRRLHP 628
TRIAE_CS42_2BS_TGACv WGREVGWVYNIATEDVVTGFRLHRNGWRSMYCRMEPDAFAGTAPINLTERLYQILRWSGGSLEMFFSRNCPLLAGRRLHP 457
TRIAE_CS42_2DS_TGACv WGSDVGWVYNIATEDVVTGFRLHRNGWRSMYCRMEPDAFAGTAPINLTERLYQILRWSGGSLEMFFSRNCPLLAGRRLHP 457
TRIAE_CS42_2AS_TGACv WGREVGWVYNIATEDVVTGFRLHRNGWRSMYCRMEPDAFAGTAPINLTERLYQILRWSGGSLEMFFSRNCPLLAGRRLHP 653
TRIAE_CS42_7AL_TGACv WGKEIGWVYDTVTEDVVTGYRMHIKGWRSRYCSIYPHAFIGTAPINLTERLTQVLRWSTGSLEIFFSKNNPLFGSTYLHP 652
TRIAE_CS42_7DL_TGACv WGKEIGWVYDTVTEDVVTGYRMHIKGWRSRYCSIYPHAFIGTAPINLTERLTQVLRWSTGSLEIFFSKNNPLFGSTYLHP 251
TRIAE_CS42_7BL_TGACv WGKEIGWVYDTVTEDVVTGYRMHIKGWRSRYCSIYPHAFIGTAPINLTERLTQVLRWSTGSLEIFFSKNNPLFGSTYLHP 694

TRIAE_CS42_5DL_TGACv LQRLAYLNTTVYPFTSIFLLLYCLLPAIPLVTRSASASAFSVTMPPSGTYMGFVAALMLTLAMVAVLEVRWSGITLGEWW 662
TRIAE_CS42_5AL_TGACv LQRLAYLNTTVYPFTSIFLLLYCLLPAIPLVTRNASTSAFSVNTPPSATYIAFVAALMLTLAMVAVLEVRWSGITLGDWW 660
TRIAE_CS42_5BL_TGACv LQRLAYLNTTVYPFTSIFLLLYCLLPAIPLVTRSASTSAFSVNTPPSATYIGFVAALMLTLAMVAALEVRWSGITLGEWW 661
TRIAE_CS42_2AS_TGACv VQRLSYINFTIYPLTSLFILMYAFCPVMWLLP------TEILVQRPYTRYIVYLIIVIAMIHVIGMFEIMWAGITWLDWW 719
TRIAE_CS42_2BS_TGACv VQRLSYINFTIYPLTSLFILMYAFCPVMWLLP------TEILIQRPYTRYIVYLLIVIAMIHVIGMFEIMWAGITWLDWW 718
```

```
TRIAE_CS42_2DS_TGACv VQRLSYINFTIYPLTSLFILMYAFCPVMWLLP------TEILVQRPYTRYIVYLIIVIVMIHVIGMFEIMWAGITWLDWW 711
TRIAE_CS42_2AS_TGACv LQRVSYLNMTVYPVTSLFILLYALSPVMWLIP------DEVYIQRPFTKYVVFLLVIILMIHVIGWLEIKWAGVTWLDYW 691
TRIAE_CS42_2DS_TGACv LQRVSYLNMTVYPVTSLFILLYALSPVMWLIP------DEVYIQRPFTKYVVFLLVIILMIHVIGWLEIKWAGVTWLDYW 691
TRIAE_CS42_2BS_TGACv LQRVSYLNMTVYPVTSLFILLYALSPVMWLIP------DEVYIQRPFTKYVVFLLVIILMIHVIGWLEIKWAGVTWLDYW 695
TRIAE_CS42_2DL_TGACv LQRIAYLNMSTHPIVTVFILSYNFFPVMWLFS------EQLYIQRPFGMYMGYLVAIIAMVHLIGMFEVRWSGITLLDWF 691
TRIAE_CS42_2BL_TGACv LQRITYLNMSTYPIVTVFILSYNFFPVMWLFS------EQLYIQRPFGTYMAYLVGIIAMVHLIGMFEVRWSGITLLDWF 492
TRIAE_CS42_2AL_TGACv LQRIAYLNMSTYPIVTVFILSYNFFPVMWLFS------EQLYIQRPFGTYMAYLVAIIAMVHLIGMFEVRWSGITLLDWF 694
TRIAE_CS42_2DL_TGACv LQRIAYFNMSTYPIVTVFILAYNFFPVMWLFS------EQLYIQRPFGTYIAYLVAVIAMMHVIGMFEVKWAGITLLDWC 687
TRIAE_CS42_2BS_TGACv LQRIAYLNMSTYPIVTVFILAYNLFPVLWLFS------EQFYIQRPFAWGFFTDQARHVLLGML-------FNVWILVLL 693
TRIAE_CS42_2DS_TGACv LQRVAYLNMSTYPIVTVFILAYNLFPVLWLFS------EQFYIQRPFGTYIMYLVAVIAMIHVIGMFEVKWAGITLLDWC 700
TRIAE_CS42_7AL_TGACv LQRIAYLNMSIYPIATMFILAYSFFPVMWLFSE-----ESYYIQRPFGTFIMYLVAVIAMMHVIGMFEVKWAGITLQDWW 678
TRIAE_CS42_7DL_TGACv LQRIAYLNMSIYPIATMFILAYSFFPVMWLFSE-----QSYYIQRPFGTFIMYLVVVIAMMHVIGMFEVKWAGITLQDWW 676
TRIAE_CS42_7BL_TGACv -------------------------------------------------------------------------------- 572
TRIAE_CS42_U_TGACv1_ MQRVAYINMTTYPVSTFFICMYYLYPVMWLFQ------GEFYIQRPFQTFALFVVVIIATVELIGMVEIRWAGLTLLDWV 699
TRIAE_CS42_1BS_TGACv MQRIAYINMTTYPVSTFFICMYYFYPVMWLFQ------GEFYIQRPFQTFALFVVVIVIATVELIGMVEIRWAGLTPLDWF 698
TRIAE_CS42_2AS_TGACv MQRVAYTNMTFYPLSALFVVCYHLLPLMWVFN------GRFYIQKPYPTYVMYVLVIIVSNEVIGMVEIVWAGLTLLDWF 705
TRIAE_CS42_2BS_TGACv MQRVAYTNMTFYPLSALFVVCYHLLPLMWVFN------GRFYIQKPYPTYVMYVLIIIISNEVIGMVEIVWAGLTLLDWF 704
TRIAE_CS42_2DS_TGACv MQRVAYTNMTFYPLSALFVVCYHLLPLMWVFN------GQFYIQKPYPTYVMYVLIIIVSNEVIGMVEIVWAGLTLLDWF 702
TRIAE_CS42_2BS_TGACv MQRIAYANMTAYPVSSVFLVFYLLFPVIWIFR------GQFYIQKPFPTYVLYLVIVIALTELIGMVEIKWAGLTLLDWI 531
TRIAE_CS42_2DS_TGACv MQRIAYANMTAYPVSSVFLVFYLLFPVIWIFR------GQFYIQKPFPTYVLYLVIVIALTELIGMVEIKWAGLTLLDWI 531
TRIAE_CS42_2AS_TGACv MQRIAYANMTAYPVSSVFLVFYLLFPVIWIFR------GQFYIQKPFPTYVLYLVIVIALTELIGMVEIKWAGLTLLDWI 727
TRIAE_CS42_7AL_TGACv LQRVAYINITTYPFTAIFLIFYTTVPALSFVTG------HFIVQRPTTMFYVYLGIVLSTLLVIAVLEVKWAGVTVFEWF 726
TRIAE_CS42_7DL_TGACv LQRVAYINITTYPFTAIFLIFYTTVPALSFVTG------HFIVQRPTTMFYVYLGIVLSTLLVIAVLEVKWAGVTVFEWF 325
TRIAE_CS42_7BL_TGACv LQRVAYINITTYPFTAIFLIFYTTVPALSFVTG------HFIVQRPTTMFYVYLGIVLSTLLVIAVLEVKWAGVTVFEWF 768

TRIAE_CS42_5DL_TGACv RNEQFWMVSATSAYAAAVVQVALKVSAGKEIAFKLTSKQRAS-SPGGGVKERFAELYAVRWTVLMVPTAVVLAVNVMSMA 741
TRIAE_CS42_5AL_TGACv RNEQFWMVSATSAYAAAVVQVALKVAAGKEIAFKLTSKHRASNSGGGVVKDRFAELYAVRWTVLMVPTAVVLAVNVTSMA 740
TRIAE_CS42_5BL_TGACv RNEQFWMVSATSAYAAAVVQVALKVAAGKEIAFKLTSKQRAPSAGGGVVKRQVRGAVRREMDGADGSDGGGADGERGVHG 741
TRIAE_CS42_2AS_TGACv RNEQFFMIGSVTAYPTAVLHMVVNLLTKKGIHFRVTTKQPVADTDDK-----YAEMYEVHWVPMMVPAVVILFSNILAIG 794
TRIAE_CS42_2BS_TGACv RNEQFFMIGSVTAYPTAVLHMVVNLLTKKGIHFRVTTKQPVADTDDK-----YAEMYEVHWVPMMVPAVVVLFSNILAIG 793
TRIAE_CS42_2DS_TGACv RNEQFFMIGSVTAYPTAVLHMVVNILTKKGIHFRVTTKQPVADTDDK-----YAEMYEVHWVPMMIPAVVVLFSNILAIG 786
TRIAE_CS42_2AS_TGACv RNEQFFMIGSTSAYPAAVLHMVVNLLTKKGIHFRVTSKQTAADTNDK-----FADLYDMRWVPMLIPTTVVLIANVGAIG 766
TRIAE_CS42_2DS_TGACv RNEQFFMIGSTSAYPAAVLHMVVNLLTKKGIHFRVTSKQTAADTNDK-----FADLYDMRWVPMLIPTTVVLIANVGAIG 766
TRIAE_CS42_2BS_TGACv RNEQFFMIGSTSAYPAAVLHMVVNLLTKKGIHFRVTSKQTAADTNDK-----FADLYDMRWVPMLIPTTVVLIANVGAIG 770
TRIAE_CS42_2DL_TGACv RNEQFYMIGATGVYPTAVLYMLLKLATGKGIYFRLTSKQTEACSNDK-----FADLYTVRWVPLLIPTTAVIIVNVAAVG 766
TRIAE_CS42_2BL_TGACv RNEQFYMIGATGVYPTAVLYMLLKLVTGKGIYFRLTSKQTEGCSNDK-----FADLYTVRWVPLLIPTAAVIIVNVAAIG 567
TRIAE_CS42_2AL_TGACv RNEQFYMIGATGVYPTAVLYMLLKLVTGKGIYFRLTSKQTEACSNDK-----FADLYTVRWVPLLIPTTAVIIVNVAAVG 769
TRIAE_CS42_2DL_TGACv RNEQFYLIAATGVYPTAVLYMALKLVTGKGMHFRLTSKQTEACSRDK-----FANLYTVRWVPLLIPTTAVLVVNVAAVG 762
TRIAE_CS42_2BS_TGACv YPFALGIMGKWGKRPVILFVMLVMAVGAVGLLYVAFHAPYPADFSEV-----AASLGEASLTGPSG-------------- 754
TRIAE_CS42_2DS_TGACv RNEQFYMIGATGVYPTAVLYMALKLVTGKGIYFRLTSKQTDACSNDK-----XXXXXXXXXXXXXXXXXXXXXXXGCRCC 775
TRIAE_CS42_7AL_TGACv RNEQFYMITATGVYPTAVLYMALKLIRGKGIYFRLTSKQTEACSGEK-----FADLYTVRWVPLLIPTVAVLVVNIAAIG 753
TRIAE_CS42_7DL_TGACv RNEQFYMIAATGVYPTAVLYMALKLIRGKGIYFRLTSKQTEACSDEK-----FADLYTVRWVPLLIPTVAVLIVNVTAVG 751
TRIAE_CS42_7BL_TGACv ----------------------------QAAPSRCSSPTATLSSPAV-----GSTLYSASLTSTCRSTRSPRCSS----- 614
TRIAE_CS42_U_TGACv1_ RNEQFYIIGTTGVYPMAMLHILLRSLGIKGVSFKLTAKKLTGGARER-----LAELYDVQWVPLLVPTVVVMAVNVAAIG 774
TRIAE_CS42_1BS_TGACv RNEQFYIIGTTGVYPMAMLHIILRSLGIKGVSFKLTAKKLTSGTRER-----LAELYDVQWVPLLVPTVVVMAVNVAAIG 773
TRIAE_CS42_2AS_TGACv RNEQFYMICATGVYPTAVLHVVLRSLGLKGMSFKMTAKQLATGARER-----FAELYNVQWAPLLIPTLVVIAVNVVAIG 780
TRIAE_CS42_2BS_TGACv RNEQFYMICATGVYPTAVLHVVLRSLGLKGISFKMTAKQLATGARER-----FAELYDVQWAPLLIPTLVVIAVNVVAIG 779
TRIAE_CS42_2DS_TGACv RNEQFYMICATGVYPTAVLHVVLRSLGLKGMSFKMTAKQLATGARER-----FAELYDVQWAPLLIPTLVVIAVNVVAIG 777
TRIAE_CS42_2BS_TGACv RNEQFYIIGATAVYPTAVFHIVLKLFGLKGVSFKLTAKQVASSTSDK-----FAELYAVQWAPMLIPTMVVIAVNVCAIG 606
TRIAE_CS42_2DS_TGACv RNEQFYIIGATAVYPTAVFHIVLKLFGLKGVSFKLTAKQVASSTSDK-----FAELYAVQWAPMLIPTMVVIAVNVCAIG 606
TRIAE_CS42_2AS_TGACv RNEQFYIIGATAVYPTAVFHIVLKLFGLKGVSFKLTAKQVASSTSDK-----FAELYAVQWAPMLIPTMVVIAVNVCAIG 802
TRIAE_CS42_7AL_TGACv RNGQFWMTASCSAYLAAVCQVLTKVIFRRDISFKLTSKLPSGDEKKD----PYADLYVVRWTPLMITPIIIIFVNIIGSA 802
TRIAE_CS42_7DL_TGACv RNGQFWMTASCSAYLAAVCQVLTKVIFRRDISFKLTSKLPSGDEKKD----PYADLYVVRWTPLMITPIIIIFVNIIGSA 401
TRIAE_CS42_7BL_TGACv RNGQFWMTASCSAYLAAVCQVLTKVIFRRDISFKLTSKLPSGDEKKD----PYADLYVVRWTPLMITPIIIIFVNIIGSA 844

TRIAE_CS42_5DL_TGACv AAVQEGRWRK-------GPAAVLAMAFNAWVVVHLHPFALGLMGRWSKTLSPLLLLVVGFTVLSLCFVLHLHML------ 808
TRIAE_CS42_5AL_TGACv AAVQEGRWRK-------GPAAVLAMAFNAWVVVHLYPFALGLMGRWSKTLSPLLLLVVVFTVLSLCFVLHLHML------ 807
TRIAE_CS42_5BL_TGACv SSGTRGTVEE-------RPRGGARDGVQRVGGGASPPVRPWSHGPLEQDVEPPALARRSVHSSITMFCPPFAYALIWLLF 814
TRIAE_CS42_2AS_TGACv VAIGKSVLYMGTWSAAQRRHGALGLLFNLWIMVLLYPFALAIIGRWAKRTGILFILLPIAFLSTALMYIGIHTFLLHFFP 874
TRIAE_CS42_2BS_TGACv VAIGKSVLYMGTWSAAQKRHGALGLLFNMWIMVLLYPFALAIIGRWAKRTGILFILLPIAFLSTALMYIGIHTFLLHFFP 873
TRIAE_CS42_2DS_TGACv VAIGKSILYMGTWSAAQKRHGALGLLFNLWIMVLLYPFALAIIGRWAKRTGILFILLPIAFLSTSLMYIGVHTFLLHFFP 866
TRIAE_CS42_2AS_TGACv VAMGKTIVYMGAWTIAQKTHAALGLLFNVWIMVLLYPFALAIMGRWAKRPVILVVLLPVAFTIVCLVYYVAVHILLLSYLT 846
TRIAE_CS42_2DS_TGACv VAMGKTIVYMGAWTIAQKTHAALGLLFNVWIMVLLYPFALAIMGRWAKRPVILLVLLPVAFTIVCLVYYVAVHILLLSYLT 846
TRIAE_CS42_2BS_TGACv VAMGKTIVYMGAWTIAQKTHAALGLLFNVWIMVLLYPFALAIMGRWAKRPVILLVLLPVAFTIVCLVYYVAVHILLLSYLT 850
TRIAE_CS42_2DL_TGACv AAIGKAATWG--FFTDEARHALLGMVFNMGILVLLYPFALGIMGKWAKRPIILFIVLVMAISVVGLLYVSLHAPYTGEWS 844
TRIAE_CS42_2BL_TGACv AAIGKAATWG--FFTDEARHALLGMVFNMGILVLLYPFALGIMGKWGKRPIILFIVLVMAISVVGLLYVTLHAPYTGEWS 645
TRIAE_CS42_2AL_TGACv AAIGKAATWG--FFTDEARHALLGMVFNMGILVLLYPFALGIMGKWGKRPIILFIVLVMAISAVGLLYVMLHAPYTGEWS 847
TRIAE_CS42_2DL_TGACv AAIGKAAAWG--FSTDQARHVLLGMVFNVGTLMLLYPFALGIMGKRGKTPVILFVLLLMAIAAVGLLYVTLYAPYPQESL 840
TRIAE_CS42_2BS_TGACv -------------------------------------------------------------------------------- 754
TRIAE_CS42_2DS_TGACv SRPSWCSS------------------------------------------------------------------------ 783
TRIAE_CS42_7AL_TGACv AAIGKAATWG--FFTDQAWHAVLGMVFNVGTLVLLYPFALGIMGQWGKRPGILLVMLVMAIGTVGLLYVTLQQDGHRMSF 831
TRIAE_CS42_7DL_TGACv AAIGKAATWG--FFTDQAWHAVLGMVFNVGTLVLLYPFALGIMGQWGKRPGILLVMLVMAIGTVGLLYVTLQQDGHRMSF 829
TRIAE_CS42_7BL_TGACv -------------------------------------------------------------------------------- 614
TRIAE_CS42_U_TGACv1_ AAAGKAIVGR--WSAAQVAGAASGLVFNVWMLLLLYPFALGIMGRWSKRPYILFIVLVTAVAATASMYVALAGSLPYLHS 852
TRIAE_CS42_1BS_TGACv AAAGKAIAGR--WSAAQVAGAASGLVFNVWMLLLLYPFALGIMGRWSKRPYILFIVLVTAVAATASVYVALAGSLPYLHS 851
TRIAE_CS42_2AS_TGACv AAVGKAITWG--WSAGQVVEAASGLMFNVWILLMFYPFALGVIGRWGKRPYVLFAMFVAAFAAIAAVYVAVQAALAGNLL 858
TRIAE_CS42_2BS_TGACv AAVGKAITWG--WSAGQVVEAASGLMFNVWILLMFYPFALGVIGRWGKKPYVLFAMFVAAFAAIAAVYVAVQAALAGNLP 857
TRIAE_CS42_2DS_TGACv VAVGKAITWG--WSAGQVVEAASGLMFNVWILLMFYPFALGVIGRWGKRPFVLFAMFVAAFAAIAAVYVAVQAALAGNLP 855
TRIAE_CS42_2BS_TGACv ASIGKAIVGG--WSLMQMADAGLGLVFNAWILVLIYPFALGMIGRWSKRPYILFILFVIAFILIALVDIAIQAMRSGFVR 684
TRIAE_CS42_2DS_TGACv ASIGKAIVGG--WSLMQMADAGLGLVFNAWILVLIYPFALGMIGRWSKRPYILFILFVIAFILIALVDIAIQAMRSGFVR 684
TRIAE_CS42_2AS_TGACv ASIGKAIVGG--WSLMQMADAGLGLVFNAWILVLIYPFALGMIGRWSKRPYILFILFVIAFILIALVDIAIQAMRSGFVR 880
```

```
TRIAE_CS42_7AL_TGACv VAFAKVLDGEW----THWLKVAGGVFFNFWVLFHLYPFAKGILGKHGKTPVVVLVWWAFTFVITAVLYINIPHMHSSGGK 878
TRIAE_CS42_7DL_TGACv VAFAKVLDGEW----THWLKVAGGVFFNFWVLFHLYPFAKGILGKHGKTPVVVLVWWAFTFVITAVLYINIPHMHSSGGK 477
TRIAE_CS42_7BL_TGACv VAFAKVLDGEW----THWLKVAGGVFFNFWVLFHLYPFAKGILGKHGKTPVVVLVWWAFTFVITAVLYINIPHMHSSGGK 920

TRIAE_CS42_5DL_TGACv --------------------- 808
TRIAE_CS42_5AL_TGACv --------------------- 807
TRIAE_CS42_5BL_TGACv G-------------------- 815
TRIAE_CS42_2AS_TGACv SMLI----------------- 878
TRIAE_CS42_2BS_TGACv SMLI----------------- 877
TRIAE_CS42_2DS_TGACv SMLI----------------- 870
TRIAE_CS42_2AS_TGACv F-------------------- 847
TRIAE_CS42_2DS_TGACv F-------------------- 847
TRIAE_CS42_2BS_TGACv F-------------------- 851
TRIAE_CS42_2DL_TGACv QVAVSLGKASLTGPSGSG--- 862
TRIAE_CS42_2BL_TGACv QVAVSLGKASLTGPSGSG--- 663
TRIAE_CS42_2AL_TGACv QVAVSLGKASLTGPSGSG--- 865
TRIAE_CS42_2DL_TGACv TFLSW---------------- 845
TRIAE_CS42_2BS_TGACv --------------------- 754
TRIAE_CS42_2DS_TGACv --------------------- 783
TRIAE_CS42_7AL_TGACv LTRPSG--------------- 837
TRIAE_CS42_7DL_TGACv LTRPSG--------------- 835
TRIAE_CS42_7BL_TGACv --------------------- 614
TRIAE_CS42_U_TGACv1_ GIKLV---------------- 857
TRIAE_CS42_1BS_TGACv GIKLV---------------- 856
TRIAE_CS42_2AS_TGACv YFQLGHWSIGGAVSLPSRRV- 878
TRIAE_CS42_2BS_TGACv YFQLGHWSIGGAVSLPSRRV- 877
TRIAE_CS42_2DS_TGACv YFQLGHRSIGGAVSLASRRV- 875
TRIAE_CS42_2BS_TGACv FHFKSSGGATFPTSWGL---- 701
TRIAE_CS42_2DS_TGACv FHFKSSGGATFPTSWGL---- 701
TRIAE_CS42_2AS_TGACv FHFKSSGGATFPTSWGL---- 897
TRIAE_CS42_7AL_TGACv HTTVHGHHGKKFVDAGYYNWP 899
TRIAE_CS42_7DL_TGACv HTTVHGHHGKKFVDAGYYNWP 498
TRIAE_CS42_7BL_TGACv HTTVHGHHGKKFVDAGYYNWP 941
```

Fig. S_2F: *CslH* & *CslJ* subfamilies.

S.No	Gene name with number of splice variants (CslH)	No. of amino acids (aa)
1	TRIAE_CS42_3DS_TGACv1_271739_AA0907200.1	714 aa
2	TRIAE_CS42_3AS_TGACv1_212952_AA0704280.1	331 aa
3	TRIAE_CS42_3B_TGACv1_222234_AA0760340.1	751 aa
4	TRIAE_CS42_3B_TGACv1_221049_AA0728260.1	458 aa
5	TRIAE_CS42_3DS_TGACv1_273502_AA0931770.1	579 aa
6	TRIAE_CS42_2AL_TGACv1_094351_AA0296300.3_3_SPLICE	752 aa
7	TRIAE_CS42_2DL_TGACv1_158387_AA0517170.1	752 aa
8	TRIAE_CS42_2BL_TGACv1_129372_AA0380770.1	799 aa

S.No	Gene name with number of splice variants (CslJ)	No. of amino acids (aa)
1	TRIAE_CS42_3DS_TGACv1_272297_AA0918580.1	738 aa
2	TRIAE_CS42_3AS_TGACv1_210908_AA0681280.2_2_SPLICE	766 aa
3	TRIAE_CS42_3B_TGACv1_221705_AA0747940.1	734 aa
4	TRIAE_CS42_3DS_TGACv1_272756_AA0924850.1_2_SPLICE	734 aa

```
TRIAE_CS42_2AL_TGACv -----------------------------------------------MAGGKKLHERVALGRTAWMLADFVILLLLLALV 33
TRIAE_CS42_2BL_TGACv MHRGEDSLSGLYKCTLAFVACGCGWSCGVVLLASLLLLVASYLSATAMAGGKKLQERVALGRSAWMLADFVILFLVLALV 80
TRIAE_CS42_2DL_TGACv -----------------------------------------------MAGGKKLQERVALGRTAWMLADFVILLLLLALV 33
TRIAE_CS42_3AS_TGACv -------------------------------------------------------------------------------- 0
TRIAE_CS42_3B_TGACv1 --------------------------------------------------MSSAMKLQERVSVPRTAWKLADIFILCLLF 30
TRIAE_CS42_3DS_TGACv -----------------------------------------------MSSAMKLQERVIVPRTAWKLADIFILCLLFALL 33
TRIAE_CS42_3B_TGACv1 -----------------------------------------------MSSAMKLQERVTVPRTAWKLADIFILCLLLVLL 33
TRIAE_CS42_3DS_TGACv -----------------------------------------------MGSAMKLQERVILPRTAWKLADIFILCLLFALL 33

TRIAE_CS42_2AL_TGACv ARRAASLGE--RGGTWLAALVCEAWFAFVWILNMNGKWSPVRFDTYPENLSHRLEELPAVDMFVTTADPALEPPLITVNT 111
TRIAE_CS42_2BL_TGACv ARRAASLGE--RGGTWLAALVCEAWFAFVWILNMNGKWSPVRFDTYPENLSHRMEELPAVDMFVTTADPALEPPLITVNT 158
TRIAE_CS42_2DL_TGACv ARRAASLGE--RGGTWLAALVCEAWFAFVWILNMNGKWSPVRFDTYPDNLSHRMEELPAVDMFVTTADPALEPPLITVNT 111
TRIAE_CS42_3AS_TGACv -------------------------------------------------------------------------------- 0
TRIAE_CS42_3B_TGACv1 ALLSCRVASLREGGASVAALVCEAWFTFVWIINMNIKWNPVRFNTYPENLSQRTDELPAVDMLVTTADPELEPPLMTVNT 110
TRIAE_CS42_3DS_TGACv SCRVLSLGEGGAGAASVAALVCEAWFTFVWILNMNIRWNPVRFHTYPENLSQRMDGLPAVDMLVTTADPELEPPLMTVNT 113
TRIAE_CS42_3B_TGACv1 SCRVASLGEGGAG---AAALVCEAWFTFVWILNMNIKWNPVRFHTYPENLSQRMDELPAVDMLVTTADPELEPPLMTVNT 110
TRIAE_CS42_3DS_TGACv SCRVASLGDGGAGAASVAALVCEAWFTFVWILNMNIKWNPVRFHTYPENLSQRMDELPAVDMLVTTADPELEPPLMTVNT 113

TRIAE_CS42_2AL_TGACv VLSLLALDYPDVGKLACYVSDDGCSPVTCYALREAAKFASLWIPFCKRYDVGVRAPFMYFSSAPEVGTGTADHEFLESWA 191
TRIAE_CS42_2BL_TGACv VLSLLALDYPHVGKLACYVSDDGCSPLTCYSLREAAKFASLWVPFCKRHDVGVRAPFMYFSSAPEVDTGTVDHEFLESWA 238
TRIAE_CS42_2DL_TGACv VLSLLALDYPDVGRLACYVSDDGCSPVTCYALREAAKFAGLWVPFCKRHDVGVRAPFMYFSSAPEVGNGTVDHEFLESWA 191
TRIAE_CS42_3AS_TGACv -------------------------------------------------------------------------------- 0
TRIAE_CS42_3B_TGACv1 VLSLLAVDYPDVDKLACYVSDDGCSPVTCYALREAAGFARLWVPFCKRHGVGVRAPFMYFAS--SRPEPELAG--DWTFI 186
TRIAE_CS42_3DS_TGACv VLSLLAMDYPDVDKLACYVSDDGCSPVTCYALHEAARFAGLWVPFCKRHGVGVRAPFMYFAS--RPEPELAGDNFSDEWT 191
TRIAE_CS42_3B_TGACv1 VLSLLAVDYPDVDKLACYVSDDGCSPVTCYALREAAGFARLWVPFCKRHGVGVRAPFIYFASS-RPEPDLAGDKFSDDWI 189
TRIAE_CS42_3DS_TGACv VLSLLAVDYPDVDKLACYVSDDGCSPATCYALREAAWFARLWVPFCKRHDVRVRAPIIYFAS--RLEPELAGDTFSDEWT 191

TRIAE_CS42_2AL_TGACv LMKTEYEKLASRIENADEVSILR-DGGEEFAEFIDAERGNHPTIVKVLWDNSKSK-AGEGFPHLVYLSREKSPRHRHNFK 269
TRIAE_CS42_2BL_TGACv LMKSEYEKLASRIENADEVSILR-DGGDEFAEFIDAERGNHPTIVKVLWDNSKNK-TGEGFPHLVYLSREKSPRHRHNFK 316
TRIAE_CS42_2DL_TGACv LMKSQYEKLARRIENADEGTIMR-DGGDEFAEFIDAERGNHPTIVKVLWDNSKSK-AGEEFPHLVYLSREKSPRHRHNFK 269
TRIAE_CS42_3AS_TGACv -------------------------------------------------------------------------------- 0
TRIAE_CS42_3B_TGACv1 KSEYDKLVSRIESADEGSLLRHDDAADFTEFKEAKRGDHPAIVKVLWDNSKSSRTGSGDGFPNLVYVSREKTRKHDHHYK 266
TRIAE_CS42_3DS_TGACv FIKSEYDKLVSRIESADEGSLLRDDDAGEFTEFMEAKRGDHPGIVKVLWDNSKSSRTGEGFPNLVYVSREKSRKHDHHYK 271
TRIAE_CS42_3B_TGACv1 FIKSEYDKLVSLIESADEASLLRHDHAGEFTEFKGAECGDHPAIVKVLWDNSKSSGTGEGFPNLVYVSREKSRKHDHHYK 269
TRIAE_CS42_3DS_TGACv FIKSEYDKLVSRIESADEGSLLRHDDAGEFTEFMEAERTDHPAIVKVLWDNSKSSRTGEAFPHLVYVSSEKSRKHHHHYK 271

TRIAE_CS42_2AL_TGACv AGAMNVLTRVSAVMTNAPIMLNVDCDMFANNPQVALHAMCLLLGFDDEIHSGFVQAPQKFYGGLKDDPFGNQMQVITKKI 349
TRIAE_CS42_2BL_TGACv AGAMNVLTRVSAVMTNAPIMLNVDCDMFANNPQVALHAMCLLLGFDDEIHSGFVQAPQKFYGGLKDDPFGNQMQVITKKI 396
TRIAE_CS42_2DL_TGACv AGAMNVLTRVSAVMTNAPIMLNVDCDMFANNPQVALHAMCLLLGFDDEIHSGFVQAPQKFYGGLKDDPFGNQMQVITKKI 349
TRIAE_CS42_3AS_TGACv -------------------------------------------------------------------------------- 0
TRIAE_CS42_3B_TGACv1 AGAMNVLARVSAVMTNAPIILNMDCDMFVNNPQVVLHAMCLLLGFNDETCSGFVQVPQRFYAKLKDDPFGNQIEVLREKL 346
TRIAE_CS42_3DS_TGACv AGAMNVLARVSAVMTNAPIILNVDCDMFVNNSQVVLHAMCLLLGFDDETCSGFVQVPQRFYGKLKDDPFGNQMEVLREKL 351
TRIAE_CS42_3B_TGACv1 AGAMNVLARVSAVMTNAPIILNVDCDMFVNNPQVVLHATCLLLGFDDETCSGFVQVPQRFYGKLKDDPFGNQMEVLRS-- 347
TRIAE_CS42_3DS_TGACv AGAMNVLARVSAVMTNAPIILNVDCDMFVNNSQVVLHAMCLLLGFDDETCSGFVQVPQRFYGKLKDDPFGNQMEVLREKL 351

TRIAE_CS42_2AL_TGACv GGGLAGIQGTFYGGTGCFHRRKVIYGMPPPD--TVKHETRGSPSYKELQAKFGSSKELIESSRNIISGDLLARPTVDISS 427
TRIAE_CS42_2BL_TGACv GGGLAGIQGTFYGGTGCFHRRKVIYGMPPPD--TVKHETRGSPSYKELQAKFGSSKELIESSRNIISGDLLARPTVDISS 474
TRIAE_CS42_2DL_TGACv GGGLAGIQGMFYGGTGCFHRRKVIYGVPPPD--TVKHEMKGSPSYKELQAKFGSSKELIESSRNIISGDLLARPTVDLSS 427
TRIAE_CS42_3AS_TGACv --------------------------------------------------------------------------MIDISS 6
TRIAE_CS42_3B_TGACv1 LGGLSGLQGIYYLGTGCFHRRKIIYGVAPPSFAAVKHERQGSLTYEDLRTKFGASVELAESARNIYSREIPLKPMIDISS 426
```

```
TRIAE_CS42_3DS_TGACv FGGLAGLQGIYYLGMGCFHRRKIIYGVAPSSSAAIKHEREGSRSYEDLRTKFGASVELVESARNIYSGEIPPSPMIDISS 431
TRIAE_CS42_3B_TGACv1 ----------------------------------------LSYEDLLTKFGASMELVESSRNIYSVEIPPKPMIDITS 385
TRIAE_CS42_3DS_TGACv LGGLSGLQGIFYLGTGCFHRRKIIYGVAPSSFAAVKHEREGSLSYEDLRTKFGASVELVESTRNIYSREIPPKPMVNISS 431

TRIAE_CS42_2AL_TGACv RVEMAKQVGDCNYEAGTCWGQEIGWVYGSMTEDILTGQRIQAAGWESALLDTDPPAFLGCAPTGGPASLTQFKRWATGLL 507
TRIAE_CS42_2BL_TGACv RVEMAKQVGDCNYEAGTCWGQEIGWVYGSMTEDILTGQRIQAAGWESALLDTDPPAFLGCAPTGGPASLTQFKRWATGLL 554
TRIAE_CS42_2DL_TGACv RVEMAKQVGDCNYEAGTCWGQEIGWVYGSMTEDILTGLRIHAAGWESALLDTEPPAFLGCAPTGGPASLTQFKRWATGLL 507
TRIAE_CS42_3AS_TGACv RIQVAKQVSSCNYETDTHWGQEIGWSYGSMAEDILTGQRIHSSGWKSTLLDTNPPAFLGCAPTGGPASLTQYKRWATGLL 86
TRIAE_CS42_3B_TGACv1 RIQVAKQVSSCNYETGTHWGQEIGWSYGSMAEDILTGQRIHSAGWKSTSPDTNPPAFLGCAPTGGPASLTQYKRWATGLL 506
TRIAE_CS42_3DS_TGACv RIQVAKQVSSCNYETDTHWGQEIGWSYGSMAEDILTGQRIHSSGWKSTLLDTNPPAFLGCAPTGGPASLTQYKRWATGLL 511
TRIAE_CS42_3B_TGACv1 RIQVAKQVSTCNYETGTHWGEEASNHG----------------------------------------------------- 412
TRIAE_CS42_3DS_TGACv CIQVAKQVSSCNYETGTHWGQEIGWSYGSMAEDILTGQRIHSAGWKSTLLDTNPPAFLGCAPTGGPASLTQYKRWATGVL 511

TRIAE_CS42_2AL_TGACv EILISRNSPILGTIFKGLQLRQCLGYLIVDAWPVRAPFELCYALLGPFCLLTNQSFLPTASDEGFHIPAALFLTYNIYHL 587
TRIAE_CS42_2BL_TGACv EILISRNSPILGTIFRRLQLRQCLAYLIVNAWPMRAPFEMCYALLGPFCLLTNQSFLPTTSNEGFRIPAALFLSYHVYHL 634
TRIAE_CS42_2DL_TGACv EILISQNSPILGTIFRRLQLRQCLAYLIVEAWPVRAPFELCYALLGPFCLLTNQSFLPTASDEGFRIPAALFLTCHIYHL 587
TRIAE_CS42_3AS_TGACv EILLGQNSPIIATIFKRLQFRQFLAYLVFYVWSMRAPFELCYALLGPFCLFRNQSFLLKASNHGFSIQLALFLSYNIYNF 166
TRIAE_CS42_3B_TGACv1 EILLGPNTPIIATIFKRLQFRQYLGYLVFYVWSMRAPFELCYALLGPFCLFRNHSFLLKASNHGFSIQLALFLSYNIYNF 586
TRIAE_CS42_3DS_TGACv EILLGQNSPIMATVFKRLQFRQSLAYLVFYVWSMRAPFELCYALLGPFCLFRNQSFLLKASNHGFSIQLALFLSYNIYNF 591
TRIAE_CS42_3B_TGACv1 -FSIQLALFLSYNIYNFVEYKECGLSARTWWNNMRMRINLLLAPCFP--------------------------------- 458
TRIAE_CS42_3DS_TGACv EILLGQNCPIIATIFKRLQFRQCLAYLVFYVWSMRAPFELCYALLGPFCLFRNHSFLLKHQTMVSASN------------ 579

TRIAE_CS42_2AL_TGACv MEYKECGLSVRAWWNNHRMQRITSASAWLLAFLTVILKTLGLSETVFEVTRKESSTSSDGGAGTDDADPGLFTFDSAPVF 667
TRIAE_CS42_2BL_TGACv MEYKECGLSVRAWWNNHRMQRITSASAWLLAFLTVILKTLGLSETVFEVTRKESSTSSDGGTGTDEADTGLFTFDSAPVF 714
TRIAE_CS42_2DL_TGACv MEYKECGLSVRAWWNNHRMQRITSASAWLLAFLTVILKTLGLSETVFEVTRKESSTSSDGGAGTDEADPGLFTFDSAPVF 667
TRIAE_CS42_3AS_TGACv VEYMDCGLSARTWWNNMRMQRIVSISSWLLAFLSVVLKTIGLSKTVFEVTREDKST-SDGDPSTHETDLGWFTFDSSLVF 245
TRIAE_CS42_3B_TGACv1 VEYMECGLSARTWWNNMRMQRIVSLSSWLLAFLSVVLKTIGLSKTVFEVTRKDKST-SDGDPSTHETDLGWFTFDSSPVF 665
TRIAE_CS42_3DS_TGACv VEYMECGLSARTWWNNMRMQRIVSISSWLLDFLSVVLKTIGLSKTVFEVTRKDKST-SDGDPSTHETDLGWFTFDSSPVF 670
TRIAE_CS42_3B_TGACv1 -------------------------------------------------------------------------------- 458
TRIAE_CS42_3DS_TGACv -------------------------------------------------------------------------------- 579

TRIAE_CS42_2AL_TGACv IPVTALSVLNIVALTVAAWRAVVGTVAG-VHGGPGVGEFVCCGWMVLCFWPFVRGLVSSGKYGIPWSVRVKAGLIVAAFV 746
TRIAE_CS42_2BL_TGACv IPVTALSMLNIVALAVAAWRAVVGTAAG-VHGGPGVGEFVCCGWMVLCFWPFMRGLVSSGKYGIPWSVRVKAGLIVAAFV 793
TRIAE_CS42_2DL_TGACv IPVTVLSMLNIVALAVAAWRAVVGAAAG-VHGGPGIGEFVCCGWIVLCFWPFVRGLVSRGKYGIPWSVRVKAGLIVAAFV 746
TRIAE_CS42_3AS_TGACv IPVTTVAILNIATIAIGVWRHAIFWMITGNHDWQNIGEFICCGWAILYFWPFIKGLVGRGRYGIPWNVKLKAWVIVVAFL 325
TRIAE_CS42_3B_TGACv1 IPMTAVAILNIVTIAIGVWRHAIFWMTTGNHDCQNIGEFLCCGLMILYFWPFIKGLVGRGRYGIPWNVKLKAWVIVVAFL 745
TRIAE_CS42_3DS_TGACv ILVTTVAILNIATIAIGVWRHAIFWMITGNHDCQNIGELCVLDG------------------------------------ 714
TRIAE_CS42_3B_TGACv1 -------------------------------------------------------------------------------- 458
TRIAE_CS42_3DS_TGACv -------------------------------------------------------------------------------- 579

TRIAE_CS42_2AL_TGACv HLCTRN 752
TRIAE_CS42_2BL_TGACv HLCTRN 799
TRIAE_CS42_2DL_TGACv HICTRN 752
TRIAE_CS42_3AS_TGACv YFCRGD 331
TRIAE_CS42_3B_TGACv1 YFCRGD 751
TRIAE_CS42_3DS_TGACv ------ 714
TRIAE_CS42_3B_TGACv1 ------ 458
TRIAE_CS42_3DS_TGACv ------ 579
```

Color Align Conservation results

```
TRIAE_CS42_3B_TGACv1 MAAKPSQDAPLQLHTVEVDQPIATVNRLLAVLHVALAAAAIAHRGAHVMLAADLVLLFLWALSQAPMWRPVSRAAFPSRL 80
TRIAE_CS42_3DS_TGACv MATKPSQDAPLPLHTVQTDQPLATVNRLLAAVHLALGAAAIAHRGAHVMLAADLVLLFLWALSQAPMWRPVSRTAFPSRL 80
TRIAE_CS42_3AS_TGACv MAARPSQDAPLQLHTVQTDQPLATVNRLLAALHVALAAAAIAHRGAHVMLAPDLVLLFLWALSQAPMWRPVSRAAFPSRL 80
TRIAE_CS42_3DS_TGACv MAAEPSQDAPLQLHTVQTDQPLATVNRHLAALHVALAAAAIAHRGAHVMLAADLVLLFLWALSQAPMWRPVSRAAFPSRL 80

TRIAE_CS42_3B_TGACv1 SRAALPAVDVMVVTADPDKEPAAKVMNTVVSAMALNYPGGRLSVYLSDDAGSPRTLLAARKAYAFARAWVPFCRKYGVRC 160
TRIAE_CS42_3DS_TGACv SRAALPAVDVMVVTADPEKEPAAKVMNTVVSAMALDYPGGRLSVYLSDDAGSPRTLLAARKAYAFARAWVPFCRKYGVRC 160
TRIAE_CS42_3AS_TGACv SRPALPAVDVMVVTADPDKEPAAKVMNTVVSAMALDYPGGRLSVYLSDDAGSPRTLLAARKAYALARAWVPFCRKYGVRC 160
TRIAE_CS42_3DS_TGACv SRAALPAVDVMVVTADPDKEPAAKVMNTVVSAMALDYPGGRLSVYLSDDAGSPRTLLAARKAYAFARAWVPFCRKYGVRC 160

TRIAE_CS42_3B_TGACv1 PCPDRFFAGDDQLDLDGHHRQELDDDRLRIKKMYETFKEGVEEVMSDAALSQSWTKADHDAHVEIITGDE-QDSSNSNSG 239
TRIAE_CS42_3DS_TGACv PCPDRFFAGDDKLDLGSHHHHELADDRLRIKNMYETFNEGVREVMSDADLSQSCTKADHDAHVEIITGDE-QDSSNSNSG 239
TRIAE_CS42_3AS_TGACv PCPDRFFAGDDQLDLGDHHRQELDDDRLRIKNVYETFKEGVEEVMNDATLSQSWTKADHHAHVEIITDEQGQDSSHSNSG 240
TRIAE_CS42_3DS_TGACv PCPDRFFAGDDQLDLGGHHRQELDDDRLRIKNMYETFKEGVEKVMNDAALSQSWTKADHDAHVE-------QDSSNSNSG 233

TRIAE_CS42_3B_TGACv1 DGEEDEDATPLLVYVSRGKRRSSTHHFKAGALNVLLRVSSLMSNSPYVMVIDCDMYCNSRSSILEAMCFHLDGRRRADLA 319
TRIAE_CS42_3DS_TGACv DGEEDEDAMPLLVYVSREKRRSSTHHFKAGALNVLLRVSSLLSNSPYVMVIDCDMYCNSRSSILEAMCFHLDGRRRADLA 319
TRIAE_CS42_3AS_TGACv DGDGDEDAMPLLVYVSREKRRSSTHHFKAGALNVLLRVSSLMSNSPYVMVIDCDMYCNSRSSLLEAMCFHLDGRRRADLA 320
TRIAE_CS42_3DS_TGACv DGEEDEDAMPLLVYVSREKRRSSAHHFKAGALNVLLRVSSLMSNSPYVMVIDCDMYCNSWSSVLEAMCFHLDGRRRADLA 313

TRIAE_CS42_3B_TGACv1 FVQFPQMFHNLSTSDIYANELRSIFWT----------------------------RWKGLDGLRGPILSGTGFCARRDAI 371
TRIAE_CS42_3DS_TGACv FVQFPQMFHNLSSSDIYANELRSIFWT----------------------------RWKGLDGLRGPILSGTGFCARRDAI 371
TRIAE_CS42_3AS_TGACv FVQFPQMFHNLSSSDIYANELRPIFWVRKKTNRPCIASVIFSEFSSNLGACMVQTRWKGLDGLRGPILSGTGFCVRRDAV 400
TRIAE_CS42_3DS_TGACv FVQFPQMFHNLSSSDIYANELRSIFWAGPTG---------------------LRDAVERRGRPPGPILSGTGFCVRRDAV 372

TRIAE_CS42_3B_TGACv1 YGALPASSQDQ-FSGVEVGELKRRFGVSNGHIASLRRPGTGSTIVARDALP---QDAELVACCDYETGTEWGEEVGFLYQ 447
TRIAE_CS42_3DS_TGACv YGARPASSQDQ-FSGVEVGELKRRFGVSNGHIASLRRSGTGSTIVARDALPQ--EDAELVASCAYETGTEWGEQVGFLYQ 448
TRIAE_CS42_3AS_TGACv YGAGPGSSQEQ-FSGVEVGELKRRFGVSNGHIASLRRSGTGSTIVAAGDVLP--QDAELVASCDYETGTEWGEDVGFLYQ 477
TRIAE_CS42_3DS_TGACv YGAGPGSSQDHQSSGVEVGELKRRFGVSNGHIASLRRSGTGSTIVARDGLPQPQEDAELVASCDYETGTEWGEEVGFLYQ 452
```

```
TRIAE_CS42_3B_TGACv1 SVVEDYFTGYRQLYCRGWTSVYCFPATGSRPPFLGSVPTNLNDALVQNKRWMSGMLAVGLSRHCPLASAAAISVPESMGF 527
TRIAE_CS42_3DS_TGACv SVVEDYFTGYRQLYCRGWTSVYCFPAAASRPPFLGSVPTNLNDALVQNKRWMSGMLAVGLSRHCPLAS-AAICVPQSMGF 527
TRIAE_CS42_3AS_TGACv SVVEDYFTGYRQLYCRGWTSVYCFPATGSRPPFLGSVPTNLNDALVQNKRWMSGLLAVGLSRHCPLASAAAISVPQSMGF 557
TRIAE_CS42_3DS_TGACv SVVEDYFTGYRQLYCPGWTSVYCFPATGTRPPFLGSVPTNLNDALVQNKRWMSGMLAVGLSRHCPLASAAAVSVPQSMGF 532

TRIAE_CS42_3B_TGACv1 AYYAFMALYAFPVLCYAIVPQLCFFRGGTSFP-EASTLWFAAVFVSSSLQHLVEVSVAKRGLAARTCWNEQRFWALNAVT 606
TRIAE_CS42_3DS_TGACv AYYAFMALYAFPVLCYATVPQLCFLRGGTSFPGAASTLWFAAVFASSSLQHLVEVSVAKRGLALRTWWNEQRFWALNAVT 607
TRIAE_CS42_3AS_TGACv AYYAFTPLYAFPLLCYATVPQLCFLRGATSFPEAASTLWFAAVFASSSLQHLVEVSVAKRGLAARTWWNEQRFWALNAVT 637
TRIAE_CS42_3DS_TGACv AYYAFMALYAFPVLCYATVPQLCFLRGGTSFP-GESALWFAAVLASSSLQHLVEVSFAKRGLAARAWWNEQRFWALNAVT 611

TRIAE_CS42_3B_TGACv1 GQLFACLSVALNLVDGAGGRAVDFDLTSKASDDRLYRDGVFDFAGCSTLLLPATTLCLLNAAALVGGVWKMVGRGGNMP- 685
TRIAE_CS42_3DS_TGACv GQLFACLGVALNLVG-AGGRAVDFDLTSKASDDRLYRDGVFDFAGCTTLLLPATTLCLLNAAALVGGVWKMVGRGGSVS- 685
TRIAE_CS42_3AS_TGACv GQIFACLGVALSLVG-AGGRAVDFDLTSKASGDRLYRDGVFDFAGCSALLLPATTLCLLNAAALVGGVWKMVGRGGNVSG 716
TRIAE_CS42_3DS_TGACv GQLFACVSVALSLVG-AGGRAVDFDLTSKASDDRLYRDSVFDFAGCSALLLPATTLCLLNTAALVGGVWKMVGRGGSVS- 689

TRIAE_CS42_3B_TGACv1 -GELFLLCYIAALSYPLLQGMFLRRDLARVPARITAMSVAMVATLLSLFG 734
TRIAE_CS42_3DS_TGACv -GELFLLCYVAALSYPLLQGMFLRRDPARVPAPITAMSVAMVAALLSLFG 734
TRIAE_CS42_3AS_TGACv TGELFLLCYVAALSYPLLQGMFLRRDPARVPARITAVSVAIVATLLSLFG 766
TRIAE_CS42_3DS_TGACv -GELFLLCYVAALSYPLLEGMFLRRDPARVPAWITAMSVAMVATLLSLFG 738
```
